# Establishment of a Combined Diagnostic Model of Abdominal Aortic Aneurysm with Random Forest and Artificial Neural Network

**DOI:** 10.1155/2022/7173972

**Published:** 2022-03-07

**Authors:** Yixuan Duan, Enrui Xie, Chang Liu, Jingjing Sun, Jie Deng

**Affiliations:** Department of Cardiology, Second Affiliated Hospital of Xi'an Jiaotong University, Xi'an, China

## Abstract

Objectives. Abdominal aortic aneurysm (AAA), a disease with high mortality, is limited by the current diagnostic methods in the early screening. This study aimed to screen novel and significant biomarkers and construct a diagnostic model for AAA by using a novel machine learning method, i.e., an ensemble of the random forest (RF) algorithm and artificial neural network (ANN). Methods and Results. Through a search of the Gene Expression Omnibus (GEO) database, two large-sample gene expression datasets (GSE57691 and GSE47472) were downloaded and preprocessed. Differentially expressed genes (DEGs) in GSE57691 were identified by R software, followed by Gene Ontology (GO) and Kyoto Encyclopedia of Genes and Genomes (KEGG) enrichment using the Database for Annotation, Visualization, and Integrated Discovery (DAVID). Essential metabolic pathways related to positive regulation of cell death and NAD binding were found. Then, RF was used to identify key genes from the DEGs, and an AAA diagnostic model was established by ANN. A transcription factor (TF) regulatory network of key genes related to angiogenesis and endothelial migration was constructed. Finally, a validation dataset was used to validate the model and the area under the receiver operating characteristic curve (AUC) value was high. Conclusion. Potential AAA-associated gene biomarkers were identified by RF, and a novel early diagnostic model of AAA was established by ANN. The AUC indicated that the diagnostic model had a highly satisfactory diagnostic performance. In conclusion, this study will provide a promising theoretical basis for further clinical and experimental studies.

## 1. Introduction

AAA is a localized dilatation of the infrarenal aorta, a permanent and irreversible enlargement of the abdominal aorta to a diameter of 3 cm or larger, exceeding the normal diameter by more than 50% [1, 2]. Although it is usually asymptomatic before enlargement, AAA is naturally progressive, leading to a high risk of irreversible aneurysmal growth and unpredictable rupture at any time, which leads to a high mortality rate of up to 80% [3]. Approximately 150,000–200,000 deaths are associated with AAA worldwide every year [4]. With the aging of the population as well as the improvements in living standard and diagnostic techniques, an increasing incidence of AAA has been reported in recent years. Early diagnosis of AAA before rupture can reduce the risk of death associated with this disease. Therefore, novel diagnostic model for patients needs to be urgently established. Moreover, finding therapeutic agents that can prevent AAA growth also need genetic and basic science research to identify pivotal cell-signaling pathways involved [2].

The etiology of AAA is complex. AAA is associated with various factors, including smoking, male sex, advanced age, atherosclerosis, hyperlipidemia, race, chronic obstructive pulmonary disease, and family history [5]. It results from both genetic and environmental factors [6]. The risk of AAA nearly doubles if the patient has a family genetic history [7]. One review showed that in asymptomatic men 65 years of age and older, population-based AAA screening showed statistically significant reductions in AAA-related mortality and rupture [8]. Previous AAA studies have confirmed several susceptibility genes that can help diagnose AAA, including CTLA4, NKTR, CD8A, CANX, CD44, DAXX, STAT1, IL6R, LDLR, and STAT3 [9–13]. Therefore, searching for AAA-related biomarkers has become a necessary research direction for AAA screening and diagnosis.

Unlike traditional statistical methods, machine learning is not rule-based programming but learning from examples [14]. The most important step before choosing a machine learning approach is to predict the number of variables. In general, simple prediction tasks can be performed with traditional models (e.g., logistic regression), and complex tasks require more complex models (e.g., neural networks). Therefore, in order to establish a new diagnostic model of AAA, we chose RF combined with ANN model to learn from the data sets and then verify the diagnostic model in the validation set. With their continuous optimization, machine learning algorithms have become powerful tools for data utilization thanks to their high classification accuracy and convenient use. Among machine learning methods, random forest (RF) [15] algorithms, and artificial neural networks (ANNs) [16] have shown particularly strong computing power. This study aimed to use a novel method, i.e., an RF-ANN ensemble, for AAA risk factor screening and establishment of an AAA diagnostic model. The findings of this study provide potential biomarkers for early clinical screening of AAA.

## 2. Materials and Methods

### 2.1. Research Design

**Figure 1:**
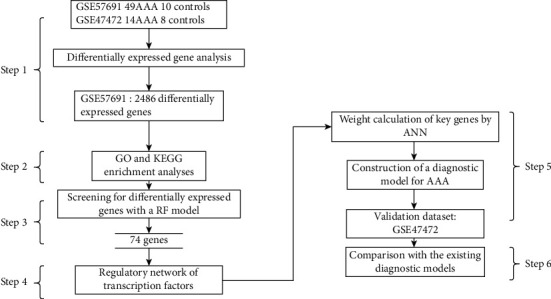
Schematic illustration of the research design.

Figure 1 is the research framework of this study. Two large-sample gene expression datasets (GSE57691 and GSE47472) were obtained through a search of the Gene Expression Omnibus database (GEO, https://www.ncbi.nlm.nih.gov/geo/) [17] with “Abdominal aortic aneurysm” as the keyword. The differentially expressed genes (DEGs) between the two sample groups (AAA and non-AAA) in each dataset were identified by using the “limma” package of R software (Step 1). Gene Ontology (GO) and Kyoto Encyclopedia of Genes and Genomes (KEGG) enrichment analyses were performed through the Database for Annotation, Visualization, and Integrated Discovery (DAVID) [18] based on the DEGs in GSE57691, followed by functional classification of these genes (Step 2). The DEGs identified in GSE57691 were subject to RF analysis using the “randomForest” package of R, through which 74 genes with a mean decrease accuracy > 0.001 and mean decrease Gini > 0.05 were determined (Step 3). Further, the genes identified by weighted gene co-correlation network analysis (WGCNA) [19] combined with the Enrichr database were used to construct a weighted gene coexpression network, and a regulatory network was then made of the genes with correlation values >0.1 and their related TFs (Step 4). A diagnostic model was constructed using the “neuralnet” [20] package based on the DEGs identified in Step 3 and was validated by in GSE47472 (Step 5). Finally, the performance of the constructed model was compared with the performance of existing diagnostic models (Step 6).

### 2.2. Data Preprocessing

The Illumina HumanHT-12 V4.0 expression beadchip data of GSE57691 and GSE47472 were downloaded from the GEO database. 59 samples (10 control samples and 49 AAA samples) in GSE57691 were selected for study. 20 small AAA samples (mean maximum diameter = 54.3 ± 2.3 mm) and 29 large AAA samples (mean maximum aortic diameter = 68.4 ± 14.3 mm) in GSE57691 were merged as AAA. More specific sample information can be queried in GEO website (GEO, https://www.ncbi.nlm.nih.gov/geo/). The constructed diagnostic model for AAA was validated using 8 control samples and 14 AAA samples obtained from GSE47472 (Table 1). The datasets downloaded from the database were normalized by Genespring GX version 11.5.1 software for luminal single-color arrays. Then the probe ID was converted into gene symbols through R software. After mapping the probes to genes, the unidentifiable probes were removed. If multiple probes could be mapped to the same gene, the expression level of the gene was represented by the maximum mean expression value for subsequent analysis.

### 2.3. Screening for DEGs

The “limma” [21] package of R software was used to analyze DEGs in the GSE57691 and GSE47472 datasets, with FDR < 0.001 as a threshold. Volcano plots and heatmaps were, respectively, visualized with the “ggplot2” [22] and “pheatmap” [23] packages in R software.

### 2.4. Functional Enrichment Analysis of DEGs

To further understand the function of DEGs, they were subject to GO enrichment, which categorizes genes into biological process (BP), cellular component (CC), and molecular function (MF) [24, 25]. Moreover, KEGG analysis [26] was used to describe metabolic pathways, using DAVID 6.8 (https://david.ncifcrf.gov/home.jisp). The results were visualized by R software.

### 2.5. RF Analysis to Further Screen DEGs

The DEGs in GSE57691 were further screened with the “randomForest” package in R. First, we conducted cyclical computing and obtained the out-of-bag (OOB) error rates when using different numbers of DEGs as a variable number, through which the optimal number of variables was determined based on the lowest OOB error. The OOB errors when the number of trees ranged from 1 to 3000 were calculated, and the optimal number of decision trees was determined by considering both OOB error and stability. Finally, based on the parameters determined, an RF model was constructed, and the candidate genes for AAA diagnosis were determined according to the mean decrease accuracy and mean decrease Gini.

### 2.6. Construction of a Transcription Factor Regulatory Network

The “WGCNA” package in R was applied to calculate pairwise correlations between genes identified from the RF screening. The relevant genes with correlation values >0.1 were subject to transcription factors (TF)-mRNA regulatory relationship analysis using the Enrichr database (http://amp.pharm.mssm.edu/Enrichr/), through which the TFs that regulated the DEGs were identified for cytoscape-aided construction of a TF regulatory network.

### 2.7. Construction and Validation of an a NN Model

GSE57691 was used for training, and GSE47472 was used for validation. According to the DEGs selected by RF, an ANN model was constructed by the “neuralnet” package of R based on the training dataset. The model was validated in the validation dataset, and its diagnostic performance was assessed by calculating the area under the AUC.

## 3. Results

### 3.1. Screening of DEGs

**Figure 2:**
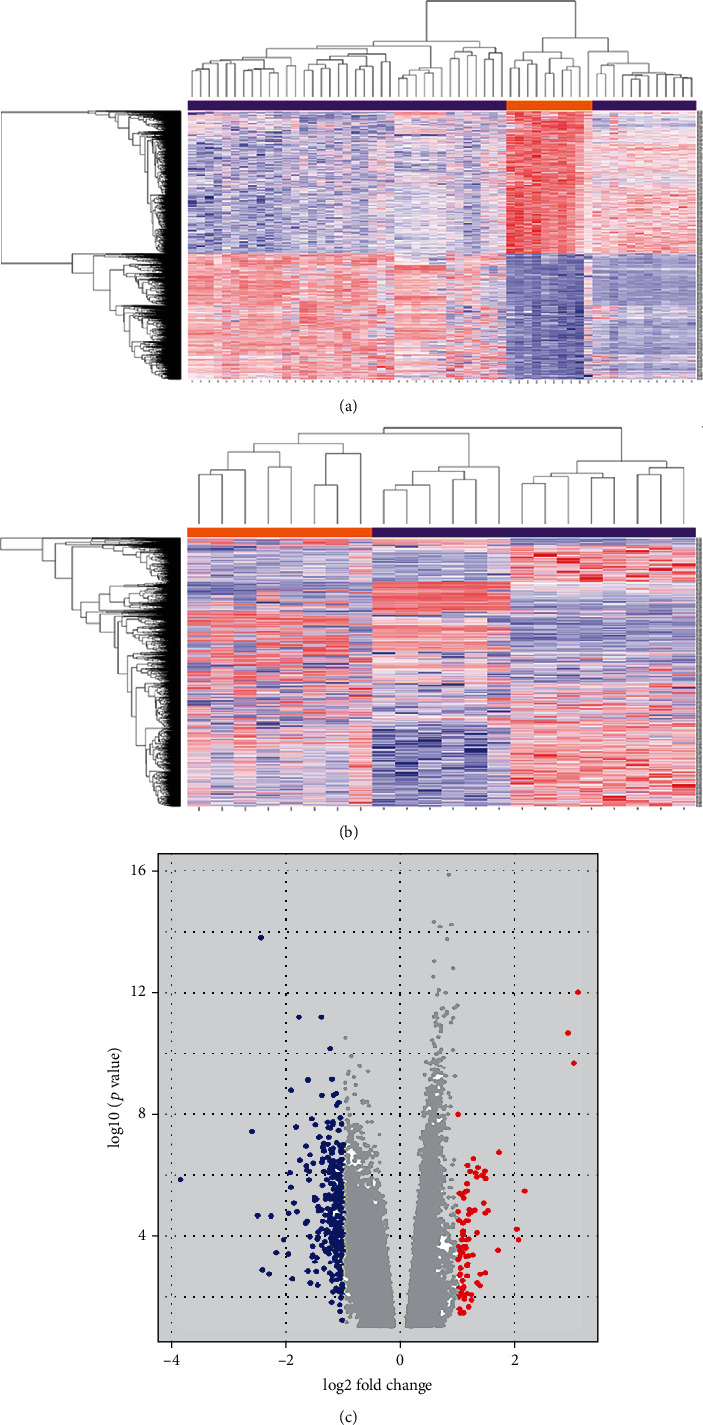
Screening of DEGs in the datasets. (a) The heatmap of 2486 DEGs in GSE57691, which was derived from clustering analysis of gene expression data in 49 AAA and 10 control samples. (b) The heatmap of 1464 DEGs in GSE47472, which was derived from clustering analysis of gene expression data in 14 AAA and 8 control samples. (c) Volcano plots demonstrated the distribution of DEGs in GSE57691. The *x*-axis shows −log10 (*p* value), the *y*-axis refers to |log2 (fold change)|, and the cutoff value is |log2 fold change| ≥1.

Differential expression analysis derived 2486 DEGs in GSE57691 and 1464 DEGs in GSE47472 when using false discovery rate (FDR) < 0.001 as the threshold (Supplementary Table 1 and Supplementary Table 2). There were 178 same DEGs shared by both datasets.  Figure 2(a) and Figure 2(b) were the heatmaps of DEGs in GSE57691 and GSE47472, respectively. Both heatmaps showed satisfactory separation of gene expression.  Figure 2(c) shows the volcano plot of average gene expression levels. Genes mentioned before such as IL6R, and STAT1 were identified.

### 3.2. GO and KEGG Enrichment Analysis of DEGs

**Figure 3:**
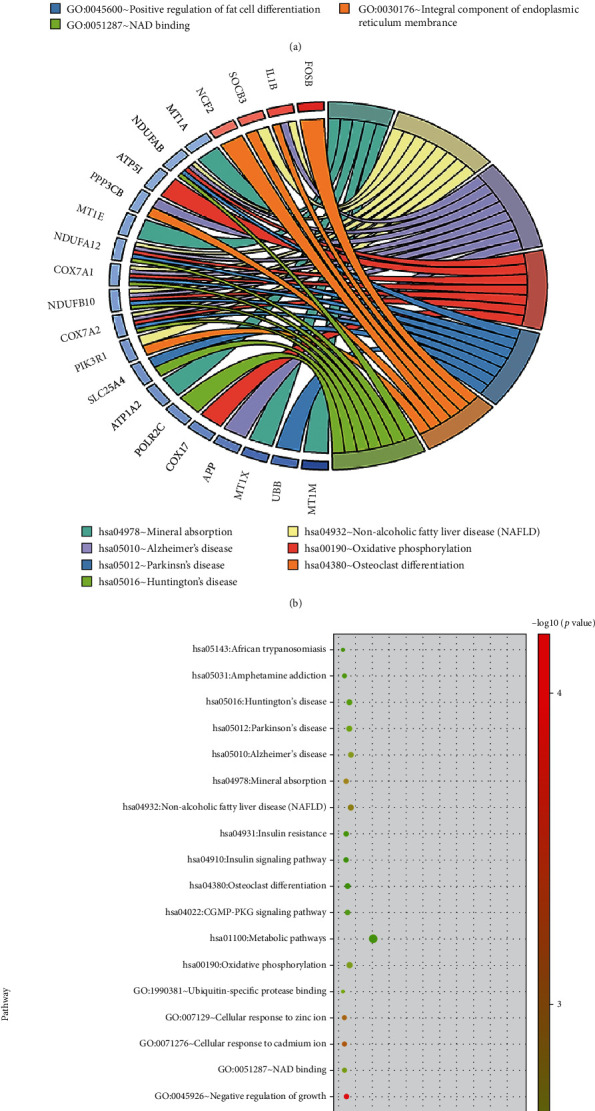
Functional analysis and visualization of 2486 DEGs in DAVID. (a) Circle diagram of enriched GO functional clusters. (b) Circle diagram of enriched KEGG pathways. (c) Functional enrichment bubble diagram of the 2486 DEGs.

All 2486 DEGs were imported into DAVID 6.8 for functional enrichment analysis (Supplementary Table 3 and Supplementary Table 4). GO analysis of the DEGs yielded 86 enriched annotations, including 52 BP, 17 CC, and 17 MF, as well as 13 KEGG enriched pathways. We find that genes such as HBA2, HBB, UCP2 and ZC3H12A enriched in the pathway of positive regulation of cell death were significantly upregulated. Besides, significantly downregulated genes, such as MT1M, MT1X, and GPD1L, are enriched in the pathway of cellular response to cadmium ion, cellular response to zinc ion, and NAD binding (Figure 3(a)). In KEGG pathway analysis, significantly downregulated genes are related with neurodegenerative diseases such as Alzheimer's disease, Huntington's disease, and Parkinson's disease (Figure 3(b)). The biological process of clustering was consistent with the pathophysiological mechanism of AAA.

### 3.3. Diagnostic Feature Genes Identified and Classified by RF

**Figure 4:**
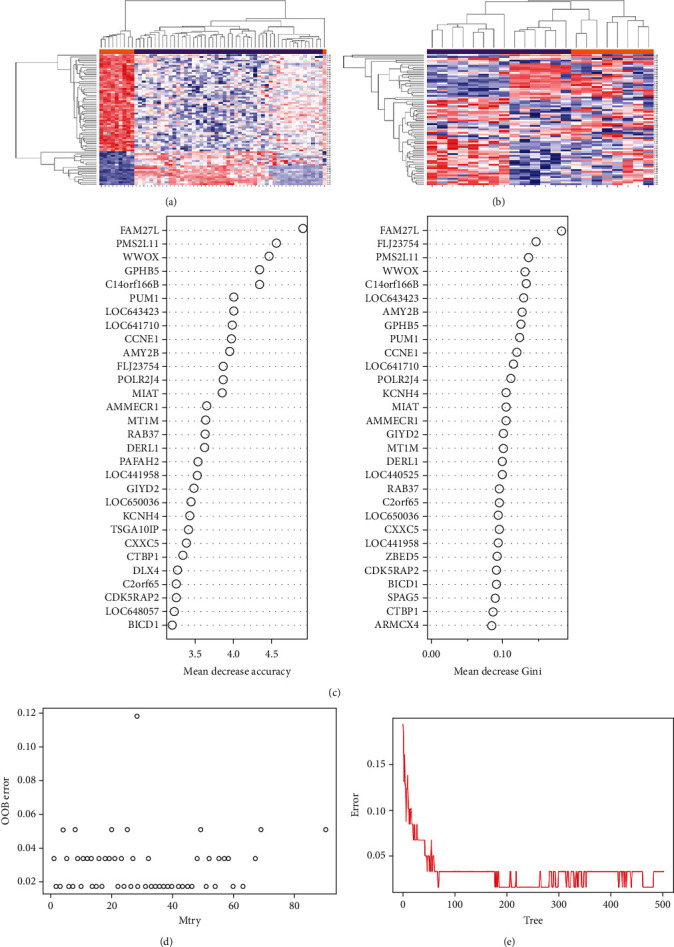
Heatmaps of 74 feature genes selected by RF in GSE57691 and GSE47472. (a) Clustering of the 74 genes in GSE57691. (b) Clustering of the 74 genes in GSE47472. (c) Ranks of input variables in the RF model, based on which the genes were classified in both the AAA and control groups. (d) Determination of the optimal number of feature genes. (e) The impact of the number of decision trees on the OOB error.

To identify DEGs that were more reliable, the R package “randomForest” was used to further screen the 2486 DEGs. The classification was optimal when the number of variables was three and the optimal tree number was set at 100. A mean decrease accuracy > 0.001 and mean decrease Gini > 0.05 were key thresholds used for screening, which yielded 74 DEGs (Supplementary Table 5 and Figure 4). In the training dataset GSE57691, all 74 genes were clustered satisfactorily except in one control sample. In the validation dataset GSE47472, the clustering of these genes was fully satisfactory.

### 3.4. Construction of a Transcription Factors (TFs) Regulatory Network of Feature Genes

**Figure 5:**
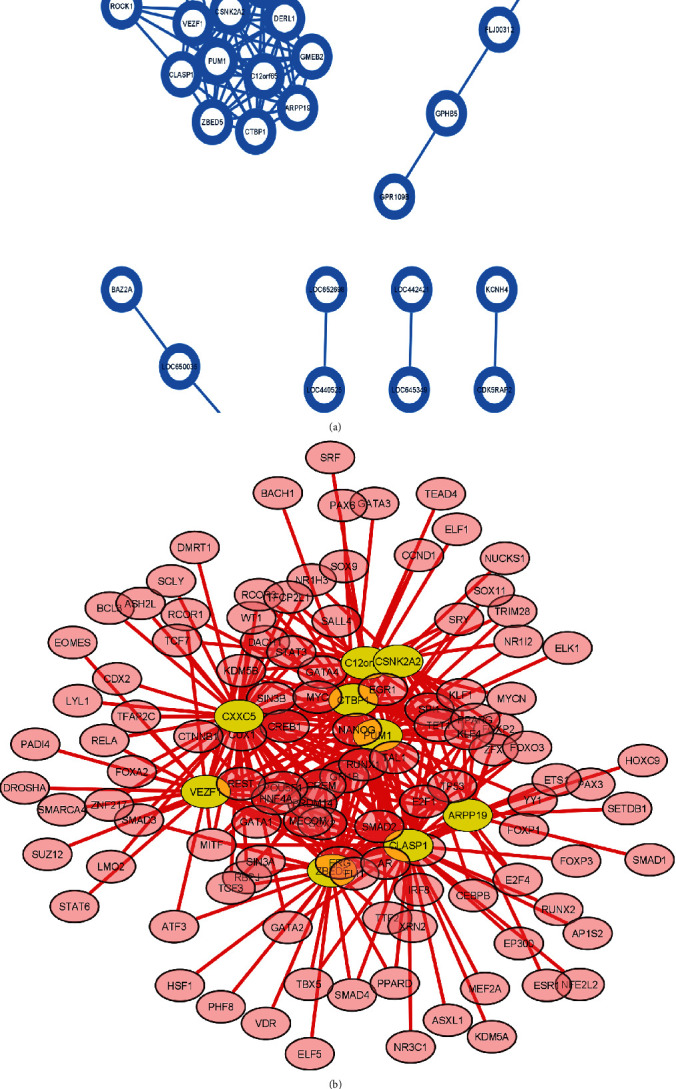
The WGCNA network and TF-mRNA network. (a) The WGCNA network. (b) 9 genes in yellow circles had correlation values > 0.1. Related gene-regulatory factors were in red circles.

The 74 feature genes selected by RF were used to construct a network (Figure 5). The 74 DEGs formed 1084 interaction pairs (Supplementary Table 6). The WGCNA package of R was used to calculate the pairwise correlations between the 74 genes and conduct the WGCNA (Figure 5(a)). The relevant genes with correlation values > 0.1 were selected to construct the TF network (Figure 5(b)).

### 3.5. Construction and Validation of the ANN Model

**Figure 6:**
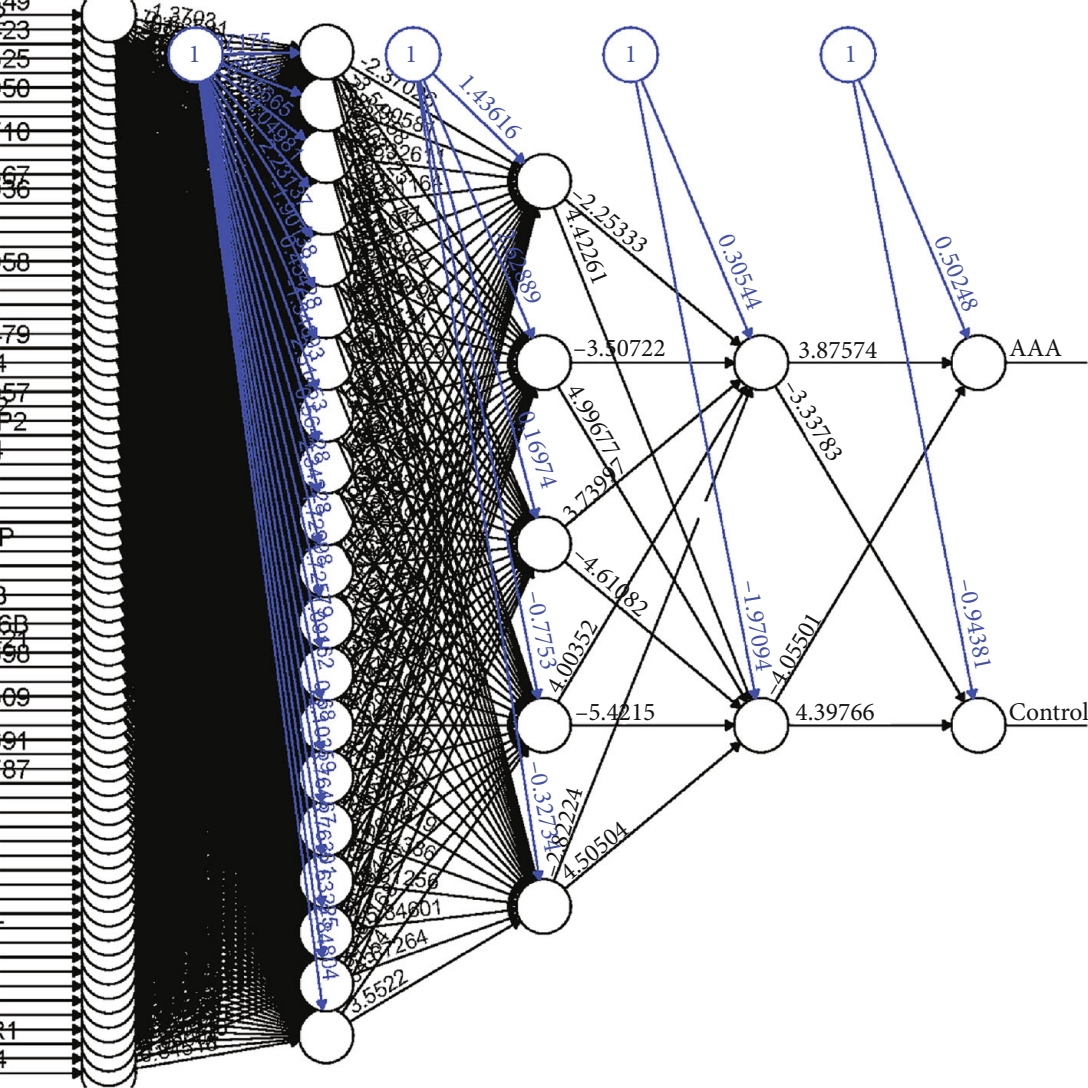
Construction of a neural network: the neural network topology of the dataset (GSE57691) with 1 input layer, 3 hidden layers, and 1 output layer.

**Figure 7:**
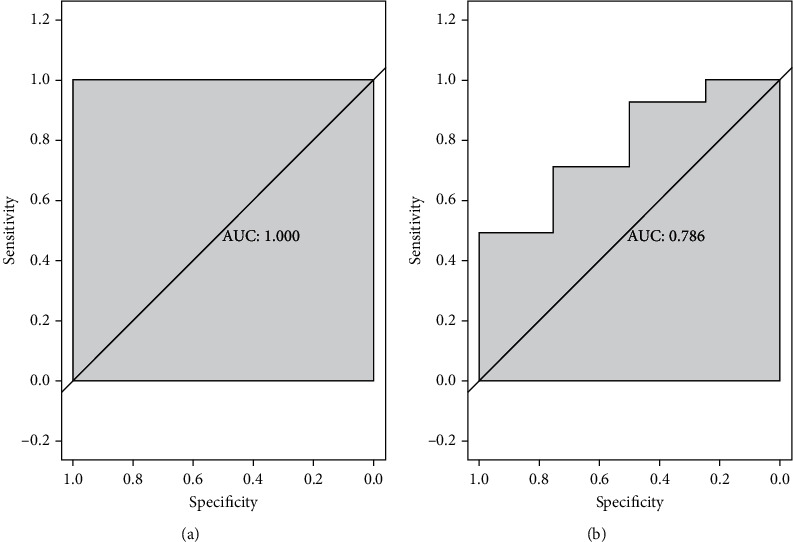
ROC curves of the ANN diagnostic model of AAA. (a) Prediction by the constructed ANN model in the GSE57691 dataset. (b) Validation of the ANN model in the GSE47472 dataset.

The 74 DEGs selected by RF were used to construct a neural network with the GSE57691 dataset. The weight of each gene was calculated for optimal differentiation between the AAA and control samples. A diagnostic model was then constructed based on the weights of the genes and the neural network (Figure 6). Prediction by the model had an AUC of 0.786 in GSE47472 and 1 in the original dataset GSE57691 (Supplementary Table 8 and Supplementary Table 9), suggesting that the ANN is highly stable in diagnosing AAA (Figure 7). These findings show that we successfully constructed an AAA diagnostic model through the differential gene expression between AAA and control samples.

## 4. Discussion

Conventionally, AAA is diagnosed based on imaging findings that confirm the presence of an aneurysm, and the first-choice imaging method for AAA screening is abdominal ultrasound. However, the accuracy of ultrasound diagnosis depends on the operator's experience and skill. Other factors such as the direction of the scanning plane, patient compliance, obesity, or intestinal gas accumulation can also significantly affect the accuracy of ultrasound diagnosis [27]. The application of computed tomography angiography and magnetic resonance angiography is limited to AAA screening due to such disadvantages as their use of contrast agents, radiation damage, and high cost. A previous study showed that early screening for AAA can reduce the AAA-associated mortality by approximately 50% in men [28]. In 2005, the guidelines on screening for AAA published by the American Heart Association recommended that men aged 60 or older who have siblings or offspring with AAA should be given a physical examination for AAA [29]. Considering the lack of effective examination methods for early AAA screening and diagnosis, as well as the lack of sensitive and specific biomarkers that can be used in clinical practice, it is crucial to develop a model for early diagnosis and screening of AAA [30].

Instead of focusing on phenotypic diagnosis, we further explore the molecular level diagnosis of AAA, and machine learning methods show great advantages in gene selection and classification. Previous machine learning studies have focused on predicting the risk of rupture in abdominal aortic aneurysms. Liang et al. [31] used a machine learning method to predict the rupture risk of ascending aortic aneurysm based on the shape characteristics of the aneurysm. This machine learning-based method was much faster than finite element analysis. Another study developed an auxiliary tool to assess the possibility of AAA rupture and predict the progression of AAA through machine learning [32]. Therefore, we proposed a new idea to combine the classification advantages of machine learning with the diagnosis of AAA to achieve the purpose of early diagnosis.

The question we are faced with is a dichotomous question whether the patient has an AAA. Machine learning offers a principled approach for developing sophisticated, automatic, and objective algorithms for analysis of high-dimensional and multimodal biomedical data [33]. The random forest algorithm is popular in the life sciences because it supports *p* ≫ *n* datasets and is robust to large amounts of noise, requires little parameter tuning, and requires no predictor transformation. Meanwhile, RF has high-prediction accuracy and provides information on importance of variables for classification [34]. Most applications of ANN to medicine are classification problems [35]. In supervised learning, network patterns are trained by providing inputs and outputs to the network at this stage, and the neural network can adjust the connection weights to match its output with the actual output in the iterative process until the result is needed. Based on the advantages mentioned before, we chose the method of random forest combined with artificial neural network to construct the diagnostic model of AAA.

This study aimed to develop a diagnostic model based on gene expression data. We used as many samples as possible from the GEO database and ensured that the samples of the selected dataset had come from the same sequencing platform GPL10558, which minimized the effect of confounding factors to a certain extent. First, 2486 DEGs were selected from the GSE57691 dataset, and then GO enrichment analysis and KEGG pathway analysis were conducted. Cell growth and death regulation were the most significantly enriched GO terms. The association of cell growth and death with aneurysm development has been investigated before. The formation of aneurysms involves chronic inflammatory cell infiltration into the tunica adventitia and tunica media along with elastin rupture, degeneration, and attenuation [36]. H19 promotes apoptosis and suppresses the proliferation of smooth muscle cells, resulting in aortic enlargement [37]. Among the KEGG pathways, we found that metabolic pathways had the most genes. A number of studies have also reported metabolic pathway changes in the aneurysmal wall compared with the normal arterial wall; for example, BAF60a deficiency in vascular smooth muscle cells can prevent the occurrence and progression of AAA by reducing inflammation and extracellular matrix degradation [38]. The findings from GO enrichment analysis and KEGG pathway analysis in the present study elucidated the pathogenesis of AAA. Further, 74 key genes were identified using the RF algorithm, providing more susceptibility genes as targets of AAA research. 9 genes (ZBED5, VEZF1, CLASP1, ARPP19, CTBP1, C12orf16, PUM1, CXXC5, and CSNK2A2) with correlation values > 0.1 were obtained through WGCNA. Among these 9 genes, vascular endothelial zinc finger 1 (VEZF1) encodes the Krüppel-like zinc finger protein, which plays an important role in vascular development and can inhibit the expression of antiangiogenic factor Cited2 in endothelial cells, providing a biological target for the diagnosis and treatment of AAA [35]. CXXC5 is a member of the CXXC zinc finger protein family. Functionally, CXXC5 mediates bMP4-induced inhibition of Wnt signaling in neural stem cells, as well as endothelial migration and angiogenesis [39]. Based on these genes with stronger correlations, a regulatory network of TFs was constructed, through which the pathogenesis underlying AAA can be further determined. Finally, using the ANN to calculate the weight of each key gene, a diagnostic model for AAA was established. The accuracy of the model was verified in an independent dataset, which had a prediction AUC of 0.786. The high AUC indicates that the constructed model reliably distinguishes AAA samples from normal samples.

Our study also has some limitations. First, it is difficult to obtain abdominal aorta specimens, which may limit the clinical application scenarios of this diagnostic model. Second, the etiology of AAA involves both genetic and environmental factors. Many environmental factors are associated with AAA, they may interfere with the diagnostic performance of our model that was constructed based on susceptibility genes. Third, the diagnosis of AAA using an ANN based on gene expression data depends highly on the source of the samples. Diagnosis based vascular samples from other locations would have a lower accuracy than diagnosis based on samples from the abdominal aortic segment in patients with AAA. Fourth, this diagnostic model will have certain significance in scientific research and can guide the clinical screening and diagnosis of AAA, prospective experimental, and clinical studies are needed to further verify the results.

## 5. Conclusion

In this study, 74 genetic biomarkers such as VEZF1 and CXXC5 associated with AAA were identified and used to construct an early AAA diagnostic model with satisfactory diagnostic performance. Meanwhile, our study provides a valuable reference for the early screening of AAA, sheds new light on the pathogenesis of AAA, and offers potential biomarkers as targets for the clinical treatment of AAA.

## Figures and Tables

**Table 1:** Source of datasets.

Dataset	Platform	AAA samples	Control samples
GSE57691	GPL10558	49	10
GSE47472	GPL10558	14	8

## Data Availability

The data used to support the findings of this study are available from the corresponding author upon request.

## Supplementary Materials

Supplementary 1 Supplementary Table 1: 2486 differentially expressed genes in GSE57691 dataset.

Supplementary 2 Supplementary Table 2: 1464 differentially expressed genes in GSE47472 dataset.

Supplementary 3 Supplementary Table 3: Significantly enriched GO terms in BP, CC, and MF from GSE57691 dataset.

Supplementary 4 Supplementary Table 4: KEGG Analysis with DEGs from GSE57691 dataset.

Supplementary 5 Supplementary Table 5: Random Forest Selected Genes.

Supplementary 6 Supplementary Table 6: TF-mRNA Network.

Supplementary 7 Supplementary Table 7: WGCNA Correlation Table.

Supplementary 8 Supplementary Table 8: ANN in GSE57691 dataset.

Supplementary 9 Supplementary Table 9: ANN in GSE47472 dataset.
