# Supplementary material for: Establishment of a Combined Diagnostic Model of Abdominal Aortic Aneurysm with Random Forest and Artificial Neural Network
Source: Biomed Res Int. 2022 Mar 7;2022:7173972. doi: 10.1155/2022/7173972 (PMC8922147; doi:10.1155/2022/7173972)
Supplement: Supplementary 1 — Supplementary Table 1: 2486 differentially expressed genes in GSE57691 dataset. [file 7173972.f1.docx]

| Supplementary Table 1. Differentially Expressed Genes in GSE57691 (2486) | | | | | | | |
| --- | --- | --- | --- | --- | --- | --- | --- |
| Symbol | Entrez | logFC | AveExpr | t | P.Value | adj.P.Val | B |
| C9orf110 | 401549 | 0.842367868 | -0.008808771 | 11.27861262 | 1.31E-16 | 5.18E-12 | 27.07294549 |
| AMY2B | 280 | 0.584443221 | -0.12115341 | 10.33724015 | 4.51E-15 | 6.26E-11 | 23.74172185 |
| AMMECR1 | 9949 | 0.894164654 | -0.106241398 | 10.28548374 | 5.49E-15 | 6.26E-11 | 23.5553181 |
| MT1M | 4499 | -2.42997476 | 0.006491834 | -10.02855987 | 1.47E-14 | 1.07E-10 | 22.62528472 |
| CDK5RAP2 | 55755 | 0.813025382 | -0.08325786 | 10.00240327 | 1.62E-14 | 1.07E-10 | 22.53017084 |
| OR4D1 | 26689 | 0.91910427 | -0.092630985 | 9.433004461 | 1.47E-13 | 7.25E-10 | 20.44117729 |
| BICD1 | 636 | 0.573484394 | -0.088714938 | 9.272539532 | 2.75E-13 | 1.20E-09 | 19.84651387 |
| BAZ2A | 11176 | 0.670213641 | -0.055125195 | 9.017402399 | 7.47E-13 | 2.95E-09 | 18.89618422 |
| HBA2 | 3040 | 3.096343812 | 0.092451692 | 8.97063391 | 8.98E-13 | 3.04E-09 | 18.72137973 |
| TSGA10IP | 254187 | 0.791182912 | -0.10780884 | 8.963449827 | 9.24E-13 | 3.04E-09 | 18.69451235 |
| SCN5A | 6331 | 0.640702638 | -0.048180606 | 8.927069458 | 1.07E-12 | 3.23E-09 | 18.55839183 |
| LILRA5 | 353514 | 0.992572754 | 0.000846421 | 8.718791255 | 2.42E-12 | 6.82E-09 | 17.77714832 |
| CABP5 | 56344 | 0.9417517 | -0.124917317 | 8.671960441 | 2.91E-12 | 7.14E-09 | 17.60105525 |
| LOC641710 | 641710 | 0.717855036 | -0.054070415 | 8.658105073 | 3.08E-12 | 7.14E-09 | 17.54892758 |
| LOC644133 | 644133 | 0.808589331 | -0.08682111 | 8.598297354 | 3.90E-12 | 8.54E-09 | 17.32376803 |
| CCNE1 | 898 | 0.6211169 | -0.004124323 | 8.568528071 | 4.39E-12 | 9.10E-09 | 17.2116083 |
| ERVWE1 | 30816 | 0.760385333 | -0.098784556 | 8.519890295 | 5.32E-12 | 9.38E-09 | 17.02823939 |
| LOC650036 | 650036 | 0.58590054 | -0.096509072 | 8.501345823 | 5.72E-12 | 9.38E-09 | 16.9582869 |
| CXXC5 | 51523 | -1.773566773 | -0.040828818 | -8.496146065 | 5.84E-12 | 9.38E-09 | 16.93866894 |
| AGER | 177 | 0.91860834 | -0.008147844 | 8.482559331 | 6.16E-12 | 9.38E-09 | 16.88740051 |
| RAB37 | 326624 | 0.64134779 | -0.041838229 | 8.481576968 | 6.19E-12 | 9.38E-09 | 16.88369322 |
| LOC440525 | 440525 | 0.883586671 | -0.035859573 | 8.393581297 | 8.77E-12 | 1.23E-08 | 16.5513864 |
| PRDM10 | 56980 | 0.693670074 | -0.035739159 | 8.345567969 | 1.06E-11 | 1.44E-08 | 16.36988924 |
| LOC645349 | 645349 | 0.709924037 | -0.125065737 | 8.301378149 | 1.26E-11 | 1.61E-08 | 16.20273974 |
| HBB | 3043 | 2.919893002 | 0.045929485 | 8.197494283 | 1.91E-11 | 2.28E-08 | 15.80942316 |
| FLJ23754 | 201252 | 0.663470275 | 0.001382771 | 8.121536456 | 2.58E-11 | 2.99E-08 | 15.52153524 |
| ARL16 | 339231 | -0.966393582 | 0.000486054 | -8.107259272 | 2.73E-11 | 3.08E-08 | 15.46739707 |
| LOC646491 | 646491 | 0.557152763 | -0.033585349 | 8.060586019 | 3.29E-11 | 3.60E-08 | 15.29036096 |
| AGAP7 | 653268 | 0.584572437 | -0.030085012 | 7.993580855 | 4.29E-11 | 4.57E-08 | 15.03606869 |
| SPAG5 | 10615 | 0.60330906 | -0.020110187 | 7.942055199 | 5.27E-11 | 5.47E-08 | 14.84042454 |
| MFHAS1 | 9258 | -1.231082875 | -0.045203825 | -7.902139542 | 6.18E-11 | 6.24E-08 | 14.68881092 |
| RNASEN | 29102 | 0.712802049 | -0.129001962 | 7.883523272 | 6.65E-11 | 6.55E-08 | 14.61808526 |
| LOC648057 | 648057 | 0.916968877 | -0.0547723 | 7.86964102 | 7.03E-11 | 6.76E-08 | 14.56533909 |
| ANKZF1 | 55139 | 0.684422651 | -0.010353001 | 7.821925807 | 8.50E-11 | 7.98E-08 | 14.38400868 |
| LOC653867 | 653867 | 0.626876739 | -0.016543873 | 7.797781234 | 9.35E-11 | 8.58E-08 | 14.29223415 |
| TBXA2R | 6915 | -0.863511433 | -0.194918549 | -7.760249281 | 1.09E-10 | 9.73E-08 | 14.14955132 |
| CHKA | 1119 | 0.749371238 | -0.066941685 | 7.731859045 | 1.22E-10 | 1.07E-07 | 14.04160579 |
| CLEC10A | 10462 | 0.670224985 | -0.03088885 | 7.701914901 | 1.37E-10 | 1.15E-07 | 13.92773874 |
| GPHB5 | 122876 | 0.696556134 | -0.002411503 | 7.687422598 | 1.45E-10 | 1.19E-07 | 13.87262525 |
| LOC338829 | 338829 | 0.671492425 | -0.076088845 | 7.66797981 | 1.57E-10 | 1.26E-07 | 13.79868134 |
| FAM27L | 284123 | 0.579570264 | -0.06591917 | 7.656137113 | 1.64E-10 | 1.30E-07 | 13.7536397 |
| POLR2J4 | 84820 | 0.539412457 | -0.008478514 | 7.639523274 | 1.76E-10 | 1.33E-07 | 13.6904495 |
| DHRS12 | 79758 | 0.524937071 | 0.009555332 | 7.617006582 | 1.92E-10 | 1.40E-07 | 13.60480414 |
| GPRC5C | 55890 | 0.284037675 | 0.052429751 | 4.446252481 | 3.73E-05 | 0.000637336 | 2.005049181 |
| TRY1 | 136541 | 0.687711418 | -0.142557262 | 7.568268491 | 2.33E-10 | 1.61E-07 | 13.41941041 |
| ADCK4 | 79934 | -0.717703334 | -0.0197561 | -7.5429024 | 2.58E-10 | 1.75E-07 | 13.32291676 |
| BCR | 613 | 0.755476871 | -0.124393118 | 7.526094857 | 2.76E-10 | 1.84E-07 | 13.25897937 |
| DLEC1 | 9940 | 0.601720279 | -0.060169952 | 7.480104541 | 3.31E-10 | 2.15E-07 | 13.08402795 |
| MGAM | 8972 | 0.602563578 | -0.070049475 | 7.478732161 | 3.33E-10 | 2.15E-07 | 13.07880734 |
| CCR9 | 10803 | 0.551592189 | -0.138260452 | 7.473862126 | 3.40E-10 | 2.16E-07 | 13.06028148 |
| ADCY2 | 108 | 0.65311197 | -0.013369988 | 7.46855127 | 3.47E-10 | 2.17E-07 | 13.0400788 |
| ABI2 | 10152 | -0.57874938 | -0.164898752 | -7.438671384 | 3.91E-10 | 2.38E-07 | 12.92641678 |
| DLX4 | 1748 | 0.611641523 | -0.072538188 | 7.436926146 | 3.94E-10 | 2.38E-07 | 12.91977809 |
| MRPL24 | 79590 | -0.888542821 | -0.334135768 | -4.79980432 | 1.05E-05 | 0.000272413 | 3.198433761 |
| SLMO1 | 10650 | 0.631052536 | -0.054525323 | 7.423682839 | 4.15E-10 | 2.44E-07 | 12.86940265 |
| IRF6 | 3664 | 0.706388979 | -0.157587339 | 7.410408019 | 4.37E-10 | 2.54E-07 | 12.81890852 |
| GNB4 | 59345 | -0.925169117 | -0.067095494 | -7.388791993 | 4.77E-10 | 2.72E-07 | 12.73668951 |
| LOC149950 | 149950 | 0.575221748 | -0.027586837 | 7.37176621 | 5.10E-10 | 2.83E-07 | 12.67193306 |
| LOC648823 | 648823 | 0.777648149 | -0.053768443 | 7.362096168 | 5.30E-10 | 2.90E-07 | 12.63515504 |
| BCAS4 | 55653 | 0.788963162 | -0.061743195 | 7.351928941 | 5.52E-10 | 2.94E-07 | 12.59648723 |
| ACTR3B | 57180 | 0.793229873 | -0.106634672 | 7.351895179 | 5.52E-10 | 2.94E-07 | 12.59635883 |
| MRAS | 22808 | 0.949987939 | -0.031995778 | 7.348926384 | 5.59E-10 | 2.94E-07 | 12.5850682 |
| ROCK1 | 6093 | -0.818080358 | -0.092729459 | -7.330756718 | 6.01E-10 | 3.12E-07 | 12.51596958 |
| MAP3K6 | 9064 | -1.201710855 | -0.026737665 | -7.290400274 | 7.05E-10 | 3.56E-07 | 12.36251244 |
| PMS2L11 | 441263 | 0.516942712 | -0.033954266 | 7.286146206 | 7.17E-10 | 3.58E-07 | 12.34633769 |
| MCAM | 4162 | -1.618174525 | -0.035346276 | -7.278195126 | 7.40E-10 | 3.62E-07 | 12.31610706 |
| C14orf166B | 145497 | 0.605889734 | -0.08113346 | 7.253994297 | 8.15E-10 | 3.87E-07 | 12.22410077 |
| LOC643423 | 643423 | 0.766175074 | 0.025142624 | 7.240668221 | 8.59E-10 | 4.03E-07 | 12.17344284 |
| UGCGL2 | 55757 | -0.971875824 | 0.006562832 | -7.224949554 | 9.15E-10 | 4.24E-07 | 12.11369448 |
| RAB27A | 5873 | -0.789262968 | -0.118821851 | -7.184378851 | 1.08E-09 | 4.87E-07 | 11.95950702 |
| NIN | 51199 | 0.715448394 | -0.090936695 | 7.174851939 | 1.12E-09 | 5.00E-07 | 11.92330623 |
| C10orf93 | 255352 | 0.550919188 | -0.124318585 | 7.16769666 | 1.15E-09 | 5.09E-07 | 11.89611886 |
| LOC653521 | 653521 | 0.605889968 | -0.132013683 | 7.156447486 | 1.20E-09 | 5.26E-07 | 11.85337901 |
| 5-Mar | 54708 | 0.52462147 | -0.040903543 | 7.13964104 | 1.28E-09 | 5.56E-07 | 11.78953161 |
| LOC644898 | 644898 | 0.468982968 | -0.092760214 | 7.117280404 | 1.40E-09 | 5.98E-07 | 11.70459681 |
| KLF15 | 28999 | -0.969638897 | -0.112688996 | -7.116226203 | 1.41E-09 | 5.98E-07 | 11.7005929 |
| GRIPAP1 | 56850 | 0.608483794 | -0.098544938 | 7.109639292 | 1.45E-09 | 6.00E-07 | 11.67557628 |
| LOC442421 | 442421 | 0.730333909 | -0.043853619 | 7.107933906 | 1.46E-09 | 6.00E-07 | 11.66909956 |
| ZNF331 | 55422 | 0.601129262 | -0.000340065 | 7.107237283 | 1.46E-09 | 6.00E-07 | 11.66645396 |
| TM2D3 | 80213 | 0.60160898 | -0.0837972 | 7.101067032 | 1.50E-09 | 6.03E-07 | 11.6430215 |
| ZBTB16 | 7704 | -1.825989897 | -0.200393871 | -6.383836495 | 2.55E-08 | 4.59E-06 | 8.933188673 |
| GPR109B | 8843 | 0.559879448 | 0.039922049 | 7.076104498 | 1.65E-09 | 6.52E-07 | 11.54823537 |
| OR4X1 | 390113 | 0.655795808 | -0.014644376 | 7.070476746 | 1.69E-09 | 6.60E-07 | 11.52686896 |
| NEB | 4703 | 0.592988651 | -0.062272352 | 7.058941725 | 1.77E-09 | 6.71E-07 | 11.48307842 |
| LOC643577 | 643577 | 0.458071339 | -0.016362178 | 7.032704271 | 1.96E-09 | 7.38E-07 | 11.38349094 |
| LOC643272 | 643272 | 0.834602245 | -0.091920215 | 7.028885516 | 1.99E-09 | 7.42E-07 | 11.36899854 |
| LOC653136 | 653136 | 0.719176609 | -0.029136816 | 7.023209445 | 2.04E-09 | 7.50E-07 | 11.34745858 |
| NXPH3 | 11248 | -1.127259239 | -0.156685153 | -7.021383148 | 2.05E-09 | 7.50E-07 | 11.34052828 |
| DHX40 | 79665 | 0.454602067 | -0.064256985 | 7.005035129 | 2.19E-09 | 7.87E-07 | 11.27849793 |
| WDR55 | 54853 | -0.804704197 | -0.069636078 | -7.004577254 | 2.20E-09 | 7.87E-07 | 11.27676074 |
| CLASP1 | 23332 | -1.174105537 | -0.114996822 | -6.990372675 | 2.32E-09 | 8.25E-07 | 11.22287248 |
| SYNC1 | 81493 | -1.373084136 | 0.056730095 | -6.987488654 | 2.35E-09 | 8.27E-07 | 11.21193233 |
| LOC402483 | 402483 | 0.563516863 | -0.129224608 | 6.979381801 | 2.43E-09 | 8.47E-07 | 11.18118194 |
| FOXK2 | 3607 | 0.509885997 | -0.103598686 | 6.952450372 | 2.70E-09 | 9.26E-07 | 11.07904794 |
| OR2B3P | 442184 | 0.669849412 | -0.075532041 | 6.947120904 | 2.76E-09 | 9.33E-07 | 11.05884046 |
| SIM2 | 6493 | 0.87099811 | -0.095906606 | 6.946118928 | 2.77E-09 | 9.33E-07 | 11.05504146 |
| BRI3BP | 140707 | 0.531548586 | -0.085335855 | 6.932429401 | 2.92E-09 | 9.69E-07 | 11.0031422 |
| SPRY1 | 10252 | 0.696072581 | -0.077806573 | 6.930130168 | 2.95E-09 | 9.70E-07 | 10.99442629 |
| LOC644530 | 644530 | 0.598231583 | -0.077833648 | 6.927734403 | 2.98E-09 | 9.71E-07 | 10.98534471 |
| LRRC37A2 | 474170 | 0.582992328 | -0.131255494 | 6.914432178 | 3.14E-09 | 1.01E-06 | 10.93492524 |
| LOC646064 | 646064 | 0.579294502 | -0.106591527 | 6.904594432 | 3.27E-09 | 1.05E-06 | 10.89764273 |
| NLRP12 | 91662 | 0.587326219 | -0.113742145 | 6.896794735 | 3.37E-09 | 1.07E-06 | 10.86808732 |
| CDKN2B | 1030 | 0.645251927 | -0.126730982 | 6.885479816 | 3.52E-09 | 1.09E-06 | 10.82521712 |
| ICA1 | 3382 | 0.470910891 | -0.058869401 | 6.884598314 | 3.53E-09 | 1.09E-06 | 10.82187754 |
| NAIP | 4671 | 0.548176693 | -0.100494592 | 6.88312098 | 3.56E-09 | 1.09E-06 | 10.81628074 |
| ATG12 | 9140 | -0.667133561 | -0.114805351 | -6.881579117 | 3.58E-09 | 1.09E-06 | 10.81043959 |
| KIAA1632 | 57724 | 0.604034535 | -0.048130305 | 6.879277413 | 3.61E-09 | 1.09E-06 | 10.80172011 |
| TUFM | 7284 | -0.953684284 | -0.069681919 | -6.875309429 | 3.67E-09 | 1.10E-06 | 10.78668896 |
| ENDOG | 2021 | -0.595988535 | -0.096523936 | -6.870044933 | 3.74E-09 | 1.11E-06 | 10.76674775 |
| LOC728226 | 728226 | 0.694623582 | -0.174888322 | 6.860526703 | 3.89E-09 | 1.14E-06 | 10.73069769 |
| CCDC107 | 203260 | -1.083795619 | -0.073264371 | -6.850545885 | 4.05E-09 | 1.18E-06 | 10.69290082 |
| RYR2 | 6262 | -0.662538948 | -0.169668968 | -6.836730905 | 4.27E-09 | 1.22E-06 | 10.64059316 |
| MSH5 | 4439 | 0.629302332 | -0.034283439 | 6.836201918 | 4.28E-09 | 1.22E-06 | 10.63859046 |
| INVS | 27130 | 0.663697024 | -0.061245437 | 6.820546995 | 4.56E-09 | 1.28E-06 | 10.57932959 |
| DDX11 | 1663 | 0.654092464 | -0.106451454 | 6.819596787 | 4.57E-09 | 1.28E-06 | 10.57573308 |
| GCC2 | 9648 | 0.478090942 | -0.055674069 | 6.811013861 | 4.73E-09 | 1.30E-06 | 10.54324929 |
| LOC646426 | 646426 | 0.677177843 | -0.074659025 | 6.809131514 | 4.77E-09 | 1.31E-06 | 10.53612575 |
| DUX3 | 26582 | 0.68773911 | -0.073684155 | 6.799906219 | 4.94E-09 | 1.34E-06 | 10.5012166 |
| CTBP1 | 1487 | -1.120577959 | -0.136059331 | -6.798964512 | 4.96E-09 | 1.34E-06 | 10.49765339 |
| VPS16 | 64601 | 0.564509098 | -0.123987865 | 6.795065425 | 5.04E-09 | 1.35E-06 | 10.48290071 |
| GCAT | 23464 | -0.56474276 | -0.085734443 | -6.787469741 | 5.19E-09 | 1.38E-06 | 10.4541641 |
| FLJ00312 | 399761 | 0.614028489 | 0.014517235 | 6.784686952 | 5.25E-09 | 1.39E-06 | 10.4436369 |
| HAL | 3034 | 0.789147595 | -0.110678917 | 6.77729966 | 5.41E-09 | 1.42E-06 | 10.4156933 |
| HCG27 | 253018 | 0.566662325 | -0.088386682 | 6.77354375 | 5.49E-09 | 1.43E-06 | 10.40148726 |
| LOC647295 | 647295 | 0.877310752 | -0.14472521 | 6.77268499 | 5.51E-09 | 1.43E-06 | 10.39823928 |
| CHAT | 1103 | 0.569356956 | 0.027209605 | 6.767301612 | 5.63E-09 | 1.44E-06 | 10.37787944 |
| ZNF699 | 374879 | 0.424017975 | -0.064504021 | 6.748688591 | 6.06E-09 | 1.54E-06 | 10.30749937 |
| ZHX3 | 23051 | -0.922064197 | -0.070450986 | -6.731635194 | 6.48E-09 | 1.63E-06 | 10.24303608 |
| LOC93349 | 93349 | 0.759124673 | -0.066148082 | 6.722994435 | 6.70E-09 | 1.67E-06 | 10.21038055 |
| LOC651860 | 651860 | 0.624871578 | -0.109894928 | 6.716839415 | 6.87E-09 | 1.70E-06 | 10.18712226 |
| NFRKB | 4798 | 0.560647291 | 0.053285277 | 6.681884298 | 7.89E-09 | 1.93E-06 | 10.05508449 |
| GSR | 2936 | 0.503103109 | -0.012651021 | 6.672856686 | 8.17E-09 | 1.99E-06 | 10.02099786 |
| NEK11 | 79858 | 0.505520227 | 0.065555452 | 6.668102522 | 8.33E-09 | 2.00E-06 | 10.00304934 |
| LOC647645 | 647645 | 0.40705563 | -0.079212533 | 6.664218572 | 8.46E-09 | 2.01E-06 | 9.988387363 |
| LOC642691 | 642691 | 0.54632802 | -0.009552739 | 6.663889106 | 8.47E-09 | 2.01E-06 | 9.987143673 |
| LOC392713 | 392713 | 0.563786847 | -0.065001644 | 6.659171616 | 8.63E-09 | 2.04E-06 | 9.969336649 |
| DNMT3A | 1788 | 0.547163379 | -0.11326407 | 6.64883038 | 8.99E-09 | 2.11E-06 | 9.930307458 |
| LOC643738 | 643738 | 0.468670762 | -0.06158746 | 6.646446609 | 9.07E-09 | 2.12E-06 | 9.921311905 |
| C19orf46 | 163183 | 0.574762251 | -0.042540238 | 6.641153455 | 9.26E-09 | 2.15E-06 | 9.901338824 |
| LOC391509 | 391509 | 0.602066175 | -0.002121856 | 6.638247305 | 9.37E-09 | 2.15E-06 | 9.890373703 |
| LOC283710 | 283710 | 0.59412029 | -0.073934179 | 6.637389038 | 9.40E-09 | 2.15E-06 | 9.887135519 |
| LOC389816 | 389816 | 0.828350846 | -0.050093127 | 6.629624653 | 9.69E-09 | 2.21E-06 | 9.857843524 |
| LOC641996 | 641996 | 1.006258021 | -0.017882368 | 6.62555559 | 9.85E-09 | 2.23E-06 | 9.842494353 |
| ELK1 | 2002 | -0.892924136 | -0.103775015 | -6.615434987 | 1.03E-08 | 2.31E-06 | 9.80432327 |
| WFDC2 | 10406 | 0.564108326 | -0.03107156 | 6.609722101 | 1.05E-08 | 2.35E-06 | 9.782779906 |
| ANO9 | 338440 | 0.743715141 | -0.027393367 | 6.602564661 | 1.08E-08 | 2.40E-06 | 9.75579269 |
| LOC642451 | 642451 | 0.539190175 | -0.118003111 | 6.590734811 | 1.13E-08 | 2.48E-06 | 9.711196917 |
| CNTNAP4 | 85445 | 0.530646689 | -0.069202697 | 6.582709024 | 1.17E-08 | 2.52E-06 | 9.680947902 |
| LOC649151 | 649151 | 0.668928406 | -0.049706641 | 6.582236896 | 1.17E-08 | 2.52E-06 | 9.67916862 |
| ASAM | 79827 | -1.055754965 | 0.014886113 | -6.55988414 | 1.28E-08 | 2.72E-06 | 9.594949882 |
| ACTL7B | 10880 | 0.53529399 | -0.087785581 | 6.557661327 | 1.29E-08 | 2.73E-06 | 9.586577204 |
| SNHG5 | 387066 | -1.550819322 | -0.178672208 | -6.541943268 | 1.37E-08 | 2.87E-06 | 9.527383678 |
| GJB3 | 2707 | 0.521433048 | -0.051156117 | 6.527723588 | 1.45E-08 | 3.02E-06 | 9.473850958 |
| C20orf20 | 55257 | -0.93629563 | -0.111107106 | -6.51803739 | 1.51E-08 | 3.11E-06 | 9.437395334 |
| DST | 667 | 0.596606953 | -0.112375384 | 6.500246261 | 1.61E-08 | 3.31E-06 | 9.37045676 |
| MGC24125 | 439935 | 0.509546513 | -0.11702165 | 6.485187537 | 1.71E-08 | 3.48E-06 | 9.313820711 |
| CHMP4C | 92421 | -0.58467847 | -0.171505075 | -6.481933443 | 1.73E-08 | 3.48E-06 | 9.3015847 |
| CASZ1 | 54897 | -0.513604879 | -0.224906234 | -6.481625604 | 1.74E-08 | 3.48E-06 | 9.300427216 |
| DERL1 | 79139 | -1.185889309 | -0.156158389 | -6.481477431 | 1.74E-08 | 3.48E-06 | 9.299870084 |
| BTBD6 | 90135 | -0.917198827 | -0.156268356 | -6.456318943 | 1.92E-08 | 3.80E-06 | 9.20530313 |
| LOC644187 | 644187 | 0.757705193 | -0.129037134 | 6.448256016 | 1.98E-08 | 3.90E-06 | 9.175008227 |
| ANGPTL6 | 83854 | 0.722577974 | 0.047652793 | 6.442835324 | 2.02E-08 | 3.95E-06 | 9.154644453 |
| PLXNB1 | 5364 | -1.030732743 | -0.072065708 | -6.44251633 | 2.03E-08 | 3.95E-06 | 9.153446184 |
| BDKRB2 | 624 | 0.714322846 | -0.07179121 | 6.4400276 | 2.05E-08 | 3.97E-06 | 9.144097842 |
| FOXO1 | 2308 | -1.185069654 | -0.072430439 | -6.432506829 | 2.11E-08 | 4.04E-06 | 9.115851381 |
| OR8J3 | 81168 | 0.664998024 | -0.106409372 | 6.432078991 | 2.11E-08 | 4.04E-06 | 9.114244671 |
| C12orf65 | 91574 | -0.769512452 | -0.089150326 | -6.432021591 | 2.11E-08 | 4.04E-06 | 9.11402911 |
| LOC654055 | 654055 | 0.65829921 | -0.096091031 | 6.42813521 | 2.14E-08 | 4.08E-06 | 9.09943497 |
| FKBP5 | 2289 | -1.486210035 | -0.056987391 | -6.426207846 | 2.16E-08 | 4.08E-06 | 9.092197869 |
| LRRC8A | 56262 | -0.819458264 | -0.1008568 | -6.424801737 | 2.17E-08 | 4.08E-06 | 9.086918268 |
| C21orf81 | 114035 | 0.475967923 | -0.005973794 | 6.423543486 | 2.18E-08 | 4.08E-06 | 9.082193999 |
| CYorf15B | 84663 | 0.633411415 | -0.086366865 | 6.423271019 | 2.19E-08 | 4.08E-06 | 9.081171006 |
| RPL32P3 | 132241 | 0.52088084 | 0.03663722 | 6.401147331 | 2.38E-08 | 4.43E-06 | 8.998130513 |
| EDA | 1896 | 0.469723194 | -0.037181882 | 6.3989194 | 2.40E-08 | 4.45E-06 | 8.98977072 |
| C2orf65 | 130951 | 0.472766529 | -0.002812297 | 6.39761378 | 2.42E-08 | 4.45E-06 | 8.984871917 |
| POLR3H | 171568 | -0.803886211 | -0.026899423 | -6.394939035 | 2.44E-08 | 4.48E-06 | 8.974836559 |
| LOC649379 | 649379 | 0.534433947 | -0.161231252 | 6.391497699 | 2.48E-08 | 4.52E-06 | 8.961926083 |
| NSL1 | 25936 | 0.503253535 | -0.051658556 | 6.388229689 | 2.51E-08 | 4.56E-06 | 8.949666957 |
| ABCB11 | 8647 | 0.616914861 | -0.057857752 | 6.386365916 | 2.53E-08 | 4.57E-06 | 8.942675954 |
| HES6 | 55502 | -0.700342308 | -0.082463625 | -6.383073263 | 2.56E-08 | 4.59E-06 | 8.93032609 |
| SLC20A2 | 6575 | -0.900101961 | -0.031021776 | -6.381072514 | 2.58E-08 | 4.60E-06 | 8.922822347 |
| ZNF280B | 140883 | 0.476063628 | -0.000944323 | 6.379580183 | 2.59E-08 | 4.61E-06 | 8.917225673 |
| CCL15 | 6359 | 0.709632688 | -0.182139214 | 6.37844403 | 2.61E-08 | 4.61E-06 | 8.912964921 |
| ANKRD46 | 157567 | -1.264393539 | -0.159923223 | -6.373378948 | 2.66E-08 | 4.68E-06 | 8.893971666 |
| LOC642103 | 642103 | 0.608294977 | -0.125432157 | 6.370143637 | 2.69E-08 | 4.68E-06 | 8.881841133 |
| LOC653118 | 653118 | 0.78386462 | -0.150514868 | 6.367925449 | 2.72E-08 | 4.68E-06 | 8.87352484 |
| LOC441958 | 441958 | 0.481095782 | 0.025566074 | 6.367840398 | 2.72E-08 | 4.68E-06 | 8.873205985 |
| LOC644473 | 644473 | 0.637290794 | -0.08401438 | 6.367815076 | 2.72E-08 | 4.68E-06 | 8.873111051 |
| MRPS16 | 51021 | -0.963867575 | -0.150961473 | -6.365473783 | 2.74E-08 | 4.70E-06 | 8.864333794 |
| LOC649063 | 649063 | 0.622347064 | -0.061302356 | 6.364474984 | 2.75E-08 | 4.70E-06 | 8.860589575 |
| UNC84B | 25777 | -1.226146414 | -0.216239529 | -6.360632681 | 2.79E-08 | 4.75E-06 | 8.84618681 |
| LOC85390 | 85390 | 0.830680086 | 0.017691539 | 6.353784805 | 2.87E-08 | 4.86E-06 | 8.820521533 |
| PRMT1 | 3276 | -0.763309264 | -0.047388496 | -6.34626909 | 2.96E-08 | 4.98E-06 | 8.792358881 |
| RHOT1 | 55288 | 0.542062486 | -0.072496023 | 6.343267174 | 2.99E-08 | 5.02E-06 | 8.781111847 |
| C17orf38 | 146850 | 0.46281792 | -0.119110166 | 6.33822524 | 3.05E-08 | 5.10E-06 | 8.762223787 |
| LOC652288 | 652288 | 0.714362051 | -0.082634005 | 6.337221731 | 3.06E-08 | 5.10E-06 | 8.758464769 |
| AGBL5 | 60509 | -0.73425657 | -0.02620418 | -6.326095627 | 3.20E-08 | 5.30E-06 | 8.716794961 |
| LOC652565 | 652565 | 0.530298695 | -0.104578007 | 6.321321124 | 3.26E-08 | 5.38E-06 | 8.698917415 |
| HSPB2 | 3316 | -1.085235482 | -0.194017809 | -6.318183993 | 3.30E-08 | 5.42E-06 | 8.687172145 |
| LOC645447 | 645447 | 0.702215004 | -0.060880035 | 6.315696421 | 3.33E-08 | 5.44E-06 | 8.677859552 |
| FER1L5 | 90342 | 0.592349879 | -0.041293575 | 6.314080987 | 3.35E-08 | 5.44E-06 | 8.67181229 |
| N4BP1 | 9683 | 0.636709234 | -0.068066434 | 6.313795293 | 3.36E-08 | 5.44E-06 | 8.670742848 |
| WDR20 | 91833 | 0.467048967 | -0.123184079 | 5.607367501 | 5.18E-07 | 3.60E-05 | 6.060221488 |
| NR2F6 | 2063 | -0.796674618 | -0.080979519 | -6.308719253 | 3.43E-08 | 5.51E-06 | 8.65174307 |
| LOC646990 | 646990 | 0.508737693 | -0.078256545 | 6.307477748 | 3.44E-08 | 5.52E-06 | 8.647096501 |
| DEDD | 9191 | -0.820452191 | -0.009350698 | -6.30586013 | 3.46E-08 | 5.53E-06 | 8.641042516 |
| ZNF107 | 51427 | 0.507724118 | -0.037558949 | 6.299860181 | 3.55E-08 | 5.63E-06 | 8.618590025 |
| LOC440934 | 440934 | 0.657676026 | -0.097514009 | 6.299162991 | 3.56E-08 | 5.63E-06 | 8.615981315 |
| CTSZ | 1522 | -2.589356495 | -0.614303723 | -6.297103612 | 3.59E-08 | 5.65E-06 | 8.608275956 |
| LOC388755 | 388755 | 0.466410415 | -0.126319279 | 6.280731271 | 3.82E-08 | 5.98E-06 | 8.547034036 |
| MYL6B | 140465 | -0.823824183 | -0.070530735 | -6.275961988 | 3.89E-08 | 6.07E-06 | 8.529199825 |
| PKP4 | 8502 | 0.532919551 | -0.002183468 | 6.272119747 | 3.95E-08 | 6.14E-06 | 8.514834047 |
| PAIP1 | 10605 | 0.46306421 | -0.055058422 | 6.270811765 | 3.97E-08 | 6.14E-06 | 8.509944008 |
| ABHD11 | 83451 | 0.399557542 | -0.096255736 | 6.269424607 | 4.00E-08 | 6.14E-06 | 8.504758172 |
| C16orf68 | 79091 | -0.679903332 | 0.009567478 | -6.26887818 | 4.00E-08 | 6.14E-06 | 8.502715434 |
| LOC650155 | 650155 | 0.448129995 | -0.061302729 | 6.261518384 | 4.12E-08 | 6.30E-06 | 8.475205237 |
| ZNF544 | 27300 | 0.534402651 | -0.01847722 | 6.258490534 | 4.17E-08 | 6.35E-06 | 8.463889225 |
| KRT20 | 54474 | 0.588703774 | -0.075915069 | 6.239088158 | 4.50E-08 | 6.76E-06 | 8.39140162 |
| SLC7A2 | 6542 | 0.608155784 | -0.126605716 | 6.238478562 | 4.51E-08 | 6.76E-06 | 8.389124871 |
| PDGFA | 5154 | -0.879298686 | -0.141813559 | -6.236353487 | 4.55E-08 | 6.79E-06 | 8.381188361 |
| PPP4R1L | 55370 | -0.373351123 | -0.191520359 | -6.234588993 | 4.58E-08 | 6.81E-06 | 8.374598916 |
| LOC400708 | 400708 | 0.515455947 | -0.087050358 | 6.230904849 | 4.65E-08 | 6.89E-06 | 8.360841779 |
| C10orf64 | 159491 | 0.505821187 | -0.085380267 | 6.215279845 | 4.94E-08 | 7.28E-06 | 8.302513536 |
| LOC344382 | 344382 | 0.507786022 | -0.10679437 | 6.214816445 | 4.95E-08 | 7.28E-06 | 8.300784104 |
| PLA2G2D | 26279 | 0.45621433 | -0.03680419 | 6.210499559 | 5.03E-08 | 7.37E-06 | 8.284674502 |
| LOC653125 | 653125 | 0.601967285 | 0.01138313 | 6.208026462 | 5.08E-08 | 7.42E-06 | 8.275446495 |
| DDX19B | 11269 | 0.557958597 | 0.001667506 | 6.203951401 | 5.16E-08 | 7.51E-06 | 8.260242593 |
| LOC649546 | 649546 | 0.716373751 | -0.159837294 | 6.19859344 | 5.27E-08 | 7.64E-06 | 8.240255295 |
| LOC642101 | 642101 | 0.632231035 | -0.211061965 | 6.197340366 | 5.30E-08 | 7.65E-06 | 8.235581338 |
| LOC647521 | 647521 | 0.390751927 | -0.090638346 | 6.189696288 | 5.46E-08 | 7.80E-06 | 8.207073127 |
| EIF2B4 | 8890 | -0.910273954 | -0.205633061 | -6.189462469 | 5.46E-08 | 7.80E-06 | 8.206201222 |
| SH3RF3 | 344558 | -0.844516908 | -0.148822331 | -6.187784921 | 5.50E-08 | 7.83E-06 | 8.199945892 |
| TOR2A | 27433 | 0.485780646 | 0.02293402 | 6.18410228 | 5.58E-08 | 7.84E-06 | 8.186215078 |
| POLR2C | 5432 | -1.258694638 | -0.198112043 | -6.183759883 | 5.58E-08 | 7.84E-06 | 8.184938524 |
| BBS7 | 55212 | -0.739691711 | -0.061092252 | -6.183712652 | 5.59E-08 | 7.84E-06 | 8.184762435 |
| DYNLRB1 | 83658 | -1.425209649 | -0.102378753 | -6.183591049 | 5.59E-08 | 7.84E-06 | 8.18430907 |
| ACCN3 | 9311 | 0.437460925 | -0.093703759 | 6.173487402 | 5.81E-08 | 8.09E-06 | 8.146646602 |
| PPP1R14D | 54866 | 0.356230543 | -0.012181866 | 6.172825581 | 5.83E-08 | 8.09E-06 | 8.144180033 |
| EID1 | 23741 | -0.684396044 | -0.130482116 | -6.168924398 | 5.92E-08 | 8.13E-06 | 8.129641648 |
| LOC401895 | 401895 | 0.594294741 | -0.147499554 | 6.16876408 | 5.92E-08 | 8.13E-06 | 8.129044236 |
| ABCC1 | 4363 | 0.627587921 | -0.145417915 | 6.165331582 | 6.00E-08 | 8.19E-06 | 8.116254118 |
| LOC441743 | 441743 | -0.847452689 | -0.210413322 | -6.164564001 | 6.02E-08 | 8.19E-06 | 8.113394173 |
| LOC646632 | 646632 | 0.552899164 | -0.044531051 | 6.164297131 | 6.03E-08 | 8.19E-06 | 8.112399853 |
| PACSIN1 | 29993 | 0.801627877 | -0.0880441 | 6.16224005 | 6.07E-08 | 8.23E-06 | 8.104735764 |
| CHN2 | 1124 | 0.44308296 | -0.126816942 | 6.159933646 | 6.13E-08 | 8.28E-06 | 8.096143405 |
| MDN1 | 23195 | 0.528001479 | -0.097513065 | 6.15796773 | 6.18E-08 | 8.29E-06 | 8.088820035 |
| C1orf127 | 148345 | 0.545073074 | -0.073752495 | 6.157757427 | 6.18E-08 | 8.29E-06 | 8.088036649 |
| LOC729444 | 729444 | 0.688115842 | -0.031263906 | 6.15427798 | 6.27E-08 | 8.37E-06 | 8.075076411 |
| FXR2 | 9513 | -0.661121105 | -0.030129917 | -6.152746146 | 6.30E-08 | 8.40E-06 | 8.069371124 |
| PSG3 | 5671 | 0.538131336 | -0.114726346 | 6.149570031 | 6.38E-08 | 8.47E-06 | 8.057542691 |
| C8orf17 | 56988 | 0.711223612 | -0.082043067 | 6.140731142 | 6.61E-08 | 8.74E-06 | 8.024631795 |
| OPRL1 | 4987 | 0.421032552 | -0.022580885 | 6.124706783 | 7.03E-08 | 9.21E-06 | 7.964991931 |
| LOC728689 | 728689 | 0.561065416 | -0.014918473 | 6.123746551 | 7.06E-08 | 9.21E-06 | 7.961419169 |
| GRIK2 | 2898 | 0.469330839 | -0.112143762 | 6.123444355 | 7.07E-08 | 9.21E-06 | 7.960294804 |
| IQCA1 | 79781 | 0.476166875 | -0.053264681 | 6.122942525 | 7.08E-08 | 9.21E-06 | 7.9584277 |
| LOC339674 | 339674 | 0.793093229 | -0.126400744 | 6.119312029 | 7.18E-08 | 9.31E-06 | 7.944921072 |
| C9orf68 | 55064 | 0.592599 | -0.077231736 | 6.114306313 | 7.32E-08 | 9.47E-06 | 7.926301004 |
| LOC391427 | 391427 | 0.651183057 | -0.042283978 | 6.112150437 | 7.38E-08 | 9.50E-06 | 7.918282673 |
| LOC644670 | 644670 | 0.541178434 | -0.101693654 | 6.111630246 | 7.40E-08 | 9.50E-06 | 7.916348021 |
| LRRC47 | 57470 | -0.882439423 | -0.146726384 | -6.109951335 | 7.45E-08 | 9.53E-06 | 7.910104197 |
| H2AFJ | 55766 | -0.657465996 | -0.118061133 | -4.684722758 | 1.60E-05 | 0.000355269 | 2.805343511 |
| LOC643801 | 643801 | 0.596240727 | -0.042148788 | 6.101932529 | 7.68E-08 | 9.77E-06 | 7.880287599 |
| LOC285941 | 285941 | 0.624035214 | -0.162833293 | 6.101021552 | 7.71E-08 | 9.78E-06 | 7.87690082 |
| LOC641518 | 641518 | 0.5695964 | -0.026341935 | 6.099452836 | 7.76E-08 | 9.80E-06 | 7.871068996 |
| DUSP10 | 11221 | 0.638302583 | -9.10E-05 | 6.09572398 | 7.87E-08 | 9.91E-06 | 7.857207991 |
| CECR7 | 27438 | -0.868072312 | -0.096686228 | -6.095160208 | 7.89E-08 | 9.91E-06 | 7.855112482 |
| SGTB | 54557 | 0.586358296 | -0.155884724 | 6.090450714 | 8.04E-08 | 1.01E-05 | 7.837609224 |
| DNMT3B | 1789 | 0.450527368 | -0.087415097 | 6.087377472 | 8.13E-08 | 1.01E-05 | 7.826188841 |
| LOC653180 | 653180 | 0.667252235 | -0.009984704 | 6.086998704 | 8.14E-08 | 1.01E-05 | 7.8247814 |
| LOC653711 | 653711 | 0.564966415 | -0.05066973 | 6.083163135 | 8.27E-08 | 1.02E-05 | 7.810530135 |
| WIPI2 | 26100 | -0.801860673 | -0.138627616 | -6.080317258 | 8.36E-08 | 1.03E-05 | 7.799957399 |
| CAB39L | 81617 | 0.485353484 | 0.042187063 | 6.078126966 | 8.43E-08 | 1.03E-05 | 7.791820978 |
| C2CD2 | 25966 | -1.121712402 | -0.176400759 | -6.077779742 | 8.44E-08 | 1.03E-05 | 7.790531182 |
| ADNP | 23394 | 0.5311648 | -0.065000272 | 6.077620185 | 8.45E-08 | 1.03E-05 | 7.789938499 |
| CCDC132 | 55610 | 0.43214842 | -0.077602867 | 6.070139333 | 8.70E-08 | 1.06E-05 | 7.762154299 |
| NAV2 | 89797 | -1.343829595 | -0.132009184 | -6.066335938 | 8.83E-08 | 1.07E-05 | 7.748031249 |
| LOC649248 | 649248 | 0.574545572 | -0.182113932 | 6.055428222 | 9.21E-08 | 1.11E-05 | 7.707538889 |
| TMEM169 | 92691 | 0.432658968 | -0.074264048 | 6.054290605 | 9.25E-08 | 1.11E-05 | 7.703316693 |
| MSRB3 | 253827 | -0.629149526 | -0.144289638 | -6.052708752 | 9.31E-08 | 1.11E-05 | 7.697446037 |
| MUC16 | 94025 | 0.509435332 | -0.113227958 | 6.050360545 | 9.39E-08 | 1.12E-05 | 7.688731887 |
| TMEM181 | 57583 | -1.273263045 | -0.133204178 | -6.050298007 | 9.39E-08 | 1.12E-05 | 7.688499823 |
| MYO1C | 4641 | -1.00685214 | -0.174845998 | -6.045244544 | 9.58E-08 | 1.13E-05 | 7.669749159 |
| DCI | 1632 | -0.773038949 | -0.044821805 | -6.045161248 | 9.58E-08 | 1.13E-05 | 7.66944012 |
| CACNB2 | 783 | 0.514714481 | -0.10055195 | 6.042097194 | 9.70E-08 | 1.14E-05 | 7.658072839 |
| UTRN | 7402 | 0.557034059 | -0.022119258 | 6.041881901 | 9.71E-08 | 1.14E-05 | 7.657274178 |
| RAB11FIP3 | 9727 | -1.066608231 | -0.095922651 | -6.03674055 | 9.90E-08 | 1.16E-05 | 7.638203469 |
| DNAJB12 | 54788 | 0.529449607 | -0.034797936 | 6.033673092 | 1.00E-07 | 1.16E-05 | 7.62682717 |
| RLN2 | 6019 | 0.495940121 | -0.058441061 | 6.032544529 | 1.01E-07 | 1.16E-05 | 7.622641996 |
| KCNE3 | 10008 | 0.49930414 | -0.076824802 | 6.028870729 | 1.02E-07 | 1.18E-05 | 7.609019279 |
| LOC641989 | 641989 | 0.664113007 | 0.024688557 | 6.028046184 | 1.02E-07 | 1.18E-05 | 7.605962069 |
| CYP4Z1 | 199974 | 0.46698068 | -0.083557106 | 6.025315888 | 1.04E-07 | 1.18E-05 | 7.595839483 |
| PMM1 | 5372 | -0.838368746 | -0.053499132 | -6.022096926 | 1.05E-07 | 1.19E-05 | 7.583906521 |
| NAT6 | 24142 | -0.57429683 | -0.064558117 | -6.021740214 | 1.05E-07 | 1.19E-05 | 7.58258425 |
| C20orf108 | 116151 | -1.031970778 | -0.139521004 | -6.020492431 | 1.05E-07 | 1.19E-05 | 7.577959073 |
| ARMCX4 | 158947 | 0.543219631 | -0.062536059 | 6.018224877 | 1.06E-07 | 1.20E-05 | 7.569554459 |
| FBXO44 | 93611 | 0.654608277 | 0.033076067 | 6.016861333 | 1.07E-07 | 1.21E-05 | 7.56450088 |
| FBXO32 | 114907 | -1.651361977 | -0.064239118 | -6.016117753 | 1.07E-07 | 1.21E-05 | 7.561745131 |
| STARD10 | 10809 | -0.611857151 | -0.107996617 | -6.014638423 | 1.08E-07 | 1.21E-05 | 7.556262879 |
| MANBAL | 63905 | -1.020493665 | -0.199252322 | -6.011469263 | 1.09E-07 | 1.21E-05 | 7.544519343 |
| UBL5 | 59286 | -0.465959875 | -0.150032222 | -6.0110955 | 1.09E-07 | 1.21E-05 | 7.543134434 |
| SNORA64 | 26784 | 0.65509279 | -0.008888888 | 6.00644377 | 1.11E-07 | 1.23E-05 | 7.525899985 |
| LOC652231 | 652231 | 0.60106149 | -0.010729377 | 6.00010664 | 1.14E-07 | 1.26E-05 | 7.502426214 |
| OSBPL5 | 114879 | -0.848985428 | -0.072309895 | -4.930418071 | 6.54E-06 | 0.000197164 | 3.649585239 |
| LOC642787 | 642787 | 0.507486509 | 0.013065754 | 5.990379817 | 1.19E-07 | 1.30E-05 | 7.466407759 |
| CLDN8 | 9073 | 0.546617998 | 0.018868105 | 5.989687758 | 1.19E-07 | 1.30E-05 | 7.463845586 |
| LOC440927 | 440927 | -1.358126041 | -0.158968278 | -5.988675101 | 1.19E-07 | 1.30E-05 | 7.460096606 |
| C14orf147 | 171546 | -1.040255827 | -0.226951065 | -5.987788173 | 1.20E-07 | 1.30E-05 | 7.456813212 |
| LOC641753 | 641753 | 0.465032264 | 0.000208363 | 5.981325338 | 1.23E-07 | 1.33E-05 | 7.43289138 |
| STARD7 | 56910 | 0.600042505 | -0.1079949 | 5.978192631 | 1.24E-07 | 1.34E-05 | 7.421298035 |
| KCNIP4 | 80333 | 0.574529434 | -0.103132514 | 5.976176704 | 1.25E-07 | 1.34E-05 | 7.413838373 |
| CNTN6 | 27255 | 0.578021255 | 0.058231074 | 5.973726677 | 1.26E-07 | 1.35E-05 | 7.404773186 |
| LOC652698 | 652698 | 0.431384419 | -0.087321053 | 5.970798899 | 1.28E-07 | 1.36E-05 | 7.393941467 |
| DKFZp451A211 | 400169 | -1.180579193 | -0.250473963 | -5.970542607 | 1.28E-07 | 1.36E-05 | 7.392993341 |
| SERGEF | 26297 | -0.748843875 | -0.063723569 | -5.967451194 | 1.30E-07 | 1.37E-05 | 7.381557723 |
| SIGMAR1 | 10280 | -0.496743474 | -0.108076734 | -5.198100368 | 2.43E-06 | 9.89E-05 | 4.589388111 |
| PHF2 | 5253 | -0.737506727 | -0.113290479 | -5.962717404 | 1.32E-07 | 1.39E-05 | 7.364049453 |
| RUFY3 | 22902 | 0.625470903 | -0.141023138 | 5.959700184 | 1.34E-07 | 1.40E-05 | 7.352891783 |
| BTC | 685 | -0.706587905 | -0.310666074 | -5.955005706 | 1.36E-07 | 1.42E-05 | 7.335534324 |
| ZNF532 | 55205 | -1.196671079 | -0.244061304 | -5.954538498 | 1.36E-07 | 1.42E-05 | 7.333807043 |
| TRIM36 | 55521 | 0.467946408 | -0.076522802 | 5.954531619 | 1.36E-07 | 1.42E-05 | 7.333781611 |
| LOC641978 | 641978 | 0.477952424 | -0.029961014 | 5.952313025 | 1.37E-07 | 1.43E-05 | 7.32557984 |
| BBX | 56987 | -1.221226391 | -0.181408076 | -5.950806003 | 1.38E-07 | 1.43E-05 | 7.320009058 |
| LOC654053 | 654053 | 0.634927562 | -0.117264234 | 5.94596855 | 1.41E-07 | 1.45E-05 | 7.302129472 |
| CSNK2A2 | 1459 | -0.958179853 | -0.167442679 | -5.945009857 | 1.41E-07 | 1.46E-05 | 7.298586491 |
| ALB | 213 | 0.647835985 | -0.061858282 | 5.943097662 | 1.42E-07 | 1.46E-05 | 7.291520128 |
| PRR3 | 80742 | -0.527617334 | -0.121603097 | -5.938707042 | 1.45E-07 | 1.48E-05 | 7.275297039 |
| LOC642047 | 642047 | 0.532336067 | -0.043825455 | 5.938519036 | 1.45E-07 | 1.48E-05 | 7.274602433 |
| CTSE | 1510 | 0.507266715 | -0.081406082 | 5.937347583 | 1.46E-07 | 1.48E-05 | 7.27027451 |
| ACSM3 | 6296 | 0.345858621 | -0.057638882 | 5.936484076 | 1.46E-07 | 1.48E-05 | 7.267084425 |
| FAM108B1 | 51104 | 0.576752613 | 0.045505891 | 5.934464732 | 1.47E-07 | 1.49E-05 | 7.25962473 |
| TXNL2 | 10539 | 0.429115118 | -0.075064129 | 5.925628676 | 1.52E-07 | 1.54E-05 | 7.226990623 |
| LOC653468 | 653468 | 0.557477102 | -0.100108783 | 5.924268933 | 1.53E-07 | 1.54E-05 | 7.221969759 |
| CETN3 | 1070 | -1.043891493 | -0.244022274 | -5.923803832 | 1.53E-07 | 1.54E-05 | 7.220252436 |
| MTE | 644314 | -1.343096632 | -0.03130789 | -5.922832298 | 1.54E-07 | 1.54E-05 | 7.216665281 |
| LOC402670 | 402670 | 0.714363379 | -0.142033796 | 5.921521314 | 1.55E-07 | 1.55E-05 | 7.211825021 |
| PRDM1 | 639 | 0.71163704 | -0.033222114 | 5.920250522 | 1.56E-07 | 1.55E-05 | 7.207133408 |
| RUTBC2 | 129049 | 0.485695631 | -0.137452135 | 5.915382988 | 1.59E-07 | 1.57E-05 | 7.189165346 |
| CCDC71 | 64925 | -0.750872515 | -0.264173142 | -5.911873839 | 1.61E-07 | 1.59E-05 | 7.176213916 |
| LOC642622 | 642622 | 0.626813951 | -0.171133966 | 5.911309959 | 1.61E-07 | 1.59E-05 | 7.174132946 |
| PAFAH2 | 5051 | -0.750340341 | -0.192120648 | -5.910084406 | 1.62E-07 | 1.59E-05 | 7.169610278 |
| FAM50B | 26240 | -0.716247505 | -0.099384976 | -5.909034995 | 1.62E-07 | 1.59E-05 | 7.165737817 |
| XPR1 | 9213 | -0.616515999 | -0.085777286 | -4.636240259 | 1.90E-05 | 0.000402687 | 2.64103922 |
| GEN1 | 348654 | 0.601642763 | -0.054009839 | 5.905796517 | 1.65E-07 | 1.61E-05 | 7.153788493 |
| LOC643363 | 643363 | 0.594219115 | -0.009571678 | 5.903163469 | 1.66E-07 | 1.61E-05 | 7.144074285 |
| TAF5L | 27097 | -0.59123703 | -0.122467902 | -5.900503682 | 1.68E-07 | 1.63E-05 | 7.13426253 |
| WWOX | 51741 | -0.44775748 | -0.211280267 | -5.895473453 | 1.71E-07 | 1.64E-05 | 7.115709422 |
| HPS6 | 79803 | -1.037420625 | -0.247842955 | -5.895044281 | 1.72E-07 | 1.64E-05 | 7.114126681 |
| AMACR | 23600 | 0.499156877 | -0.042615146 | 5.894970428 | 1.72E-07 | 1.64E-05 | 7.113854323 |
| FCN1 | 2219 | 1.718216833 | 0.093639847 | 5.894957203 | 1.72E-07 | 1.64E-05 | 7.113805552 |
| PPAPDC3 | 84814 | -0.630921665 | -0.080676636 | -5.889380321 | 1.75E-07 | 1.67E-05 | 7.093241314 |
| LOC644982 | 644982 | 0.595001665 | -0.076666312 | 5.888651962 | 1.76E-07 | 1.67E-05 | 7.09055592 |
| LOC205251 | 205251 | -0.699348629 | -0.076168024 | -5.886020182 | 1.78E-07 | 1.69E-05 | 7.080853485 |
| EXOSC4 | 54512 | -0.628711903 | -0.131784938 | -5.885628581 | 1.78E-07 | 1.69E-05 | 7.079409883 |
| DBT | 1629 | -1.20338177 | -0.297758375 | -5.880843025 | 1.81E-07 | 1.71E-05 | 7.061770351 |
| CD200R1 | 131450 | 0.45110424 | -0.037147259 | 5.874842817 | 1.85E-07 | 1.74E-05 | 7.039658761 |
| RAX | 30062 | 0.683585301 | -0.091749517 | 5.872103814 | 1.87E-07 | 1.76E-05 | 7.029567073 |
| TBC1D26 | 353149 | 0.670197761 | -0.080189697 | 5.871096747 | 1.88E-07 | 1.76E-05 | 7.025856894 |
| TIMM8A | 1678 | -0.407464971 | -0.114708355 | -5.8680539 | 1.90E-07 | 1.77E-05 | 7.014647611 |
| DEF8 | 54849 | -0.71617552 | -0.02713847 | -5.863650287 | 1.94E-07 | 1.80E-05 | 6.998428149 |
| LOC650520 | 650520 | 0.579156518 | -0.021997393 | 5.859621098 | 1.97E-07 | 1.82E-05 | 6.98359051 |
| ASGR2 | 433 | 0.489363732 | -0.095436915 | 5.854067116 | 2.01E-07 | 1.85E-05 | 6.963142066 |
| LOC646817 | 646817 | 0.643180756 | -0.111082128 | 5.850464052 | 2.04E-07 | 1.87E-05 | 6.949879116 |
| FOXJ2 | 55810 | -0.955383089 | -0.182425769 | -5.848236345 | 2.05E-07 | 1.88E-05 | 6.94167994 |
| STOML1 | 9399 | -0.545409928 | -0.097511933 | -5.848001996 | 2.06E-07 | 1.88E-05 | 6.940817452 |
| APP | 351 | -1.581773601 | -0.130978268 | -5.847613979 | 2.06E-07 | 1.88E-05 | 6.939389439 |
| LOC441124 | 441124 | 0.538624827 | 0.00754685 | 5.843778345 | 2.09E-07 | 1.90E-05 | 6.925274499 |
| GYPE | 2996 | 0.419688816 | -0.049068515 | 5.842911035 | 2.10E-07 | 1.90E-05 | 6.922083178 |
| FDPSL2A | 619190 | 0.616180863 | -0.051976708 | 5.842434722 | 2.10E-07 | 1.90E-05 | 6.920330605 |
| PTPRU | 10076 | -0.58661566 | -0.218306445 | -5.838272084 | 2.14E-07 | 1.93E-05 | 6.90501595 |
| UQCR | 10975 | -0.446561484 | -0.18686665 | -5.838028418 | 2.14E-07 | 1.93E-05 | 6.904119572 |
| FLJ34503 | 285759 | 0.509602417 | -0.002243647 | 5.831023331 | 2.20E-07 | 1.97E-05 | 6.87835405 |
| C6orf25 | 80739 | 0.425474481 | -0.025637422 | 5.826942631 | 2.23E-07 | 2.00E-05 | 6.863348498 |
| UNC13A | 23025 | 0.535013752 | -0.14347257 | 5.825006542 | 2.25E-07 | 2.01E-05 | 6.856230073 |
| TXNDC15 | 79770 | -0.769380152 | -0.0531251 | -5.818625192 | 2.30E-07 | 2.05E-05 | 6.832772159 |
| ZNF526 | 116115 | -0.65544014 | -0.09655062 | -5.817632983 | 2.31E-07 | 2.05E-05 | 6.829125395 |
| LOC400221 | 400221 | 0.454498586 | 0.03114933 | 5.815644393 | 2.33E-07 | 2.06E-05 | 6.821817032 |
| GMEB2 | 26205 | -0.782601676 | -0.10866167 | -5.813170597 | 2.35E-07 | 2.07E-05 | 6.812726389 |
| DALRD3 | 55152 | 0.54429174 | -0.035410105 | 5.809906737 | 2.38E-07 | 2.10E-05 | 6.800734005 |
| LDB3 | 11155 | -0.761707693 | -0.188352719 | -5.806588955 | 2.41E-07 | 2.12E-05 | 6.788545325 |
| WDR40B | 139170 | 0.597074637 | -0.101712886 | 5.805855402 | 2.42E-07 | 2.12E-05 | 6.785850694 |
| GEFT | 115557 | -0.592386286 | -0.15077596 | -5.805186649 | 2.43E-07 | 2.12E-05 | 6.783394173 |
| RABL2A | 11159 | 0.541406445 | -0.048807873 | 5.804512132 | 2.43E-07 | 2.12E-05 | 6.780916555 |
| C12orf43 | 64897 | -0.470477687 | -0.095185028 | -4.604363752 | 2.13E-05 | 0.000439002 | 2.533445221 |
| LOC644652 | 644652 | 0.406496157 | -0.102885156 | 5.804151388 | 2.44E-07 | 2.12E-05 | 6.779591514 |
| LOC392145 | 392145 | 0.499185719 | -0.100213328 | 5.80268033 | 2.45E-07 | 2.12E-05 | 6.774188422 |
| ATP2C1 | 27032 | -0.735764068 | -0.077082087 | -5.802090099 | 2.46E-07 | 2.12E-05 | 6.772020648 |
| TNPO1 | 3842 | -1.274207762 | -0.326570121 | -5.80038065 | 2.47E-07 | 2.13E-05 | 6.765742593 |
| CPSF3L | 54973 | -0.493945489 | -0.101521338 | -5.7945566 | 2.53E-07 | 2.17E-05 | 6.74435713 |
| LOC441019 | 441019 | -0.603090666 | -0.191823785 | -5.793965112 | 2.53E-07 | 2.17E-05 | 6.742185555 |
| GRIK1 | 2897 | 0.533114158 | -0.04008507 | 5.792247723 | 2.55E-07 | 2.18E-05 | 6.735880705 |
| DDX19A | 55308 | -0.681113823 | -0.131631552 | -5.790604912 | 2.57E-07 | 2.19E-05 | 6.729850115 |
| ARPC2 | 10109 | 0.527438367 | -0.086515667 | 5.78402837 | 2.63E-07 | 2.24E-05 | 6.705712918 |
| LRRC37B | 114659 | 0.52377294 | -0.097496325 | 5.783531287 | 2.64E-07 | 2.24E-05 | 6.703888825 |
| LOC645869 | 645869 | 0.576711957 | -0.057283545 | 5.781798957 | 2.65E-07 | 2.25E-05 | 6.697532211 |
| PRKDC | 5591 | 0.494983561 | -0.094980405 | 5.780497624 | 2.67E-07 | 2.25E-05 | 6.692757438 |
| RN7SL1 | 6029 | 1.274423023 | -0.03424356 | 5.773517761 | 2.74E-07 | 2.31E-05 | 6.667152307 |
| LOC651483 | 651483 | 0.608403623 | 0.026955546 | 5.772017714 | 2.76E-07 | 2.32E-05 | 6.661650586 |
| ACADL | 33 | -0.92811798 | -0.111964952 | -5.770959936 | 2.77E-07 | 2.32E-05 | 6.657771211 |
| LOC440248 | 440248 | 0.523141325 | -0.070666837 | 5.770408944 | 2.77E-07 | 2.32E-05 | 6.655750536 |
| FVT1 | 2531 | -0.941057294 | -0.061065786 | -5.769464768 | 2.78E-07 | 2.33E-05 | 6.652288044 |
| PCYOX1 | 51449 | -1.357184351 | -0.182449106 | -5.768300223 | 2.80E-07 | 2.33E-05 | 6.648017628 |
| ZC3H11A | 9877 | 0.606392079 | -0.10062237 | 5.766490359 | 2.82E-07 | 2.34E-05 | 6.641381273 |
| DNAJB6 | 10049 | -1.026118847 | -0.214869785 | -5.759802805 | 2.89E-07 | 2.39E-05 | 6.616864487 |
| LYRM1 | 57149 | -1.027237736 | -0.201764154 | -5.758698804 | 2.90E-07 | 2.40E-05 | 6.612817932 |
| LOC651125 | 651125 | 0.597173902 | -0.061278942 | 5.756668431 | 2.92E-07 | 2.41E-05 | 6.605376452 |
| ZNF718 | 255403 | 0.513544619 | 0.008611874 | 5.756424225 | 2.93E-07 | 2.41E-05 | 6.604481467 |
| LOC400145 | 400145 | 0.589069412 | -0.174286242 | 5.752907569 | 2.97E-07 | 2.44E-05 | 6.591594494 |
| LOC647417 | 647417 | 0.771417298 | -0.166425028 | 5.751671797 | 2.98E-07 | 2.44E-05 | 6.587066457 |
| LOC127602 | 127602 | 0.652491209 | -0.215681335 | 5.747412886 | 3.03E-07 | 2.46E-05 | 6.571463275 |
| LOC643304 | 643304 | 0.554020977 | -0.083602168 | 5.746832177 | 3.04E-07 | 2.46E-05 | 6.569336004 |
| HLX | 3142 | 0.475657921 | 0.012061626 | 5.74680132 | 3.04E-07 | 2.46E-05 | 6.569222971 |
| TMC3 | 342125 | 0.440367377 | -0.111453067 | 5.746556606 | 3.04E-07 | 2.46E-05 | 6.568326545 |
| LOC643624 | 643624 | 0.940721679 | 0.026381741 | 5.74615607 | 3.04E-07 | 2.46E-05 | 6.566859344 |
| LOC642362 | 642362 | 0.533769853 | -0.088741297 | 5.742140987 | 3.09E-07 | 2.49E-05 | 6.552153272 |
| LOC645968 | 645968 | -1.758650168 | -0.204102731 | -5.741862061 | 3.10E-07 | 2.49E-05 | 6.551131753 |
| GAGE7 | 2579 | 0.460469069 | -0.00061454 | 5.734442697 | 3.18E-07 | 2.56E-05 | 6.523964664 |
| YIPF6 | 286451 | -1.220940203 | -0.226094651 | -5.73409577 | 3.19E-07 | 2.56E-05 | 6.52269458 |
| MGC39900 | 286527 | -0.608480736 | -0.052854164 | -4.481745982 | 3.29E-05 | 0.000585806 | 2.122881603 |
| SLC26A1 | 10861 | 0.452622756 | -0.128589767 | 5.731500114 | 3.22E-07 | 2.57E-05 | 6.513192668 |
| GALNTL2 | 117248 | -1.176626065 | 0.015785467 | -5.73067724 | 3.23E-07 | 2.57E-05 | 6.510180624 |
| MTHFD2 | 10797 | -1.165183727 | -0.199891555 | -5.640501562 | 4.56E-07 | 3.32E-05 | 6.180848386 |
| MUC1 | 4582 | -0.289318728 | -0.073548484 | -4.337048118 | 5.46E-05 | 0.000815014 | 1.64547595 |
| LOC643558 | 643558 | 0.651878975 | -0.167092656 | 5.723942455 | 3.32E-07 | 2.62E-05 | 6.485533199 |
| LOC643154 | 643154 | 0.598671984 | -0.072082254 | 5.717184201 | 3.40E-07 | 2.68E-05 | 6.460808042 |
| MANSC1 | 54682 | -0.659389184 | -0.158220662 | -5.714119199 | 3.44E-07 | 2.69E-05 | 6.449597394 |
| SLC9A3 | 6550 | 0.54028808 | -0.104853624 | 5.714037519 | 3.44E-07 | 2.69E-05 | 6.449298662 |
| LOC142937 | 142937 | 0.663763728 | -0.098723217 | 5.71329636 | 3.45E-07 | 2.69E-05 | 6.44658804 |
| B3GNTL1 | 146712 | 0.609847257 | -0.153322065 | 5.711452317 | 3.48E-07 | 2.70E-05 | 6.4398443 |
| NELF | 26012 | -0.918292792 | -0.093726685 | -5.704001838 | 3.58E-07 | 2.77E-05 | 6.412603855 |
| NPTX2 | 4885 | -1.372132355 | 0.051396889 | -5.703556824 | 3.59E-07 | 2.77E-05 | 6.410977113 |
| DKFZp434J1015 | 54753 | 0.597301955 | -0.081045024 | 5.703285423 | 3.59E-07 | 2.77E-05 | 6.409985029 |
| VEZF1 | 7716 | -1.175688179 | -0.231589595 | -5.701596629 | 3.61E-07 | 2.78E-05 | 6.403812076 |
| ARVCF | 421 | -0.360643431 | -0.12890695 | -5.701041058 | 3.62E-07 | 2.78E-05 | 6.401781444 |
| LOC646210 | 646210 | 0.536867772 | -0.006641507 | 5.69947388 | 3.64E-07 | 2.79E-05 | 6.396053651 |
| LASS6 | 253782 | -1.066755227 | -0.082824602 | -5.696779654 | 3.68E-07 | 2.81E-05 | 6.386207719 |
| GOLGA8B | 440270 | 0.402080098 | -0.041301025 | 5.695962783 | 3.69E-07 | 2.81E-05 | 6.38322276 |
| BIN2 | 51411 | 0.61678302 | 0.019473144 | 5.688774342 | 3.79E-07 | 2.89E-05 | 6.356960467 |
| C17orf95 | 124512 | -0.51690942 | -0.108761582 | -5.685767871 | 3.84E-07 | 2.91E-05 | 6.345979419 |
| RBPMS2 | 348093 | -1.525108254 | -0.006527559 | -5.685555733 | 3.84E-07 | 2.91E-05 | 6.345204651 |
| SLC4A4 | 8671 | 0.482457765 | -0.051480005 | 5.68305102 | 3.88E-07 | 2.94E-05 | 6.336057628 |
| MRPL11 | 65003 | 0.424301463 | -0.047717493 | 5.681918392 | 3.90E-07 | 2.94E-05 | 6.331921733 |
| HIST1H2AG | 8969 | 0.473297815 | -0.10578269 | 5.677959728 | 3.96E-07 | 2.98E-05 | 6.317468171 |
| ABCA10 | 10349 | 0.408210219 | -0.019250648 | 5.677874097 | 3.96E-07 | 2.98E-05 | 6.317155551 |
| LOC650733 | 650733 | 0.536354113 | -0.103839968 | 5.673209582 | 4.03E-07 | 3.02E-05 | 6.300128634 |
| TRK1 | 7206 | -1.209003724 | -0.060020508 | -5.672381329 | 4.04E-07 | 3.03E-05 | 6.297105675 |
| CCT5 | 22948 | -0.530131327 | -0.075457519 | -5.662837045 | 4.19E-07 | 3.14E-05 | 6.26228016 |
| LOC650020 | 650020 | 0.533231089 | 0.025971066 | 5.661934862 | 4.21E-07 | 3.14E-05 | 6.25898912 |
| GUCY2F | 2986 | 0.58105128 | -0.081285289 | 5.65921261 | 4.25E-07 | 3.16E-05 | 6.249059646 |
| C1orf114 | 57821 | 0.497633642 | -0.074241389 | 5.656048719 | 4.30E-07 | 3.18E-05 | 6.237521027 |
| CCDC27 | 148870 | 0.430249038 | -0.089952339 | 5.653574894 | 4.34E-07 | 3.21E-05 | 6.228500368 |
| LOC645089 | 645089 | 0.554244241 | -0.04452401 | 5.652299637 | 4.36E-07 | 3.22E-05 | 6.223850668 |
| SLC9A4 | 389015 | 0.908104959 | 0.093147914 | 5.651377721 | 4.38E-07 | 3.22E-05 | 6.220489473 |
| ITGA10 | 8515 | -1.184775135 | -0.145496337 | -5.650773267 | 4.39E-07 | 3.22E-05 | 6.218285789 |
| ARRDC2 | 27106 | 0.390724599 | -0.077093749 | 5.648616416 | 4.43E-07 | 3.24E-05 | 6.210423034 |
| LOC644330 | 644330 | 1.169944773 | 0.222276356 | 5.641084247 | 4.55E-07 | 3.32E-05 | 6.182971571 |
| TIMP4 | 7079 | -1.64368688 | 0.06613203 | -5.630533199 | 4.74E-07 | 3.44E-05 | 6.144535719 |
| CKS1B | 1163 | -1.007479128 | -0.117619878 | -5.630503672 | 4.74E-07 | 3.44E-05 | 6.144428188 |
| LOC343296 | 343296 | 0.433346156 | -0.089119508 | 5.629114573 | 4.77E-07 | 3.45E-05 | 6.139369502 |
| GABRP | 2568 | 0.54592455 | -0.051961213 | 5.628549218 | 4.78E-07 | 3.45E-05 | 6.137310754 |
| MRRF | 92399 | 0.512373244 | 0.034648236 | 5.627266186 | 4.80E-07 | 3.46E-05 | 6.132638798 |
| GPATCH4 | 54865 | -0.730538732 | -0.124426897 | -5.62698699 | 4.81E-07 | 3.46E-05 | 6.131622193 |
| LOC645574 | 645574 | 0.355842542 | -0.031114163 | 5.62508751 | 4.84E-07 | 3.47E-05 | 6.124706226 |
| LOC442117 | 442117 | 0.694851496 | -0.166667272 | 5.624586024 | 4.85E-07 | 3.47E-05 | 6.12288044 |
| PHYH | 5264 | -1.12056216 | -0.198278028 | -5.624100052 | 4.86E-07 | 3.47E-05 | 6.121111184 |
| GIYD2 | 79008 | 0.645708764 | 0.03677479 | 5.623042015 | 4.88E-07 | 3.48E-05 | 6.11725939 |
| R3HCC1 | 203069 | -0.673273884 | -0.151514874 | -5.622343439 | 4.89E-07 | 3.48E-05 | 6.114716338 |
| PCNT | 5116 | -0.737236188 | -0.009407519 | -5.621437108 | 4.91E-07 | 3.48E-05 | 6.111417126 |
| TMEM50B | 757 | -1.221813403 | -0.18524621 | -5.621131966 | 4.92E-07 | 3.48E-05 | 6.11030639 |
| WFDC12 | 128488 | 0.504517702 | -0.024401804 | 5.620862073 | 4.92E-07 | 3.48E-05 | 6.109323976 |
| FAM21C | 253725 | 0.55747309 | -0.045356878 | 5.617741207 | 4.98E-07 | 3.52E-05 | 6.097965004 |
| NDUFB6 | 4712 | -0.850852716 | -0.165512697 | -5.614906426 | 5.03E-07 | 3.55E-05 | 6.087648918 |
| LDHC | 3948 | 0.52214025 | -0.066827076 | 5.611798976 | 5.09E-07 | 3.59E-05 | 6.076342341 |
| IL17RD | 54756 | 0.56856167 | -0.124603986 | 5.610790633 | 5.11E-07 | 3.59E-05 | 6.072673849 |
| HYDIN | 54768 | 0.57650033 | -0.133801258 | 5.280316578 | 1.79E-06 | 8.04E-05 | 4.881792308 |
| EXOSC10 | 5394 | -0.897346908 | -0.179599452 | -4.663139992 | 1.73E-05 | 0.000374961 | 2.732103474 |
| LOC652491 | 652491 | 0.506859481 | -0.134779143 | 5.608226418 | 5.16E-07 | 3.60E-05 | 6.063345764 |
| CROP | 51747 | 0.384086224 | -0.113111257 | 5.607856839 | 5.17E-07 | 3.60E-05 | 6.062001418 |
| ERH | 2079 | -0.977477907 | -0.065514699 | -5.607655372 | 5.18E-07 | 3.60E-05 | 6.061268588 |
| PTPN6 | 5777 | 0.675262167 | -0.145061629 | 5.606552625 | 5.20E-07 | 3.60E-05 | 6.05725754 |
| ETV4 | 2118 | 0.507182542 | -0.102783404 | 5.605810117 | 5.21E-07 | 3.60E-05 | 6.054556926 |
| NAB2 | 4665 | -0.535372989 | -0.109086196 | -5.604896042 | 5.23E-07 | 3.61E-05 | 6.051232447 |
| SERF1B | 728492 | -0.555520864 | -0.125753792 | -5.603178607 | 5.26E-07 | 3.62E-05 | 6.044986601 |
| LOC401357 | 401357 | 1.351882667 | 0.143099848 | 5.599951609 | 5.33E-07 | 3.66E-05 | 6.033252431 |
| FGF20 | 26281 | 0.466694184 | -0.105900911 | 5.599810105 | 5.33E-07 | 3.66E-05 | 6.032737932 |
| HORMAD1 | 84072 | 0.461740125 | -0.106888039 | 5.59858204 | 5.36E-07 | 3.67E-05 | 6.02827295 |
| JPH2 | 57158 | -0.608458041 | -0.233133002 | -5.59243769 | 5.48E-07 | 3.74E-05 | 6.005937833 |
| TTLL12 | 23170 | -0.604462705 | -0.094271478 | -5.590755395 | 5.52E-07 | 3.76E-05 | 5.999823869 |
| HNRNPCL1 | 343069 | 0.678142755 | -0.083419447 | 5.590490298 | 5.53E-07 | 3.76E-05 | 5.998860479 |
| ZSWIM1 | 90204 | -0.766048971 | -0.074898387 | -5.589546096 | 5.55E-07 | 3.76E-05 | 5.995429258 |
| ZFAT | 57623 | 0.532212126 | -0.01061103 | 5.588064437 | 5.58E-07 | 3.78E-05 | 5.990045278 |
| LOC645946 | 645946 | 0.681458988 | -0.066439789 | 5.587262414 | 5.59E-07 | 3.78E-05 | 5.987131106 |
| ZMYND11 | 10771 | -1.068095944 | -0.265905583 | -5.586607802 | 5.61E-07 | 3.79E-05 | 5.98475265 |
| PRRX2 | 51450 | -0.906484504 | -0.164911791 | -5.585897636 | 5.62E-07 | 3.79E-05 | 5.982172442 |
| RGPD5 | 84220 | 0.542133362 | -0.082991105 | 5.584188732 | 5.66E-07 | 3.81E-05 | 5.975963977 |
| CDAN1 | 146059 | 0.478715822 | -0.122634869 | 5.58242556 | 5.70E-07 | 3.83E-05 | 5.969558957 |
| LOC649926 | 649926 | 0.428682025 | -0.131623052 | 5.581886129 | 5.71E-07 | 3.83E-05 | 5.967599506 |
| LOC645123 | 645123 | 0.742531567 | -0.034493994 | 5.581317211 | 5.72E-07 | 3.83E-05 | 5.965533008 |
| UCHL5IP | 55559 | -0.925431825 | -0.188317941 | -5.581047248 | 5.73E-07 | 3.83E-05 | 5.964552436 |
| ALOX15 | 246 | 0.397921032 | -0.074290411 | 5.579116268 | 5.77E-07 | 3.84E-05 | 5.957539058 |
| PCDHGB6 | 56100 | 0.624995422 | 0.001286785 | 5.57549065 | 5.85E-07 | 3.89E-05 | 5.944372701 |
| SLC16A5 | 9121 | 0.463855795 | -0.018918147 | 5.57335428 | 5.90E-07 | 3.91E-05 | 5.936615738 |
| MUC12 | 10071 | 0.527482497 | -0.0503159 | 5.572935044 | 5.91E-07 | 3.91E-05 | 5.935093636 |
| LOC643331 | 643331 | 0.547099841 | -0.078858479 | 5.57192042 | 5.93E-07 | 3.91E-05 | 5.931410033 |
| C1orf87 | 127795 | 0.642007924 | -0.101932625 | 5.571883362 | 5.93E-07 | 3.91E-05 | 5.931275497 |
| SNORD73A | 8944 | 0.506335747 | -0.049109529 | 5.565153773 | 6.09E-07 | 4.00E-05 | 5.906848884 |
| PGRMC1 | 10857 | -1.237451982 | -0.257009758 | -5.563866846 | 6.12E-07 | 4.01E-05 | 5.90217871 |
| TBX21 | 30009 | -0.414589022 | -0.221492282 | -5.561611727 | 6.17E-07 | 4.04E-05 | 5.893995831 |
| LOC652317 | 652317 | 0.629521766 | -0.034347408 | 5.560710744 | 6.19E-07 | 4.05E-05 | 5.890726829 |
| LOC653333 | 653333 | 0.489764873 | -0.098954542 | 5.557305393 | 6.27E-07 | 4.08E-05 | 5.87837279 |
| LOC643475 | 643475 | 0.591258214 | -0.063740924 | 5.55709961 | 6.28E-07 | 4.08E-05 | 5.87762632 |
| UBE2CBP | 90025 | 0.365049618 | -0.043066368 | 5.556549196 | 6.29E-07 | 4.08E-05 | 5.875629752 |
| DCST1 | 149095 | 0.495159151 | -0.110580943 | 5.556098607 | 6.30E-07 | 4.08E-05 | 5.873995333 |
| T-SP1 | 203074 | 0.581483871 | -0.026934145 | 5.555585216 | 6.31E-07 | 4.08E-05 | 5.872133166 |
| PAGE3 | 139793 | 0.451940931 | -0.095758614 | 5.555050078 | 6.32E-07 | 4.08E-05 | 5.87019217 |
| UPF2 | 26019 | -0.931542822 | -0.133072925 | -5.554990118 | 6.33E-07 | 4.08E-05 | 5.869974694 |
| PDE3A | 5139 | -0.424788362 | -0.20852772 | -5.553090478 | 6.37E-07 | 4.10E-05 | 5.863085019 |
| LOC643920 | 643920 | 0.565637255 | -0.047382342 | 5.55123249 | 6.42E-07 | 4.12E-05 | 5.85634711 |
| DNAJC5G | 285126 | 0.47497329 | -0.160670152 | 5.551026169 | 6.42E-07 | 4.12E-05 | 5.855598941 |
| PPP1CB | 5500 | -1.196066688 | -0.267586999 | -5.549938522 | 6.45E-07 | 4.13E-05 | 5.851655005 |
| ACSM2B | 348158 | 0.768662192 | 0.057393451 | 5.54828843 | 6.49E-07 | 4.14E-05 | 5.845672037 |
| OR13C9 | 286362 | 0.578487907 | -0.093938215 | 5.548072176 | 6.50E-07 | 4.14E-05 | 5.844887977 |
| LOC730525 | 730525 | -1.634394106 | -0.19957947 | -5.546704348 | 6.53E-07 | 4.15E-05 | 5.839928928 |
| GRK5 | 2869 | -0.405634875 | -0.111547536 | -4.754415396 | 1.24E-05 | 0.000298676 | 3.042887953 |
| RECQL | 5965 | -0.864572069 | -0.144268919 | -5.544547241 | 6.58E-07 | 4.17E-05 | 5.83210913 |
| SPANXA1 | 30014 | 0.564611637 | -0.100191993 | 5.54299777 | 6.62E-07 | 4.18E-05 | 5.82649267 |
| NR4A1 | 3164 | 0.548428423 | -0.07010012 | 5.542269604 | 6.64E-07 | 4.18E-05 | 5.823853415 |
| STAC2 | 342667 | -0.580065779 | -0.170692182 | -5.541302866 | 6.66E-07 | 4.18E-05 | 5.820349611 |
| DDX51 | 317781 | -0.45076487 | -0.162695167 | -5.539439276 | 6.71E-07 | 4.21E-05 | 5.813595834 |
| LPAR1 | 1902 | -1.007051906 | -0.171493573 | -5.530564547 | 6.94E-07 | 4.33E-05 | 5.781442919 |
| RPS6 | 6194 | -0.94152883 | -0.209270195 | -5.52901318 | 6.98E-07 | 4.34E-05 | 5.775824008 |
| ZMAT5 | 55954 | 0.734140098 | -0.14393881 | 5.528939712 | 6.99E-07 | 4.34E-05 | 5.775557923 |
| F9 | 2158 | 0.594349115 | -0.10029787 | 5.527975321 | 7.01E-07 | 4.35E-05 | 5.772065249 |
| LOC729021 | 729021 | 1.474600077 | 0.526611535 | 5.52796102 | 7.01E-07 | 4.35E-05 | 5.77201346 |
| PDCD2 | 5134 | -0.592053924 | -0.083326715 | -5.526350532 | 7.05E-07 | 4.36E-05 | 5.766181284 |
| DSEL | 92126 | -0.900493522 | -0.143265736 | -5.525647297 | 7.07E-07 | 4.36E-05 | 5.763634778 |
| ICMT | 23463 | -0.834598787 | -0.10396064 | -5.525622025 | 7.07E-07 | 4.36E-05 | 5.763543265 |
| LOC645781 | 645781 | 0.423952548 | -0.078319388 | 5.525476068 | 7.08E-07 | 4.36E-05 | 5.763014749 |
| LOC652290 | 652290 | 0.466370782 | -0.119073995 | 5.52399943 | 7.12E-07 | 4.38E-05 | 5.757668028 |
| LOC730249 | 730249 | 0.519524557 | -0.114084011 | 5.522134391 | 7.17E-07 | 4.39E-05 | 5.750915596 |
| MGC16703 | 113691 | 1.216182459 | -0.005577861 | 5.521972397 | 7.17E-07 | 4.39E-05 | 5.750329126 |
| GMDS | 2762 | -1.597636737 | -0.391300197 | -5.521932964 | 7.17E-07 | 4.39E-05 | 5.750186367 |
| FAM19A4 | 151647 | 0.532971008 | -0.002684554 | 5.521773198 | 7.18E-07 | 4.39E-05 | 5.749607966 |
| ARPM1 | 84517 | -0.384483549 | -0.147080124 | -5.519908317 | 7.23E-07 | 4.41E-05 | 5.742856962 |
| LPIN1 | 23175 | -1.3407411 | -0.254282913 | -5.519830868 | 7.23E-07 | 4.41E-05 | 5.742576604 |
| LOC654203 | 654203 | 0.527438368 | -0.056418047 | 5.516882636 | 7.31E-07 | 4.45E-05 | 5.731905298 |
| TPRG1L | 127262 | -0.858145555 | -0.072359358 | -5.512749084 | 7.43E-07 | 4.51E-05 | 5.716946674 |
| KLRC3 | 3823 | 0.454737109 | -0.053104874 | 5.511836467 | 7.45E-07 | 4.51E-05 | 5.713644544 |
| MGC57359 | 441272 | 0.497508357 | -0.071874914 | 5.511608416 | 7.46E-07 | 4.51E-05 | 5.712819412 |
| C3orf39 | 84892 | -0.655116634 | -0.011802421 | -5.511337779 | 7.47E-07 | 4.51E-05 | 5.71184021 |
| TSPYL1 | 7259 | -0.968145843 | -0.067034319 | -5.508981368 | 7.54E-07 | 4.54E-05 | 5.703315029 |
| C2orf21 | 285175 | 0.636035674 | -0.15649556 | 5.508645281 | 7.55E-07 | 4.54E-05 | 5.702099204 |
| LOC729249 | 729249 | 0.566905934 | -0.117120506 | 5.508378061 | 7.55E-07 | 4.54E-05 | 5.701132526 |
| UFSP2 | 55325 | -0.889005116 | -0.178723162 | -5.505895968 | 7.62E-07 | 4.57E-05 | 5.692154203 |
| LIX1L | 128077 | -0.553373207 | -0.060977009 | -5.504306572 | 7.67E-07 | 4.58E-05 | 5.68640565 |
| LOC643594 | 643594 | 0.598498059 | -0.155401276 | 5.503632591 | 7.69E-07 | 4.59E-05 | 5.68396814 |
| MXI1 | 4601 | -0.605165782 | -0.183260436 | -5.500076164 | 7.80E-07 | 4.64E-05 | 5.671107601 |
| GPD1L | 23171 | -1.244739718 | -0.252869563 | -5.498555891 | 7.84E-07 | 4.66E-05 | 5.665610885 |
| FLJ40113 | 374650 | 0.645424681 | -0.067536204 | 5.497538995 | 7.87E-07 | 4.67E-05 | 5.661934454 |
| UBN1 | 29855 | 0.55288939 | -0.025474057 | 5.496629729 | 7.90E-07 | 4.68E-05 | 5.658647325 |
| CPNE2 | 221184 | 0.615170325 | -0.083986337 | 5.496310154 | 7.91E-07 | 4.68E-05 | 5.657492059 |
| RPL17 | 6139 | -0.648554867 | -0.211085856 | -4.327078611 | 5.65E-05 | 0.000834569 | 1.612878042 |
| IDO2 | 169355 | 0.506433106 | -0.03389001 | 5.494137662 | 7.97E-07 | 4.71E-05 | 5.649639029 |
| PRPS2 | 5634 | -0.773249171 | -0.113157796 | -5.493156011 | 8.00E-07 | 4.71E-05 | 5.646090927 |
| MSH3 | 4437 | 1.309955165 | 0.085386828 | 5.492936906 | 8.01E-07 | 4.71E-05 | 5.645299015 |
| ZBED5 | 58486 | -1.170044813 | -0.279914718 | -5.491715401 | 8.05E-07 | 4.73E-05 | 5.640884315 |
| LOC650146 | 650146 | 0.441050535 | -0.122071806 | 5.487457877 | 8.18E-07 | 4.79E-05 | 5.625499442 |
| LOC645241 | 645241 | 0.522393531 | -0.038455516 | 5.487389539 | 8.18E-07 | 4.79E-05 | 5.625252528 |
| AK3L1 | 205 | -0.777788297 | -0.12692224 | -4.728500258 | 1.36E-05 | 0.000318903 | 2.954372759 |
| LOC389365 | 389365 | 0.431208241 | 0.001438605 | 5.483340395 | 8.31E-07 | 4.85E-05 | 5.610624246 |
| HNRNPUL2 | 221092 | -0.6859514 | -0.093028955 | -5.482221177 | 8.34E-07 | 4.86E-05 | 5.606581475 |
| MYH6 | 4624 | 0.480714304 | -0.053103863 | 5.481922591 | 8.35E-07 | 4.86E-05 | 5.605502986 |
| PTGIR | 5739 | -0.601880612 | -0.173028066 | -5.481721892 | 8.36E-07 | 4.86E-05 | 5.604778071 |
| SLC22A7 | 10864 | 0.693170414 | -0.090736522 | 5.479726051 | 8.42E-07 | 4.89E-05 | 5.597569683 |
| NBPF9 | 440670 | 0.43651057 | 0.025908463 | 5.47949404 | 8.43E-07 | 4.89E-05 | 5.596731782 |
| HSBP1 | 3281 | -0.71806115 | -0.071027019 | -5.476381747 | 8.53E-07 | 4.93E-05 | 5.585492931 |
| LOC727909 | 727909 | 0.403252467 | 0.008262754 | 5.476286236 | 8.53E-07 | 4.93E-05 | 5.585148062 |
| C7orf29 | 113763 | 0.510873501 | -0.02571937 | 5.475901366 | 8.54E-07 | 4.93E-05 | 5.583758401 |
| FAM39DP | 374666 | 0.535855442 | -0.039064351 | 5.473949809 | 8.61E-07 | 4.95E-05 | 5.576712347 |
| LOC642755 | 642755 | -0.696176276 | -0.121895409 | -5.149883483 | 2.91E-06 | 0.000113012 | 4.418689428 |
| SRGAP3 | 9901 | 0.466109511 | -0.029381002 | 5.473294989 | 8.63E-07 | 4.95E-05 | 5.574348315 |
| PPOX | 5498 | -0.633283915 | -0.123644297 | -5.472705335 | 8.65E-07 | 4.96E-05 | 5.572219625 |
| NR1I3 | 9970 | 0.490549401 | -0.059746813 | 5.471519118 | 8.69E-07 | 4.96E-05 | 5.567937521 |
| AKAP8L | 26993 | -0.754120832 | 0.000104408 | -5.470949903 | 8.71E-07 | 4.96E-05 | 5.565882831 |
| MAGEB1 | 4112 | 0.475426237 | -0.110002123 | 5.469013204 | 8.77E-07 | 4.99E-05 | 5.558892458 |
| TRIM58 | 25893 | 0.500839432 | -0.080168015 | 5.467859704 | 8.81E-07 | 5.00E-05 | 5.554729361 |
| TAS2R41 | 259287 | 0.568738253 | -0.131448493 | 5.463721217 | 8.95E-07 | 5.07E-05 | 5.53979549 |
| PHPT1 | 29085 | -1.082589514 | -0.210166071 | -5.463077344 | 8.97E-07 | 5.07E-05 | 5.537472382 |
| NAP1L4 | 4676 | -0.827775288 | -0.156805006 | -5.461369321 | 9.03E-07 | 5.10E-05 | 5.531310224 |
| FLJ35816 | 401114 | 0.506027753 | 0.039228283 | 5.459922441 | 9.08E-07 | 5.12E-05 | 5.526090698 |
| LOC645153 | 645153 | 0.482500434 | -0.097846668 | 5.458357794 | 9.13E-07 | 5.14E-05 | 5.520446842 |
| ALDH8A1 | 64577 | 0.56745287 | -0.127589432 | 5.45479548 | 9.26E-07 | 5.21E-05 | 5.507599137 |
| CLCA4 | 22802 | 0.468746082 | -0.115994322 | 5.453615561 | 9.30E-07 | 5.22E-05 | 5.503344286 |
| LOC400456 | 400456 | 0.548511019 | -0.096666321 | 5.450024185 | 9.43E-07 | 5.29E-05 | 5.490395438 |
| AKIRIN1 | 79647 | -0.954369619 | -0.219580601 | -5.448783774 | 9.47E-07 | 5.30E-05 | 5.485923734 |
| ATL3 | 25923 | -0.833502145 | -0.159704937 | -5.447454711 | 9.52E-07 | 5.31E-05 | 5.481132804 |
| RPL36A | 6173 | 0.518802756 | 0.051052291 | 5.447179363 | 9.53E-07 | 5.31E-05 | 5.480140294 |
| POTEA | 340441 | 0.559650981 | -0.018438318 | 5.447063972 | 9.53E-07 | 5.31E-05 | 5.479724365 |
| LOC728492 | 728492 | -0.977359601 | -0.100995756 | -5.446620904 | 9.55E-07 | 5.31E-05 | 5.478127341 |
| OR8K5 | 219453 | 0.600151345 | -0.18372735 | 5.446418158 | 9.55E-07 | 5.31E-05 | 5.477396566 |
| ANAPC11 | 51529 | -0.702212606 | -0.193139874 | -5.112383866 | 3.34E-06 | 0.000123323 | 4.286347301 |
| SCN1A | 6323 | 0.600231964 | -0.048371302 | 5.444935011 | 9.61E-07 | 5.33E-05 | 5.472050985 |
| KLHDC5 | 57542 | -1.099521298 | -0.192859005 | -5.443697704 | 9.65E-07 | 5.33E-05 | 5.467591832 |
| SLTM | 79811 | 0.619375007 | -0.010294183 | 5.443600323 | 9.66E-07 | 5.33E-05 | 5.467240891 |
| NAG18 | 57051 | 1.425474065 | 0.064067313 | 5.443574897 | 9.66E-07 | 5.33E-05 | 5.467149265 |
| LOC649456 | 649456 | 0.432018816 | -0.023218919 | 5.442590574 | 9.69E-07 | 5.34E-05 | 5.463602105 |
| LOC647011 | 647011 | 0.442704386 | -0.064551609 | 5.44254676 | 9.70E-07 | 5.34E-05 | 5.463444219 |
| LOC648897 | 648897 | 0.393148042 | -0.022980121 | 5.441309527 | 9.74E-07 | 5.36E-05 | 5.458985971 |
| ALDH3A2 | 224 | -0.884824301 | -0.117428739 | -4.567299855 | 2.43E-05 | 0.000479409 | 2.408782797 |
| TUBGCP6 | 85378 | 0.617398686 | -0.067403976 | 5.439401863 | 9.81E-07 | 5.38E-05 | 5.452112541 |
| NHP2 | 55651 | -0.704253893 | -0.101165383 | -4.95676635 | 5.94E-06 | 0.000183654 | 3.741210045 |
| TLE1 | 7088 | -0.95055279 | -0.026206068 | -5.436890628 | 9.91E-07 | 5.41E-05 | 5.443065609 |
| KIAA0831 | 22863 | -0.989049372 | -0.125461565 | -5.436166284 | 9.93E-07 | 5.42E-05 | 5.440456356 |
| PQLC2 | 54896 | 0.721554876 | -0.052686449 | 5.43130098 | 1.01E-06 | 5.51E-05 | 5.422933371 |
| SORCS3 | 22986 | 0.539161405 | -0.121511418 | 5.430413895 | 1.02E-06 | 5.51E-05 | 5.419738983 |
| LOC283970 | 283970 | 0.481163972 | -0.007239033 | 5.42909614 | 1.02E-06 | 5.53E-05 | 5.414994069 |
| PCDH7 | 5099 | -0.329028067 | -0.139212331 | -4.458459447 | 3.57E-05 | 0.000619134 | 2.045521859 |
| PARVA | 55742 | -0.818570379 | -0.066893744 | -5.426761059 | 1.03E-06 | 5.57E-05 | 5.40658695 |
| ATPBD1B | 54707 | -0.901777219 | -0.121815388 | -5.426168321 | 1.03E-06 | 5.57E-05 | 5.404453071 |
| LOC642616 | 642616 | 0.610397337 | -0.174368882 | 5.424168387 | 1.04E-06 | 5.59E-05 | 5.397253807 |
| LOC197135 | 197135 | 0.756288275 | 0.027084625 | 5.424104767 | 1.04E-06 | 5.59E-05 | 5.397024807 |
| LAMA3 | 3909 | 0.466645194 | -0.0092862 | 5.423959088 | 1.04E-06 | 5.59E-05 | 5.396500433 |
| OLIG1 | 116448 | 0.474775232 | -0.036094397 | 5.421711305 | 1.05E-06 | 5.63E-05 | 5.388410128 |
| LOC653337 | 653337 | 0.474215668 | -0.053842414 | 5.419106711 | 1.06E-06 | 5.67E-05 | 5.379036955 |
| MGC39581 | 257062 | 0.538983319 | -0.0113451 | 5.417764326 | 1.06E-06 | 5.69E-05 | 5.374206688 |
| RDH11 | 51109 | -1.051325143 | -0.073706805 | -5.413779941 | 1.08E-06 | 5.76E-05 | 5.35987212 |
| ZC3H12A | 80149 | 1.33305897 | 0.323354135 | 5.413750245 | 1.08E-06 | 5.76E-05 | 5.359765297 |
| PHB | 5245 | -0.792032068 | -0.198478883 | -5.410930017 | 1.09E-06 | 5.81E-05 | 5.349621135 |
| PSCDBP | 9595 | 0.469357677 | -0.059212443 | 5.40650183 | 1.11E-06 | 5.89E-05 | 5.333696809 |
| SEZ6 | 124925 | 0.597708313 | -0.04219204 | 5.406354382 | 1.11E-06 | 5.89E-05 | 5.33316664 |
| RECK | 8434 | -1.180578818 | -0.2632437 | -5.406102814 | 1.11E-06 | 5.89E-05 | 5.332262108 |
| LOC651436 | 651436 | -0.886686798 | -0.189605814 | -5.405504277 | 1.12E-06 | 5.89E-05 | 5.330110077 |
| FAM44B | 91272 | -0.783067592 | -0.200216407 | -5.40506699 | 1.12E-06 | 5.90E-05 | 5.328537869 |
| MGC45922 | 284365 | 0.479849279 | -0.091969236 | 5.403920793 | 1.12E-06 | 5.91E-05 | 5.32441707 |
| LOC440104 | 440104 | 0.499860554 | -0.134935616 | 5.403368607 | 1.12E-06 | 5.92E-05 | 5.322431962 |
| LOC440345 | 440345 | 0.655867601 | -0.20625816 | 5.401588993 | 1.13E-06 | 5.95E-05 | 5.316034701 |
| SPEF2 | 79925 | 0.420065721 | -0.061293668 | 5.400712851 | 1.14E-06 | 5.96E-05 | 5.312885453 |
| ATR | 545 | 0.566218738 | -0.100809828 | 5.39946329 | 1.14E-06 | 5.97E-05 | 5.308394264 |
| FLJ13305 | 84140 | -0.515252794 | -0.235772234 | -5.399450284 | 1.14E-06 | 5.97E-05 | 5.308347521 |
| FLJ35767 | 400629 | 0.532487039 | -0.136881048 | 5.396475103 | 1.15E-06 | 6.02E-05 | 5.297655494 |
| GPR141 | 353345 | 0.424143841 | -0.055306618 | 5.012205542 | 4.84E-06 | 0.000160247 | 3.934646789 |
| LOC728308 | 728308 | 0.367580354 | -0.106814271 | 5.394603089 | 1.16E-06 | 6.03E-05 | 5.290928973 |
| MUT | 4594 | -0.870436622 | -0.121725793 | -5.394438687 | 1.16E-06 | 6.03E-05 | 5.290338281 |
| WBP11P1 | 441818 | 0.573287525 | -0.02213598 | 5.394287056 | 1.16E-06 | 6.03E-05 | 5.28979348 |
| NT5C1B | 93034 | 0.665775739 | -0.178252471 | 5.394120976 | 1.16E-06 | 6.03E-05 | 5.289196771 |
| EHMT1 | 79813 | 0.476688239 | -0.044374039 | 5.39354332 | 1.17E-06 | 6.04E-05 | 5.287121359 |
| LOC642155 | 642155 | 0.502213184 | -0.0252181 | 5.392836176 | 1.17E-06 | 6.05E-05 | 5.284580821 |
| C21orf74 | 54143 | 0.493715446 | -0.12953412 | 5.391873577 | 1.17E-06 | 6.05E-05 | 5.281122695 |
| KIF5C | 3800 | 0.405245062 | -0.158465934 | 5.390538795 | 1.18E-06 | 6.07E-05 | 5.276327851 |
| CNGA4 | 1262 | 0.666836676 | -0.137230589 | 5.389584476 | 1.18E-06 | 6.09E-05 | 5.272899959 |
| LOC645197 | 645197 | 0.545028576 | -0.098733421 | 5.388611589 | 1.19E-06 | 6.10E-05 | 5.269405584 |
| TBC1D3B | 414059 | 0.752264702 | 0.022171017 | 5.387185373 | 1.20E-06 | 6.13E-05 | 5.264283343 |
| TTC1 | 7265 | -0.749041049 | -0.153465453 | -5.386425488 | 1.20E-06 | 6.14E-05 | 5.26155441 |
| AXIN2 | 8313 | -0.989200232 | -0.02940388 | -5.385465419 | 1.20E-06 | 6.15E-05 | 5.258106753 |
| STRADA | 92335 | 0.578727838 | -0.007197789 | 5.385219708 | 1.20E-06 | 6.15E-05 | 5.257224425 |
| ELOVL3 | 83401 | 0.555206653 | -0.179336836 | 5.38497563 | 1.21E-06 | 6.15E-05 | 5.256347974 |
| LOC653598 | 653598 | 0.556370606 | -0.072982796 | 5.383414636 | 1.21E-06 | 6.18E-05 | 5.250742978 |
| LOC644019 | 644019 | 0.526023155 | -0.055334443 | 5.381642523 | 1.22E-06 | 6.20E-05 | 5.244380591 |
| CLUAP1 | 23059 | 1.488710788 | 0.206293561 | 5.38163375 | 1.22E-06 | 6.20E-05 | 5.244349097 |
| LOC648287 | 648287 | 0.374572101 | -0.070686222 | 5.380855441 | 1.22E-06 | 6.21E-05 | 5.24155497 |
| LAD1 | 3898 | 0.44970905 | -0.100681906 | 5.379841486 | 1.23E-06 | 6.23E-05 | 5.237915083 |
| RCE1 | 9986 | 0.509546466 | 0.046185472 | 5.378412127 | 1.24E-06 | 6.25E-05 | 5.232784374 |
| KLF9 | 687 | -1.091105032 | -0.202202237 | -5.374068928 | 1.26E-06 | 6.34E-05 | 5.217197215 |
| LOC652699 | 652699 | 0.574856235 | -0.096064707 | 5.372501552 | 1.26E-06 | 6.37E-05 | 5.211573158 |
| LOC653319 | 653319 | 0.438561027 | 0.000831719 | 5.371704189 | 1.27E-06 | 6.38E-05 | 5.208712278 |
| LOC647873 | 647873 | 0.472993808 | -0.074139455 | 5.370938373 | 1.27E-06 | 6.39E-05 | 5.205964716 |
| LOC648460 | 648460 | 0.680363824 | -0.082549509 | 5.370761925 | 1.27E-06 | 6.39E-05 | 5.205331686 |
| MAP1LC3B | 81631 | -0.927561935 | -0.137818512 | -5.369963764 | 1.28E-06 | 6.40E-05 | 5.202468253 |
| TMEM159 | 57146 | -0.871987267 | -0.170905542 | -5.364454867 | 1.30E-06 | 6.51E-05 | 5.182708834 |
| SPHAR | 10638 | -0.599564661 | -0.119709679 | -5.364391468 | 1.30E-06 | 6.51E-05 | 5.182481472 |
| PTK6 | 5753 | 0.50264012 | -0.063616893 | 5.364080914 | 1.30E-06 | 6.51E-05 | 5.181367781 |
| SEC15L2 | 23233 | 0.588254095 | -0.084795004 | 5.36386368 | 1.31E-06 | 6.51E-05 | 5.18058876 |
| C21orf51 | 54065 | -0.436820777 | -0.105136856 | -4.434819559 | 3.88E-05 | 0.000652989 | 1.967193049 |
| LOC645364 | 645364 | 0.602454235 | -0.009035604 | 5.361112444 | 1.32E-06 | 6.54E-05 | 5.170723527 |
| CLEC9A | 283420 | 0.453688088 | 0.035734283 | 5.360946707 | 1.32E-06 | 6.54E-05 | 5.170129292 |
| RPLP0 | 6175 | 0.491777259 | -0.095924875 | 5.220064121 | 2.24E-06 | 9.43E-05 | 4.667339682 |
| PRRT3 | 285368 | -0.421189495 | -0.136314115 | -5.360241469 | 1.32E-06 | 6.55E-05 | 5.167600793 |
| OAS1 | 4938 | 0.892768069 | -0.059932123 | 5.359267188 | 1.33E-06 | 6.55E-05 | 5.164107877 |
| MXRA7 | 439921 | -1.288512163 | -0.172337446 | -5.358533241 | 1.33E-06 | 6.55E-05 | 5.161476728 |
| ACBD6 | 84320 | -0.579947387 | -0.067473539 | -5.358316236 | 1.33E-06 | 6.55E-05 | 5.160698806 |
| DCTN3 | 11258 | 0.583504204 | -0.047899335 | 5.356285757 | 1.34E-06 | 6.59E-05 | 5.15342043 |
| SNF8 | 11267 | -0.78775493 | -0.112665045 | -5.35516257 | 1.35E-06 | 6.61E-05 | 5.149394701 |
| MAOA | 4128 | -1.602892028 | -0.219844456 | -5.355073544 | 1.35E-06 | 6.61E-05 | 5.149075627 |
| C17orf76 | 388341 | 0.400218153 | -0.085342761 | 5.354424667 | 1.35E-06 | 6.62E-05 | 5.146750065 |
| NHLH2 | 4808 | 0.569254501 | -0.098451924 | 5.353776295 | 1.36E-06 | 6.62E-05 | 5.144426411 |
| CDS2 | 8760 | -0.857954245 | -0.115135233 | -5.353559782 | 1.36E-06 | 6.62E-05 | 5.143650486 |
| RBMS3 | 27303 | -0.861595657 | 0.027993553 | -5.35301099 | 1.36E-06 | 6.63E-05 | 5.141683809 |
| RNF38 | 152006 | -1.061622751 | -0.160669077 | -5.349628849 | 1.38E-06 | 6.69E-05 | 5.129564951 |
| SMARCD3 | 6604 | -0.625009892 | -0.110968532 | -5.349444078 | 1.38E-06 | 6.69E-05 | 5.128902955 |
| FGF13 | 2258 | -0.881521014 | -0.142865729 | -5.346275089 | 1.39E-06 | 6.76E-05 | 5.117550378 |
| FRMPD2L2 | 728603 | 0.499236332 | -0.071289125 | 5.346198448 | 1.40E-06 | 6.76E-05 | 5.117275848 |
| BMP1 | 649 | 0.551992521 | -0.087320774 | 4.435590618 | 3.87E-05 | 0.000652057 | 1.969744612 |
| C8orf78 | 157376 | 0.526242341 | -0.044558644 | 5.345110939 | 1.40E-06 | 6.77E-05 | 5.113380511 |
| MRFAP1L1 | 114932 | 0.526360683 | -0.09256162 | 5.342225259 | 1.42E-06 | 6.83E-05 | 5.103045643 |
| LOC440900 | 440900 | 0.595518657 | -0.137471823 | 5.340063121 | 1.43E-06 | 6.86E-05 | 5.095303352 |
| SNAPIN | 23557 | -0.537634287 | -0.052198074 | -5.339141554 | 1.43E-06 | 6.87E-05 | 5.092003687 |
| NCF4 | 4689 | 0.663193157 | -0.04167213 | 5.338957419 | 1.43E-06 | 6.87E-05 | 5.091344416 |
| LOC642514 | 642514 | 0.449833109 | -0.080056615 | 5.338868425 | 1.43E-06 | 6.87E-05 | 5.091025788 |
| CYTSB | 92521 | 0.491210319 | -0.0147594 | 5.338344071 | 1.44E-06 | 6.88E-05 | 5.089148461 |
| LOC648682 | 648682 | 0.537973051 | -0.112651748 | 5.335850858 | 1.45E-06 | 6.92E-05 | 5.080222971 |
| AURKAIP1 | 54998 | -0.673456247 | -0.065408536 | -5.333306149 | 1.46E-06 | 6.97E-05 | 5.071114615 |
| PKP1 | 5317 | 0.701704044 | -0.140345529 | 5.331657752 | 1.47E-06 | 7.01E-05 | 5.065215259 |
| LOC647400 | 647400 | 0.483937498 | -0.07163645 | 5.329984522 | 1.48E-06 | 7.04E-05 | 5.059227672 |
| SATB1 | 6304 | 0.762349699 | 0.090087869 | 5.329440876 | 1.49E-06 | 7.04E-05 | 5.057282398 |
| LOC641953 | 641953 | 0.493900396 | -0.085912319 | 5.329186346 | 1.49E-06 | 7.04E-05 | 5.056371662 |
| NOSIP | 51070 | -0.824019086 | -0.151428815 | -5.329034785 | 1.49E-06 | 7.04E-05 | 5.055829369 |
| MLKL | 197259 | 0.527327549 | -0.132927508 | 5.088460633 | 3.65E-06 | 0.000131235 | 4.20211212 |
| PQBP1 | 10084 | -0.547452898 | -0.073206789 | -5.323471972 | 1.52E-06 | 7.18E-05 | 5.035928983 |
| MAP3K7IP1 | 10454 | 0.651472256 | 0.063774832 | 5.321992198 | 1.53E-06 | 7.20E-05 | 5.030636462 |
| ASCC1 | 51008 | -0.761155765 | -0.120228182 | -5.321844358 | 1.53E-06 | 7.20E-05 | 5.030107728 |
| CCDC57 | 284001 | 0.434706088 | -0.090972207 | 5.320778192 | 1.54E-06 | 7.22E-05 | 5.026294861 |
| SPATA5 | 166378 | 0.60169588 | 0.003667768 | 5.319940844 | 1.54E-06 | 7.24E-05 | 5.023300487 |
| ANKRD20A1 | 84210 | 0.385291115 | 0.010924984 | 5.319456133 | 1.54E-06 | 7.24E-05 | 5.021567227 |
| C1orf86 | 199990 | -0.692058185 | -0.041750758 | -5.318164052 | 1.55E-06 | 7.26E-05 | 5.016947185 |
| ZDHHC11 | 79844 | 0.405601792 | -0.081378923 | 5.317394977 | 1.55E-06 | 7.26E-05 | 5.014197422 |
| PTPRG | 5793 | -0.716220201 | -0.07005235 | -5.317359268 | 1.55E-06 | 7.26E-05 | 5.014069752 |
| LOC649092 | 649092 | 0.622079106 | -0.09053605 | 5.315116708 | 1.57E-06 | 7.32E-05 | 5.006052476 |
| ALDH1A2 | 8854 | 0.648895682 | -0.167039553 | 5.314639482 | 1.57E-06 | 7.32E-05 | 5.004346518 |
| BVES | 11149 | -0.72761182 | -0.064181226 | -5.313499439 | 1.58E-06 | 7.34E-05 | 5.000271381 |
| LOC648623 | 648623 | 0.488147778 | -0.077341387 | 5.307516532 | 1.61E-06 | 7.50E-05 | 4.97889021 |
| LOC652479 | 652479 | 0.694511878 | 0.005087407 | 5.304312464 | 1.63E-06 | 7.56E-05 | 4.967443269 |
| LOC649812 | 649812 | 0.451287492 | 0.03146794 | 5.302297338 | 1.65E-06 | 7.61E-05 | 4.960245217 |
| KCNIP3 | 30818 | -0.708298959 | -0.136135426 | -5.30124182 | 1.65E-06 | 7.63E-05 | 4.956475277 |
| DNM1L | 10059 | -0.656479149 | -0.029194167 | -4.769613832 | 1.18E-05 | 0.000290018 | 3.09489961 |
| C1orf89 | 79363 | -0.393205113 | -0.164906688 | -5.296314926 | 1.68E-06 | 7.74E-05 | 4.938881636 |
| KIAA0090 | 23065 | -0.679094887 | -0.138101851 | -5.296232243 | 1.68E-06 | 7.74E-05 | 4.938586428 |
| LOC254028 | 254028 | 0.539153344 | -0.03573034 | 5.294760401 | 1.69E-06 | 7.78E-05 | 4.933331714 |
| ATG4A | 115201 | -0.544147517 | -0.121485418 | -5.292317164 | 1.71E-06 | 7.84E-05 | 4.9246101 |
| NSMCE4A | 54780 | -0.910409449 | -0.131499844 | -5.290930718 | 1.72E-06 | 7.87E-05 | 4.91966154 |
| FER1L4 | 80307 | 0.463541149 | -0.090836138 | 5.290677299 | 1.72E-06 | 7.87E-05 | 4.918757076 |
| PPP2R3B | 28227 | -0.383527588 | -0.131487328 | -5.289859118 | 1.72E-06 | 7.88E-05 | 4.915837057 |
| CENPM | 79019 | 0.490436123 | -0.090688915 | 5.289618947 | 1.73E-06 | 7.88E-05 | 4.914979934 |
| CCT7 | 10574 | -0.804586802 | -0.291593549 | -5.287673927 | 1.74E-06 | 7.93E-05 | 4.908039068 |
| ZDHHC9 | 51114 | -0.912283012 | -0.260046215 | -5.287304163 | 1.74E-06 | 7.93E-05 | 4.906719656 |
| DC36 | 389760 | 0.375311015 | -0.010053182 | 5.286036559 | 1.75E-06 | 7.95E-05 | 4.90219677 |
| PUS1 | 80324 | 0.591204998 | -0.03324865 | 5.285456304 | 1.75E-06 | 7.96E-05 | 4.900126513 |
| VPS26A | 9559 | -0.915337855 | -0.213602473 | -5.285196477 | 1.75E-06 | 7.96E-05 | 4.899199517 |
| ESAM | 90952 | -0.856335997 | -0.107311768 | -5.284866802 | 1.76E-06 | 7.96E-05 | 4.898023346 |
| TASP1 | 55617 | -0.531019959 | -0.043458152 | -5.284142289 | 1.76E-06 | 7.97E-05 | 4.895438613 |
| PRAP1 | 118471 | 0.399244819 | -0.071784558 | 5.281696477 | 1.78E-06 | 8.03E-05 | 4.886714001 |
| TRIM13 | 10206 | 0.505375058 | -0.156907641 | 5.281412911 | 1.78E-06 | 8.03E-05 | 4.885702568 |
| LOC401447 | 401447 | 0.596707821 | -0.121716294 | 5.281385654 | 1.78E-06 | 8.03E-05 | 4.885605348 |
| RPPH1 | 85495 | 1.162640993 | 0.082535898 | 5.280407245 | 1.79E-06 | 8.04E-05 | 4.882115676 |
| HIC1 | 3090 | 0.498666572 | 0.075735139 | 5.277560797 | 1.81E-06 | 8.10E-05 | 4.871964613 |
| PKP3 | 11187 | 0.477920518 | -0.099991961 | 5.275985619 | 1.82E-06 | 8.13E-05 | 4.866348013 |
| LOC651296 | 651296 | 0.427164403 | -0.106719493 | 5.27582908 | 1.82E-06 | 8.13E-05 | 4.865789878 |
| FCN3 | 8547 | -0.549732482 | -0.210375349 | -4.971994423 | 5.62E-06 | 0.000176066 | 3.794256283 |
| HDHD2 | 84064 | -0.897758644 | -0.11216904 | -5.274105127 | 1.83E-06 | 8.17E-05 | 4.85964354 |
| SLC26A8 | 116369 | 0.608768227 | -0.105304841 | 5.273728419 | 1.83E-06 | 8.17E-05 | 4.858300576 |
| MTMR10 | 54893 | 0.468468849 | -0.102378633 | 5.273113115 | 1.84E-06 | 8.18E-05 | 4.856107089 |
| LOC390427 | 390427 | 0.367059732 | -0.079434353 | 5.271251442 | 1.85E-06 | 8.21E-05 | 4.849470994 |
| MRPL38 | 64978 | -0.682186899 | -0.132462333 | -5.270756219 | 1.85E-06 | 8.21E-05 | 4.847705868 |
| PIK3R1 | 5295 | -1.166728585 | -0.140208603 | -5.269827527 | 1.86E-06 | 8.23E-05 | 4.844395886 |
| MDH1 | 4190 | -1.169944182 | -0.209067454 | -5.268180257 | 1.87E-06 | 8.27E-05 | 4.838525312 |
| POLR3F | 10621 | -0.57268155 | -0.077914068 | -5.267960148 | 1.87E-06 | 8.27E-05 | 4.837740932 |
| HPGDS | 27306 | 0.511931883 | -0.087738353 | 5.265366363 | 1.89E-06 | 8.34E-05 | 4.828498613 |
| C8orf54 | 439941 | 0.474846056 | -0.074885573 | 5.263655534 | 1.90E-06 | 8.39E-05 | 4.822403381 |
| PPM1B | 5495 | -1.000064779 | -0.100955181 | -5.263362366 | 1.90E-06 | 8.39E-05 | 4.821358972 |
| LOC651017 | 651017 | 0.434613492 | -0.123110928 | 5.261062617 | 1.92E-06 | 8.45E-05 | 4.813166855 |
| MAP9 | 79884 | -0.411072588 | -0.142829336 | -5.260476685 | 1.93E-06 | 8.45E-05 | 4.811079863 |
| AFARP1 | 246182 | 0.400102184 | -0.069137279 | 5.26010465 | 1.93E-06 | 8.45E-05 | 4.809754781 |
| CBY1 | 25776 | -0.362977645 | -0.164903833 | -5.255968036 | 1.96E-06 | 8.57E-05 | 4.795023612 |
| COX7B | 1349 | -0.932178397 | -0.247503131 | -5.254863401 | 1.97E-06 | 8.59E-05 | 4.791090524 |
| SCAMP1 | 9522 | -0.98198739 | -0.253681641 | -5.253316112 | 1.98E-06 | 8.63E-05 | 4.785581856 |
| RGPD1 | 400966 | 0.481966314 | -0.09668397 | 5.25279988 | 1.98E-06 | 8.64E-05 | 4.783744094 |
| COMMD6 | 170622 | -1.048769849 | -0.244042097 | -5.251955529 | 1.99E-06 | 8.66E-05 | 4.780738383 |
| LOC651612 | 651612 | 0.572315546 | -0.058354648 | 5.250516546 | 2.00E-06 | 8.70E-05 | 4.775616304 |
| ACVR1 | 90 | -1.015291904 | -0.192267194 | -5.250149377 | 2.00E-06 | 8.70E-05 | 4.774309443 |
| CYC1 | 1537 | -0.96026915 | -0.273679794 | -5.249052032 | 2.01E-06 | 8.72E-05 | 4.770403868 |
| C19orf12 | 83636 | -0.896994995 | -0.168873606 | -5.247939026 | 2.02E-06 | 8.75E-05 | 4.766442856 |
| LOC730760 | 730760 | 0.436305393 | 0.018525522 | 5.247026979 | 2.02E-06 | 8.76E-05 | 4.763197248 |
| C14orf4 | 64207 | -1.363327352 | -0.058299992 | -5.246929338 | 2.03E-06 | 8.76E-05 | 4.762849798 |
| CCT3 | 7203 | -0.80541933 | -0.08370617 | -5.24535985 | 2.04E-06 | 8.81E-05 | 4.757265137 |
| CEP152 | 22995 | 0.390147564 | -0.028975148 | 5.243446761 | 2.05E-06 | 8.86E-05 | 4.75045867 |
| ZDHHC19 | 131540 | 0.65508245 | -0.131013451 | 5.242767088 | 2.06E-06 | 8.86E-05 | 4.748040716 |
| TGM3 | 7053 | 0.422626037 | -0.081181264 | 5.242743215 | 2.06E-06 | 8.86E-05 | 4.747955788 |
| ZNF689 | 115509 | -0.853395548 | -0.140406622 | -5.24232608 | 2.06E-06 | 8.87E-05 | 4.746471879 |
| LOC641693 | 641693 | 0.536565345 | -0.017386461 | 5.241516351 | 2.07E-06 | 8.89E-05 | 4.743591483 |
| CEP170L | 645455 | 0.627723642 | -0.127978879 | 5.239710333 | 2.08E-06 | 8.94E-05 | 4.737167633 |
| LOC642891 | 642891 | 0.442534911 | -0.068062122 | 5.23923769 | 2.08E-06 | 8.94E-05 | 4.735486615 |
| NLRP1 | 22861 | 0.701494176 | 0.035270489 | 5.237886035 | 2.10E-06 | 8.98E-05 | 4.730679578 |
| HOXC4 | 3221 | -0.948234426 | -0.16497477 | -5.237546172 | 2.10E-06 | 8.98E-05 | 4.729470958 |
| LOC654056 | 654056 | 0.638561827 | -0.118230754 | 5.236997178 | 2.10E-06 | 8.98E-05 | 4.727518684 |
| BRD7 | 29117 | -0.816026208 | -0.150812279 | -5.236988005 | 2.10E-06 | 8.98E-05 | 4.727486062 |
| CAMKK1 | 84254 | 0.509169205 | -0.041821592 | 5.236661301 | 2.10E-06 | 8.98E-05 | 4.726324309 |
| KRTAP10-12 | 386685 | 0.524974184 | -0.104815439 | 5.234545247 | 2.12E-06 | 9.04E-05 | 4.718800294 |
| DAG1 | 1605 | -0.929619403 | -0.133140702 | -5.232833517 | 2.13E-06 | 9.09E-05 | 4.712714734 |
| LOC646774 | 646774 | 0.407274621 | -0.029154221 | 5.230868201 | 2.15E-06 | 9.15E-05 | 4.705728511 |
| LOC649604 | 649604 | 0.590784401 | -0.121803524 | 5.229807018 | 2.16E-06 | 9.16E-05 | 4.701956659 |
| ACPP | 55 | 0.495045888 | -0.048947052 | 5.229419047 | 2.16E-06 | 9.17E-05 | 4.70057773 |
| LOC642987 | 642987 | 0.646584823 | -0.126248705 | 5.225720392 | 2.19E-06 | 9.28E-05 | 4.687433821 |
| ZCCHC14 | 23174 | -0.804116087 | -0.076561557 | -5.225576555 | 2.19E-06 | 9.28E-05 | 4.686922737 |
| LSM14A | 26065 | -0.898508594 | -0.188350535 | -5.222403426 | 2.22E-06 | 9.37E-05 | 4.675649198 |
| LOC440349 | 440349 | 0.434158519 | -0.016752187 | 5.220496003 | 2.24E-06 | 9.43E-05 | 4.668873679 |
| MRPL45 | 84311 | -0.90476175 | -0.166220843 | -5.218936716 | 2.25E-06 | 9.45E-05 | 4.663335476 |
| BNIPL | 149428 | 0.388090241 | -0.095202662 | 5.218799169 | 2.25E-06 | 9.45E-05 | 4.662846975 |
| SHMT2 | 6472 | -0.90569554 | -0.16593406 | -5.218134998 | 2.26E-06 | 9.45E-05 | 4.660488206 |
| ST3GAL1 | 6482 | -1.078018405 | -0.180193057 | -5.217559208 | 2.26E-06 | 9.45E-05 | 4.658443411 |
| DIXDC1 | 85458 | -1.035543406 | -0.168769573 | -5.217434133 | 2.26E-06 | 9.45E-05 | 4.657999244 |
| VNN3 | 55350 | 0.511160842 | -0.096323649 | 5.217265905 | 2.26E-06 | 9.45E-05 | 4.657401841 |
| EPB41L4B | 54566 | 0.553157798 | -0.055625929 | 5.21719562 | 2.26E-06 | 9.45E-05 | 4.657152246 |
| TSC22D1 | 8848 | 0.383224158 | -0.102676254 | 5.215598465 | 2.28E-06 | 9.50E-05 | 4.651480863 |
| LOC653225 | 653225 | 0.505793733 | -0.121402861 | 5.215245886 | 2.28E-06 | 9.50E-05 | 4.650228968 |
| LOC728095 | 728095 | 0.520315896 | -0.099705886 | 5.213263478 | 2.30E-06 | 9.56E-05 | 4.643190637 |
| FAM181B | 220382 | -0.668208741 | -0.218023323 | -5.21277097 | 2.30E-06 | 9.57E-05 | 4.641442191 |
| FLAD1 | 80308 | 0.406664583 | -0.056899536 | 5.211428856 | 2.31E-06 | 9.61E-05 | 4.636677878 |
| MAP2 | 4133 | -0.415762878 | -0.184132917 | -5.211125522 | 2.32E-06 | 9.61E-05 | 4.635601149 |
| UBB | 7314 | -1.913068808 | -0.325212625 | -5.210931561 | 2.32E-06 | 9.61E-05 | 4.634912666 |
| ZNF519 | 162655 | 0.482611528 | -0.001387297 | 4.333539601 | 5.53E-05 | 0.000821288 | 1.633999506 |
| INTS3 | 65123 | -0.85250526 | -0.048568951 | -5.21015694 | 2.32E-06 | 9.61E-05 | 4.632163171 |
| NDUFAF2 | 91942 | -0.875325497 | -0.139651448 | -5.207805365 | 2.34E-06 | 9.67E-05 | 4.62381725 |
| EBF2 | 64641 | 0.592152701 | -0.139747466 | 5.206483593 | 2.36E-06 | 9.70E-05 | 4.619126783 |
| FLJ45422 | 441140 | 0.445400298 | -0.016940114 | 5.20611603 | 2.36E-06 | 9.70E-05 | 4.617822521 |
| ZNF155 | 7711 | 0.492495801 | -0.044658535 | 5.205862305 | 2.36E-06 | 9.70E-05 | 4.616922221 |
| CSNK1G3 | 1456 | 0.479757436 | -0.140627035 | 5.205286896 | 2.37E-06 | 9.71E-05 | 4.614880538 |
| SPDYC | 387778 | 0.465987287 | -0.081682647 | 5.204300236 | 2.38E-06 | 9.73E-05 | 4.611379841 |
| PHRF1 | 57661 | 0.859580948 | 0.084104509 | 5.203020322 | 2.39E-06 | 9.76E-05 | 4.606839031 |
| TDG | 6996 | -0.77325444 | -0.159841997 | -5.202958568 | 2.39E-06 | 9.76E-05 | 4.606619955 |
| KIAA0368 | 23392 | -0.861558597 | -0.152868655 | -5.198636618 | 2.43E-06 | 9.88E-05 | 4.591289877 |
| CYP4F3 | 4051 | 0.627118236 | 0.034555568 | 5.1972437 | 2.44E-06 | 9.91E-05 | 4.58635016 |
| NDUFS3 | 4722 | -0.845892527 | -0.189892809 | -5.19616165 | 2.45E-06 | 9.94E-05 | 4.582513214 |
| CRBN | 51185 | -0.842457203 | -0.04358215 | -5.195678553 | 2.45E-06 | 9.95E-05 | 4.58080025 |
| ZNF426 | 79088 | -0.655453001 | -0.122410281 | -5.194962568 | 2.46E-06 | 9.97E-05 | 4.578261618 |
| UGCGL1 | 56886 | -0.706085382 | -0.094710087 | -5.192071222 | 2.49E-06 | 0.000100637 | 4.568011234 |
| KCNH4 | 23415 | 0.454695676 | -0.000478072 | 5.191335609 | 2.49E-06 | 0.00010081 | 4.565403679 |
| PRMT5 | 10419 | -0.494641716 | -0.090617987 | -4.499630399 | 3.09E-05 | 0.000561801 | 2.182429669 |
| XAGE1 | 9503 | 0.747084519 | -0.058142726 | 5.187728036 | 2.53E-06 | 0.000101878 | 4.552617762 |
| PAX8 | 7849 | 0.537416505 | -0.126341172 | 5.187659296 | 2.53E-06 | 0.000101878 | 4.552374167 |
| FLJ42220 | 400207 | 0.409014803 | 0.018201463 | 5.186871202 | 2.53E-06 | 0.000101878 | 4.549581464 |
| TXNDC3 | 51314 | 0.427481541 | -0.067379147 | 5.186633169 | 2.54E-06 | 0.000101878 | 4.548737997 |
| DDT | 1652 | -0.846224945 | -0.188742941 | -5.186321943 | 2.54E-06 | 0.000101878 | 4.547635197 |
| KLK5 | 25818 | 0.583377407 | -0.10134889 | 5.183606308 | 2.57E-06 | 0.00010281 | 4.538013617 |
| IMMP2L | 83943 | -0.697519766 | -0.114903019 | -5.179678058 | 2.60E-06 | 0.000104115 | 4.524099015 |
| CD1A | 909 | 0.510084852 | -0.035105051 | 5.177900247 | 2.62E-06 | 0.000104702 | 4.517802964 |
| LOC653853 | 653853 | 0.606983531 | 0.002596563 | 5.177575644 | 2.62E-06 | 0.000104722 | 4.516653482 |
| LONP1 | 9361 | -0.639515404 | -0.159780337 | -5.174781602 | 2.65E-06 | 0.000105712 | 4.506760343 |
| LOC643595 | 643595 | 0.574452036 | -0.067648163 | 5.17357408 | 2.66E-06 | 0.000105921 | 4.502485366 |
| GPX4 | 2879 | -0.871065044 | -0.245869282 | -5.173377623 | 2.67E-06 | 0.000105921 | 4.501789887 |
| ERLIN1 | 10613 | -0.483878099 | -0.10504642 | -5.173327367 | 2.67E-06 | 0.000105921 | 4.501611978 |
| PREB | 10113 | -0.635822064 | -0.057066125 | -5.173017017 | 2.67E-06 | 0.000105921 | 4.500513329 |
| C3orf70 | 285382 | -0.979716992 | 0.023654188 | -4.456696189 | 3.59E-05 | 0.00062135 | 2.039672298 |
| CCNI | 10983 | -0.954623297 | -0.253130257 | -5.171093297 | 2.69E-06 | 0.000106528 | 4.493703846 |
| NSFL1C | 55968 | -0.897708953 | -0.20139442 | -5.170513831 | 2.69E-06 | 0.000106652 | 4.49165287 |
| MGC52282 | 124221 | 0.611795278 | -0.124089694 | 5.169917076 | 2.70E-06 | 0.000106782 | 4.489540788 |
| DDX46 | 9879 | -0.662958953 | -0.111829716 | -5.169485514 | 2.70E-06 | 0.000106846 | 4.488013428 |
| NR2C1 | 7181 | 0.385429853 | -0.057476487 | 5.168938649 | 2.71E-06 | 0.000106957 | 4.486078063 |
| ADAMTS8 | 11095 | -1.139858514 | -0.012401762 | -5.166960924 | 2.73E-06 | 0.000107588 | 4.479079496 |
| LOC127295 | 127295 | -0.894992972 | -0.186996715 | -5.166790994 | 2.73E-06 | 0.000107588 | 4.478478212 |
| LOC651301 | 651301 | 0.506929389 | -0.047790412 | 5.16655609 | 2.73E-06 | 0.000107588 | 4.477647035 |
| PUM1 | 9698 | -1.056738832 | -0.237493724 | -5.165758452 | 2.74E-06 | 0.0001078 | 4.474824802 |
| GLIS3 | 169792 | -0.754698973 | -0.010636344 | -5.164631811 | 2.75E-06 | 0.000108146 | 4.470838757 |
| PHF17 | 79960 | -0.32445144 | -0.13986641 | -4.886241578 | 7.69E-06 | 0.000220765 | 3.496420354 |
| LOC391811 | 391811 | -0.687939439 | -0.165843311 | -5.16312956 | 2.77E-06 | 0.000108536 | 4.465524315 |
| FBXL12 | 54850 | -0.53411001 | -0.085955699 | -5.161732843 | 2.78E-06 | 0.000108886 | 4.460583735 |
| ATP6V1G2 | 534 | 0.479118538 | 0.003928559 | 5.159980009 | 2.80E-06 | 0.00010949 | 4.454384178 |
| NKIRAS1 | 28512 | -0.854593439 | -0.194180831 | -5.153502551 | 2.87E-06 | 0.000112052 | 4.431481074 |
| LOC646734 | 646734 | 0.450648294 | -0.035128203 | 5.153077061 | 2.88E-06 | 0.000112088 | 4.429976999 |
| PDZD3 | 79849 | 0.366686564 | -0.06513416 | 5.152883325 | 2.88E-06 | 0.000112088 | 4.429292173 |
| FAHD1 | 81889 | -0.55236822 | -0.150405929 | -4.37106597 | 4.85E-05 | 0.000757983 | 1.756996998 |
| PVRL3 | 25945 | -0.615902154 | -0.134055765 | -5.150559399 | 2.90E-06 | 0.000112839 | 4.421078207 |
| FBXW4 | 6468 | -0.684923983 | -0.133546125 | -5.149262268 | 2.92E-06 | 0.000113162 | 4.416494076 |
| AGXT2L2 | 85007 | 0.55435746 | -0.014092159 | 5.147895108 | 2.93E-06 | 0.000113627 | 4.411662931 |
| CDK10 | 8558 | 0.364144946 | -0.060088176 | 5.147600378 | 2.93E-06 | 0.00011364 | 4.410621506 |
| LOC645408 | 645408 | 0.570417192 | 0.014288554 | 5.14652234 | 2.95E-06 | 0.000113985 | 4.406812458 |
| NHSL2 | 340527 | 0.314689518 | -0.028665161 | 5.145910096 | 2.95E-06 | 0.000114132 | 4.404649342 |
| PLXNA4B | 91584 | 0.554635163 | -0.052868297 | 5.145461549 | 2.96E-06 | 0.000114191 | 4.403064644 |
| NARF | 26502 | -0.730709159 | -0.081905754 | -5.144940766 | 2.96E-06 | 0.000114209 | 4.401224803 |
| DEFB131 | 644414 | 0.66747847 | -0.098128749 | 5.144452911 | 2.97E-06 | 0.000114305 | 4.39950136 |
| C20orf127 | 140851 | -1.017922392 | -0.170259126 | -5.141024691 | 3.01E-06 | 0.000115546 | 4.387392221 |
| MYL1 | 4632 | 0.559669745 | -0.096208722 | 5.1397683 | 3.02E-06 | 0.000115974 | 4.38295517 |
| SMG5 | 23381 | -0.665333715 | -0.185162288 | -5.138296175 | 3.04E-06 | 0.000116497 | 4.377756759 |
| RNF208 | 727800 | 0.409079518 | -0.071599568 | 5.137421759 | 3.05E-06 | 0.000116688 | 4.374669265 |
| FAM153B | 202134 | 1.161330532 | -0.024288458 | 5.137223303 | 3.05E-06 | 0.000116688 | 4.373968558 |
| POLR2I | 5438 | -0.60735143 | -0.05571413 | -5.137070994 | 3.05E-06 | 0.000116688 | 4.373430795 |
| ZNF37A | 7587 | 0.460829272 | -0.049649476 | 5.136552885 | 3.06E-06 | 0.0001168 | 4.371601533 |
| ZFR | 51663 | -0.951760325 | -0.179775102 | -5.136291592 | 3.06E-06 | 0.0001168 | 4.370679027 |
| LOC651914 | 651914 | 0.479876824 | -0.172961374 | 5.135377676 | 3.07E-06 | 0.000117085 | 4.36745254 |
| LONP2 | 83752 | -0.67745583 | -0.051603231 | -5.134365953 | 3.08E-06 | 0.000117412 | 4.363881011 |
| SIK1 | 150094 | 2.169663275 | 0.645896528 | 5.133792477 | 3.09E-06 | 0.000117543 | 4.361856678 |
| GK5 | 256356 | -0.578820525 | -0.120048077 | -5.133547508 | 3.09E-06 | 0.000117543 | 4.360991978 |
| LOC652501 | 652501 | 0.551185577 | -0.104322923 | 5.132748251 | 3.10E-06 | 0.000117779 | 4.358170844 |
| LOC646521 | 646521 | 0.623783042 | -0.062534934 | 5.131249467 | 3.12E-06 | 0.000118323 | 4.352881046 |
| LOC651381 | 651381 | 0.53133891 | -0.025249733 | 5.129570996 | 3.14E-06 | 0.000118777 | 4.346957755 |
| COL9A3 | 1299 | 0.523627408 | -0.139567755 | 5.12954276 | 3.14E-06 | 0.000118777 | 4.346858118 |
| ZNF354B | 117608 | 0.478320659 | -0.04834504 | 5.129444178 | 3.14E-06 | 0.000118777 | 4.346510248 |
| LOC651285 | 651285 | 0.502503098 | -0.125799151 | 5.129015955 | 3.14E-06 | 0.000118837 | 4.344999188 |
| C1QTNF6 | 114904 | 0.609036313 | 0.006071821 | 5.128792168 | 3.15E-06 | 0.000118837 | 4.344209537 |
| CAPN2 | 824 | -0.600621354 | -0.064816629 | -5.127761049 | 3.16E-06 | 0.000119125 | 4.340571317 |
| DNAJC6 | 9829 | 0.491362498 | -0.019811596 | 5.127624046 | 3.16E-06 | 0.000119125 | 4.340087933 |
| ATP5S | 27109 | -0.425555974 | -0.102906054 | -5.126851359 | 3.17E-06 | 0.000119341 | 4.337361778 |
| LOC642398 | 642398 | 0.480064267 | -0.075506679 | 5.126463941 | 3.17E-06 | 0.000119341 | 4.335994969 |
| ZNF25 | 219749 | -1.074846216 | -0.216827369 | -5.126311477 | 3.18E-06 | 0.000119341 | 4.335457091 |
| LOC389137 | 389137 | -0.809984098 | -0.130259202 | -5.126110544 | 3.18E-06 | 0.000119341 | 4.334748222 |
| SPDYE1 | 285955 | 0.658191876 | -0.035523267 | 5.124544504 | 3.20E-06 | 0.000119923 | 4.329223786 |
| C1orf168 | 199920 | 0.448733127 | -0.087504991 | 5.123640502 | 3.21E-06 | 0.000120212 | 4.326035079 |
| LOC729486 | 729486 | 0.554436636 | -0.046115811 | 4.431180764 | 3.93E-05 | 0.000658576 | 1.955154672 |
| MYBPC3 | 4607 | 0.505288759 | -0.152199386 | 5.122647105 | 3.22E-06 | 0.000120427 | 4.322531293 |
| FOXRED2 | 80020 | 0.446511634 | -0.062045479 | 5.121500026 | 3.23E-06 | 0.000120827 | 4.318485784 |
| PIGM | 93183 | -0.68094617 | -0.142983983 | -5.120155755 | 3.25E-06 | 0.000121317 | 4.313745259 |
| ATPAF1 | 64756 | -0.794084691 | -0.112564921 | -5.119520104 | 3.26E-06 | 0.000121488 | 4.311503824 |
| DENND4C | 55667 | -0.987042721 | -0.106010491 | -5.118838559 | 3.27E-06 | 0.000121681 | 4.309100677 |
| C11orf49 | 79096 | 0.374606101 | -0.079791573 | 5.116824702 | 3.29E-06 | 0.000122401 | 4.302000468 |
| SH3RF2 | 153769 | -0.415334982 | -0.196085514 | -5.116741215 | 3.29E-06 | 0.000122401 | 4.301706145 |
| TBC1D16 | 125058 | -0.67646469 | -0.063545651 | -5.115788038 | 3.30E-06 | 0.000122605 | 4.298345942 |
| AKR1A1 | 10327 | 0.470181204 | -0.172256999 | 5.115685489 | 3.30E-06 | 0.000122605 | 4.297984444 |
| MIAT | 440823 | 1.128206861 | 0.191060747 | 5.115531943 | 3.31E-06 | 0.000122605 | 4.297443184 |
| OR11H4 | 390442 | 0.506651644 | -0.067387768 | 5.113873455 | 3.33E-06 | 0.000123001 | 4.291597262 |
| LOC642859 | 642859 | 0.492810357 | -0.108420301 | 5.11339854 | 3.33E-06 | 0.000123001 | 4.289923388 |
| AK2 | 204 | -0.659068655 | -0.076276788 | -5.112192078 | 3.35E-06 | 0.000123323 | 4.285671398 |
| C1orf151 | 440574 | -0.57613382 | -0.293424721 | -5.111274751 | 3.36E-06 | 0.000123628 | 4.282438678 |
| C1orf91 | 56063 | 0.426185572 | -0.057532338 | 5.111003419 | 3.36E-06 | 0.000123637 | 4.281482527 |
| JMJD1C | 221037 | 0.564359747 | -0.144857524 | 5.11045766 | 3.37E-06 | 0.000123772 | 4.279559383 |
| CLEC2A | 387836 | 0.611199134 | -0.040588497 | 5.109863089 | 3.38E-06 | 0.00012393 | 4.277464323 |
| ADAMTS13 | 11093 | 0.435156539 | 0.022732326 | 5.109589772 | 3.38E-06 | 0.00012394 | 4.27650128 |
| LOC649186 | 649186 | 0.505351093 | -0.135222157 | 5.108510058 | 3.39E-06 | 0.000124207 | 4.272697063 |
| MGC3196 | 79064 | -0.49298201 | -0.060850178 | -5.107666961 | 3.40E-06 | 0.00012448 | 4.269726748 |
| FKBP10 | 60681 | -0.630517447 | -0.179688389 | -5.10651273 | 3.42E-06 | 0.000124899 | 4.265660581 |
| DIMT1L | 27292 | -0.874094474 | -0.135320513 | -5.103892928 | 3.45E-06 | 0.000126002 | 4.25643276 |
| SMC1B | 27127 | 0.474365934 | -0.073116718 | 5.102209146 | 3.47E-06 | 0.000126557 | 4.250502875 |
| RPL27A | 6157 | -0.76062587 | -0.219596096 | -5.101286576 | 3.49E-06 | 0.000126874 | 4.247254121 |
| FAM188A | 80013 | -0.872718847 | -0.205103371 | -5.100126833 | 3.50E-06 | 0.000127303 | 4.243170503 |
| SCUBE3 | 222663 | -0.605421732 | -0.220913128 | -5.099853054 | 3.50E-06 | 0.000127315 | 4.242206538 |
| ELP3 | 55140 | -0.827108613 | -0.116560448 | -5.096401661 | 3.55E-06 | 0.000128836 | 4.230056068 |
| MARCHF6 | 10299 | -1.082302122 | -0.273958568 | -5.095940298 | 3.55E-06 | 0.000128938 | 4.228432101 |
| RPL34 | 6164 | -0.628872895 | -0.157062704 | -4.260808666 | 7.11E-05 | 0.000972519 | 1.397189106 |
| C20orf52 | 140823 | -0.632868676 | -0.084043282 | -5.092111007 | 3.61E-06 | 0.000130542 | 4.21495545 |
| MMP14 | 4323 | -0.43822204 | -0.244882821 | -5.090816742 | 3.62E-06 | 0.000130786 | 4.210401352 |
| LOC154872 | 154872 | 0.435348224 | -0.035289051 | 5.09077534 | 3.62E-06 | 0.000130786 | 4.210255681 |
| IL9R | 3581 | 0.624858804 | -0.165558241 | 5.090707168 | 3.62E-06 | 0.000130786 | 4.210015818 |
| AKR1C3 | 8644 | -1.114824706 | -0.28391034 | -5.090616927 | 3.63E-06 | 0.000130786 | 4.209698307 |
| UBE2G2 | 7327 | -0.697104338 | -0.095439191 | -5.090288869 | 3.63E-06 | 0.000130826 | 4.208544067 |
| LOC645172 | 645172 | 0.34406383 | -0.066670948 | 5.089711618 | 3.64E-06 | 0.000130977 | 4.206513136 |
| ARL17P1 | 51326 | 1.032892859 | 0.152266011 | 5.089288209 | 3.64E-06 | 0.000130977 | 4.205023517 |
| SETD3 | 84193 | -0.813795457 | -0.165181016 | -5.089237146 | 3.64E-06 | 0.000130977 | 4.204843874 |
| LILRA3 | 11026 | 0.758979095 | -0.308559527 | 5.087486292 | 3.67E-06 | 0.000131552 | 4.198684641 |
| SCMH1 | 22955 | -0.747034246 | -0.068135487 | -5.087318159 | 3.67E-06 | 0.000131552 | 4.19809322 |
| NOTCH2 | 4853 | -0.670420479 | -0.035586996 | -5.085555472 | 3.69E-06 | 0.000132294 | 4.191893269 |
| LOC253820 | 253820 | 0.533380148 | -0.094268255 | 5.083756967 | 3.72E-06 | 0.000133058 | 4.185568199 |
| C15orf44 | 81556 | 0.503518245 | -0.089144393 | 4.432436304 | 3.91E-05 | 0.000656509 | 1.959307871 |
| CNIH4 | 29097 | -1.017966083 | -0.24029067 | -5.082471918 | 3.74E-06 | 0.000133451 | 4.181049406 |
| NIPAL4 | 348938 | 0.674697922 | -0.211227108 | 5.081088821 | 3.76E-06 | 0.000134015 | 4.17618633 |
| LOC642846 | 642846 | 0.609213477 | -0.085297168 | 5.077167404 | 3.81E-06 | 0.00013573 | 4.162401122 |
| THAP11 | 57215 | -0.850094451 | -0.17350895 | -5.075380466 | 3.84E-06 | 0.000136508 | 4.156120758 |
| GLYCAM1 | 644076 | 0.618686377 | -0.136667969 | 5.0750973 | 3.84E-06 | 0.000136528 | 4.155125624 |
| LOC284837 | 284837 | 0.432624667 | -0.063611991 | 5.074716074 | 3.85E-06 | 0.000136597 | 4.15378591 |
| BCAP29 | 55973 | -0.645941038 | -0.086271152 | -5.07419531 | 3.85E-06 | 0.000136738 | 4.151955891 |
| LOC653392 | 653392 | 0.428954729 | -0.05383471 | 5.071591024 | 3.89E-06 | 0.000137938 | 4.14280526 |
| RBMS1 | 5937 | -0.844336509 | -0.152429835 | -4.713582761 | 1.44E-05 | 0.000330025 | 2.903519472 |
| LOC283412 | 283412 | -0.84802555 | -0.108839207 | -5.069566964 | 3.92E-06 | 0.000138602 | 4.135694626 |
| PRPF19 | 27339 | -0.605745948 | -0.033943552 | -5.06701896 | 3.96E-06 | 0.000139555 | 4.126744925 |
| JUP | 3728 | 0.382470198 | -0.020632971 | 5.066988789 | 3.96E-06 | 0.000139555 | 4.126638961 |
| LRRC1 | 55227 | -0.810448385 | -0.12707866 | -5.066656717 | 3.96E-06 | 0.000139576 | 4.125472711 |
| TGFB1I1 | 7041 | -1.14525965 | -0.160040709 | -5.064178425 | 4.00E-06 | 0.000140612 | 4.1167698 |
| P2RY13 | 53829 | 0.472817137 | -0.060316205 | 5.063984571 | 4.00E-06 | 0.000140612 | 4.116089123 |
| PPARBP | 5469 | -0.812891568 | -0.173561306 | -5.062579588 | 4.02E-06 | 0.000141125 | 4.111156122 |
| C6orf78 | 221301 | 0.596387558 | -0.104092152 | 5.062518257 | 4.02E-06 | 0.000141125 | 4.110940797 |
| PCYT2 | 5833 | -0.537101174 | -0.101740609 | -5.06016382 | 4.06E-06 | 0.000142233 | 4.10267543 |
| ANKRD58 | 347454 | 0.570658057 | 0.039927299 | 5.056799082 | 4.11E-06 | 0.000143886 | 4.090865973 |
| C14orf101 | 54916 | 0.434631755 | -0.045549658 | 5.056282661 | 4.12E-06 | 0.00014391 | 4.089053727 |
| MPPED1 | 758 | 0.444347612 | -0.059104848 | 5.056274704 | 4.12E-06 | 0.00014391 | 4.089025806 |
| IL17B | 27190 | -0.806601304 | -0.142196453 | -5.056016432 | 4.12E-06 | 0.00014392 | 4.088119493 |
| SLC14A1 | 6563 | -0.475487058 | -0.215308499 | -5.054232709 | 4.15E-06 | 0.000144616 | 4.08186067 |
| TMED4 | 222068 | -1.255082578 | -0.361388516 | -5.053684623 | 4.16E-06 | 0.000144782 | 4.07993769 |
| CROT | 54677 | -0.623948136 | -0.107713913 | -5.053174269 | 4.16E-06 | 0.000144927 | 4.078147168 |
| LOC654114 | 654114 | 0.448213164 | 0.001343532 | 5.051588997 | 4.19E-06 | 0.000145628 | 4.072585868 |
| ATM | 472 | 0.582385478 | -0.095742877 | 5.051391414 | 4.19E-06 | 0.000145628 | 4.071892772 |
| THAP10 | 56906 | -0.480841324 | -0.093042995 | -5.048912663 | 4.23E-06 | 0.000146839 | 4.063198567 |
| IGFBP5 | 3488 | -1.307879349 | -0.374415474 | -5.048601142 | 4.24E-06 | 0.000146879 | 4.062106026 |
| BTNL3 | 10917 | 0.517103965 | -0.064281124 | 5.048260343 | 4.24E-06 | 0.000146935 | 4.060910837 |
| LOC644548 | 644548 | 0.454514361 | -0.053492131 | 4.374975699 | 4.79E-05 | 0.000750764 | 1.769842916 |
| DSC2 | 1824 | 0.390287902 | -0.03950147 | 5.046948601 | 4.26E-06 | 0.00014739 | 4.056310823 |
| MAP3K5 | 4217 | -0.796069013 | -0.161310992 | -5.046064938 | 4.28E-06 | 0.000147613 | 4.053212263 |
| TULP4 | 56995 | 0.581185763 | -0.031431133 | 5.045143443 | 4.29E-06 | 0.000147987 | 4.049981271 |
| XAGE2 | 9502 | 0.524392647 | -0.085118464 | 5.044085225 | 4.31E-06 | 0.000148437 | 4.046271185 |
| SHFM1 | 7979 | -0.693256933 | -0.304240947 | -5.042401768 | 4.33E-06 | 0.000149233 | 4.040369658 |
| WFS1 | 7466 | -1.123637913 | -0.278946095 | -5.042062258 | 4.34E-06 | 0.00014929 | 4.039179565 |
| LOC644068 | 644068 | 0.494726193 | -0.004263919 | 5.041694072 | 4.35E-06 | 0.000149304 | 4.03788899 |
| KRTAP19-6 | 337973 | 0.370526496 | 0.056868564 | 5.04156458 | 4.35E-06 | 0.000149304 | 4.037435101 |
| LOC653349 | 653349 | 0.717010185 | -0.160082241 | 5.04107113 | 4.36E-06 | 0.000149446 | 4.035705521 |
| ATXN2 | 6311 | -0.757552685 | -0.12510784 | -5.040392623 | 4.37E-06 | 0.00014967 | 4.033327416 |
| C10orf53 | 282966 | 0.547977792 | -0.032298001 | 5.040195688 | 4.37E-06 | 0.00014967 | 4.0326372 |
| PRKACA | 5566 | -0.304143765 | -0.082566537 | -5.039923538 | 4.37E-06 | 0.00014969 | 4.031683392 |
| LOC389435 | 389435 | -0.79389581 | -0.199632173 | -5.038492186 | 4.40E-06 | 0.000150353 | 4.026667233 |
| LOC654192 | 654192 | 0.370036517 | -0.053869909 | 5.037786357 | 4.41E-06 | 0.000150615 | 4.024193868 |
| PDE8B | 8622 | -1.06850166 | -0.045648175 | -5.037550551 | 4.41E-06 | 0.000150616 | 4.023367588 |
| PAPD5 | 64282 | -0.81006163 | -0.060400617 | -5.035886302 | 4.44E-06 | 0.000151413 | 4.017536387 |
| ZNF696 | 79943 | -0.431142625 | -0.089201496 | -5.034703467 | 4.46E-06 | 0.000151944 | 4.013392428 |
| LOC729355 | 729355 | 0.586705942 | -0.127740392 | 5.032819954 | 4.49E-06 | 0.000152873 | 4.006794508 |
| LOC730118 | 730118 | 0.610209211 | -0.034653124 | 5.031617016 | 4.51E-06 | 0.00015342 | 4.002581144 |
| FEZ1 | 9638 | -1.268190656 | -0.45871873 | -5.03122159 | 4.52E-06 | 0.000153512 | 4.001196225 |
| LOC150207 | 150207 | 0.387231245 | -0.008442877 | 5.030957781 | 4.52E-06 | 0.000153529 | 4.000272301 |
| ZNF555 | 148254 | 0.334099927 | -0.139463834 | 5.029539407 | 4.54E-06 | 0.00015407 | 3.995305133 |
| SCRN2 | 90507 | 0.436796176 | -0.000963187 | 5.029212566 | 4.55E-06 | 0.000154123 | 3.99416061 |
| LOC643012 | 643012 | 0.545052198 | -0.123007223 | 5.026545026 | 4.60E-06 | 0.000155381 | 3.984820589 |
| GRTP1 | 79774 | 0.467300623 | -0.081817335 | 5.02524585 | 4.62E-06 | 0.000155994 | 3.980272421 |
| PPP3CB | 5532 | -1.046812749 | -0.238040265 | -5.024510762 | 4.63E-06 | 0.000156087 | 3.977699227 |
| TMEM203 | 94107 | -0.779169032 | -0.104986618 | -5.024387034 | 4.63E-06 | 0.000156087 | 3.977266129 |
| LOC255809 | 255809 | 0.686781557 | 0.008691864 | 5.023934213 | 4.64E-06 | 0.000156087 | 3.975681106 |
| FAM126A | 84668 | 0.486676497 | -0.022105178 | 5.023745771 | 4.64E-06 | 0.000156087 | 3.975021513 |
| KIAA1161 | 57462 | -0.389081998 | -0.186162975 | -5.023559645 | 4.65E-06 | 0.000156087 | 3.974370038 |
| INPP5A | 3632 | 0.364534702 | -0.111061354 | 4.347399602 | 5.27E-05 | 0.000796732 | 1.679363853 |
| BNIP1 | 662 | 0.474076121 | -0.029835617 | 5.021179916 | 4.69E-06 | 0.00015704 | 3.966041391 |
| ODZ1 | 10178 | 0.583252971 | -0.121387719 | 5.021121299 | 4.69E-06 | 0.00015704 | 3.965836258 |
| LOC650517 | 650517 | 0.572229117 | -0.147097275 | 5.0195383 | 4.72E-06 | 0.000157826 | 3.960296928 |
| FBXO9 | 26268 | -0.332611298 | -0.165664773 | -5.019029131 | 4.72E-06 | 0.000157989 | 3.95851536 |
| IRF2BP1 | 26145 | -0.503896116 | -0.18666039 | -5.018640217 | 4.73E-06 | 0.000158081 | 3.95715461 |
| C9orf106 | 414318 | 0.479790584 | -0.130464186 | 5.017137721 | 4.76E-06 | 0.000158728 | 3.951898003 |
| ZBTB9 | 221504 | -0.527501201 | -0.075725245 | -5.016939459 | 4.76E-06 | 0.000158728 | 3.951204411 |
| SLC35E3 | 55508 | -0.865228537 | -0.299822449 | -5.016844969 | 4.76E-06 | 0.000158728 | 3.950873859 |
| LOC643444 | 643444 | 0.572370222 | -0.114791163 | 5.016562234 | 4.77E-06 | 0.000158759 | 3.949884778 |
| FBXL21 | 26223 | 0.485921587 | -0.092162707 | 5.015135562 | 4.79E-06 | 0.000159462 | 3.944894258 |
| NUMA1 | 4926 | -0.744147136 | -0.10089469 | -5.014493117 | 4.80E-06 | 0.000159696 | 3.942647163 |
| FBXL16 | 146330 | 0.500484351 | -0.009630583 | 5.014088779 | 4.81E-06 | 0.000159696 | 3.941232957 |
| HADH | 3033 | -1.195800915 | -0.313894879 | -5.013759735 | 4.82E-06 | 0.000159696 | 3.940082134 |
| ATP5EP2 | 432369 | -0.898762135 | -0.208363438 | -5.01366245 | 4.82E-06 | 0.000159696 | 3.939741889 |
| LOC441155 | 441155 | 0.533660147 | -0.039152809 | 5.013595578 | 4.82E-06 | 0.000159696 | 3.93950801 |
| CEP70 | 80321 | -0.454493987 | -0.133642265 | -5.012783756 | 4.83E-06 | 0.00016004 | 3.936668847 |
| TTTY19 | 252952 | 0.381554544 | -0.087490653 | 5.011647688 | 4.85E-06 | 0.000160443 | 3.932696017 |
| FCGR3A | 2214 | 0.461548524 | -0.052837131 | 5.011141271 | 4.86E-06 | 0.000160608 | 3.930925192 |
| WDR13 | 64743 | -0.654791122 | -0.111709136 | -5.010741633 | 4.87E-06 | 0.00016071 | 3.929527801 |
| HSF5 | 124535 | 0.425030545 | 0.002815326 | 5.010120483 | 4.88E-06 | 0.000160858 | 3.927355949 |
| SLC22A3 | 6581 | -0.926525249 | -0.074516726 | -5.01003842 | 4.88E-06 | 0.000160858 | 3.927069023 |
| LIMCH1 | 22998 | -1.500541969 | -0.390865534 | -5.009655121 | 4.89E-06 | 0.000160951 | 3.925728881 |
| LOC391045 | 391045 | 0.841089231 | 0.084982558 | 5.008165214 | 4.92E-06 | 0.000161702 | 3.920520055 |
| USP32 | 84669 | 0.344372397 | -0.042062546 | 5.007718753 | 4.93E-06 | 0.000161834 | 3.918959314 |
| GSDMB | 55876 | 1.178320374 | 0.499404964 | 4.541753399 | 2.66E-05 | 0.000507921 | 2.323138766 |
| FAM62B | 57488 | -1.21822238 | -0.407996287 | -5.006892018 | 4.94E-06 | 0.000161923 | 3.916069359 |
| U2AF1L4 | 199746 | 0.707527212 | -0.011771854 | 5.005417334 | 4.97E-06 | 0.00016267 | 3.910914891 |
| NEUROG1 | 4762 | 0.524260964 | -0.16505921 | 5.004803453 | 4.98E-06 | 0.000162903 | 3.90876937 |
| ATP5J | 522 | -0.917566108 | -0.299998867 | -5.003569851 | 5.00E-06 | 0.000163422 | 3.904458239 |
| LOC728654 | 728654 | 0.539898122 | -0.113752659 | 5.003402811 | 5.00E-06 | 0.000163422 | 3.903874508 |
| DUOX1 | 53905 | 0.582378456 | -0.122238842 | 4.596835945 | 2.19E-05 | 0.000446977 | 2.508087251 |
| PRR13 | 54458 | 0.387271727 | -0.054859622 | 5.003039378 | 5.01E-06 | 0.000163422 | 3.9026045 |
| METTL2A | 339175 | -0.54641555 | -0.217493403 | -5.002080526 | 5.03E-06 | 0.000163845 | 3.899253988 |
| PGRMC2 | 10424 | -0.94409883 | -0.067347566 | -5.001889687 | 5.03E-06 | 0.000163845 | 3.89858717 |
| USP9X | 8239 | -0.393767318 | -0.109108334 | -5.001089119 | 5.05E-06 | 0.000164193 | 3.895789993 |
| FIBIN | 387758 | -0.79693867 | 0.021650613 | -5.000336479 | 5.06E-06 | 0.000164378 | 3.89316044 |
| UNC119 | 9094 | 0.380929933 | -0.106009364 | 4.998183322 | 5.10E-06 | 0.00016555 | 3.885638673 |
| ATRN | 8455 | 0.426239289 | -0.021927278 | 4.996763873 | 5.13E-06 | 0.000166281 | 3.880680735 |
| MGST3 | 4259 | -1.133924764 | -0.270031027 | -4.996060733 | 5.14E-06 | 0.000166575 | 3.878224973 |
| SEMA4A | 64218 | 1.105076097 | 0.281670853 | 4.99462749 | 5.17E-06 | 0.000167319 | 3.873219715 |
| HRAS | 3265 | -0.511826374 | -0.099037938 | -4.993982814 | 5.18E-06 | 0.000167579 | 3.870968524 |
| MRPL12 | 6182 | -0.758972421 | -0.11414727 | -4.993711567 | 5.19E-06 | 0.000167609 | 3.870021375 |
| WDR36 | 134430 | -0.777468578 | -0.156910094 | -4.993449039 | 5.19E-06 | 0.000167627 | 3.869104689 |
| SERPINB13 | 5275 | 0.489849467 | -0.057766129 | 4.993237803 | 5.20E-06 | 0.000167627 | 3.868367114 |
| CLDN12 | 9069 | -0.758513782 | -0.122982737 | -4.992941447 | 5.20E-06 | 0.000167647 | 3.867332351 |
| PDZRN3 | 23024 | -0.631284009 | -0.052881283 | -4.992760659 | 5.20E-06 | 0.000167647 | 3.866701118 |
| LOC654252 | 654252 | 0.551495794 | -0.076925368 | 4.992276889 | 5.21E-06 | 0.000167809 | 3.865012054 |
| ZBTB17 | 7709 | -0.62941563 | -0.036599782 | -4.990566073 | 5.25E-06 | 0.000168594 | 3.859039333 |
| SLC25A12 | 8604 | -0.572281381 | -0.061198702 | -4.989456161 | 5.27E-06 | 0.000168899 | 3.855164903 |
| LARP6 | 55323 | -1.011646506 | -0.177478522 | -4.989411325 | 5.27E-06 | 0.000168899 | 3.855008398 |
| HS3ST3B1 | 9953 | 0.483627289 | 0.058874802 | 4.988510711 | 5.29E-06 | 0.000169185 | 3.851864844 |
| KLHDC10 | 23008 | 0.386975052 | -0.113129209 | 4.987686903 | 5.30E-06 | 0.000169561 | 3.848989577 |
| MRPS6 | 64968 | -0.925897645 | -0.196329443 | -4.987031058 | 5.32E-06 | 0.000169701 | 3.846700674 |
| PIGO | 84720 | 0.42588588 | -0.017908644 | 4.984475561 | 5.37E-06 | 0.000170884 | 3.837783142 |
| LHX5 | 64211 | 0.368640625 | -0.060400573 | 4.983657265 | 5.38E-06 | 0.000171122 | 3.834928051 |
| PCNP | 57092 | -1.01155092 | -0.266708955 | -4.982788535 | 5.40E-06 | 0.000171532 | 3.831897197 |
| ALDH3B1 | 221 | 0.514682887 | -0.041933596 | 4.982298563 | 5.41E-06 | 0.000171703 | 3.830187863 |
| LOC644097 | 644097 | 0.63694642 | -0.164304417 | 4.981331666 | 5.43E-06 | 0.000172176 | 3.826814911 |
| PCCA | 5095 | -0.647028145 | -0.100325789 | -4.98071658 | 5.44E-06 | 0.000172427 | 3.824669363 |
| IKZF4 | 64375 | -0.393991103 | -0.148051763 | -4.979057097 | 5.47E-06 | 0.000173038 | 3.818881282 |
| EXOSC2 | 23404 | -0.441316565 | -0.074528857 | -4.979034678 | 5.47E-06 | 0.000173038 | 3.818803092 |
| PHF20 | 51230 | -0.630479521 | -0.122575448 | -4.978950494 | 5.48E-06 | 0.000173038 | 3.818509491 |
| LOC642423 | 642423 | 0.482955794 | -0.011259109 | 4.978774369 | 5.48E-06 | 0.000173038 | 3.817895237 |
| GDA | 9615 | 0.555608595 | -0.026460972 | 4.9786643 | 5.48E-06 | 0.000173038 | 3.817511368 |
| FAM177A1 | 283635 | -0.378321922 | -0.156920499 | -4.978136011 | 5.49E-06 | 0.000173236 | 3.815668981 |
| C20orf100 | 84969 | -1.195436148 | -0.119897949 | -4.97649962 | 5.53E-06 | 0.000174143 | 3.809962637 |
| MRPL20 | 55052 | -1.469478797 | 0.030365296 | -4.973906056 | 5.58E-06 | 0.000175391 | 3.800920052 |
| SLC25A4 | 291 | -1.21551906 | -0.166305564 | -4.283088943 | 6.59E-05 | 0.000923008 | 1.469509644 |
| LOC651876 | 651876 | 0.57295098 | 0.027487332 | 4.972317292 | 5.61E-06 | 0.000176053 | 3.795381699 |
| SNURF | 8926 | -1.309806125 | -0.093113923 | -4.972230922 | 5.61E-06 | 0.000176053 | 3.795080638 |
| LOC647135 | 647135 | 0.403127889 | -0.02195029 | 4.971008455 | 5.64E-06 | 0.000176565 | 3.7908197 |
| NDUFB10 | 4716 | -1.090447225 | -0.21532914 | -4.969267067 | 5.67E-06 | 0.000177558 | 3.784750785 |
| RIMS3 | 9783 | -0.894826637 | -0.126531905 | -4.966238664 | 5.74E-06 | 0.00017926 | 3.774198561 |
| TCEB2 | 6923 | -0.762980592 | -0.118544605 | -4.965993017 | 5.74E-06 | 0.00017928 | 3.77334274 |
| WDR4 | 10785 | -0.84870328 | -0.266645231 | -4.965755679 | 5.75E-06 | 0.000179294 | 3.772515884 |
| AKAP10 | 11216 | 0.481468203 | -0.026216929 | 4.964412255 | 5.78E-06 | 0.000179918 | 3.76783586 |
| FBN2 | 2201 | 0.526523212 | -0.118827327 | 4.964379731 | 5.78E-06 | 0.000179918 | 3.767722564 |
| FLJ33534 | 285150 | 0.58419132 | -0.135889308 | 4.963628463 | 5.79E-06 | 0.000180273 | 3.765105638 |
| LOC643502 | 643502 | 0.417772343 | -0.119574341 | 4.963188529 | 5.80E-06 | 0.000180422 | 3.763573273 |
| TAGAP | 117289 | 0.7346115 | -0.004201237 | 4.962677415 | 5.81E-06 | 0.000180619 | 3.761793044 |
| LOC727934 | 727934 | 0.364528337 | -0.016884206 | 4.96214395 | 5.82E-06 | 0.000180831 | 3.759935046 |
| TMEM52 | 339456 | 0.628114431 | -0.10644297 | 4.960386869 | 5.86E-06 | 0.000181859 | 3.75381591 |
| FLJ44005 | 400797 | 0.647007232 | -0.175577074 | 4.958629718 | 5.90E-06 | 0.000182892 | 3.747697417 |
| DEGS1 | 8560 | -0.850435877 | -0.300518093 | -4.424738045 | 4.02E-05 | 0.000670788 | 1.933852106 |
| ACSS2 | 55902 | -0.364013984 | -0.152006416 | -4.95681228 | 5.94E-06 | 0.000183654 | 3.741369939 |
| OBFC2B | 79035 | -0.553939282 | -0.150937234 | -4.956615608 | 5.94E-06 | 0.000183654 | 3.740685274 |
| LOC51149 | 51149 | 0.430347025 | -0.080283863 | 4.956429656 | 5.95E-06 | 0.000183654 | 3.740037942 |
| MT1E | 4493 | -1.052878973 | -0.218644942 | -4.955244872 | 5.97E-06 | 0.00018431 | 3.735913713 |
| PHF14 | 9678 | 0.400853225 | -0.0146127 | 4.954864256 | 5.98E-06 | 0.000184423 | 3.734588877 |
| LOC440434 | 440434 | 0.456027406 | -0.053870159 | 4.952759643 | 6.03E-06 | 0.000185709 | 3.72726396 |
| ROCK2 | 9475 | 1.146141231 | 0.07327579 | 4.537052831 | 2.71E-05 | 0.000511999 | 2.30740536 |
| DTX2 | 113878 | 0.53264041 | -0.011712832 | 4.952192717 | 6.04E-06 | 0.000185805 | 3.725291043 |
| C20orf96 | 140680 | 0.478352632 | -0.008445312 | 4.951698112 | 6.05E-06 | 0.000185998 | 3.723569878 |
| KLK15 | 55554 | 0.429836072 | -0.081244581 | 4.949322662 | 6.11E-06 | 0.000187335 | 3.715304605 |
| LOC642009 | 642009 | 0.601492366 | -0.067847043 | 4.94760621 | 6.14E-06 | 0.000188225 | 3.709333298 |
| GFM2 | 84340 | 0.367154513 | -0.091236437 | 4.947040287 | 6.16E-06 | 0.00018847 | 3.707364713 |
| LOC390354 | 390354 | -1.4960244 | -0.177124157 | -4.945815976 | 6.18E-06 | 0.000189025 | 3.703106223 |
| LOC641751 | 641751 | 0.468754177 | -0.079784473 | 4.944199147 | 6.22E-06 | 0.000190002 | 3.697483111 |
| CX3CR1 | 1524 | 0.430377579 | -0.029160022 | 4.943552249 | 6.24E-06 | 0.000190307 | 3.695233498 |
| HMGCLL1 | 54511 | 0.563650145 | -0.022009473 | 4.943077446 | 6.25E-06 | 0.000190491 | 3.693582431 |
| TACSTD1 | 4072 | 0.362558104 | 0.02135706 | 4.942252651 | 6.27E-06 | 0.00019092 | 3.690714466 |
| KLK1 | 3816 | -0.71692495 | -0.146929604 | -4.941408304 | 6.29E-06 | 0.000191299 | 3.687778721 |
| SOCS7 | 30837 | 0.350098052 | -0.125137243 | 4.941182346 | 6.29E-06 | 0.000191299 | 3.686993114 |
| FLJ41733 | 400870 | 0.476766887 | -0.107976234 | 4.941081032 | 6.29E-06 | 0.000191299 | 3.68664087 |
| CDC42EP4 | 23580 | -0.894909223 | -0.080138621 | -4.939092146 | 6.34E-06 | 0.000192551 | 3.679726638 |
| LOC647359 | 647359 | 0.431444534 | -0.017341705 | 4.938412631 | 6.36E-06 | 0.000192883 | 3.677364611 |
| LOC641714 | 641714 | 0.567221891 | -0.076045629 | 4.93802847 | 6.36E-06 | 0.000193006 | 3.676029308 |
| LOC400163 | 400163 | 0.412591064 | -0.120229739 | 4.937224986 | 6.38E-06 | 0.000193427 | 3.673236624 |
| LOC648590 | 648590 | 0.511730824 | -0.068074723 | 4.936830078 | 6.39E-06 | 0.000193558 | 3.671864102 |
| PGPEP1 | 54858 | -0.307398864 | -0.09582385 | -4.934119308 | 6.46E-06 | 0.000195342 | 3.662443924 |
| TLR3 | 7098 | 0.363855567 | -0.063540342 | 4.932502925 | 6.49E-06 | 0.000196201 | 3.656827862 |
| IL2RA | 3559 | 0.468618559 | -0.045946237 | 4.932119539 | 6.50E-06 | 0.000196327 | 3.655495912 |
| CBX6 | 23466 | -0.91899485 | -0.147878553 | -4.930339649 | 6.55E-06 | 0.000197164 | 3.649312833 |
| RPS24 | 6229 | -1.129047444 | -0.287906355 | -4.930331877 | 6.55E-06 | 0.000197164 | 3.649285837 |
| ZNF569 | 148266 | 0.413841001 | 0.024499834 | 4.928035975 | 6.60E-06 | 0.000198678 | 3.641311579 |
| LOC642222 | 642222 | 0.397586277 | -0.034508039 | 4.92778886 | 6.61E-06 | 0.000198707 | 3.640453374 |
| GNAS | 2778 | 0.398184639 | 0.058944785 | 4.926215028 | 6.65E-06 | 0.000199475 | 3.634988057 |
| BRMS1 | 25855 | -0.641436994 | -0.122880775 | -4.925983256 | 6.65E-06 | 0.000199475 | 3.634183262 |
| RFXANK | 8625 | -0.642194224 | -0.133707963 | -4.925827075 | 6.66E-06 | 0.000199475 | 3.633640956 |
| LOC340069 | 340069 | 0.431552157 | -0.123416633 | 4.925644292 | 6.66E-06 | 0.000199475 | 3.633006288 |
| MS4A14 | 84689 | 0.567727924 | -0.108672921 | 4.925414374 | 6.67E-06 | 0.000199475 | 3.63220797 |
| LOC401525 | 401525 | 0.432529455 | -0.002569514 | 4.925385607 | 6.67E-06 | 0.000199475 | 3.632108087 |
| LOC388743 | 388743 | 0.463120184 | -0.028855654 | 4.925282719 | 6.67E-06 | 0.000199475 | 3.631750847 |
| GLG1 | 2734 | -0.778105205 | -0.090545621 | -4.924412936 | 6.69E-06 | 0.000199959 | 3.62873097 |
| LOC646750 | 646750 | 0.651757912 | -0.053373387 | 4.922915474 | 6.73E-06 | 0.000200907 | 3.623532319 |
| LOC645445 | 645445 | 0.477114959 | -0.029185869 | 4.922565444 | 6.74E-06 | 0.000201012 | 3.622317235 |
| PAK1IP1 | 55003 | -0.494774297 | -0.054946835 | -4.523657425 | 2.84E-05 | 0.000529273 | 2.262612417 |
| LOC220998 | 220998 | 0.48225179 | -0.117691068 | 4.921244239 | 6.77E-06 | 0.000201682 | 3.61773117 |
| DEPDC7 | 91614 | 0.35895889 | -0.015401372 | 4.920602941 | 6.78E-06 | 0.000202004 | 3.61550533 |
| MYO9A | 4649 | -0.822208584 | -0.154182899 | -4.919668185 | 6.81E-06 | 0.000202543 | 3.61226116 |
| SON | 6651 | 0.475798736 | -0.087154349 | 4.580359423 | 2.32E-05 | 0.00046556 | 2.45265339 |
| PML | 5371 | 0.535286809 | 0.065086939 | 4.91859494 | 6.83E-06 | 0.000203034 | 3.608536668 |
| HDAC7A | 51564 | 0.439561414 | 0.063535101 | 4.918074071 | 6.85E-06 | 0.00020323 | 3.60672921 |
| ALKBH5 | 54890 | -0.680886807 | -0.092123004 | -4.917921096 | 6.85E-06 | 0.00020323 | 3.60619839 |
| GSTM4 | 2948 | 0.420422764 | -0.032958815 | 4.917651278 | 6.86E-06 | 0.000203278 | 3.605262146 |
| MAGEA3 | 4102 | 0.493992582 | -0.01176679 | 4.917373224 | 6.86E-06 | 0.000203332 | 3.604297347 |
| KCNJ14 | 3770 | 0.331523724 | -0.060335784 | 4.916876782 | 6.88E-06 | 0.000203549 | 3.60257483 |
| TEX2 | 55852 | -0.92260889 | -0.152272498 | -4.91592662 | 6.90E-06 | 0.000204105 | 3.599278235 |
| CHST10 | 9486 | -0.365669555 | -0.092002128 | -4.915441306 | 6.91E-06 | 0.000204161 | 3.597594539 |
| KHDRBS3 | 10656 | -0.997630137 | -0.166965174 | -4.914923176 | 6.93E-06 | 0.000204396 | 3.595797071 |
| ZNHIT1 | 10467 | -0.547647718 | -0.061968937 | -4.910757706 | 7.03E-06 | 0.000207365 | 3.581349315 |
| LRAT | 9227 | 0.445727618 | -0.075374019 | 4.91048876 | 7.04E-06 | 0.000207365 | 3.580416662 |
| TAF3 | 83860 | 0.574261176 | -0.148744926 | 4.909001074 | 7.08E-06 | 0.000208251 | 3.575258046 |
| LOC652222 | 652222 | 0.409473329 | -0.109588193 | 4.908393618 | 7.09E-06 | 0.000208505 | 3.573151853 |
| FOXD1 | 2297 | -0.713788671 | -0.177089075 | -4.908056551 | 7.10E-06 | 0.000208505 | 3.571983212 |
| PTPN22 | 26191 | 0.671937864 | -0.032960503 | 4.906505214 | 7.14E-06 | 0.000209398 | 3.566605011 |
| PEX16 | 9409 | -0.697120917 | -0.106242677 | -4.906279832 | 7.15E-06 | 0.000209398 | 3.565823715 |
| RPS17 | 6218 | -0.524706799 | -0.111281361 | -4.905875391 | 7.16E-06 | 0.000209398 | 3.564421735 |
| TSPAN19 | 144448 | 0.486452908 | -0.122144296 | 4.905760927 | 7.16E-06 | 0.000209398 | 3.56402496 |
| LOC652640 | 652640 | 0.386865841 | -0.120743249 | 4.905669483 | 7.16E-06 | 0.000209398 | 3.563707981 |
| FLJ40453 | 401217 | 0.603975256 | -0.06603692 | 4.90471535 | 7.19E-06 | 0.000209975 | 3.560400768 |
| TBC1D3E | 729877 | 0.387550703 | -0.066282003 | 4.904217007 | 7.20E-06 | 0.000210202 | 3.55867352 |
| EFCAB5 | 374786 | 0.517040037 | -0.153805682 | 4.903027571 | 7.23E-06 | 0.000210962 | 3.554551253 |
| MRPL54 | 116541 | -0.644322573 | -0.116733552 | -4.90189209 | 7.26E-06 | 0.000211684 | 3.550616374 |
| C14orf169 | 79697 | -0.604163866 | -0.082888241 | -4.901430296 | 7.28E-06 | 0.000211885 | 3.549016186 |
| CHSY3 | 337876 | -1.017139233 | -0.285354628 | -4.901116973 | 7.29E-06 | 0.000211971 | 3.547930514 |
| LOC652571 | 652571 | 0.494875279 | -0.025093634 | 4.89974016 | 7.32E-06 | 0.000212884 | 3.543160154 |
| ARG2 | 384 | -0.379001231 | -0.172355862 | -4.89625698 | 7.42E-06 | 0.000215453 | 3.531094198 |
| MRPL18 | 29074 | -0.876049559 | -0.110822103 | -4.894750046 | 7.46E-06 | 0.000216484 | 3.525875203 |
| OR5D14 | 219436 | 0.540155762 | -0.062935763 | 4.89394712 | 7.48E-06 | 0.00021696 | 3.52309469 |
| MT1X | 4501 | -1.862236777 | -0.514895499 | -4.892920292 | 7.51E-06 | 0.000217616 | 3.519539088 |
| AMY2A | 279 | 0.400601658 | -0.112821319 | 4.891869242 | 7.54E-06 | 0.000218089 | 3.515899939 |
| C11orf17 | 56672 | -0.263415478 | -0.101762443 | -4.891782003 | 7.54E-06 | 0.000218089 | 3.515597897 |
| GALNTL4 | 374378 | -0.772790619 | -0.084075452 | -4.891597187 | 7.54E-06 | 0.000218089 | 3.51495803 |
| LGR6 | 59352 | -0.733402494 | -0.137886007 | -4.891364982 | 7.55E-06 | 0.000218089 | 3.514154108 |
| UCP2 | 7351 | 1.454127085 | 0.324258679 | 4.891322002 | 7.55E-06 | 0.000218089 | 3.514005308 |
| NUDC | 10726 | -0.609935524 | -0.127641261 | -4.890544534 | 7.57E-06 | 0.000218549 | 3.511313747 |
| QTRTD1 | 79691 | 0.44814705 | -0.065285113 | 4.890053037 | 7.59E-06 | 0.000218782 | 3.509612295 |
| TRIM69 | 140691 | 0.41182092 | -0.08931311 | 4.889718753 | 7.59E-06 | 0.000218889 | 3.508455124 |
| AVP | 551 | 0.763476799 | -0.062580679 | 4.889100294 | 7.61E-06 | 0.000219224 | 3.506314327 |
| POU5F1 | 5460 | 0.384372558 | -0.085253384 | 4.887148543 | 7.67E-06 | 0.000220471 | 3.499559081 |
| SNX15 | 29907 | -0.329935374 | -0.130920294 | -4.886184859 | 7.69E-06 | 0.000220765 | 3.496224075 |
| EIF3I | 8668 | -0.711547077 | -0.156836423 | -4.885648262 | 7.71E-06 | 0.000220915 | 3.494367205 |
| AP3S2 | 10239 | -0.371978759 | -0.096533167 | -4.885568354 | 7.71E-06 | 0.000220915 | 3.494090694 |
| HPX | 3263 | 0.440792893 | -0.070842713 | 4.885401467 | 7.72E-06 | 0.000220915 | 3.493513208 |
| SNTB2 | 6645 | -0.561999593 | -0.141498333 | -4.884979481 | 7.73E-06 | 0.000221095 | 3.492053036 |
| LYSMD1 | 388695 | -0.371796604 | -0.076449153 | -4.884177684 | 7.75E-06 | 0.000221583 | 3.48927877 |
| FTSJ1 | 24140 | 0.431693152 | 0.060226312 | 4.88258763 | 7.80E-06 | 0.000222551 | 3.483777659 |
| LAT1-3TM | 81893 | 0.618630501 | 0.052048529 | 4.88224288 | 7.81E-06 | 0.00022267 | 3.482585025 |
| SLC25A29 | 123096 | -0.510098318 | -0.132115724 | -4.880986472 | 7.84E-06 | 0.000223532 | 3.478238896 |
| DCAKD | 79877 | -0.740593973 | -0.251324329 | -4.880131627 | 7.87E-06 | 0.000223907 | 3.475282113 |
| SYF2 | 25949 | -0.818708825 | -0.134011723 | -4.879880552 | 7.87E-06 | 0.000223951 | 3.474413722 |
| TBX19 | 9095 | 0.500479803 | 0.084913952 | 4.878225446 | 7.92E-06 | 0.000225012 | 3.468689701 |
| ZNF271 | 10778 | -0.670211423 | -0.079095902 | -4.878190012 | 7.92E-06 | 0.000225012 | 3.468567163 |
| DBNDD2 | 55861 | 0.677558753 | -0.117550433 | 4.87748037 | 7.94E-06 | 0.000225433 | 3.466113196 |
| LOC440776 | 440776 | 0.73533917 | 0.161254966 | 4.875546258 | 8.00E-06 | 0.000226867 | 3.459425733 |
| KDELR1 | 10945 | -0.700455662 | -0.107257942 | -4.872457552 | 8.09E-06 | 0.000229274 | 3.448748436 |
| C21orf57 | 54059 | -0.637523258 | -0.111369137 | -4.871460666 | 8.12E-06 | 0.000229944 | 3.44530293 |
| TLE2 | 7089 | -0.737314512 | -0.180186391 | -4.870464148 | 8.15E-06 | 0.000230285 | 3.441858994 |
| DEAF1 | 10522 | 0.643470393 | -0.192824448 | 4.870247642 | 8.15E-06 | 0.000230302 | 3.441110798 |
| OR1J1 | 347168 | 0.401233618 | -0.080554448 | 4.868735236 | 8.20E-06 | 0.000230831 | 3.43588464 |
| AUTS2 | 26053 | -0.952229915 | -0.191523267 | -4.868684076 | 8.20E-06 | 0.000230831 | 3.43570787 |
| ZFHX3 | 463 | -0.872792625 | -0.060501777 | -4.868464843 | 8.21E-06 | 0.000230831 | 3.434950365 |
| LOC654174 | 654174 | 0.516305267 | -0.034948259 | 4.868443496 | 8.21E-06 | 0.000230831 | 3.434876606 |
| KNDC1 | 85442 | 0.558474166 | 0.066146063 | 4.867502673 | 8.24E-06 | 0.000231337 | 3.431626001 |
| LOC651427 | 651427 | 0.608083204 | -0.153255553 | 4.867452428 | 8.24E-06 | 0.000231337 | 3.431452407 |
| MFSD11 | 79157 | -0.892551944 | -0.16608376 | -4.866705031 | 8.26E-06 | 0.000231803 | 3.428870303 |
| RIOK1 | 83732 | 0.443467342 | -0.110896354 | 4.864853156 | 8.32E-06 | 0.000232945 | 3.422473182 |
| LAMC2 | 3918 | 0.431372033 | -0.084821612 | 4.864772769 | 8.32E-06 | 0.000232945 | 3.422195515 |
| ADCY9 | 115 | -0.701424044 | -0.128219779 | -4.862929684 | 8.38E-06 | 0.000234183 | 3.415829833 |
| CHML | 1122 | 0.356685493 | 0.02665197 | 4.862222765 | 8.40E-06 | 0.000234621 | 3.413388534 |
| ALDH1L1 | 10840 | -0.890033124 | -0.284458743 | -4.861686899 | 8.41E-06 | 0.000234913 | 3.411538059 |
| TTTY4C | 474150 | 0.44272699 | -0.142505375 | 4.860665876 | 8.44E-06 | 0.000235623 | 3.408012455 |
| ATOH8 | 84913 | -1.04168199 | -0.036072955 | -4.859835324 | 8.47E-06 | 0.000236003 | 3.405144784 |
| PCDH15 | 65217 | 0.573572649 | -0.137149682 | 4.858712032 | 8.50E-06 | 0.000236804 | 3.401266696 |
| COPG2 | 26958 | -0.59105314 | -0.190624652 | -4.858469887 | 8.51E-06 | 0.000236831 | 3.400430754 |
| ARMCX3 | 51566 | 0.464810421 | -0.015918708 | 4.857992776 | 8.53E-06 | 0.000236831 | 3.398783714 |
| ZNF32 | 7580 | -0.695753494 | -0.094750283 | -4.857917425 | 8.53E-06 | 0.000236831 | 3.398523599 |
| KLHDC2 | 23588 | -1.135246788 | -0.20853522 | -4.857907086 | 8.53E-06 | 0.000236831 | 3.398487907 |
| MRPS28 | 28957 | -0.770861635 | -0.231414086 | -4.857145942 | 8.55E-06 | 0.000237322 | 3.395860513 |
| BFAR | 51283 | -0.786145838 | -0.112149971 | -4.856522929 | 8.57E-06 | 0.000237694 | 3.393710065 |
| LOC649641 | 649641 | 0.436593847 | 0.007414841 | 4.856057899 | 8.59E-06 | 0.000237865 | 3.392104999 |
| SRRM1L | 401475 | 0.578307954 | -0.043623493 | 4.855897733 | 8.59E-06 | 0.000237865 | 3.3915522 |
| MADCAM1 | 8174 | 0.386880799 | 0.009169663 | 4.855746571 | 8.60E-06 | 0.000237865 | 3.391030481 |
| STARD8 | 9754 | -0.713780337 | -0.160456438 | -4.855541953 | 8.60E-06 | 0.000237876 | 3.390324276 |
| GPR171 | 29909 | 0.380792844 | -0.015622126 | 4.854759372 | 8.63E-06 | 0.000238221 | 3.387623447 |
| TMEM150C | 441027 | 0.419677882 | -0.060249649 | 4.8542921 | 8.64E-06 | 0.00023846 | 3.386010896 |
| C14orf121 | 90668 | 0.373371405 | 0.025192141 | 4.851849073 | 8.72E-06 | 0.000240424 | 3.377581114 |
| FGL1 | 2267 | 0.406946154 | -0.151976552 | 4.851537632 | 8.73E-06 | 0.000240529 | 3.376506602 |
| PSMG3 | 84262 | -0.507585123 | -0.145430504 | -4.85111756 | 8.74E-06 | 0.000240729 | 3.375057348 |
| DCTN2 | 10540 | -0.710335957 | -0.12797794 | -4.849814782 | 8.79E-06 | 0.00024149 | 3.370563082 |
| MXD4 | 10608 | -0.843680784 | -0.14851323 | -4.84975065 | 8.79E-06 | 0.00024149 | 3.370341859 |
| NQO1 | 1728 | -1.00422984 | -0.166301411 | -4.849676465 | 8.79E-06 | 0.00024149 | 3.370085953 |
| MMP10 | 4319 | 0.465980561 | -0.020766322 | 4.84907793 | 8.81E-06 | 0.000241848 | 3.368021354 |
| MRPL53 | 116540 | -0.641481801 | -0.115865188 | -4.84775991 | 8.85E-06 | 0.000242843 | 3.363475331 |
| TRPC1 | 7220 | -0.93907058 | -0.199759693 | -4.847561629 | 8.86E-06 | 0.000242849 | 3.362791479 |
| SIM1 | 6492 | 0.539301834 | -0.132256367 | 4.847311283 | 8.87E-06 | 0.000242902 | 3.361928079 |
| NKTR | 4820 | 0.966832942 | 0.144520442 | 4.84524138 | 8.93E-06 | 0.00024457 | 3.354790069 |
| C16orf30 | 79652 | -0.903022357 | -0.211697742 | -4.844558801 | 8.95E-06 | 0.000245008 | 3.352436499 |
| EIF4G1 | 1981 | 0.400335369 | -0.015501589 | 4.844361166 | 8.96E-06 | 0.000245015 | 3.351755068 |
| LOC389517 | 389517 | 0.812310192 | -0.038945664 | 4.843409893 | 8.99E-06 | 0.000245625 | 3.348475319 |
| AHCYL1 | 10768 | -1.124168663 | -0.114734433 | -4.842304799 | 9.03E-06 | 0.000246173 | 3.344665586 |
| HRCT1 | 646962 | -1.158050177 | 0.059954027 | -4.841894087 | 9.04E-06 | 0.000246371 | 3.34324978 |
| GPR17 | 2840 | 0.67385408 | -0.094934659 | 4.841549861 | 9.05E-06 | 0.000246452 | 3.342063204 |
| NOTCH2NL | 388677 | 0.401455309 | -0.10795726 | 4.841312682 | 9.06E-06 | 0.000246452 | 3.341245647 |
| PRDX1 | 5052 | -0.81307935 | -0.219029922 | -4.386430546 | 4.60E-05 | 0.000730229 | 1.807512991 |
| TTLL4 | 9654 | 0.344285776 | -0.051728661 | 4.840554681 | 9.09E-06 | 0.000246602 | 3.338632937 |
| RPL27 | 6155 | -0.623920453 | -0.098591043 | -4.840546474 | 9.09E-06 | 0.000246602 | 3.33860465 |
| FLJ45300 | 399957 | 0.47438218 | -0.034225891 | 4.840499191 | 9.09E-06 | 0.000246602 | 3.338441679 |
| SEC14L1 | 6397 | 0.540382505 | -0.026015349 | 4.840294869 | 9.10E-06 | 0.000246616 | 3.337737448 |
| C10orf37 | 221017 | 0.475236439 | -0.092839905 | 4.838824685 | 9.14E-06 | 0.000247603 | 3.332670575 |
| LOC644623 | 644623 | 0.474032238 | -0.113925227 | 4.83865647 | 9.15E-06 | 0.000247603 | 3.332090876 |
| LOC646892 | 646892 | 0.421488995 | -0.092291723 | 4.838631678 | 9.15E-06 | 0.000247603 | 3.332005439 |
| PRKCZ | 5590 | 0.505484419 | -0.042453505 | 4.838320925 | 9.16E-06 | 0.000247713 | 3.330934556 |
| RPS9 | 6203 | -0.669362314 | -0.114492613 | -4.837939138 | 9.17E-06 | 0.000247887 | 3.329618925 |
| LOC643438 | 643438 | -0.36143278 | -0.18838481 | -4.837679602 | 9.18E-06 | 0.000247951 | 3.328724594 |
| PHIP | 55023 | -0.990077158 | -0.147529223 | -4.836890407 | 9.21E-06 | 0.000248494 | 3.326005248 |
| PADI2 | 11240 | 0.407335725 | -0.025820616 | 4.836385686 | 9.23E-06 | 0.000248781 | 3.324266219 |
| DKFZp686I15217 | 401232 | 0.318232196 | -0.031087473 | 4.835904012 | 9.24E-06 | 0.00024889 | 3.322606671 |
| LOC651404 | 651404 | 0.613355496 | -0.137264843 | 4.835889454 | 9.24E-06 | 0.00024889 | 3.322556517 |
| EPPB9 | 27077 | -0.422777937 | -0.159856264 | -4.835095013 | 9.27E-06 | 0.00024927 | 3.319819527 |
| LZTS2 | 84445 | -0.520736505 | -0.091188081 | -4.834336023 | 9.29E-06 | 0.000249699 | 3.317204857 |
| LOC643201 | 643201 | 0.551409035 | -0.098311489 | 4.834247761 | 9.30E-06 | 0.000249699 | 3.316900814 |
| C9orf123 | 90871 | -0.743781927 | -0.052581252 | -4.833835734 | 9.31E-06 | 0.000249805 | 3.315481492 |
| C1orf116 | 79098 | 0.44413985 | 0.02611868 | 4.833500181 | 9.32E-06 | 0.000249868 | 3.314325641 |
| LOC652566 | 652566 | 0.54070892 | -0.030999524 | 4.832093861 | 9.37E-06 | 0.000250979 | 3.309481792 |
| LOC643637 | 643637 | 0.580964734 | -0.096928637 | 4.831045282 | 9.41E-06 | 0.000251663 | 3.305870521 |
| SDHAP3 | 728609 | 0.41008333 | 0.003179965 | 4.830971995 | 9.41E-06 | 0.000251663 | 3.305618134 |
| SLC24A3 | 57419 | -1.070760082 | 0.064687484 | -4.830641133 | 9.42E-06 | 0.000251762 | 3.304478735 |
| BLID | 414899 | 0.459368276 | -0.091009597 | 4.830491816 | 9.43E-06 | 0.000251762 | 3.303964538 |
| RBM46 | 166863 | 0.623208086 | -0.090482379 | 4.827526598 | 9.53E-06 | 0.000253984 | 3.293754762 |
| RPL37A | 6168 | -0.954016549 | -0.229014765 | -4.827387125 | 9.53E-06 | 0.000253984 | 3.293274598 |
| TBC1D3C | 414060 | 0.52259666 | -0.045936654 | 4.82733138 | 9.53E-06 | 0.000253984 | 3.293082688 |
| RDH14 | 57665 | -0.825421734 | -0.240431558 | -4.826759222 | 9.55E-06 | 0.000254341 | 3.291112993 |
| LOC652593 | 652593 | 0.470268769 | -0.080635749 | 4.82649714 | 9.56E-06 | 0.000254411 | 3.290210788 |
| ZNF846 | 162993 | 0.460515409 | -0.090340279 | 4.826209402 | 9.57E-06 | 0.000254427 | 3.289220292 |
| CHD8 | 57680 | -0.726220759 | -0.089507802 | -4.82610935 | 9.58E-06 | 0.000254427 | 3.288875882 |
| HYPB | 29072 | 0.543888323 | -0.012636296 | 4.824604246 | 9.63E-06 | 0.000255651 | 3.283695236 |
| LOC647955 | 647955 | 0.457493612 | -0.075227485 | 4.824181414 | 9.64E-06 | 0.000255664 | 3.282239952 |
| LOC441046 | 441046 | -0.294182864 | -0.122621072 | -4.824034905 | 9.65E-06 | 0.000255664 | 3.281735717 |
| LOC648164 | 648164 | 0.657797183 | -0.055670442 | 4.823837794 | 9.66E-06 | 0.000255675 | 3.281057337 |
| PXMP2 | 5827 | -0.611213201 | -0.103807031 | -4.823241924 | 9.68E-06 | 0.000255987 | 3.279006655 |
| ELOVL6 | 79071 | -0.708367042 | -0.013056173 | -4.822980335 | 9.69E-06 | 0.000255987 | 3.278106435 |
| ID1 | 3397 | -1.121620375 | -0.090635307 | -4.820119753 | 9.79E-06 | 0.000258459 | 3.26826353 |
| CCNB1IP1 | 57820 | -0.589109423 | -0.115289995 | -4.819660366 | 9.80E-06 | 0.000258557 | 3.266683073 |
| MRPS15 | 64960 | -0.664437673 | -0.131517431 | -4.819646995 | 9.80E-06 | 0.000258557 | 3.266637072 |
| HIGD1A | 25994 | -1.140969327 | -0.229964414 | -4.818858697 | 9.83E-06 | 0.000259126 | 3.263925196 |
| ADAMTS6 | 11174 | 0.388542815 | -0.036301709 | 4.81859381 | 9.84E-06 | 0.000259202 | 3.263013986 |
| LYPLA1 | 10434 | -0.321242659 | -0.079332035 | -4.818280024 | 9.85E-06 | 0.000259301 | 3.261934589 |
| CAP2 | 10486 | -1.436074522 | -0.26980685 | -4.818121085 | 9.86E-06 | 0.000259301 | 3.261387866 |
| ALOX5 | 240 | 0.658567852 | -0.166088773 | 4.816369745 | 9.92E-06 | 0.000260782 | 3.255364056 |
| UQCRQ | 27089 | -0.896299122 | -0.254098517 | -4.815558501 | 9.95E-06 | 0.000261204 | 3.252574071 |
| FXR1 | 8087 | -0.845961414 | -0.171877848 | -4.813548348 | 1.00E-05 | 0.000262926 | 3.245661745 |
| LOC646279 | 646279 | 0.482387818 | 0.002659744 | 4.813382577 | 1.00E-05 | 0.000262926 | 3.245091762 |
| TAF6 | 6878 | 0.639113827 | -0.074560972 | 4.812216435 | 1.01E-05 | 0.000263867 | 3.241082378 |
| COMMD5 | 28991 | -0.881206475 | -0.244295477 | -4.811531391 | 1.01E-05 | 0.000264349 | 3.238727283 |
| LOC647471 | 647471 | 0.399475871 | -0.047628849 | 4.811245096 | 1.01E-05 | 0.000264448 | 3.237743079 |
| CCDC102A | 92922 | -0.949160055 | -0.218534877 | -4.809872288 | 1.02E-05 | 0.000265593 | 3.233024099 |
| RAB34 | 83871 | -0.79019813 | -0.15697656 | -4.809552026 | 1.02E-05 | 0.000265726 | 3.231923294 |
| TMEM174 | 134288 | 0.364764774 | -0.080700299 | 4.807659488 | 1.02E-05 | 0.000267381 | 3.225418911 |
| SDHAF1 | 644096 | -0.577766423 | -0.143720276 | -4.807005665 | 1.03E-05 | 0.000267839 | 3.223172074 |
| COX17 | 10063 | -1.438418711 | -0.556934279 | -4.805547501 | 1.03E-05 | 0.000269082 | 3.218161631 |
| TXNRD2 | 10587 | 0.549537954 | -0.006932288 | 4.803445804 | 1.04E-05 | 0.000270423 | 3.210941092 |
| CYTSA | 23384 | -0.804853074 | -0.199663754 | -4.803442135 | 1.04E-05 | 0.000270423 | 3.210928485 |
| LOC641825 | 641825 | 0.9129255 | 0.176857066 | 4.803268175 | 1.04E-05 | 0.000270423 | 3.210330894 |
| GDF9 | 2661 | 0.478200762 | -0.043209688 | 4.803079829 | 1.04E-05 | 0.00027043 | 3.209683895 |
| LOC650546 | 650546 | 0.36638881 | -0.118685021 | 4.802209493 | 1.04E-05 | 0.000271107 | 3.206694293 |
| CSF3R | 1441 | 0.796375434 | 0.053744675 | 4.801772162 | 1.05E-05 | 0.000271359 | 3.205192154 |
| LOC643817 | 643817 | 0.426083773 | -0.097796504 | 4.800315913 | 1.05E-05 | 0.000272385 | 3.200190666 |
| AASS | 10157 | -0.460968152 | -0.095717904 | -4.800201354 | 1.05E-05 | 0.000272385 | 3.199797244 |
| NUDT2 | 318 | -0.660956502 | -0.107368243 | -4.80018815 | 1.05E-05 | 0.000272385 | 3.199751899 |
| DLST | 1743 | -0.979695622 | -0.366616123 | -4.799798832 | 1.05E-05 | 0.000272413 | 3.198414915 |
| LRTOMT | 220074 | -0.372278588 | -0.110857824 | -4.797822522 | 1.06E-05 | 0.000274193 | 3.191628661 |
| FLJ12688 | 79613 | 0.45202886 | -0.013868104 | 4.797080562 | 1.06E-05 | 0.000274752 | 3.189081235 |
| LOC651524 | 651524 | 0.644933225 | -0.111668634 | 4.79631204 | 1.07E-05 | 0.000275339 | 3.186442796 |
| LOC441876 | 441876 | -0.596155714 | -0.113429679 | -4.795907478 | 1.07E-05 | 0.000275397 | 3.185053954 |
| LARGE | 9215 | -0.940143761 | -0.213589192 | -4.795893439 | 1.07E-05 | 0.000275397 | 3.18500576 |
| GFOD1 | 54438 | -0.866490145 | -0.167007543 | -4.795635817 | 1.07E-05 | 0.000275474 | 3.184121382 |
| FHL5 | 9457 | -1.161404856 | -0.194513022 | -4.794361614 | 1.07E-05 | 0.000276389 | 3.179747546 |
| PKD1L1 | 168507 | 0.309883159 | -0.068739362 | 4.793998863 | 1.08E-05 | 0.000276572 | 3.178502461 |
| Magmas | 51025 | -0.569866182 | -0.070223725 | -4.793625063 | 1.08E-05 | 0.000276745 | 3.17721949 |
| C12orf64 | 283310 | 0.463210742 | -0.035080746 | 4.793467033 | 1.08E-05 | 0.000276745 | 3.176677107 |
| TM9SF3 | 56889 | -0.814563211 | -0.128774335 | -4.791544878 | 1.09E-05 | 0.000278216 | 3.170080614 |
| RGS12 | 6002 | 0.383607867 | -0.080971156 | 4.760593491 | 1.21E-05 | 0.000294744 | 3.064021565 |
| RPL32 | 6161 | -0.391353541 | -0.123418731 | -4.790565614 | 1.09E-05 | 0.000278778 | 3.166720399 |
| ZMYND19 | 116225 | -0.497048416 | -0.138194574 | -4.790334198 | 1.09E-05 | 0.000278778 | 3.165926368 |
| RUNX1T1 | 862 | 0.395239119 | -0.032167533 | 4.790248934 | 1.09E-05 | 0.000278778 | 3.165633819 |
| GTF3A | 2971 | -0.772472002 | -0.128711524 | -4.790194524 | 1.09E-05 | 0.000278778 | 3.16544713 |
| SPINT2 | 10653 | -1.000365587 | -0.180282123 | -4.789185173 | 1.10E-05 | 0.000279577 | 3.16198411 |
| NFYB | 4801 | -0.42210062 | -0.157657654 | -4.789048336 | 1.10E-05 | 0.000279577 | 3.161514657 |
| C2orf49 | 79074 | -0.438245652 | -0.106591723 | -4.787437593 | 1.10E-05 | 0.000280746 | 3.15598903 |
| C21orf33 | 8209 | -0.877345475 | -0.141393508 | -4.787361871 | 1.10E-05 | 0.000280746 | 3.155729289 |
| SNHG3 | 8420 | 0.468693358 | -0.089854259 | 4.786669346 | 1.11E-05 | 0.000281154 | 3.153353862 |
| LOC642255 | 642255 | 0.35737797 | -0.079917454 | 4.786604519 | 1.11E-05 | 0.000281154 | 3.153131506 |
| MCM3 | 4172 | -0.836037996 | -0.16901082 | -4.785793853 | 1.11E-05 | 0.000281618 | 3.15035105 |
| SETMAR | 6419 | -0.707123171 | -0.071727654 | -4.785266307 | 1.11E-05 | 0.000281763 | 3.148541767 |
| LOC642093 | 642093 | 0.461870858 | 0.030092224 | 4.785119206 | 1.11E-05 | 0.000281763 | 3.148037281 |
| C6orf47 | 57827 | -0.470491565 | -0.137952432 | -4.784575763 | 1.11E-05 | 0.000281938 | 3.146173589 |
| C8orf40 | 114926 | -0.833614185 | -0.164523216 | -4.784535608 | 1.11E-05 | 0.000281938 | 3.146035885 |
| LOC286297 | 286297 | 0.489696287 | -0.061112039 | 4.784415723 | 1.11E-05 | 0.000281938 | 3.145624765 |
| OTP | 23440 | 0.505091351 | -0.142221251 | 4.783935278 | 1.12E-05 | 0.000282239 | 3.14397722 |
| KCNA10 | 3744 | 0.525894033 | -0.082380789 | 4.783457961 | 1.12E-05 | 0.000282239 | 3.142340477 |
| IARS2 | 55699 | -0.965967989 | -0.25717432 | -4.78343041 | 1.12E-05 | 0.000282239 | 3.142246005 |
| PAQR3 | 152559 | -0.543344176 | -0.081383038 | -4.783407212 | 1.12E-05 | 0.000282239 | 3.142166459 |
| FLJ46361 | 375940 | 0.504497005 | -0.058233799 | 4.783236737 | 1.12E-05 | 0.000282239 | 3.141581912 |
| HOXC8 | 3224 | -0.814160173 | -0.023240994 | -4.783044411 | 1.12E-05 | 0.000282255 | 3.14092245 |
| MRPS12 | 6183 | 0.417211991 | -0.069409615 | 4.266370698 | 6.98E-05 | 0.000958811 | 1.415224476 |
| TACC1 | 6867 | -0.918228504 | -0.23822179 | -4.782147637 | 1.12E-05 | 0.000282716 | 3.137847678 |
| FLJ25439 | 153657 | 0.439101268 | -0.096721578 | 4.782065039 | 1.12E-05 | 0.000282716 | 3.137564489 |
| PRNP | 5621 | -0.865758934 | -0.139398164 | -4.780722603 | 1.13E-05 | 0.000283914 | 3.132962183 |
| CALU | 813 | -1.052169192 | -0.223546929 | -4.779391435 | 1.13E-05 | 0.000284922 | 3.12839907 |
| GFOD2 | 81577 | -0.448029521 | -0.156825146 | -4.779062182 | 1.14E-05 | 0.000285081 | 3.12727051 |
| LOC652557 | 652557 | 0.345205504 | -0.058874602 | 4.778552587 | 1.14E-05 | 0.000285317 | 3.125523873 |
| TCEAL8 | 90843 | -1.275830957 | -0.426840909 | -4.778482684 | 1.14E-05 | 0.000285317 | 3.125284284 |
| BRP44L | 51660 | -0.715351772 | -0.138943207 | -4.778174564 | 1.14E-05 | 0.000285454 | 3.124228248 |
| PITX2 | 5308 | 0.462149151 | -0.030806376 | 4.777200229 | 1.14E-05 | 0.000286281 | 3.120889053 |
| PLAC9 | 219348 | -0.981582055 | -0.290003924 | -4.7768765 | 1.14E-05 | 0.000286355 | 3.119779651 |
| NUS1 | 116150 | -0.429139259 | -0.104396571 | -4.776723219 | 1.15E-05 | 0.000286355 | 3.119254378 |
| LOC93622 | 93622 | -0.510471242 | -0.029476819 | -4.776603095 | 1.15E-05 | 0.000286355 | 3.118842736 |
| KIAA1409 | 57578 | 0.422800433 | -0.024233093 | 4.776323088 | 1.15E-05 | 0.000286464 | 3.117883217 |
| LOC652656 | 652656 | 0.525858647 | -0.123084578 | 4.776021356 | 1.15E-05 | 0.000286509 | 3.11684928 |
| LOC650680 | 650680 | 0.491117218 | -0.092206163 | 4.775930199 | 1.15E-05 | 0.000286509 | 3.116536922 |
| EVPL | 2125 | 0.528386794 | -0.004934653 | 4.775403615 | 1.15E-05 | 0.000286875 | 3.114732573 |
| C20orf51 | 63930 | 0.578640096 | 0.09262012 | 4.775126664 | 1.15E-05 | 0.000286956 | 3.113783632 |
| FLJ37673 | 285456 | 0.525945097 | -0.102944275 | 4.77497594 | 1.15E-05 | 0.000286956 | 3.113267204 |
| SLC2A9 | 56606 | 0.37815977 | -0.053886918 | 4.774054574 | 1.16E-05 | 0.000287734 | 3.110110468 |
| MTMR3 | 8897 | 0.535729467 | 0.01393283 | 4.773637834 | 1.16E-05 | 0.000287986 | 3.108682744 |
| MTMR9 | 66036 | -0.7346583 | -0.217407733 | -4.773293912 | 1.16E-05 | 0.000288164 | 3.107504532 |
| SLC30A4 | 7782 | 0.486597398 | -0.074971944 | 4.772897347 | 1.16E-05 | 0.000288231 | 3.10614602 |
| EIF1AX | 1964 | -0.55138542 | -0.096269456 | -4.7727612 | 1.16E-05 | 0.000288231 | 3.105679632 |
| LOC644264 | 644264 | 0.544251887 | -0.101542787 | 4.772707607 | 1.16E-05 | 0.000288231 | 3.105496047 |
| PSMB6 | 5694 | -0.648296878 | -0.130634889 | -4.772279506 | 1.16E-05 | 0.000288497 | 3.104029584 |
| POLDIP2 | 26073 | -0.47617338 | -0.07214201 | -4.771191009 | 1.17E-05 | 0.00028936 | 3.100301191 |
| C11orf74 | 119710 | -0.819993618 | -0.190963256 | -4.770914432 | 1.17E-05 | 0.00028938 | 3.099353902 |
| LOXL1 | 4016 | -0.786476551 | -0.013300645 | -4.770557386 | 1.17E-05 | 0.000289382 | 3.098131037 |
| LOC440910 | 440910 | 0.462053098 | -0.081439231 | 4.770435232 | 1.17E-05 | 0.000289382 | 3.097712675 |
| CST11 | 140880 | 0.548628807 | -0.077570016 | 4.769143251 | 1.18E-05 | 0.000290149 | 3.093288097 |
| KRTAP4-8 | 83898 | 0.348973076 | -0.093660531 | 4.768557941 | 1.18E-05 | 0.000290295 | 3.09128379 |
| FAM119B | 25895 | 0.477226438 | -0.076069471 | 4.768439266 | 1.18E-05 | 0.000290295 | 3.090877419 |
| TIMM44 | 10469 | -0.49033929 | -0.082429245 | -4.768410801 | 1.18E-05 | 0.000290295 | 3.090779949 |
| PKLR | 5313 | 0.41580507 | -0.080344893 | 4.768314496 | 1.18E-05 | 0.000290295 | 3.09045018 |
| UBE2E2 | 7325 | -0.981133857 | -0.113857653 | -4.767270406 | 1.19E-05 | 0.000291127 | 3.086875204 |
| FAM123A | 219287 | 0.578075134 | -0.066525296 | 4.767144285 | 1.19E-05 | 0.000291127 | 3.086443386 |
| MITF | 4286 | 0.327166123 | -0.023626692 | 4.766834718 | 1.19E-05 | 0.000291127 | 3.085383505 |
| KPNA4 | 3840 | -0.925400535 | -0.270087014 | -4.766623237 | 1.19E-05 | 0.000291169 | 3.084659462 |
| KLK6 | 5653 | 0.459759273 | -0.013592541 | 4.76433722 | 1.20E-05 | 0.000293041 | 3.076833793 |
| LOC375295 | 375295 | 0.56899823 | -0.030729914 | 4.763659892 | 1.20E-05 | 0.000293495 | 3.074515429 |
| LEAP-2 | 116842 | 0.400048347 | -0.055904259 | 4.763417486 | 1.20E-05 | 0.000293495 | 3.073685758 |
| DLG5 | 9231 | -0.395939824 | -0.132529304 | -4.76317694 | 1.20E-05 | 0.000293545 | 3.072862467 |
| BRMS1L | 84312 | -0.595086394 | -0.014895678 | -4.762419688 | 1.21E-05 | 0.000294168 | 3.07027083 |
| C7orf55 | 154791 | -0.535726833 | -0.068450214 | -4.762159406 | 1.21E-05 | 0.000294231 | 3.069380079 |
| TBC1D28 | 254272 | 0.375662588 | -0.051591218 | 4.762019065 | 1.21E-05 | 0.000294231 | 3.068899803 |
| C21orf70 | 85395 | -0.49341782 | -0.117516564 | -4.761408996 | 1.21E-05 | 0.000294403 | 3.066812102 |
| CTRB2 | 440387 | 0.407434307 | -0.059092361 | 4.761345508 | 1.21E-05 | 0.000294403 | 3.066594845 |
| LOC646154 | 646154 | 0.378536208 | -0.080984685 | 4.760866659 | 1.21E-05 | 0.000294732 | 3.064956281 |
| ALDH4A1 | 8659 | -0.580302072 | -0.140441211 | -4.760243385 | 1.22E-05 | 0.000294852 | 3.062823618 |
| HNRNPAB | 3182 | -0.759744561 | -0.2489351 | -4.75991776 | 1.22E-05 | 0.000295018 | 3.061709477 |
| LOC343705 | 343705 | 0.424909165 | -0.055573163 | 4.759439302 | 1.22E-05 | 0.000295347 | 3.060072465 |
| ZMPSTE24 | 10269 | -0.966329648 | -0.175616217 | -4.758567698 | 1.22E-05 | 0.00029608 | 3.057090523 |
| CCDC12 | 151903 | -0.723111807 | -0.15751055 | -4.758134228 | 1.23E-05 | 0.00029608 | 3.05560762 |
| TBC1D3G | 654341 | 0.67634314 | 0.049747042 | 4.758106651 | 1.23E-05 | 0.00029608 | 3.055513283 |
| LOC648003 | 648003 | 0.512082546 | -0.093676325 | 4.757651681 | 1.23E-05 | 0.00029608 | 3.053956897 |
| MT1A | 4489 | -1.000367048 | -0.120606449 | -4.757607966 | 1.23E-05 | 0.00029608 | 3.053807356 |
| C14orf104 | 55172 | -0.490578222 | -0.083118912 | -4.757607813 | 1.23E-05 | 0.00029608 | 3.053806835 |
| ZNF223 | 7766 | 1.206899942 | 0.009540411 | 4.757567637 | 1.23E-05 | 0.00029608 | 3.053669401 |
| MKKS | 8195 | -0.341076794 | -0.133800342 | -4.410373132 | 4.23E-05 | 0.000695194 | 1.886411253 |
| ATP1A2 | 477 | -1.220357086 | 0.069349719 | -4.755389553 | 1.24E-05 | 0.000298056 | 3.046219471 |
| NEXN | 91624 | -1.377044058 | -0.216593821 | -4.753306993 | 1.25E-05 | 0.000299394 | 3.039097691 |
| LOC643696 | 643696 | 0.528562977 | -0.010640671 | 4.752925162 | 1.25E-05 | 0.000299625 | 3.037792088 |
| LOC650029 | 650029 | 0.441040987 | -0.077560706 | 4.752357858 | 1.25E-05 | 0.000300057 | 3.035852373 |
| C13orf1 | 57213 | -0.419293834 | -0.109801672 | -4.751414514 | 1.26E-05 | 0.000300727 | 3.032627144 |
| NF2 | 4771 | 0.523298181 | 0.024793971 | 4.75140437 | 1.26E-05 | 0.000300727 | 3.032592464 |
| KLHL30 | 377007 | -0.641963071 | -0.170963224 | -4.750899495 | 1.26E-05 | 0.000301093 | 3.030866446 |
| LOC653394 | 653394 | 0.444042628 | -0.039670373 | 4.75046894 | 1.26E-05 | 0.000301379 | 3.029394572 |
| PTH | 5741 | 0.646512984 | -0.172796759 | 4.749717057 | 1.26E-05 | 0.000302016 | 3.026824365 |
| KIAA1328 | 57536 | -0.403865279 | -0.114374463 | -4.749451246 | 1.26E-05 | 0.000302123 | 3.025915773 |
| NUFIP1 | 26747 | -0.317102371 | -0.071135575 | -4.749205579 | 1.27E-05 | 0.000302208 | 3.025076053 |
| SMARCA2 | 6595 | 0.480988212 | -0.02420752 | 4.748923434 | 1.27E-05 | 0.000302333 | 3.024111674 |
| ITIH5 | 80760 | 1.297726626 | 0.102335637 | 4.747807974 | 1.27E-05 | 0.000303294 | 3.020299249 |
| PLGLB1 | 5343 | 0.454316413 | -0.060649547 | 4.747587405 | 1.27E-05 | 0.000303294 | 3.019545435 |
| LOC642245 | 642245 | 0.503945231 | -0.12225562 | 4.747392608 | 1.27E-05 | 0.000303294 | 3.01887971 |
| LOC730686 | 730686 | 0.5149694 | -0.141055356 | 4.747245951 | 1.27E-05 | 0.000303294 | 3.018378516 |
| MRPS5 | 64969 | -0.623926369 | -0.109303877 | -4.747208465 | 1.27E-05 | 0.000303294 | 3.018250411 |
| SNORA10 | 574042 | 0.632764853 | -0.009266871 | 4.746390915 | 1.28E-05 | 0.000304008 | 3.015456595 |
| ING3 | 54556 | -0.808844507 | -0.337212037 | -4.746089545 | 1.28E-05 | 0.000304054 | 3.014426776 |
| C14orf133 | 63894 | 0.528744392 | 0.025176121 | 4.74597127 | 1.28E-05 | 0.000304054 | 3.014022622 |
| LOC644961 | 644961 | 0.475126266 | -0.099145 | 4.743392116 | 1.29E-05 | 0.000306579 | 3.005210632 |
| SMUG1 | 23583 | -0.76664162 | -0.163768045 | -4.742376221 | 1.30E-05 | 0.000307521 | 3.001740289 |
| TOPORS | 10210 | -0.263263826 | -0.085502098 | -4.741746709 | 1.30E-05 | 0.000307809 | 2.999590016 |
| PPP2R2A | 5520 | -0.603659967 | -0.113280205 | -4.741617812 | 1.30E-05 | 0.000307809 | 2.99914975 |
| ISY1 | 57461 | -0.719595098 | -0.203578397 | -4.741197632 | 1.30E-05 | 0.000308091 | 2.997714596 |
| MRPS22 | 56945 | -0.650300382 | -0.111937875 | -4.739478568 | 1.31E-05 | 0.000309601 | 2.991843612 |
| LOC645460 | 645460 | 0.454828661 | -0.00354731 | 4.739412497 | 1.31E-05 | 0.000309601 | 2.991617983 |
| FBXO18 | 84893 | -0.8055262 | -0.154155306 | -4.739261025 | 1.31E-05 | 0.000309601 | 2.99110072 |
| DHX30 | 22907 | -0.478367836 | -0.063294942 | -4.738896415 | 1.31E-05 | 0.000309601 | 2.989855647 |
| COPS7A | 50813 | -0.6946551 | -0.128704261 | -4.738852142 | 1.31E-05 | 0.000309601 | 2.989704465 |
| SHC2 | 25759 | 0.458351629 | 0.046333047 | 4.738848807 | 1.31E-05 | 0.000309601 | 2.989693076 |
| LOC647096 | 647096 | 0.529427815 | -0.098594556 | 4.73820042 | 1.32E-05 | 0.000310141 | 2.987479067 |
| ALAS2 | 212 | 1.526798178 | 0.205956249 | 4.736535375 | 1.32E-05 | 0.000311825 | 2.981794166 |
| TIGD6 | 81789 | 0.348872761 | -0.062274638 | 4.735874998 | 1.33E-05 | 0.000312196 | 2.979539712 |
| LOC648984 | 648984 | 0.689922247 | -0.059462 | 4.735479954 | 1.33E-05 | 0.000312455 | 2.978191145 |
| LOC652438 | 652438 | 0.564682773 | -0.005627349 | 4.734947555 | 1.33E-05 | 0.00031287 | 2.976373763 |
| RNF220 | 55182 | -0.755230441 | -0.234120074 | -4.734640557 | 1.33E-05 | 0.00031303 | 2.975325847 |
| CCDC56 | 28958 | -0.776677913 | -0.150994627 | -4.732979613 | 1.34E-05 | 0.000314537 | 2.969656855 |
| LOC645317 | 645317 | -1.433280425 | 0.129069001 | -4.732346897 | 1.34E-05 | 0.000315069 | 2.967497559 |
| GRAP | 10750 | 0.450235239 | -0.025433251 | 4.729665443 | 1.36E-05 | 0.000317943 | 2.958347884 |
| MSL2 | 55167 | 0.425209459 | -0.039200656 | 4.729254922 | 1.36E-05 | 0.000318225 | 2.956947306 |
| LOC641806 | 641806 | 0.350892993 | -0.012912047 | 4.728213262 | 1.37E-05 | 0.000319044 | 2.953393715 |
| SCRIB | 23513 | -0.670770864 | -0.101480098 | -4.727206912 | 1.37E-05 | 0.000319825 | 2.949960915 |
| LPCAT3 | 10162 | -0.665131349 | -0.092448945 | -4.726618066 | 1.37E-05 | 0.000320316 | 2.947952436 |
| CABC1 | 56997 | -0.813817191 | -0.146990509 | -4.723528506 | 1.39E-05 | 0.000323476 | 2.937416165 |
| MMP20 | 9313 | 0.525372278 | -0.10809534 | 4.723403825 | 1.39E-05 | 0.000323476 | 2.936991033 |
| KIAA1549 | 57670 | 0.42780659 | -0.073182534 | 4.723087683 | 1.39E-05 | 0.000323653 | 2.935913086 |
| RNASEH1 | 246243 | -0.957825749 | -0.204174398 | -4.722511835 | 1.39E-05 | 0.000324135 | 2.933949706 |
| SERINC4 | 619189 | 0.40107712 | -0.091739385 | 4.722161754 | 1.40E-05 | 0.000324353 | 2.932756144 |
| WIF1 | 11197 | 0.533333034 | -0.090466624 | 4.721742416 | 1.40E-05 | 0.000324555 | 2.931326508 |
| ANGPTL3 | 27329 | 0.608902649 | -0.073489005 | 4.721605031 | 1.40E-05 | 0.000324555 | 2.930858141 |
| LCN12 | 286256 | 0.450448883 | -0.108027941 | 4.721369631 | 1.40E-05 | 0.000324555 | 2.930055634 |
| THYN1 | 29087 | -0.731240066 | -0.181815053 | -4.721335181 | 1.40E-05 | 0.000324555 | 2.92993819 |
| RAB24 | 53917 | 0.373012382 | 0.107280057 | 4.720846084 | 1.40E-05 | 0.000324937 | 2.928270866 |
| C14orf179 | 112752 | -0.582533928 | -0.070958697 | -4.720087439 | 1.41E-05 | 0.000325635 | 2.92568481 |
| LRFN3 | 79414 | -0.599719851 | -0.127884096 | -4.719418399 | 1.41E-05 | 0.000326038 | 2.923404352 |
| PTP4A2 | 8073 | 1.006962191 | 0.043784598 | 4.718958744 | 1.41E-05 | 0.000326333 | 2.92183768 |
| PPP1R3C | 5507 | -1.81067616 | -0.312335089 | -4.718618156 | 1.41E-05 | 0.000326333 | 2.920676876 |
| PSMB4 | 5692 | -0.69563482 | -0.229102561 | -4.718579784 | 1.41E-05 | 0.000326333 | 2.920546095 |
| ZNF706 | 51123 | -0.69777284 | -0.117456726 | -4.71851711 | 1.41E-05 | 0.000326333 | 2.920332494 |
| ASB4 | 51666 | 0.418583006 | -0.086061892 | 4.718313077 | 1.41E-05 | 0.000326351 | 2.919637127 |
| ALX3 | 257 | 0.361700038 | -0.097930686 | 4.718176829 | 1.42E-05 | 0.000326351 | 2.919172784 |
| GCSH | 2653 | 0.474536623 | -0.087827181 | 4.718001353 | 1.42E-05 | 0.000326367 | 2.91857476 |
| PMEPA1 | 56937 | -0.97953582 | -0.134615589 | -4.717722754 | 1.42E-05 | 0.00032641 | 2.917625314 |
| CD8B | 926 | 0.447859868 | -0.052515446 | 4.716867397 | 1.42E-05 | 0.00032713 | 2.914710469 |
| TMEM101 | 84336 | -0.624885995 | -0.114265577 | -4.715917526 | 1.43E-05 | 0.00032806 | 2.911473827 |
| SNORA65 | 26783 | 0.376992474 | -0.052521093 | 4.715561968 | 1.43E-05 | 0.000328289 | 2.910262357 |
| UPF3B | 65109 | -0.684805547 | -0.064522357 | -4.714771609 | 1.43E-05 | 0.000328937 | 2.90756956 |
| LOC727848 | 727848 | 0.510852489 | -0.01274451 | 4.714691255 | 1.43E-05 | 0.000328937 | 2.907295798 |
| LOC399715 | 399715 | 0.404522347 | -0.114198848 | 4.713451892 | 1.44E-05 | 0.000330025 | 2.903073663 |
| ZFAND2A | 90637 | -0.854974782 | -0.054888855 | -4.71303691 | 1.44E-05 | 0.000330327 | 2.901660058 |
| SOCS3 | 9021 | 1.237546944 | 0.380731957 | 4.710854548 | 1.45E-05 | 0.00033274 | 2.894226929 |
| LOC648907 | 648907 | 0.518535618 | 0.038622109 | 4.709336708 | 1.46E-05 | 0.000334189 | 2.889058078 |
| BOP1 | 23246 | -0.695419762 | -0.12828184 | -4.709325794 | 1.46E-05 | 0.000334189 | 2.889020914 |
| ZNF174 | 7727 | -0.261371736 | -0.122977566 | -4.707990093 | 1.47E-05 | 0.000335606 | 2.884472951 |
| ZNHIT3 | 9326 | -0.991636525 | -0.167810526 | -4.707597599 | 1.47E-05 | 0.000335886 | 2.88313665 |
| C20orf123 | 128506 | 0.378589066 | -0.054707978 | 4.70654756 | 1.48E-05 | 0.000336963 | 2.879561889 |
| ICF45 | 54974 | 0.358589981 | 0.054672529 | 4.705853498 | 1.48E-05 | 0.000337611 | 2.877199219 |
| VENTXP7 | 391518 | 0.464417216 | -0.116465736 | 4.704696222 | 1.49E-05 | 0.000338824 | 2.873260063 |
| DDX10 | 1662 | -0.646030753 | -0.085671689 | -4.702584589 | 1.50E-05 | 0.000341212 | 2.866073583 |
| LIPL3 | 340654 | 0.484740238 | -0.01466101 | 4.702284515 | 1.50E-05 | 0.000341383 | 2.865052464 |
| RSRC1 | 51319 | -0.454510635 | -0.122779952 | -4.701039182 | 1.51E-05 | 0.000342719 | 2.860815057 |
| USP13 | 8975 | -0.580513712 | -0.132716956 | -4.699267878 | 1.52E-05 | 0.000344711 | 2.854788839 |
| SP8 | 221833 | 0.360139843 | 0.013830315 | 4.698383981 | 1.52E-05 | 0.00034561 | 2.851782088 |
| C3orf26 | 84319 | -0.482789534 | -0.075590472 | -4.69690911 | 1.53E-05 | 0.000347069 | 2.846765596 |
| MKRN2 | 23609 | -0.835846595 | -0.171110539 | -4.696588945 | 1.53E-05 | 0.000347069 | 2.845676711 |
| KCNQ4 | 9132 | 0.504559891 | 0.039589106 | 4.696512678 | 1.53E-05 | 0.000347069 | 2.84541733 |
| CD99L2 | 83692 | -0.673419999 | -0.149353063 | -4.695891575 | 1.53E-05 | 0.000347321 | 2.843305064 |
| EPHA1 | 2041 | 0.430582926 | -0.062500463 | 4.695109198 | 1.54E-05 | 0.000348099 | 2.840644507 |
| TMEM120A | 83862 | -0.803231214 | -0.197271974 | -4.694655035 | 1.54E-05 | 0.000348468 | 2.839100175 |
| HOXA9 | 3205 | -0.946710664 | -0.21094351 | -4.694242277 | 1.54E-05 | 0.000348786 | 2.837696692 |
| RBMX2 | 51634 | 0.300538803 | -0.038303294 | 4.693988917 | 1.54E-05 | 0.000348904 | 2.836835229 |
| POLR3A | 11128 | -0.800815225 | -0.206205082 | -4.693826936 | 1.55E-05 | 0.000348907 | 2.836284483 |
| MAPK1 | 5594 | -0.380182668 | -0.108430566 | -4.693322949 | 1.55E-05 | 0.00034934 | 2.834570942 |
| RHOXF2B | 727940 | 0.446070686 | -0.088011178 | 4.692897477 | 1.55E-05 | 0.000349675 | 2.833124416 |
| GNG12 | 55970 | -0.866062794 | -0.095998995 | -4.692675794 | 1.55E-05 | 0.000349754 | 2.83237076 |
| SPOP | 8405 | -0.323444204 | -0.121884255 | -4.692147871 | 1.55E-05 | 0.000350218 | 2.830576043 |
| CDCP1 | 64866 | 0.485232479 | -0.091272424 | 4.691955457 | 1.56E-05 | 0.000350261 | 2.829921936 |
| OSBPL1A | 114876 | -0.647522983 | -0.123965825 | -4.691784349 | 1.56E-05 | 0.000350276 | 2.829340273 |
| CHCHD3 | 54927 | -0.502726927 | -0.075664332 | -4.691607432 | 1.56E-05 | 0.000350299 | 2.82873887 |
| FLJ32011 | 148930 | 0.427960633 | -0.086920478 | 4.691336888 | 1.56E-05 | 0.00035044 | 2.827819218 |
| C8orf59 | 401466 | -0.954789777 | -0.15008769 | -4.690449746 | 1.56E-05 | 0.000350878 | 2.824803748 |
| STK16 | 8576 | -0.477238048 | -0.205010186 | -4.690377852 | 1.56E-05 | 0.000350878 | 2.824559384 |
| RSU1 | 6251 | -0.904977523 | -0.279167212 | -4.690262856 | 1.57E-05 | 0.000350878 | 2.824168525 |
| IL1B | 3553 | 1.483322516 | 0.478963021 | 4.690198486 | 1.57E-05 | 0.000350878 | 2.823949742 |
| ABCG5 | 64240 | 0.317993379 | -0.047086441 | 4.690198121 | 1.57E-05 | 0.000350878 | 2.8239485 |
| C1orf71 | 163882 | -1.227429278 | -0.166301007 | -4.690014124 | 1.57E-05 | 0.00035091 | 2.823323127 |
| CSHL1 | 1444 | 0.360358417 | -0.070432602 | 4.689501383 | 1.57E-05 | 0.000351358 | 2.821580472 |
| ZCCHC9 | 84240 | -0.806601899 | -0.203816452 | -4.689006896 | 1.57E-05 | 0.000351784 | 2.819899938 |
| FAM86B1 | 85002 | 0.424652609 | -0.083375037 | 4.6870369 | 1.58E-05 | 0.000354083 | 2.813205632 |
| ID3 | 3399 | -0.837898794 | -0.238073943 | -4.686774217 | 1.58E-05 | 0.000354178 | 2.812313097 |
| DAK | 26007 | 0.391232367 | -0.001178004 | 4.686646746 | 1.59E-05 | 0.000354178 | 2.81187999 |
| ZYG11B | 79699 | -1.000229418 | -0.289853986 | -4.686132162 | 1.59E-05 | 0.000354633 | 2.810131641 |
| IL1RL1 | 9173 | 0.511860626 | -0.113626033 | 4.685783412 | 1.59E-05 | 0.000354756 | 2.808946781 |
| LOC650874 | 650874 | 0.363959785 | -0.007886855 | 4.685720339 | 1.59E-05 | 0.000354756 | 2.8087325 |
| LOC649397 | 649397 | 0.356809632 | -0.042515737 | 4.684333941 | 1.60E-05 | 0.000355353 | 2.804022711 |
| SCRG1 | 11341 | -1.955776027 | -0.328670222 | -4.68430967 | 1.60E-05 | 0.000355353 | 2.803940265 |
| PPFIA1 | 8500 | 0.444078114 | -0.083814929 | 4.397516136 | 4.42E-05 | 0.000714732 | 1.84401641 |
| LOC651345 | 651345 | 0.58131115 | -0.08175271 | 4.683731084 | 1.60E-05 | 0.000355589 | 2.801974918 |
| LOC641697 | 641697 | 0.530932517 | -0.124400563 | 4.68351379 | 1.60E-05 | 0.000355589 | 2.801236841 |
| OR2A5 | 393046 | 0.421084743 | -0.066461359 | 4.683498166 | 1.60E-05 | 0.000355589 | 2.801183772 |
| SHCBP1 | 79801 | 0.931421782 | -0.089537687 | 4.682678264 | 1.61E-05 | 0.000356438 | 2.798398976 |
| KCNS1 | 3787 | 0.549229363 | -0.112563297 | 4.680261936 | 1.62E-05 | 0.000359142 | 2.790193223 |
| SNIP1 | 79753 | -0.69338872 | -0.162389893 | -4.679398446 | 1.63E-05 | 0.000359854 | 2.787261314 |
| TMEM70 | 54968 | -0.429714442 | -0.099346239 | -4.679084539 | 1.63E-05 | 0.000359885 | 2.78619553 |
| SFRS2B | 10929 | -0.710909008 | -0.132468885 | -4.679062155 | 1.63E-05 | 0.000359885 | 2.786119536 |
| CACNB3 | 784 | -0.769784363 | -0.056996327 | -4.67885875 | 1.63E-05 | 0.000359946 | 2.785428948 |
| MUC13 | 56667 | 0.59599199 | -0.010990566 | 4.677773341 | 1.64E-05 | 0.00036115 | 2.781744081 |
| EVI5L | 115704 | -0.493898572 | -0.070322915 | -4.677547011 | 1.64E-05 | 0.000361242 | 2.78097576 |
| OR51F1 | 256892 | 0.452484988 | -0.12654601 | 4.67699557 | 1.64E-05 | 0.000361756 | 2.779103855 |
| LOC644634 | 644634 | 0.489201829 | -0.070791221 | 4.674692324 | 1.66E-05 | 0.000364282 | 2.771286431 |
| NRG1 | 3084 | 0.412983431 | -0.068764139 | 4.674591188 | 1.66E-05 | 0.000364282 | 2.770943208 |
| HGD | 3081 | 0.52407933 | -0.154183511 | 4.674214191 | 1.66E-05 | 0.000364572 | 2.769663828 |
| ARRDC5 | 645432 | 0.602874336 | -0.010839645 | 4.673828374 | 1.66E-05 | 0.000364874 | 2.768354564 |
| PUS3 | 83480 | -0.518621525 | -0.052059841 | -4.671861328 | 1.67E-05 | 0.000367167 | 2.761680195 |
| LOC643959 | 643959 | 0.432662299 | 0.039820998 | 4.671772515 | 1.67E-05 | 0.000367167 | 2.761378876 |
| CPN2 | 1370 | 0.493782889 | -0.170217259 | 4.671417076 | 1.67E-05 | 0.000367279 | 2.760172987 |
| PIPOX | 51268 | -0.303716705 | -0.106020892 | -4.671377337 | 1.67E-05 | 0.000367279 | 2.760038168 |
| ZNF627 | 199692 | -0.828922441 | -0.107914267 | -4.671209946 | 1.68E-05 | 0.000367296 | 2.759470284 |
| C1QTNF2 | 114898 | 0.40884093 | -0.041222155 | 4.670798794 | 1.68E-05 | 0.000367634 | 2.758075461 |
| C3orf65 | 646600 | 0.438828641 | -0.041538477 | 4.670543933 | 1.68E-05 | 0.000367766 | 2.757210879 |
| BUB3 | 9184 | -0.757204937 | -0.151915681 | -4.669773617 | 1.68E-05 | 0.000368171 | 2.754597815 |
| PIK3R2 | 5296 | -0.878082024 | -0.265207962 | -4.668302127 | 1.69E-05 | 0.000369916 | 2.749606785 |
| DKFZp667M2411 | 147172 | 0.538491717 | -0.038268186 | 4.667623114 | 1.70E-05 | 0.000370613 | 2.74730394 |
| LOC728080 | 728080 | 0.43141827 | 0.011453731 | 4.666820616 | 1.70E-05 | 0.000371476 | 2.744582496 |
| FAM21B | 55747 | 0.434323172 | -0.05546293 | 4.666181964 | 1.71E-05 | 0.000372122 | 2.742416847 |
| LOC643872 | 643872 | 0.475882675 | 0.024533 | 4.66583127 | 1.71E-05 | 0.000372385 | 2.741227709 |
| P15RS | 55197 | -0.868747999 | -0.27132861 | -4.664848986 | 1.71E-05 | 0.000373493 | 2.737897195 |
| ORC6L | 23594 | 1.189606115 | -0.12317248 | 4.663789139 | 1.72E-05 | 0.000374632 | 2.734304054 |
| ALDH6A1 | 4329 | -0.774896125 | -0.114153537 | -4.589042095 | 2.25E-05 | 0.000455555 | 2.481853754 |
| PDE7A | 5150 | 0.427031808 | -0.09756389 | 4.662941944 | 1.73E-05 | 0.000375021 | 2.731432129 |
| PLGLA | 285189 | 0.405468112 | -0.097201741 | 4.660944714 | 1.74E-05 | 0.000377187 | 2.724662624 |
| SOBP | 55084 | -0.661976952 | -0.113006778 | -4.660875503 | 1.74E-05 | 0.000377187 | 2.724428063 |
| SNORD100 | 594838 | 0.335463097 | -0.001291148 | 4.66006158 | 1.74E-05 | 0.000378082 | 2.721669717 |
| TMEM56 | 148534 | -0.327018949 | -0.093657777 | -4.658764187 | 1.75E-05 | 0.000379324 | 2.717273375 |
| POLR3C | 10623 | -0.703443641 | -0.120836687 | -4.658728891 | 1.75E-05 | 0.000379324 | 2.717153777 |
| LOC647707 | 647707 | 0.436408867 | -0.092089715 | 4.658441298 | 1.75E-05 | 0.000379324 | 2.716179319 |
| SHBG | 6462 | 0.317361086 | -0.098049809 | 4.658325154 | 1.76E-05 | 0.000379324 | 2.715785794 |
| RRAGA | 10670 | -0.72649533 | -0.159586051 | -4.65822888 | 1.76E-05 | 0.000379324 | 2.715459597 |
| FLYWCH1 | 84256 | -0.398223391 | -0.112837633 | -4.656322975 | 1.77E-05 | 0.000381715 | 2.709002619 |
| CTXN3 | 613212 | 0.501966286 | -0.119507652 | 4.653341699 | 1.79E-05 | 0.000385098 | 2.698904865 |
| PSMB7 | 5695 | -0.812669074 | -0.298920764 | -4.653250965 | 1.79E-05 | 0.000385098 | 2.69859759 |
| GLTPD1 | 80772 | -0.372028113 | -0.147575294 | -4.652888259 | 1.79E-05 | 0.000385389 | 2.697369299 |
| LOC650879 | 650879 | 0.401884746 | -0.060346605 | 4.652635545 | 1.79E-05 | 0.000385527 | 2.696513517 |
| LOC644310 | 644310 | -0.415992237 | -0.170639682 | -4.651646999 | 1.80E-05 | 0.000386684 | 2.693166148 |
| SCAND2 | 54581 | 0.469907206 | -0.054721852 | 4.650967533 | 1.80E-05 | 0.000387365 | 2.690865564 |
| CST7 | 8530 | 0.937720883 | 0.119293201 | 4.650740457 | 1.80E-05 | 0.000387365 | 2.69009675 |
| PACSIN2 | 11252 | -0.724891047 | -0.240075694 | -4.650699639 | 1.80E-05 | 0.000387365 | 2.689958553 |
| CEP164 | 22897 | -0.474841181 | -0.085774695 | -4.650300498 | 1.81E-05 | 0.000387709 | 2.688607221 |
| PPHLN1 | 51535 | 0.325388799 | 0.025793456 | 4.649337725 | 1.81E-05 | 0.000388837 | 2.685347876 |
| gm127 | 401983 | 0.379169892 | -0.099992373 | 4.648462415 | 1.82E-05 | 0.000389846 | 2.682384901 |
| DKFZp434K191 | 29797 | 0.891617871 | -0.091951134 | 4.647881142 | 1.82E-05 | 0.000390446 | 2.6804174 |
| PMAIP1 | 5366 | 0.321902644 | -0.067378993 | 4.647430154 | 1.83E-05 | 0.000390865 | 2.678890965 |
| TRMU | 55687 | -0.381813478 | -0.105226163 | -4.647196348 | 1.83E-05 | 0.00039098 | 2.678099645 |
| CD151 | 977 | -1.318250467 | 0.085218841 | -4.646616388 | 1.83E-05 | 0.000391542 | 2.676136834 |
| HACL1 | 26061 | -0.713130228 | -0.078885431 | -4.646492706 | 1.83E-05 | 0.000391542 | 2.675718263 |
| SMCHD1 | 23347 | 0.400704769 | -0.079828578 | 4.645089124 | 1.84E-05 | 0.000393293 | 2.670968526 |
| ZNF823 | 55552 | -0.501382458 | -0.132411843 | -4.644172461 | 1.85E-05 | 0.000394168 | 2.667866887 |
| UBE2A | 7319 | 0.400276134 | 0.013317979 | 4.642817508 | 1.86E-05 | 0.000395871 | 2.663282765 |
| RPS5 | 6193 | -0.989104451 | -0.273311952 | -4.641569326 | 1.86E-05 | 0.000397429 | 2.659060419 |
| RAD51L3 | 5892 | 0.439583231 | -0.070833086 | 4.641028064 | 1.87E-05 | 0.000397984 | 2.657229608 |
| PSMD8 | 5714 | -0.633524946 | -0.087772094 | -4.640362663 | 1.87E-05 | 0.000398718 | 2.654979029 |
| RPL10A | 4736 | -2.492100605 | -0.405288795 | -4.639945753 | 1.87E-05 | 0.000399098 | 2.653568998 |
| ANKRD35 | 148741 | -0.657136711 | -0.233494159 | -4.638618525 | 1.88E-05 | 0.000400642 | 2.649080566 |
| OPTN | 10133 | -1.105452254 | -0.352391888 | -4.638380177 | 1.89E-05 | 0.000400642 | 2.648274581 |
| HARBI1 | 283254 | -0.343314231 | -0.141912679 | -4.638264233 | 1.89E-05 | 0.000400642 | 2.64788252 |
| DCUN1D4 | 23142 | -0.635961601 | -0.117655513 | -4.637893978 | 1.89E-05 | 0.000400958 | 2.64663054 |
| KIAA1715 | 80856 | -0.840965634 | -0.169433956 | -4.637212801 | 1.89E-05 | 0.00040172 | 2.64432733 |
| PRRG1 | 5638 | -0.715733227 | -0.076643287 | -4.635002795 | 1.91E-05 | 0.000404257 | 2.636855893 |
| PBX2 | 5089 | -0.876059557 | -0.264151271 | -4.63478754 | 1.91E-05 | 0.000404274 | 2.636128261 |
| RPUSD4 | 84881 | -0.756018236 | -0.122145201 | -4.634690585 | 1.91E-05 | 0.000404274 | 2.635800528 |
| LOC651892 | 651892 | 0.499304933 | -0.128481317 | 4.633783435 | 1.92E-05 | 0.000405371 | 2.632734275 |
| WDR87 | 83889 | 0.442969331 | -0.062557459 | 4.633160405 | 1.92E-05 | 0.000406058 | 2.630628536 |
| LOC653140 | 653140 | 0.532875697 | -0.033136767 | 4.632374061 | 1.93E-05 | 0.000406984 | 2.627971009 |
| LOC441763 | 441763 | -2.254535595 | -0.003084652 | -4.630351933 | 1.94E-05 | 0.000409719 | 2.621137997 |
| HSD17B12 | 51144 | -0.840288107 | -0.242457653 | -4.626846125 | 1.96E-05 | 0.000414259 | 2.609294755 |
| STK36 | 27148 | -0.761666102 | -0.102251814 | -4.626802542 | 1.96E-05 | 0.000414259 | 2.609147551 |
| LOC653773 | 653773 | -0.889576318 | -0.242426155 | -4.626671761 | 1.97E-05 | 0.000414259 | 2.608705831 |
| ATP13A4 | 84239 | 0.426750767 | -0.134373184 | 4.625924829 | 1.97E-05 | 0.000415145 | 2.606183148 |
| GPR42 | 2866 | 0.478211881 | -0.072279918 | 4.625672712 | 1.97E-05 | 0.000415298 | 2.605331693 |
| MAD2L2 | 10459 | -0.624899303 | -0.145778068 | -4.624886694 | 1.98E-05 | 0.000416244 | 2.602677275 |
| FAT3 | 120114 | -0.856007177 | -0.090077596 | -4.624556803 | 1.98E-05 | 0.000416513 | 2.601563281 |
| TDRD9 | 122402 | 0.468569381 | -0.036868634 | 4.624061915 | 1.98E-05 | 0.000417028 | 2.599892186 |
| ACP1 | 52 | -1.061173836 | -0.109361319 | -4.623645784 | 1.99E-05 | 0.000417427 | 2.598487093 |
| LOC650706 | 650706 | 0.411747652 | -0.136736237 | 4.623352392 | 1.99E-05 | 0.000417642 | 2.597496474 |
| COPB1 | 1315 | -0.788040276 | -0.164919505 | -4.62272 | 1.99E-05 | 0.000418323 | 2.595361342 |
| UNC50 | 25972 | -0.95934541 | -0.237121313 | -4.622599108 | 1.99E-05 | 0.000418323 | 2.594953191 |
| LOC401237 | 401237 | 0.369890425 | -0.002264243 | 4.622066836 | 2.00E-05 | 0.000418896 | 2.593156219 |
| FOXO3 | 2309 | -1.110999381 | -0.347506964 | -4.62170306 | 2.00E-05 | 0.000419144 | 2.591928154 |
| PLEC1 | 5339 | 0.520943051 | -0.055285244 | 4.621410572 | 2.00E-05 | 0.000419144 | 2.590940778 |
| HTR3D | 200909 | 0.555820252 | -0.068045413 | 4.621307638 | 2.00E-05 | 0.000419144 | 2.590593304 |
| CAND2 | 23066 | -0.756115975 | -0.102104849 | -4.621210694 | 2.00E-05 | 0.000419144 | 2.590266053 |
| LOC441120 | 441120 | 0.415742534 | -0.097681309 | 4.621086879 | 2.01E-05 | 0.000419144 | 2.589848097 |
| CAPSL | 133690 | 0.438867423 | -0.068556431 | 4.621010437 | 2.01E-05 | 0.000419144 | 2.58959006 |
| C14orf102 | 55051 | -0.797413339 | -0.221392807 | -4.62022886 | 2.01E-05 | 0.000420094 | 2.586951884 |
| THUMPD2 | 80745 | -0.870917121 | -0.065570274 | -4.619889344 | 2.01E-05 | 0.000420381 | 2.585805929 |
| ARL1 | 400 | -0.841165059 | -0.204824291 | -4.61963638 | 2.02E-05 | 0.000420539 | 2.584952133 |
| ZNF260 | 339324 | -0.644025865 | -0.119919843 | -4.619476034 | 2.02E-05 | 0.000420557 | 2.584410953 |
| LOC647331 | 647331 | 0.359427428 | -0.022229699 | 4.618972141 | 2.02E-05 | 0.000421093 | 2.582710325 |
| NARS | 4677 | -0.972165939 | -0.221357625 | -4.618579113 | 2.02E-05 | 0.000421426 | 2.581383926 |
| C2orf28 | 51374 | -0.760638463 | -0.248407262 | -4.618454678 | 2.02E-05 | 0.000421426 | 2.580963991 |
| GORASP2 | 26003 | -0.778691931 | -0.143591073 | -4.618260776 | 2.03E-05 | 0.000421488 | 2.580309631 |
| SNHG3-RCC1 | 751867 | -0.479846494 | -0.119241085 | -4.618118171 | 2.03E-05 | 0.000421488 | 2.579828394 |
| ARPP19 | 10776 | -1.086248799 | -0.33255459 | -4.616134532 | 2.04E-05 | 0.000424039 | 2.573135085 |
| SERPINB3 | 6317 | 0.586296966 | -0.185026174 | 4.615548993 | 2.05E-05 | 0.000424703 | 2.571159581 |
| HERC4 | 26091 | 0.433811276 | -0.030684719 | 4.614077614 | 2.06E-05 | 0.000426716 | 2.566195936 |
| GPR35 | 2859 | 0.426695314 | -0.142569372 | 4.612595936 | 2.07E-05 | 0.000428529 | 2.561198297 |
| ACTN4 | 81 | -0.899912794 | -0.171448292 | -4.610000629 | 2.09E-05 | 0.000432292 | 2.552446249 |
| NFIA | 4774 | -1.234323856 | -0.212073107 | -4.608658297 | 2.10E-05 | 0.00043414 | 2.547920469 |
| LOC650683 | 650683 | 0.389061834 | -0.145000252 | 4.608362982 | 2.10E-05 | 0.00043437 | 2.546924874 |
| LOC652344 | 652344 | 0.568710777 | 0.01819997 | 4.607357278 | 2.11E-05 | 0.000435704 | 2.543534565 |
| NOS2A | 4843 | 0.559596434 | -0.129434083 | 4.605673173 | 2.12E-05 | 0.000438101 | 2.537858098 |
| PIGQ | 9091 | -0.630350127 | -0.117998966 | -4.60536371 | 2.12E-05 | 0.000438355 | 2.536815127 |
| LRIT1 | 26103 | 0.426545148 | -0.009515505 | 4.605072729 | 2.12E-05 | 0.00043858 | 2.53583447 |
| RBP1 | 5947 | -0.88586416 | -0.178972951 | -4.604728938 | 2.13E-05 | 0.000438889 | 2.534675877 |
| LOC647122 | 647122 | 0.434883492 | -0.05759636 | 4.604568256 | 2.13E-05 | 0.000438911 | 2.534134381 |
| LOC441698 | 441698 | 0.479381752 | -0.044574276 | 4.603654271 | 2.13E-05 | 0.000439695 | 2.53105445 |
| COL13A1 | 1305 | -0.718610736 | -0.172333085 | -4.603536679 | 2.14E-05 | 0.000439695 | 2.530658208 |
| NME1 | 4830 | -0.965905401 | -0.151876393 | -4.60348207 | 2.14E-05 | 0.000439695 | 2.530474201 |
| KIAA0226 | 9711 | 0.578553685 | -0.019983049 | 4.602952191 | 2.14E-05 | 0.00044017 | 2.528688786 |
| LOC650689 | 650689 | 0.598711578 | -0.098883439 | 4.602887282 | 2.14E-05 | 0.00044017 | 2.528470082 |
| FLJ44048 | 401024 | 0.400873685 | -0.055200364 | 4.602369946 | 2.14E-05 | 0.000440753 | 2.526727036 |
| SLC9A6 | 10479 | 0.425957247 | 0.004714395 | 4.601997183 | 2.15E-05 | 0.00044111 | 2.525471152 |
| MGC39372 | 221756 | 0.493926197 | 0.074769793 | 4.601681653 | 2.15E-05 | 0.000441175 | 2.524408133 |
| COPS5 | 10987 | -0.909925415 | -0.195817471 | -4.601663896 | 2.15E-05 | 0.000441175 | 2.52434831 |
| LOC388080 | 388080 | 0.440786426 | -0.054789325 | 4.601189614 | 2.15E-05 | 0.000441692 | 2.522750518 |
| TBN | 129685 | 0.320902132 | -0.036501516 | 4.599893879 | 2.16E-05 | 0.000443507 | 2.518385765 |
| ZNF207 | 7756 | -0.806421838 | -0.24397429 | -4.598180998 | 2.18E-05 | 0.000445994 | 2.512616726 |
| CGI-96 | 27341 | 0.506381843 | -0.101290404 | 4.597936136 | 2.18E-05 | 0.000446151 | 2.511792103 |
| C17orf56 | 146705 | 0.529052492 | -0.064783492 | 4.596981887 | 2.19E-05 | 0.000446977 | 2.508578681 |
| CTPS | 1503 | -0.738657417 | -0.06921979 | -4.596962787 | 2.19E-05 | 0.000446977 | 2.508514367 |
| ARMET | 7873 | -0.996195905 | -0.21865999 | -4.596112296 | 2.19E-05 | 0.000447667 | 2.505650618 |
| RPS11 | 6205 | -0.563521942 | -0.036693941 | -4.595713583 | 2.20E-05 | 0.000448072 | 2.504308172 |
| ITPK1 | 3705 | -0.617782778 | -0.100609358 | -4.595522589 | 2.20E-05 | 0.000448146 | 2.503665124 |
| PMS2CL | 441194 | 0.441869974 | -0.079710307 | 4.594906191 | 2.20E-05 | 0.000448899 | 2.501589888 |
| LOC388923 | 388923 | 0.358514502 | -0.014613379 | 4.594105827 | 2.21E-05 | 0.000449797 | 2.498895489 |
| ADAM19 | 8728 | 0.979295784 | 0.232210502 | 4.594056081 | 2.21E-05 | 0.000449797 | 2.498728027 |
| TBX22 | 50945 | 0.334238402 | -0.021313462 | 4.592779232 | 2.22E-05 | 0.000451616 | 2.494430039 |
| UQCRFS1 | 7386 | -0.825280044 | -0.129610936 | -4.592361211 | 2.22E-05 | 0.000452056 | 2.493023067 |
| IP6K1 | 9807 | -0.664667596 | -0.13796858 | -4.591632402 | 2.23E-05 | 0.000452998 | 2.49057019 |
| FH | 2271 | -0.963336621 | -0.283617171 | -4.590076177 | 2.24E-05 | 0.000455049 | 2.48533318 |
| LOC643621 | 643621 | 0.519286071 | -0.196501939 | 4.589698423 | 2.24E-05 | 0.000455427 | 2.484062089 |
| LOC643936 | 643936 | 0.480043538 | -0.033630628 | 4.589353457 | 2.25E-05 | 0.000455555 | 2.48290137 |
| TRIM6 | 117854 | -0.566878586 | -0.206468999 | -4.589208317 | 2.25E-05 | 0.000455555 | 2.482413022 |
| NOL10 | 79954 | 0.407897612 | -0.009414517 | 4.589138828 | 2.25E-05 | 0.000455555 | 2.482179219 |
| TMEM84 | 283673 | 0.566661648 | -0.045688587 | 4.588191978 | 2.26E-05 | 0.000456417 | 2.4789936 |
| DEFB110 | 245913 | 0.388158718 | -0.057246119 | 4.588173419 | 2.26E-05 | 0.000456417 | 2.478931163 |
| GSTA3 | 2940 | 0.597807767 | 0.034487313 | 4.588079158 | 2.26E-05 | 0.000456417 | 2.478614044 |
| MTMR11 | 10903 | 0.260754737 | -0.014576033 | 4.587615882 | 2.26E-05 | 0.000456529 | 2.477055512 |
| TMEM11 | 8834 | -0.354905708 | -0.124314237 | -4.587544727 | 2.26E-05 | 0.000456529 | 2.476816139 |
| LOC647121 | 647121 | 0.342027608 | 0.026152722 | 4.587386822 | 2.26E-05 | 0.000456529 | 2.476284944 |
| ZNF649 | 65251 | -0.399321029 | -0.106379501 | -4.58729176 | 2.26E-05 | 0.000456529 | 2.475965159 |
| PSMD5 | 5711 | -0.471300391 | -0.115512527 | -4.586892361 | 2.27E-05 | 0.000456945 | 2.474621624 |
| ADAM12 | 8038 | 0.474772455 | -0.060365675 | 4.58396891 | 2.29E-05 | 0.000461493 | 2.464789134 |
| CNPY4 | 245812 | 0.421490524 | -0.039794789 | 4.583105948 | 2.30E-05 | 0.000462441 | 2.461887287 |
| LOC644646 | 644646 | -0.64338012 | -0.402955163 | -4.582799403 | 2.30E-05 | 0.00046271 | 2.460856544 |
| SAMD4B | 55095 | -0.65826971 | -0.026731512 | -4.582311177 | 2.30E-05 | 0.000463278 | 2.459214973 |
| RAB11FIP1 | 80223 | 0.416655929 | -0.021425311 | 4.581976923 | 2.31E-05 | 0.000463594 | 2.458091154 |
| AFAP1L1 | 134265 | -0.468579325 | -0.064718267 | -4.581009587 | 2.31E-05 | 0.000464956 | 2.454839024 |
| MGC40489 | 146880 | 0.520957099 | 0.00428088 | 4.580571826 | 2.32E-05 | 0.000465445 | 2.453367401 |
| USF1 | 7391 | -1.211143035 | -0.351599618 | -4.578430644 | 2.34E-05 | 0.000468529 | 2.446170354 |
| ILK | 3611 | -0.872059314 | -0.258341117 | -4.577272075 | 2.34E-05 | 0.000470227 | 2.442276778 |
| C16orf73 | 254528 | 0.426280354 | -0.075516726 | 4.576871438 | 2.35E-05 | 0.000470658 | 2.440930477 |
| DGCR6 | 8214 | -0.518355963 | -0.114344295 | -4.576510946 | 2.35E-05 | 0.000471023 | 2.439719128 |
| HBXIP | 10542 | -0.781740843 | -0.204815513 | -4.576364304 | 2.35E-05 | 0.00047103 | 2.439226382 |
| LOC648132 | 648132 | 0.53348784 | -0.096044732 | 4.576184109 | 2.35E-05 | 0.000471093 | 2.438620907 |
| LOC643465 | 643465 | 0.414371755 | -0.049314345 | 4.575407075 | 2.36E-05 | 0.000472158 | 2.436010105 |
| C6orf97 | 80129 | 0.395709396 | 0.062868333 | 4.575026923 | 2.36E-05 | 0.000472557 | 2.434732888 |
| MAST2 | 23139 | -0.460289359 | -0.094384594 | -4.574242221 | 2.37E-05 | 0.000473382 | 2.43209664 |
| RNF212 | 285498 | 0.342315822 | -0.059552976 | 4.574109885 | 2.37E-05 | 0.000473382 | 2.431652072 |
| LOC285176 | 285176 | 0.699587939 | 0.016660915 | 4.573802035 | 2.37E-05 | 0.000473661 | 2.430617908 |
| ATG5 | 9474 | -0.618964977 | -0.140264278 | -4.573522879 | 2.38E-05 | 0.000473892 | 2.429680163 |
| NDUFV1 | 4723 | -0.553354681 | -0.04258718 | -4.573090414 | 2.38E-05 | 0.000474382 | 2.428227477 |
| LOC650780 | 650780 | 0.280894804 | -0.058770547 | 4.572728586 | 2.38E-05 | 0.000474753 | 2.427012116 |
| LOC654189 | 654189 | 0.392351424 | -0.049803974 | 4.572426996 | 2.39E-05 | 0.000475023 | 2.425999127 |
| UNKL | 64718 | -0.434259853 | -0.147778513 | -4.572094168 | 2.39E-05 | 0.000475335 | 2.42488125 |
| LOC554208 | 554208 | 0.509384161 | 0.041729628 | 4.571958675 | 2.39E-05 | 0.000475335 | 2.424426176 |
| C12orf31 | 84298 | -0.791060541 | -0.236122581 | -4.571420911 | 2.39E-05 | 0.000476005 | 2.422620084 |
| ZNF324B | 388569 | 0.382150767 | -0.078698861 | 4.57126293 | 2.40E-05 | 0.000476005 | 2.422089523 |
| LOC652618 | 652618 | 0.4921227 | -0.095165292 | 4.571067854 | 2.40E-05 | 0.000476005 | 2.421434389 |
| NEK2 | 4751 | 0.406355959 | -0.004612574 | 4.570996063 | 2.40E-05 | 0.000476005 | 2.421193295 |
| ABCB6 | 10058 | -0.578471921 | -0.0875278 | -4.56894715 | 2.42E-05 | 0.000478764 | 2.414313211 |
| C12orf57 | 113246 | -0.870516034 | -0.178745708 | -4.568652392 | 2.42E-05 | 0.000479026 | 2.413323559 |
| PTS | 5805 | -0.676154226 | -0.104966445 | -4.568321437 | 2.42E-05 | 0.000479125 | 2.412212413 |
| HINT2 | 84681 | -0.579473424 | -0.049195017 | -4.568208138 | 2.42E-05 | 0.000479125 | 2.411832032 |
| SERBP1 | 26135 | -0.779691879 | -0.097475192 | -4.568170938 | 2.42E-05 | 0.000479125 | 2.411707142 |
| RTN4 | 57142 | -1.006420779 | -0.256490188 | -4.568007077 | 2.42E-05 | 0.000479164 | 2.411157017 |
| LOC649167 | 649167 | 0.485076749 | -0.039025651 | 4.56763364 | 2.43E-05 | 0.000479372 | 2.409903327 |
| TMEM106B | 54664 | -0.803854672 | -0.076741478 | -4.567603249 | 2.43E-05 | 0.000479372 | 2.409801302 |
| KCNJ13 | 3769 | 0.407333419 | -0.169886451 | 4.567342425 | 2.43E-05 | 0.000479409 | 2.408925703 |
| UBE2D2 | 7322 | -0.510241011 | -0.132533631 | -4.566653422 | 2.44E-05 | 0.000480272 | 2.406612808 |
| SOD1 | 6647 | -0.943918344 | -0.266700261 | -4.566276545 | 2.44E-05 | 0.000480676 | 2.405347749 |
| RPL30 | 6156 | -0.796736535 | -0.086193317 | -4.565943061 | 2.44E-05 | 0.000481005 | 2.404228387 |
| DHRS7 | 51635 | -0.868589347 | -0.244684866 | -4.56464859 | 2.45E-05 | 0.000482983 | 2.39988378 |
| NT5DC1 | 221294 | -0.632539386 | -0.077621253 | -4.563886417 | 2.46E-05 | 0.000484052 | 2.397325993 |
| RPAIN | 84268 | -0.685218045 | -0.158343139 | -4.563550653 | 2.46E-05 | 0.000484389 | 2.39619926 |
| LOC402057 | 402057 | -0.644654249 | -0.148453164 | -4.563017147 | 2.47E-05 | 0.000485066 | 2.394409044 |
| NBPF10 | 440673 | 0.440501206 | -0.159076303 | 4.562576415 | 2.47E-05 | 0.000485585 | 2.39293021 |
| FAM91A1 | 157769 | 0.363293528 | -0.056547017 | 4.561273702 | 2.48E-05 | 0.000487596 | 2.388559488 |
| MGAT4C | 25834 | 0.404757878 | 0.002343865 | 4.561093023 | 2.48E-05 | 0.000487666 | 2.387953341 |
| OXSM | 54995 | -0.368502343 | -0.127068142 | -4.560355636 | 2.49E-05 | 0.000488586 | 2.385479648 |
| MAGEB18 | 286514 | 0.407301274 | -0.004224093 | 4.560282937 | 2.49E-05 | 0.000488586 | 2.385235776 |
| IL4 | 3565 | 0.530993329 | -0.077682402 | 4.559885835 | 2.49E-05 | 0.000489032 | 2.38390372 |
| CLN5 | 1203 | -0.886036365 | -0.147138288 | -4.559416461 | 2.50E-05 | 0.000489605 | 2.382329302 |
| KRT26 | 353288 | 0.434030139 | -0.102389782 | 4.558356186 | 2.51E-05 | 0.00049121 | 2.378773113 |
| SOLH | 6650 | 0.654746614 | 0.093558812 | 4.5575453 | 2.52E-05 | 0.000492383 | 2.376053651 |
| ARF6 | 382 | 0.305504716 | -0.072471278 | 4.556900459 | 2.52E-05 | 0.000493267 | 2.373891218 |
| OVOS2 | 144203 | 0.399022917 | -0.008236128 | 4.556625421 | 2.52E-05 | 0.000493505 | 2.37296894 |
| LOC644922 | 644922 | 0.368789948 | -0.033536886 | 4.556375919 | 2.53E-05 | 0.000493668 | 2.372132314 |
| MRPS26 | 64949 | -0.562811467 | -0.164405797 | -4.556253193 | 2.53E-05 | 0.000493668 | 2.371720799 |
| RHOBTB3 | 22836 | -1.18265613 | -0.341247021 | -4.555847296 | 2.53E-05 | 0.000494136 | 2.370359816 |
| SLC39A1 | 27173 | -0.797677802 | -0.232722282 | -4.555649923 | 2.53E-05 | 0.000494237 | 2.369698041 |
| TMEM106C | 79022 | -0.846542879 | -0.19353113 | -4.555188773 | 2.54E-05 | 0.000494803 | 2.368151897 |
| TMEM43 | 79188 | -0.787954786 | -0.137508163 | -4.553972001 | 2.55E-05 | 0.000496594 | 2.364072662 |
| ATP5H | 10476 | -0.711428669 | -0.191217268 | -4.553893058 | 2.55E-05 | 0.000496594 | 2.363808024 |
| COL2A1 | 1280 | 0.414244907 | 0.036455408 | 4.553708618 | 2.55E-05 | 0.000496674 | 2.363189738 |
| PRPF4B | 8899 | 0.58774041 | -0.063159924 | 4.553501819 | 2.55E-05 | 0.000496794 | 2.362496512 |
| SNX3 | 8724 | 0.463745718 | 0.008398669 | 4.553149369 | 2.55E-05 | 0.000497171 | 2.361315079 |
| PHF8 | 23133 | 0.470649837 | 0.035448447 | 4.552706399 | 2.56E-05 | 0.000497463 | 2.359830279 |
| LOC642981 | 642981 | 0.424685796 | -0.11140371 | 4.551709249 | 2.57E-05 | 0.000498981 | 2.356488165 |
| KIAA0649 | 9858 | -0.394768805 | -0.125979894 | -4.55099752 | 2.57E-05 | 0.000499962 | 2.354102899 |
| OSTM1 | 28962 | -0.586105045 | -0.164047887 | -4.550725937 | 2.58E-05 | 0.000499962 | 2.353192771 |
| PNMA1 | 9240 | -0.657909971 | -0.014087526 | -4.550687132 | 2.58E-05 | 0.000499962 | 2.353062732 |
| NPM3 | 10360 | -0.650133425 | -0.07826298 | -4.55060205 | 2.58E-05 | 0.000499962 | 2.352777613 |
| MYO15B | 80022 | 0.3409112 | -0.0933582 | 4.550011125 | 2.58E-05 | 0.000500766 | 2.350797427 |
| FKBP1A | 2280 | -0.758845374 | 0.063568045 | -4.549815202 | 2.59E-05 | 0.000500868 | 2.350140915 |
| CCDC92 | 80212 | -0.639387331 | -0.114236455 | -4.54923842 | 2.59E-05 | 0.000501039 | 2.348208282 |
| LOC651850 | 651850 | 0.469527106 | -0.098567066 | 4.549226629 | 2.59E-05 | 0.000501039 | 2.348168773 |
| CEBPE | 1053 | 0.437301899 | -0.017270133 | 4.548667463 | 2.60E-05 | 0.000501435 | 2.346295281 |
| CKAP4 | 10970 | -0.996358039 | -0.154254374 | -4.548516543 | 2.60E-05 | 0.00050144 | 2.345789638 |
| MOCS1 | 4337 | -0.791984444 | -0.144163273 | -4.547383564 | 2.61E-05 | 0.000502495 | 2.341993977 |
| CADPS2 | 93664 | -0.658704734 | -0.083750007 | -4.547012142 | 2.61E-05 | 0.000502912 | 2.340749753 |
| PTPN9 | 5780 | -0.249212575 | -0.096308888 | -4.546230441 | 2.62E-05 | 0.000503756 | 2.338131298 |
| CBX3 | 11335 | -0.746719807 | -0.171751173 | -4.546144972 | 2.62E-05 | 0.000503756 | 2.337845015 |
| C19orf70 | 125988 | -1.008343894 | -0.41468024 | -4.545854285 | 2.62E-05 | 0.000503756 | 2.336871366 |
| GADD45GIP1 | 90480 | -0.403824802 | -0.216770668 | -4.545536859 | 2.62E-05 | 0.000503756 | 2.335808193 |
| FAM22C | 727807 | 0.444394627 | -0.152427388 | 4.545432878 | 2.63E-05 | 0.000503756 | 2.335459929 |
| TRIOBP | 11078 | -0.810122715 | -0.145632705 | -4.545371507 | 2.63E-05 | 0.000503756 | 2.335254382 |
| LOC440895 | 440895 | 0.613752617 | 0.001400689 | 4.545327569 | 2.63E-05 | 0.000503756 | 2.335107225 |
| MRPL22 | 29093 | -0.659041855 | -0.116466148 | -4.545207621 | 2.63E-05 | 0.000503756 | 2.334705492 |
| ENPP7 | 339221 | 0.390310847 | -0.050068362 | 4.545167213 | 2.63E-05 | 0.000503756 | 2.334570159 |
| HOXA3 | 3200 | 0.442291068 | -0.089722893 | 4.5446258 | 2.63E-05 | 0.000504479 | 2.332756923 |
| LOC647519 | 647519 | 0.481339544 | -0.154788046 | 4.544310357 | 2.64E-05 | 0.000504799 | 2.331700528 |
| SLC27A5 | 10998 | -0.409268754 | -0.170799485 | -4.543276246 | 2.65E-05 | 0.000506409 | 2.328237612 |
| LOC399744 | 399744 | 0.50787993 | 0.028535087 | 4.543020785 | 2.65E-05 | 0.000506519 | 2.327382213 |
| LCLAT1 | 253558 | -0.647533598 | -0.105607331 | -4.542941632 | 2.65E-05 | 0.000506519 | 2.327117177 |
| BDNF | 627 | 0.4128187 | -0.063271352 | 4.542613663 | 2.65E-05 | 0.000506863 | 2.326019031 |
| MGC10997 | 84741 | 0.622276195 | -0.026725027 | 4.541908407 | 2.66E-05 | 0.000507887 | 2.32365773 |
| HCST | 10870 | 1.023755763 | 0.102277429 | 4.541549881 | 2.66E-05 | 0.000508042 | 2.322457402 |
| LOC283767 | 283767 | 0.38045299 | -0.020797274 | 4.541320032 | 2.66E-05 | 0.00050821 | 2.321687902 |
| TBL1X | 6907 | -0.738456675 | -0.161729642 | -4.540886243 | 2.67E-05 | 0.000508601 | 2.320235692 |
| C1orf161 | 126868 | 0.433345778 | -0.078454728 | 4.540830708 | 2.67E-05 | 0.000508601 | 2.32004978 |
| COG2 | 22796 | -0.561989485 | -0.133073629 | -4.540465039 | 2.67E-05 | 0.000508881 | 2.318825676 |
| DNAJA2 | 10294 | -0.774241073 | -0.165379845 | -4.540270835 | 2.67E-05 | 0.000508881 | 2.318175585 |
| ARHGAP30 | 257106 | 0.52665554 | -0.013570524 | 4.540267412 | 2.67E-05 | 0.000508881 | 2.318164126 |
| LOC732226 | 732226 | 0.500534083 | -0.137372005 | 4.540065581 | 2.68E-05 | 0.000509 | 2.317488518 |
| CSRP2BP | 57325 | -0.544028306 | -0.187515278 | -4.539808994 | 2.68E-05 | 0.000509012 | 2.316629639 |
| M6PRBP1 | 10226 | -0.892307335 | -0.293804768 | -4.539786803 | 2.68E-05 | 0.000509012 | 2.316555362 |
| C16orf57 | 79650 | -0.594742247 | -0.140836224 | -4.539380143 | 2.68E-05 | 0.000509502 | 2.315194192 |
| LOC650188 | 650188 | 0.564903505 | -0.103348175 | 4.53878567 | 2.69E-05 | 0.000510331 | 2.313204481 |
| LOC286310 | 286310 | 0.387835169 | 0.006710849 | 4.538330027 | 2.69E-05 | 0.000510911 | 2.311679524 |
| C1orf149 | 64769 | -0.486730994 | -0.144866675 | -4.53810966 | 2.69E-05 | 0.000510989 | 2.310942019 |
| NKIRAS2 | 28511 | -0.565536867 | -0.133664606 | -4.537959326 | 2.70E-05 | 0.000510989 | 2.310438906 |
| LOC283029 | 283029 | 0.552671553 | -0.142693606 | 4.537880135 | 2.70E-05 | 0.000510989 | 2.310173883 |
| SMN1 | 6606 | 0.486239954 | -0.060511812 | 4.537399161 | 2.70E-05 | 0.000511616 | 2.308564305 |
| IGF2BP1 | 10642 | -0.350903798 | -0.108035261 | -4.536893784 | 2.71E-05 | 0.000512042 | 2.306873148 |
| C19orf62 | 29086 | -0.567082636 | -0.079795327 | -4.536333406 | 2.71E-05 | 0.000512574 | 2.304998047 |
| HSPA6 | 3310 | 0.743074371 | -0.011382471 | 4.536330321 | 2.71E-05 | 0.000512574 | 2.304987724 |
| PTPN18 | 26469 | 0.344369014 | 0.005474151 | 4.533696239 | 2.74E-05 | 0.00051677 | 2.29617523 |
| EIF2B5 | 8893 | -0.707068857 | -0.115421154 | -4.533679031 | 2.74E-05 | 0.00051677 | 2.296117668 |
| PEX11A | 8800 | -0.43932525 | -0.09499047 | -4.533626104 | 2.74E-05 | 0.00051677 | 2.295940621 |
| ACACA | 31 | -0.681283039 | -0.102560486 | -4.533415254 | 2.74E-05 | 0.000516909 | 2.295235321 |
| KIAA0232 | 9778 | -0.391840128 | -0.13348441 | -4.532741801 | 2.75E-05 | 0.000517649 | 2.292982704 |
| STAU1 | 6780 | -0.73262417 | -0.154258129 | -4.532562114 | 2.75E-05 | 0.000517732 | 2.2923817 |
| CNR1 | 1268 | -0.587774599 | 0.002621742 | -4.532187838 | 2.75E-05 | 0.000518172 | 2.291129887 |
| NAP1L1 | 4673 | 0.315552687 | -0.048943386 | 4.531956061 | 2.75E-05 | 0.00051835 | 2.290354705 |
| SAMD10 | 140700 | 0.51492259 | -0.009654867 | 4.531280948 | 2.76E-05 | 0.000519344 | 2.288096886 |
| FTSJ2 | 29960 | 0.381384947 | -0.053258917 | 4.531120658 | 2.76E-05 | 0.000519392 | 2.287560843 |
| MAP1B | 4131 | -1.630478708 | -0.484835619 | -4.529580329 | 2.78E-05 | 0.000521738 | 2.282410133 |
| LOC402100 | 402100 | 0.694283344 | -0.148927433 | 4.52918069 | 2.78E-05 | 0.000522217 | 2.281073919 |
| FANCE | 2178 | -0.553853927 | -0.02788572 | -4.528957207 | 2.78E-05 | 0.000522217 | 2.280326716 |
| SUGT1 | 10910 | -0.503521075 | -0.108174938 | -4.528918485 | 2.78E-05 | 0.000522217 | 2.280197252 |
| TMEM97 | 27346 | -0.694458438 | -0.1361404 | -4.527553329 | 2.80E-05 | 0.000524216 | 2.275633333 |
| DOLK | 22845 | -0.517927597 | -0.025830364 | -4.527437858 | 2.80E-05 | 0.000524216 | 2.275247329 |
| SERPINA3 | 12 | -1.356675931 | -0.459426562 | -4.52703501 | 2.80E-05 | 0.00052452 | 2.273900693 |
| RBBP9 | 10741 | -0.641507115 | -0.062396668 | -4.52700635 | 2.80E-05 | 0.00052452 | 2.273804889 |
| OR4C12 | 283093 | 0.476935166 | -0.096737205 | 4.52465135 | 2.83E-05 | 0.000528564 | 2.265933805 |
| ACMSD | 130013 | 0.537230945 | 0.063603967 | 4.524413827 | 2.83E-05 | 0.000528564 | 2.265140046 |
| PDCD7 | 10081 | -0.710601191 | -0.100040071 | -4.524368435 | 2.83E-05 | 0.000528564 | 2.264988353 |
| COIL | 8161 | -0.671241746 | -0.115473377 | -4.524303391 | 2.83E-05 | 0.000528564 | 2.264770993 |
| GRID2 | 2895 | 0.587221194 | -0.147599564 | 4.523898391 | 2.83E-05 | 0.000529072 | 2.263417619 |
| FLJ36144 | 283685 | 0.332539794 | -0.001478063 | 4.523271255 | 2.84E-05 | 0.000529575 | 2.261322054 |
| KIAA0427 | 9811 | -0.621134109 | -0.049775901 | -4.523229309 | 2.84E-05 | 0.000529575 | 2.261181896 |
| DCTN6 | 10671 | -0.674325753 | -0.109957831 | -4.522933536 | 2.84E-05 | 0.000529642 | 2.260193628 |
| CDC42 | 998 | -0.845511939 | -0.12399245 | -4.5227933 | 2.85E-05 | 0.000529642 | 2.259725071 |
| EIF3K | 27335 | -0.71844205 | -0.208349625 | -4.522479579 | 2.85E-05 | 0.000529715 | 2.258676881 |
| PLEKHB2 | 55041 | -0.793255734 | -0.189025307 | -4.522363896 | 2.85E-05 | 0.000529715 | 2.258290379 |
| RNF148 | 378925 | 0.38034779 | 0.008761043 | 4.522243799 | 2.85E-05 | 0.000529715 | 2.257889131 |
| RAD17 | 5884 | 0.381931486 | 0.001720629 | 4.522221725 | 2.85E-05 | 0.000529715 | 2.257815381 |
| HTR5A | 3361 | 0.425852786 | -0.076277228 | 4.522072136 | 2.85E-05 | 0.000529746 | 2.25731561 |
| SCARB2 | 950 | -0.805011318 | -0.242391259 | -4.521064523 | 2.86E-05 | 0.00053139 | 2.253949415 |
| SLC45A1 | 50651 | -0.475843218 | -0.285733983 | -4.520563741 | 2.87E-05 | 0.000532052 | 2.252276558 |
| LMO3 | 55885 | -1.256977962 | -0.127557997 | -4.520395634 | 2.87E-05 | 0.000532052 | 2.25171502 |
| GRWD1 | 83743 | -0.599291014 | -0.130029158 | -4.520314539 | 2.87E-05 | 0.000532052 | 2.251444135 |
| ASMTL | 8623 | -0.465239072 | -0.108393539 | -4.519775914 | 2.88E-05 | 0.000532669 | 2.249645011 |
| MED20 | 9477 | -0.834748318 | -0.215404019 | -4.519721789 | 2.88E-05 | 0.000532669 | 2.249464229 |
| CXCL5 | 6374 | 0.694722057 | 0.031930993 | 4.519560206 | 2.88E-05 | 0.000532723 | 2.248924531 |
| SNRNP48 | 154007 | -0.349075137 | -0.124316237 | -4.518974633 | 2.88E-05 | 0.000533579 | 2.246968756 |
| ZPBP2 | 124626 | 0.451754092 | -0.033555068 | 4.518375225 | 2.89E-05 | 0.000534462 | 2.244966898 |
| ID2 | 3398 | -1.142753374 | -0.322710745 | -4.515597217 | 2.92E-05 | 0.000539491 | 2.235690808 |
| APOBEC3F | 200316 | 0.364712059 | -0.04506191 | 4.515315777 | 2.92E-05 | 0.000539763 | 2.234751203 |
| C1orf167 | 284498 | 0.402764002 | -0.052284406 | 4.515190153 | 2.92E-05 | 0.000539763 | 2.234331807 |
| CCDC48 | 79825 | 0.473275476 | -0.082569745 | 4.514459873 | 2.93E-05 | 0.000540906 | 2.231893883 |
| ZDHHC11B | 653082 | 0.460972618 | -0.138308867 | 4.514068087 | 2.93E-05 | 0.000541403 | 2.23058605 |
| TMEM200C | 645369 | 0.547127931 | -0.079551605 | 4.513796871 | 2.94E-05 | 0.000541484 | 2.229680726 |
| KRT18 | 3875 | 0.474425606 | -0.108893492 | 4.513761781 | 2.94E-05 | 0.000541484 | 2.229563595 |
| SYVN1 | 84447 | -1.000899888 | -0.167776713 | -4.512437304 | 2.95E-05 | 0.000543774 | 2.225142865 |
| LSMD1 | 84316 | -0.671135488 | -0.090284665 | -4.510627918 | 2.97E-05 | 0.000546925 | 2.219104664 |
| C17orf50 | 146853 | 0.459074561 | -0.023821585 | 4.510540042 | 2.97E-05 | 0.000546925 | 2.218811438 |
| UTP11L | 51118 | -0.609468781 | -0.132759756 | -4.509683834 | 2.98E-05 | 0.000548154 | 2.215954579 |
| WDHD1 | 11169 | 0.348412205 | -0.115327983 | 4.509641782 | 2.98E-05 | 0.000548154 | 2.215814274 |
| PHKB | 5257 | -0.829838363 | -0.230357852 | -4.509363632 | 2.98E-05 | 0.000548438 | 2.21488625 |
| TCEAL1 | 9338 | -0.974946838 | -0.024764066 | -4.509148669 | 2.99E-05 | 0.0005486 | 2.214169059 |
| BAX | 581 | 0.309527226 | -0.039245127 | 4.50877381 | 2.99E-05 | 0.000549072 | 2.212918445 |
| ANXA11 | 311 | -0.621991293 | -0.189246093 | -4.508641366 | 2.99E-05 | 0.000549073 | 2.212476596 |
| KTELC1 | 56983 | -0.809187417 | -0.158611726 | -4.508380303 | 2.99E-05 | 0.000549325 | 2.211605673 |
| LOC644569 | 644569 | 0.505848772 | -0.186383838 | 4.507857672 | 3.00E-05 | 0.000550008 | 2.209862216 |
| FBL | 2091 | -0.692682529 | -0.161839783 | -4.507689079 | 3.00E-05 | 0.000550008 | 2.209299821 |
| LOC647570 | 647570 | 0.375592637 | -0.059189041 | 4.507634328 | 3.00E-05 | 0.000550008 | 2.209117186 |
| DKK3 | 27122 | 0.384132557 | 0.005232549 | 4.50720445 | 3.01E-05 | 0.000550541 | 2.207683254 |
| LOC728734 | 728734 | 0.371845058 | -0.02450369 | 4.507098275 | 3.01E-05 | 0.000550541 | 2.2073291 |
| SCYL1 | 57410 | -0.744460682 | -0.111591748 | -4.50574881 | 3.02E-05 | 0.000552661 | 2.202828214 |
| RIC8B | 55188 | -0.497411643 | -0.104563232 | -4.505441039 | 3.03E-05 | 0.00055293 | 2.201801794 |
| NDUFA12 | 55967 | -1.064544458 | -0.357163337 | -4.505273895 | 3.03E-05 | 0.00055293 | 2.20124438 |
| REPIN1 | 29803 | -0.916723442 | -0.172700394 | -4.505218194 | 3.03E-05 | 0.00055293 | 2.201058625 |
| SET | 6418 | -0.846235986 | -0.2797378 | -4.504531199 | 3.04E-05 | 0.000553762 | 2.198767667 |
| ATPBD4 | 89978 | -0.438449494 | -0.101210364 | -4.50346143 | 3.05E-05 | 0.000555603 | 2.195200594 |
| EBNA1BP2 | 10969 | -0.658554172 | -0.200385416 | -4.501632856 | 3.07E-05 | 0.000558948 | 2.189104291 |
| LOC649971 | 649971 | 0.372702843 | -0.089058424 | 4.501453036 | 3.07E-05 | 0.000559045 | 2.188504853 |
| KRTAP12-1 | 353332 | 0.484287044 | -0.055492447 | 4.500015819 | 3.08E-05 | 0.000561374 | 2.183714243 |
| HNRNPU | 3192 | -0.74138931 | -0.141332495 | -4.499539287 | 3.09E-05 | 0.000561801 | 2.182126007 |
| TMEM126A | 84233 | -0.840283219 | -0.18168036 | -4.499139895 | 3.09E-05 | 0.000562335 | 2.180794931 |
| RABIF | 5877 | -0.337677355 | -0.179354779 | -4.498997393 | 3.10E-05 | 0.000562359 | 2.180320025 |
| ATRIP | 84126 | -0.395435251 | -0.080356449 | -4.497221136 | 3.11E-05 | 0.00056538 | 2.174400998 |
| CCND3 | 896 | -0.793187365 | -0.070361395 | -4.496040033 | 3.13E-05 | 0.000567483 | 2.170465836 |
| OSBPL2 | 9885 | -0.800400492 | -0.176566471 | -4.49563502 | 3.13E-05 | 0.000568034 | 2.169116543 |
| KIF13A | 63971 | 0.403274296 | -0.122278074 | 4.495227374 | 3.14E-05 | 0.000568591 | 2.167758539 |
| LOC644808 | 644808 | 0.363611951 | -0.013914768 | 4.494878929 | 3.14E-05 | 0.00056903 | 2.166597802 |
| OR10T2 | 128360 | 0.400579974 | -0.074081505 | 4.493163622 | 3.16E-05 | 0.000572064 | 2.160884425 |
| RBPMS | 11030 | -0.891809442 | -0.118049718 | -4.49300792 | 3.16E-05 | 0.000572064 | 2.16036586 |
| SERPINA2 | 390502 | 0.507533539 | -0.044119972 | 4.492983542 | 3.16E-05 | 0.000572064 | 2.16028467 |
| DTNBP1 | 84062 | 0.365168237 | -0.020097638 | 4.492360449 | 3.17E-05 | 0.000573061 | 2.158209565 |
| ZNF384 | 171017 | -0.479801932 | -0.06014531 | -4.492006754 | 3.17E-05 | 0.000573171 | 2.157031709 |
| HINFP | 25988 | 0.353010995 | -0.070994564 | 4.49198142 | 3.17E-05 | 0.000573171 | 2.156947345 |
| LOC645478 | 645478 | 0.425459414 | -0.022268163 | 4.491916685 | 3.17E-05 | 0.000573171 | 2.156731771 |
| RNF126 | 55658 | -0.666007053 | -0.071086531 | -4.491372743 | 3.18E-05 | 0.000573748 | 2.154920471 |
| LOC653352 | 653352 | 0.400659692 | -0.071479078 | 4.491027817 | 3.18E-05 | 0.000574184 | 2.153771935 |
| ARL4A | 10124 | -0.576366572 | -0.033898885 | -4.490715887 | 3.19E-05 | 0.000574554 | 2.15273331 |
| C9orf82 | 79886 | -0.506082712 | -0.12439661 | -4.490556931 | 3.19E-05 | 0.000574614 | 2.152204053 |
| BRIX1 | 55299 | -0.773354302 | -0.225545162 | -4.490198983 | 3.19E-05 | 0.000575078 | 2.151012265 |
| LOC642213 | 642213 | 0.421714571 | -0.062330186 | 4.489863095 | 3.20E-05 | 0.000575498 | 2.14989397 |
| OSBPL3 | 26031 | 0.278404296 | 0.016554383 | 4.4893909 | 3.20E-05 | 0.000575919 | 2.14832193 |
| LOC644043 | 644043 | 0.440410846 | -0.027936652 | 4.489247545 | 3.20E-05 | 0.000575919 | 2.147844685 |
| MOAP1 | 64112 | -0.692722617 | -0.04169163 | -4.489083637 | 3.21E-05 | 0.000575919 | 2.147299024 |
| CABP2 | 51475 | 0.309675023 | -0.058644042 | 4.489082498 | 3.21E-05 | 0.000575919 | 2.147295235 |
| NCK2 | 8440 | -0.84842359 | -0.190381873 | -4.488812821 | 3.21E-05 | 0.000575919 | 2.146397486 |
| NDUFA4 | 4697 | -0.859769735 | -0.258838695 | -4.488787696 | 3.21E-05 | 0.000575919 | 2.146313846 |
| UPK3B | 80761 | 0.415255797 | -0.099886574 | 4.488730756 | 3.21E-05 | 0.000575919 | 2.146124299 |
| LOC441811 | 441811 | 0.681784957 | -0.238477485 | 4.488623105 | 3.21E-05 | 0.000575919 | 2.145765942 |
| PROK2 | 60675 | 1.089542229 | 0.129683764 | 4.488358404 | 3.21E-05 | 0.000575974 | 2.144884802 |
| CRHBP | 1393 | 0.474665427 | 0.017976609 | 4.488338766 | 3.21E-05 | 0.000575974 | 2.144819434 |
| CHRNB1 | 1140 | -0.452979328 | -0.156821926 | -4.488064243 | 3.22E-05 | 0.000576094 | 2.143905627 |
| C20orf7 | 79133 | -0.446481868 | -0.02827935 | -4.488022365 | 3.22E-05 | 0.000576094 | 2.143766229 |
| ZNF136 | 7695 | -0.496455286 | -0.106264167 | -4.487517921 | 3.22E-05 | 0.000576647 | 2.142087165 |
| CREB1 | 1385 | 0.296730285 | -0.015172238 | 4.48749325 | 3.22E-05 | 0.000576647 | 2.142005049 |
| LOC647942 | 647942 | 0.425806014 | -0.032536201 | 4.48688406 | 3.23E-05 | 0.000577414 | 2.139977461 |
| LOC653480 | 653480 | 0.447135388 | -0.034727331 | 4.486860002 | 3.23E-05 | 0.000577414 | 2.13989739 |
| EEF2K | 29904 | -0.747081505 | -0.120467902 | -4.485365885 | 3.25E-05 | 0.000580204 | 2.134925056 |
| TXNDC9 | 10190 | -0.791266997 | -0.147254991 | -4.48398885 | 3.26E-05 | 0.000582608 | 2.130343082 |
| DHRS7B | 25979 | -0.541038701 | -0.11837451 | -4.483937549 | 3.26E-05 | 0.000582608 | 2.130172397 |
| LOC645015 | 645015 | 0.363274895 | -0.037498788 | 4.483324801 | 3.27E-05 | 0.000583571 | 2.128133762 |
| FAM89A | 375061 | -0.719050147 | -0.056476615 | -4.483213119 | 3.27E-05 | 0.000583571 | 2.127762206 |
| ZNF362 | 149076 | -0.710803564 | -0.163349435 | -4.482960068 | 3.28E-05 | 0.000583829 | 2.126920348 |
| AKAP1 | 8165 | -0.644862918 | -0.102387982 | -4.482711898 | 3.28E-05 | 0.000584076 | 2.126094747 |
| OSTC | 58505 | -1.657402454 | -0.210007595 | -4.479948465 | 3.31E-05 | 0.000588735 | 2.116903026 |
| PCDH11Y | 83259 | -0.369121544 | -0.143669923 | -4.47771993 | 3.34E-05 | 0.000593114 | 2.109492511 |
| LOC387870 | 387870 | 0.4728043 | -0.034438223 | 4.477080245 | 3.34E-05 | 0.000594185 | 2.107365712 |
| ATP13A3 | 79572 | 0.357643256 | 0.025782413 | 4.476148211 | 3.36E-05 | 0.000595873 | 2.104267192 |
| DHCR7 | 1717 | -0.533706445 | -0.041268497 | -4.475560969 | 3.36E-05 | 0.00059684 | 2.102315086 |
| CCDC23 | 374969 | -0.713623839 | -0.148070645 | -4.474874964 | 3.37E-05 | 0.000597881 | 2.100034832 |
| RAD50 | 10111 | -0.465177468 | -0.108877386 | -4.474811232 | 3.37E-05 | 0.000597881 | 2.099822998 |
| ATG9A | 79065 | -0.492473984 | -0.065392826 | -4.474546255 | 3.37E-05 | 0.000598171 | 2.098942277 |
| ODF3B | 440836 | 0.352627488 | -0.014504148 | 4.474103681 | 3.38E-05 | 0.000598836 | 2.097471325 |
| PCDHB4 | 56131 | -0.570986099 | -0.141445112 | -4.473901252 | 3.38E-05 | 0.00059885 | 2.096798547 |
| PCDHGB4 | 8641 | 0.433798583 | -0.005727813 | 4.473841993 | 3.38E-05 | 0.00059885 | 2.096601603 |
| LOC654187 | 654187 | 0.512476483 | -0.096444816 | 4.473548659 | 3.39E-05 | 0.000599201 | 2.095626736 |
| ZBED3 | 84327 | -0.414676539 | -0.158725931 | -4.472280357 | 3.40E-05 | 0.000601346 | 2.091412019 |
| DUSP13 | 51207 | -0.242869192 | -0.117460483 | -4.471566899 | 3.41E-05 | 0.00060232 | 2.089041376 |
| FRAS1 | 80144 | 0.483221592 | -0.146845029 | 4.471372896 | 3.41E-05 | 0.000602463 | 2.088396785 |
| UTP6 | 55813 | -0.870274829 | -0.271982836 | -4.470568939 | 3.42E-05 | 0.000603632 | 2.085725716 |
| PSMA1 | 5682 | 0.407651621 | -0.133520711 | 4.470219434 | 3.43E-05 | 0.000603726 | 2.084564593 |
| RAB11A | 8766 | -0.885091467 | -0.189107704 | -4.470065192 | 3.43E-05 | 0.000603726 | 2.084052186 |
| MRPL13 | 28998 | -0.734078891 | -0.330931286 | -4.470017066 | 3.43E-05 | 0.000603726 | 2.083892307 |
| LMO4 | 8543 | -1.056277746 | -0.362106661 | -4.46887136 | 3.44E-05 | 0.000605628 | 2.080086453 |
| BUD31 | 8896 | -0.603871498 | -0.14568569 | -4.468563183 | 3.45E-05 | 0.000606016 | 2.079062818 |
| MRPL52 | 122704 | -0.517948436 | -0.070928201 | -4.467881016 | 3.45E-05 | 0.00060701 | 2.076797074 |
| RABGAP1 | 23637 | -1.12210866 | -0.274682013 | -4.46784493 | 3.45E-05 | 0.00060701 | 2.076677222 |
| C9orf109 | 286333 | 0.441601855 | 0.003857898 | 4.46765801 | 3.46E-05 | 0.000607139 | 2.076056422 |
| DRG1 | 4733 | -0.761437474 | -0.202187942 | -4.467083136 | 3.46E-05 | 0.0006081 | 2.074147219 |
| ZDHHC8 | 29801 | -0.740902412 | -0.171977655 | -4.466815573 | 3.47E-05 | 0.000608132 | 2.07325866 |
| THSD1P | 374500 | 0.312930605 | -0.072437398 | 4.465920415 | 3.48E-05 | 0.000609655 | 2.070286101 |
| DNAJC14 | 85406 | -0.508982246 | -0.077322878 | -4.46583428 | 3.48E-05 | 0.000609655 | 2.070000087 |
| BIRC2 | 329 | 0.574908767 | -0.085978857 | 4.465667375 | 3.48E-05 | 0.000609655 | 2.069445881 |
| GNB5 | 10681 | -0.73000188 | -0.125773652 | -4.465600706 | 3.48E-05 | 0.000609655 | 2.069224509 |
| WDR34 | 89891 | -0.333535751 | -0.06020837 | -4.465374609 | 3.49E-05 | 0.00060987 | 2.068473781 |
| FAM46B | 115572 | -1.0581933 | -0.196478033 | -4.464655628 | 3.49E-05 | 0.000611146 | 2.066086608 |
| LSP1 | 4046 | 0.921684236 | 0.213062246 | 4.463622786 | 3.51E-05 | 0.000613101 | 2.062657685 |
| REC8 | 9985 | 0.813502166 | 0.03693335 | 4.46332216 | 3.51E-05 | 0.000613302 | 2.061659715 |
| ASB8 | 140461 | -0.738080745 | -0.244055578 | -4.463278176 | 3.51E-05 | 0.000613302 | 2.061513705 |
| LOC731432 | 731432 | 0.403570488 | -0.009525085 | 4.462396463 | 3.52E-05 | 0.000614937 | 2.058586938 |
| ATP2B3 | 492 | 0.4997739 | -0.129991432 | 4.462141119 | 3.53E-05 | 0.000615218 | 2.057739398 |
| FAM116A | 201627 | -0.783494561 | -0.115213418 | -4.461561107 | 3.53E-05 | 0.00061593 | 2.055814311 |
| PILRB | 29990 | 0.359060158 | -0.000629443 | 4.461235904 | 3.54E-05 | 0.000616363 | 2.054735 |
| PAICS | 10606 | -0.746843262 | -0.077766323 | -4.460516666 | 3.55E-05 | 0.00061747 | 2.052348072 |
| ZFYVE21 | 79038 | -0.778197483 | -0.168335902 | -4.460422661 | 3.55E-05 | 0.00061747 | 2.052036115 |
| DAAM1 | 23002 | -0.826886664 | -0.133473193 | -4.460350232 | 3.55E-05 | 0.00061747 | 2.051795757 |
| MBD2 | 8932 | 0.633002966 | 0.019638869 | 4.459499651 | 3.56E-05 | 0.000619038 | 2.048973243 |
| RPL9 | 6133 | -0.742487395 | -0.014718732 | -4.459379348 | 3.56E-05 | 0.000619038 | 2.048574059 |
| ZNF385D | 79750 | -0.704990317 | -0.091143116 | -4.459216149 | 3.56E-05 | 0.00061912 | 2.048032545 |
| ZNF35 | 7584 | -0.411422953 | -0.089506462 | -4.459063971 | 3.56E-05 | 0.000619134 | 2.047527614 |
| ZNF492 | 57615 | 0.349885736 | -0.091016504 | 4.458844368 | 3.57E-05 | 0.000619134 | 2.046798973 |
| LOC389142 | 389142 | 0.448217081 | -0.121978681 | 4.458813777 | 3.57E-05 | 0.000619134 | 2.046697476 |
| SPTLC1 | 10558 | -0.320543835 | -0.140824394 | -4.458651391 | 3.57E-05 | 0.000619134 | 2.046158697 |
| GOT1 | 2805 | -0.631567475 | -0.092460603 | -4.458568072 | 3.57E-05 | 0.000619134 | 2.045882254 |
| YWHAH | 7533 | -0.834420168 | -0.225786074 | -4.458163922 | 3.57E-05 | 0.000619372 | 2.04454138 |
| NRBP1 | 29959 | -0.5027723 | -0.050395803 | -4.45810073 | 3.58E-05 | 0.000619372 | 2.044331733 |
| LOC644624 | 644624 | 0.276118532 | -0.08373648 | 4.457731701 | 3.58E-05 | 0.000619904 | 2.043107442 |
| ESD | 2098 | -0.817985233 | -0.309665734 | -4.457578809 | 3.58E-05 | 0.000619966 | 2.042600223 |
| ARHGEF12 | 23365 | -0.589677637 | -0.174478036 | -4.457330181 | 3.59E-05 | 0.000620236 | 2.041775417 |
| SF3A3 | 10946 | -0.663311172 | -0.178708407 | -4.456104664 | 3.60E-05 | 0.000622372 | 2.037710189 |
| LOC651166 | 651166 | 0.409491978 | -0.11762549 | 4.454693693 | 3.62E-05 | 0.000624763 | 2.033030471 |
| ACSL1 | 2180 | -1.206647886 | -0.025335884 | -4.454641746 | 3.62E-05 | 0.000624763 | 2.032858192 |
| LOC644683 | 644683 | 0.470569501 | -0.082331891 | 4.453091439 | 3.64E-05 | 0.000627631 | 2.027717219 |
| NPR2 | 4882 | -0.621266324 | -0.092300547 | -4.452807003 | 3.64E-05 | 0.000627984 | 2.0267741 |
| SMARCE1 | 6605 | -0.723552315 | -0.216526254 | -4.452172128 | 3.65E-05 | 0.000629114 | 2.024669113 |
| VCAM1 | 7412 | 0.778174472 | 0.000475086 | 4.451454081 | 3.66E-05 | 0.000630173 | 2.022288544 |
| LOC402145 | 402145 | 0.398602178 | -0.115363762 | 4.451445774 | 3.66E-05 | 0.000630173 | 2.022261006 |
| POLR2D | 5433 | -0.474988724 | -0.134574171 | -4.450992447 | 3.67E-05 | 0.000630903 | 2.020758171 |
| C4orf27 | 54969 | -0.741053711 | -0.159515865 | -4.450655062 | 3.67E-05 | 0.00063115 | 2.01963975 |
| SCARF2 | 91179 | -0.635207276 | -0.032604259 | -4.450633313 | 3.67E-05 | 0.00063115 | 2.019567654 |
| RFNG | 5986 | 0.357502767 | -0.037458457 | 4.449453779 | 3.69E-05 | 0.00063267 | 2.01565787 |
| TECTA | 7007 | 0.26293652 | -0.048723786 | 4.448628879 | 3.70E-05 | 0.000634232 | 2.012923897 |
| GOLGA8G | 283768 | 0.463727726 | -0.035316203 | 4.448300038 | 3.70E-05 | 0.00063469 | 2.011834085 |
| CAPZA2 | 830 | -0.638033934 | -0.132507069 | -4.447336421 | 3.71E-05 | 0.000635768 | 2.008640795 |
| LOC391574 | 391574 | 0.430932281 | -0.127434446 | 4.447082181 | 3.72E-05 | 0.000635943 | 2.007798338 |
| LOC646804 | 646804 | 0.396386301 | -0.149144087 | 4.446998206 | 3.72E-05 | 0.000635943 | 2.007520078 |
| APEG1 | 10290 | -0.908867899 | -0.056970687 | -4.445661965 | 3.74E-05 | 0.000638108 | 2.003092702 |
| TRIM60 | 166655 | 0.575206791 | 0.137656442 | 4.445158947 | 3.74E-05 | 0.000638888 | 2.001426223 |
| RECQL4 | 9401 | 0.540077765 | -0.060609974 | 4.444946541 | 3.74E-05 | 0.000638888 | 2.000722558 |
| RPS14 | 6208 | -0.603348206 | -0.133072434 | -4.444945031 | 3.74E-05 | 0.000638888 | 2.000717555 |
| LOC643236 | 643236 | 0.417375164 | -0.010516067 | 4.444715281 | 3.75E-05 | 0.000639127 | 1.999956451 |
| MRPL33 | 9553 | -0.821346146 | -0.227529636 | -4.443329443 | 3.77E-05 | 0.000641694 | 1.995365935 |
| TUBGCP4 | 27229 | -0.411893349 | -0.084351042 | -4.441818567 | 3.79E-05 | 0.000644554 | 1.990362049 |
| HPS4 | 89781 | 0.347832129 | -0.039975464 | 4.441276292 | 3.79E-05 | 0.000645505 | 1.988566289 |
| RGL4 | 266747 | 0.596563564 | 0.035305257 | 4.440620682 | 3.80E-05 | 0.000646436 | 1.986395365 |
| TOP1P1 | 7151 | 0.30912266 | -0.086405967 | 4.439905298 | 3.81E-05 | 0.000647784 | 1.984026691 |
| TMEM136 | 219902 | -0.542504576 | -0.124735808 | -4.439502453 | 3.82E-05 | 0.000648422 | 1.982692936 |
| GUCA1B | 2979 | 0.337505818 | -0.000312177 | 4.439096329 | 3.82E-05 | 0.000648949 | 1.981348383 |
| FAM136A | 84908 | -0.740385185 | -0.137358134 | -4.438935738 | 3.82E-05 | 0.000648949 | 1.980816734 |
| RPS18 | 6222 | -0.817846754 | -0.245432737 | -4.438903762 | 3.83E-05 | 0.000648949 | 1.980710877 |
| ST3GAL3 | 6487 | -0.547952967 | -0.124107773 | -4.438317054 | 3.83E-05 | 0.000650008 | 1.978768618 |
| LOC651807 | 651807 | 0.289286389 | 0.016730119 | 4.437014103 | 3.85E-05 | 0.000651912 | 1.97445574 |
| LOC644208 | 644208 | 0.578786946 | -0.077564484 | 4.436995199 | 3.85E-05 | 0.000651912 | 1.974393174 |
| LOC130773 | 130773 | -0.420378475 | -0.100124843 | -4.436595475 | 3.86E-05 | 0.000652057 | 1.973070184 |
| ASNS | 440 | -0.99647799 | -0.076173019 | -4.436506831 | 3.86E-05 | 0.000652057 | 1.972776801 |
| LOC653717 | 653717 | 0.566039826 | -0.043897303 | 4.436467215 | 3.86E-05 | 0.000652057 | 1.972645686 |
| LOC642995 | 642995 | 0.265268986 | -0.065960462 | 4.436219648 | 3.86E-05 | 0.000652057 | 1.971826339 |
| MUC6 | 4588 | 0.461759197 | -0.006555271 | 4.436122116 | 3.86E-05 | 0.000652057 | 1.971503556 |
| CUL4A | 8451 | -0.844841259 | -0.155758886 | -4.435907662 | 3.87E-05 | 0.000652057 | 1.970793827 |
| EGLN2 | 112398 | -0.676533232 | -0.258731149 | -4.43582033 | 3.87E-05 | 0.000652057 | 1.970504809 |
| RPS6KA3 | 6197 | 0.446150021 | -0.041865658 | 4.270594916 | 6.88E-05 | 0.000947569 | 1.428930187 |
| KATNAL1 | 84056 | -0.658940354 | -0.179559889 | -4.435738341 | 3.87E-05 | 0.000652057 | 1.970233477 |
| NUP205 | 23165 | -0.739254383 | -0.09671663 | -4.435601396 | 3.87E-05 | 0.000652057 | 1.969780279 |
| KLHL9 | 55958 | -1.004200102 | -0.029333302 | -4.435310925 | 3.87E-05 | 0.00065242 | 1.968819037 |
| MBOAT1 | 154141 | 0.35263565 | -0.051739683 | 4.434976932 | 3.88E-05 | 0.000652906 | 1.967713805 |
| MAGEH1 | 28986 | -0.627148167 | -0.064764616 | -4.434546974 | 3.88E-05 | 0.000653335 | 1.966291076 |
| NUDT18 | 79873 | -0.591180845 | -0.087783399 | -4.433260583 | 3.90E-05 | 0.000655734 | 1.962034819 |
| RNF121 | 55298 | -0.661826555 | -0.070039 | -4.432492549 | 3.91E-05 | 0.000656509 | 1.959493935 |
| PCDHB12 | 56124 | -0.435343978 | -0.135769076 | -4.432323278 | 3.91E-05 | 0.000656509 | 1.958933968 |
| LRRC16 | 55604 | -0.531058751 | -0.110073735 | -4.432318242 | 3.91E-05 | 0.000656509 | 1.958917308 |
| LSM5 | 23658 | -0.568554956 | -0.097638517 | -4.432187096 | 3.92E-05 | 0.000656532 | 1.958483469 |
| LOC642968 | 642968 | 0.395516553 | -0.004435594 | 4.430288737 | 3.94E-05 | 0.000660361 | 1.952204298 |
| FOXD2 | 2306 | -0.757922128 | -0.133364382 | -4.429412219 | 3.95E-05 | 0.000662115 | 1.949305509 |
| NDUFA8 | 4702 | -1.009339105 | -0.184631558 | -4.429213364 | 3.96E-05 | 0.000662296 | 1.948647903 |
| NDUFA9 | 4704 | -0.679459291 | -0.172429234 | -4.428855084 | 3.96E-05 | 0.000662848 | 1.947463121 |
| FLJ32658 | 147872 | 0.484255569 | -0.028849959 | 4.428312498 | 3.97E-05 | 0.00066383 | 1.945668958 |
| RHOQ | 23433 | -0.953684351 | -0.191337646 | -4.427670711 | 3.98E-05 | 0.000664961 | 1.943546909 |
| IKZF2 | 22807 | 0.326418239 | 0.0099164 | 4.425859708 | 4.00E-05 | 0.000668684 | 1.937559717 |
| ESRRAP2 | 144832 | -0.464749712 | -0.073769291 | -4.425753869 | 4.01E-05 | 0.000668684 | 1.937209851 |
| MAML1 | 9794 | 0.454718451 | -0.025271539 | 4.422746387 | 4.05E-05 | 0.000674364 | 1.927269922 |
| RPS4X | 6191 | -0.996865604 | 0.040364049 | -4.422740126 | 4.05E-05 | 0.000674364 | 1.927249231 |
| CLDN4 | 1364 | 0.310786754 | -0.128984005 | 4.422612421 | 4.05E-05 | 0.000674364 | 1.926827234 |
| TTLL1 | 25809 | 0.390711589 | -0.00710256 | 4.422527438 | 4.05E-05 | 0.000674364 | 1.926546413 |
| KLHL5 | 51088 | 0.568361818 | -0.10176626 | 4.42249952 | 4.05E-05 | 0.000674364 | 1.926454161 |
| LOC653766 | 653766 | 0.43881536 | -0.101338065 | 4.422335941 | 4.05E-05 | 0.000674364 | 1.925913634 |
| LOC648749 | 648749 | 0.323205479 | -0.001225746 | 4.422259368 | 4.06E-05 | 0.000674364 | 1.925660611 |
| HIPK2 | 28996 | -0.779024605 | -0.146856193 | -4.422021033 | 4.06E-05 | 0.000674644 | 1.924873087 |
| RAB11FIP5 | 26056 | -0.602061314 | -0.157357346 | -4.421591505 | 4.07E-05 | 0.000675376 | 1.923453863 |
| EI24 | 9538 | -0.930400411 | -0.129464424 | -4.420727976 | 4.08E-05 | 0.00067714 | 1.920600843 |
| C1orf19 | 116461 | -1.012190603 | -0.109248071 | -4.420442657 | 4.08E-05 | 0.000677532 | 1.919658237 |
| LOC441179 | 441179 | 0.323274315 | -0.07825708 | 4.418784031 | 4.11E-05 | 0.000681198 | 1.914179256 |
| LIMD2 | 80774 | 0.402366405 | -0.126387856 | 4.418358012 | 4.11E-05 | 0.000681692 | 1.912772142 |
| SNAPC3 | 6619 | -0.568969422 | -0.103123841 | -4.418186102 | 4.11E-05 | 0.000681692 | 1.912204354 |
| FAM58A | 92002 | -0.543604762 | -0.101845998 | -4.41808076 | 4.12E-05 | 0.000681692 | 1.911856433 |
| C9orf144 | 389715 | 0.347193977 | 0.009051655 | 4.417918926 | 4.12E-05 | 0.000681692 | 1.911321943 |
| RAB5C | 5878 | -1.082994744 | -0.265927695 | -4.41786877 | 4.12E-05 | 0.000681692 | 1.911156293 |
| PSMC3IP | 29893 | 0.437962121 | -0.029957081 | 4.417858222 | 4.12E-05 | 0.000681692 | 1.911121456 |
| FETUB | 26998 | 0.379346247 | -0.099792827 | 4.417523446 | 4.12E-05 | 0.000682206 | 1.910015822 |
| LOC147804 | 147804 | 0.514325065 | -0.139214468 | 4.416942869 | 4.13E-05 | 0.00068331 | 1.908098509 |
| LOC390683 | 390683 | 0.339261839 | -0.037891196 | 4.416789809 | 4.13E-05 | 0.00068339 | 1.90759306 |
| KRTAP15-1 | 254950 | 0.507558495 | -0.112525517 | 4.415754405 | 4.15E-05 | 0.000685588 | 1.904174083 |
| AP1M1 | 8907 | -0.657029255 | -0.17507853 | -4.415520246 | 4.15E-05 | 0.000685647 | 1.90340093 |
| BLVRA | 644 | -0.842838657 | -0.213130588 | -4.415490789 | 4.15E-05 | 0.000685647 | 1.903303671 |
| CIAO1 | 9391 | -0.713450085 | -0.24020694 | -4.415297278 | 4.16E-05 | 0.000685825 | 1.902664748 |
| UBFD1 | 56061 | -0.394465853 | -0.122951892 | -4.415145297 | 4.16E-05 | 0.000685903 | 1.902162955 |
| DPM3 | 54344 | -0.473391447 | -0.123209441 | -4.414142569 | 4.17E-05 | 0.000688031 | 1.898852494 |
| NEDD8 | 4738 | -0.964487787 | -0.267661865 | -4.413802996 | 4.18E-05 | 0.000688562 | 1.897731496 |
| ANKRD23 | 200539 | 0.514231383 | -0.12990528 | 4.413361515 | 4.18E-05 | 0.00068934 | 1.89627414 |
| LOC642751 | 642751 | 0.449950446 | -0.009815777 | 4.411568729 | 4.21E-05 | 0.000693395 | 1.890356808 |
| TMEM16A | 55107 | -1.060316909 | -0.263692386 | -4.411232609 | 4.22E-05 | 0.000693923 | 1.889247534 |
| SF3B14 | 51639 | -0.965561544 | -0.136129446 | -4.410533549 | 4.23E-05 | 0.000695194 | 1.88694061 |
| ALOX15B | 247 | -0.950683223 | -0.079941686 | -4.408887943 | 4.25E-05 | 0.000698481 | 1.881510775 |
| FBXO22 | 26263 | -0.404650623 | -0.133131301 | -4.408622 | 4.25E-05 | 0.00069884 | 1.880633369 |
| EMG1 | 10436 | -0.59332101 | -0.147794932 | -4.408106021 | 4.26E-05 | 0.000699104 | 1.878931108 |
| OPHN1 | 4983 | 0.371273754 | -0.097333233 | 4.407941636 | 4.26E-05 | 0.000699104 | 1.878388808 |
| CD3D | 915 | 0.415028641 | -0.136915791 | 4.407862667 | 4.27E-05 | 0.000699104 | 1.878128296 |
| H2AFB1 | 474382 | 0.357953664 | -0.058843461 | 4.40780157 | 4.27E-05 | 0.000699104 | 1.877926747 |
| ABLIM1 | 3983 | 0.390417166 | -0.034398771 | 4.407304276 | 4.27E-05 | 0.000700032 | 1.876286282 |
| C11orf1 | 64776 | -0.576838309 | -0.073797922 | -4.406852544 | 4.28E-05 | 0.000700849 | 1.874796198 |
| LOC647987 | 647987 | 0.415881375 | -0.04568533 | 4.406178546 | 4.29E-05 | 0.000702115 | 1.872573091 |
| EVI1 | 2122 | -1.122991587 | -0.083178039 | -4.406100244 | 4.29E-05 | 0.000702115 | 1.872314832 |
| KCNK17 | 89822 | -0.77179541 | -0.17747056 | -4.405902512 | 4.29E-05 | 0.00070231 | 1.871662675 |
| LOC646176 | 646176 | 0.436587703 | -0.107447466 | 4.40470183 | 4.31E-05 | 0.000704978 | 1.867702908 |
| LOC388849 | 388849 | 0.486147605 | -0.183549594 | 4.404517432 | 4.32E-05 | 0.000705141 | 1.867094825 |
| SUZ12 | 23512 | -0.986680613 | -0.332587182 | -4.404395115 | 4.32E-05 | 0.000705148 | 1.866691473 |
| PIN1 | 5300 | -0.630420173 | -0.150727167 | -4.404277711 | 4.32E-05 | 0.000705148 | 1.866304323 |
| DDRGK1 | 65992 | -0.680878879 | -0.147254454 | -4.403672473 | 4.33E-05 | 0.000706352 | 1.864308595 |
| RCOR3 | 55758 | -0.781131289 | -0.111375111 | -4.40298484 | 4.34E-05 | 0.000707762 | 1.862041346 |
| LOC730994 | 730994 | 0.679494909 | 0.122761137 | 4.402733232 | 4.34E-05 | 0.000707784 | 1.861211792 |
| TRAPPC2L | 51693 | -0.66094479 | -0.130596432 | -4.402726654 | 4.34E-05 | 0.000707784 | 1.861190105 |
| LOC645688 | 645688 | -0.770737942 | -0.094717817 | -4.402381568 | 4.35E-05 | 0.000707784 | 1.860052395 |
| MRVI1 | 10335 | -0.641716797 | -0.119268742 | -4.402337171 | 4.35E-05 | 0.000707784 | 1.859906026 |
| BANF1 | 8815 | 0.40638886 | -0.047076179 | 4.402285204 | 4.35E-05 | 0.000707784 | 1.859734701 |
| LOC649049 | 649049 | -0.821248323 | -0.200587197 | -4.40207431 | 4.35E-05 | 0.000707971 | 1.859039437 |
| PROS1 | 5627 | -0.840536537 | -0.221467854 | -4.401950983 | 4.35E-05 | 0.000707985 | 1.858632867 |
| LOC646630 | 646630 | -0.591711161 | -0.187235296 | -4.4007828 | 4.37E-05 | 0.000710593 | 1.85478202 |
| GLS | 2744 | -0.863426589 | -0.251106329 | -4.400440253 | 4.38E-05 | 0.000710939 | 1.853652934 |
| HCCA2 | 81532 | -0.516050484 | -0.054565888 | -4.400408482 | 4.38E-05 | 0.000710939 | 1.853548215 |
| C9orf130 | 286354 | 0.843261024 | -0.049591818 | 4.400119635 | 4.38E-05 | 0.000711277 | 1.85259617 |
| ZNF770 | 54989 | -0.661601354 | -0.103033053 | -4.4000376 | 4.38E-05 | 0.000711277 | 1.852325787 |
| LOC645895 | 645895 | 0.916586787 | -0.033753368 | 4.399657354 | 4.39E-05 | 0.00071169 | 1.851072546 |
| CSTF2T | 23283 | -0.679913187 | -0.125497632 | -4.399636613 | 4.39E-05 | 0.00071169 | 1.85100419 |
| LOC727805 | 727805 | 0.50476065 | -0.00259724 | 4.399087359 | 4.40E-05 | 0.000712684 | 1.849194024 |
| PPIE | 10450 | -0.724812013 | -0.21053265 | -4.399002899 | 4.40E-05 | 0.000712684 | 1.848915682 |
| NKX1-1 | 54729 | 0.426268023 | -0.161760582 | 4.398477965 | 4.41E-05 | 0.000713409 | 1.847185791 |
| FLJ38482 | 201931 | -0.750139483 | -0.179636626 | -4.398058275 | 4.41E-05 | 0.000713871 | 1.8458028 |
| SLC12A4 | 6560 | -0.444349992 | -0.07338922 | -4.397362458 | 4.43E-05 | 0.000714732 | 1.843510049 |
| LOC642918 | 642918 | 0.52368923 | -0.088458728 | 4.397152518 | 4.43E-05 | 0.000714964 | 1.84281832 |
| LOC641977 | 641977 | 0.595122844 | -0.049948206 | 4.396925044 | 4.43E-05 | 0.000715241 | 1.84206884 |
| TNNC2 | 7125 | -0.328720509 | -0.13699712 | -4.396326558 | 4.44E-05 | 0.000716447 | 1.840097045 |
| CPEB3 | 22849 | -0.665097693 | -0.178920553 | -4.396037441 | 4.45E-05 | 0.000716879 | 1.839144557 |
| LOC643094 | 643094 | 0.645885986 | -0.041483842 | 4.395632763 | 4.45E-05 | 0.000717308 | 1.837811409 |
| HMGA2 | 8091 | 0.371134798 | -0.04430519 | 4.394981947 | 4.46E-05 | 0.000718649 | 1.83566753 |
| ZFAND5 | 7763 | -0.735184362 | -0.204122637 | -4.39462171 | 4.47E-05 | 0.000719262 | 1.834480926 |
| LOC642194 | 642194 | 0.532844259 | -0.061645983 | 4.393711037 | 4.48E-05 | 0.000720788 | 1.83148144 |
| ASB2 | 51676 | -0.395486028 | -0.199669001 | -4.393665708 | 4.48E-05 | 0.000720788 | 1.831332146 |
| LOC203547 | 203547 | -0.948075406 | -0.214577948 | -4.393393247 | 4.49E-05 | 0.000721182 | 1.830434804 |
| DPY30 | 84661 | -0.761569863 | -0.232362855 | -4.393093791 | 4.49E-05 | 0.00072149 | 1.829448592 |
| YSK4 | 80122 | 0.531411285 | -0.16395221 | 4.393038167 | 4.49E-05 | 0.00072149 | 1.829265405 |
| LOC653515 | 653515 | 0.447023741 | -0.194397183 | 4.392402054 | 4.50E-05 | 0.000722337 | 1.827170584 |
| NDUFV2 | 4729 | -0.622272245 | -0.136143607 | -4.392353333 | 4.50E-05 | 0.000722337 | 1.827010145 |
| LOC222967 | 222967 | 0.543608431 | -0.134221711 | 4.391515229 | 4.52E-05 | 0.000722374 | 1.82425039 |
| CCNYL1 | 151195 | -0.41919204 | -0.11860078 | -4.391513741 | 4.52E-05 | 0.000722374 | 1.824245491 |
| C17orf85 | 55421 | -0.508342061 | -0.081805254 | -4.391419307 | 4.52E-05 | 0.000722374 | 1.823934549 |
| AIF1L | 83543 | -0.753233888 | -0.008028223 | -4.391358358 | 4.52E-05 | 0.000722374 | 1.823733864 |
| FAM98A | 25940 | -0.814562547 | -0.247451853 | -4.391331606 | 4.52E-05 | 0.000722374 | 1.82364578 |
| LCE1E | 353135 | 0.364059494 | -0.020521681 | 4.391307814 | 4.52E-05 | 0.000722374 | 1.823567443 |
| ZMAT2 | 153527 | -0.73776777 | -0.113368827 | -4.391293759 | 4.52E-05 | 0.000722374 | 1.823521165 |
| PELO | 53918 | -0.7397671 | -0.094138444 | -4.390684666 | 4.53E-05 | 0.000723621 | 1.821515729 |
| MED30 | 90390 | -0.746083618 | -0.136933787 | -4.389633575 | 4.55E-05 | 0.000725698 | 1.818055346 |
| TES | 26136 | -0.791742037 | -0.002484469 | -4.389431303 | 4.55E-05 | 0.000725791 | 1.817389479 |
| LOC651454 | 651454 | 0.51703446 | -0.138631206 | 4.389365508 | 4.55E-05 | 0.000725791 | 1.817172889 |
| MORC2 | 22880 | -0.503829916 | -0.133669099 | -4.388994226 | 4.56E-05 | 0.000725979 | 1.8159507 |
| ZNF747 | 65988 | 0.420286455 | -0.119843136 | 4.38893218 | 4.56E-05 | 0.000725979 | 1.815746459 |
| C3orf67 | 200844 | -0.228106189 | -0.093226675 | -4.388497595 | 4.56E-05 | 0.000726322 | 1.814315967 |
| SAE1 | 10055 | -0.716277677 | -0.200210069 | -4.388462938 | 4.57E-05 | 0.000726322 | 1.814201893 |
| DCAF6 | 55827 | -0.76208669 | -0.17660922 | -4.38791528 | 4.57E-05 | 0.00072742 | 1.812399313 |
| TXN | 7295 | -0.881242275 | -0.316144785 | -4.387589458 | 4.58E-05 | 0.000727956 | 1.811326946 |
| PECI | 10455 | -0.86705312 | -0.308117775 | -4.387414529 | 4.58E-05 | 0.000728108 | 1.810751227 |
| ARL5C | 390790 | 0.48166953 | -0.041470235 | 4.386351595 | 4.60E-05 | 0.000730229 | 1.807253184 |
| EXOC4 | 60412 | -0.544776839 | -0.122882354 | -4.386098458 | 4.60E-05 | 0.000730461 | 1.806420191 |
| CAMLG | 819 | -0.879578163 | -0.347527299 | -4.386030218 | 4.60E-05 | 0.000730461 | 1.806195639 |
| SLC16A12 | 387700 | 0.619607738 | -0.255515352 | 4.385815779 | 4.61E-05 | 0.000730461 | 1.80549001 |
| SLC38A2 | 54407 | -0.964330399 | -0.245671882 | -4.385800537 | 4.61E-05 | 0.000730461 | 1.805439859 |
| LOC732371 | 732371 | 0.360692061 | -0.013925608 | 4.385462397 | 4.61E-05 | 0.000731031 | 1.804327225 |
| GALNT10 | 55568 | 0.347660678 | -0.078776686 | 4.384230303 | 4.63E-05 | 0.00073389 | 1.800273444 |
| TGM1 | 7051 | 0.309841136 | -0.052667854 | 4.38385902 | 4.64E-05 | 0.000734519 | 1.799051978 |
| FUT10 | 84750 | 0.384798662 | 0.03970068 | 4.383755368 | 4.64E-05 | 0.000734519 | 1.798710987 |
| LOC730109 | 730109 | 0.689620574 | -0.049663683 | 4.382397583 | 4.66E-05 | 0.000737716 | 1.794244583 |
| SUZ12P | 440423 | 0.494435394 | -0.120307853 | 4.38217392 | 4.67E-05 | 0.000737997 | 1.793508916 |
| C16orf11 | 146325 | 0.475724387 | -0.06474807 | 4.381963244 | 4.67E-05 | 0.000738244 | 1.792815981 |
| EGR3 | 1960 | 0.311661784 | -0.033824257 | 4.381649793 | 4.68E-05 | 0.000738757 | 1.791785045 |
| CEP170 | 9859 | 0.42949843 | -0.068040233 | 4.381482552 | 4.68E-05 | 0.000738893 | 1.791235003 |
| LOC390372 | 390372 | 0.432472098 | 0.0391388 | 4.380736962 | 4.69E-05 | 0.000740523 | 1.788782962 |
| N6AMT2 | 221143 | -0.503771672 | -0.077257493 | -4.380044142 | 4.70E-05 | 0.000742021 | 1.786504658 |
| PRKY | 5616 | 0.566638018 | -0.006234447 | 4.379829062 | 4.70E-05 | 0.000742134 | 1.785797414 |
| BCL6 | 604 | -0.682483804 | -0.181354851 | -4.379756449 | 4.71E-05 | 0.000742134 | 1.785558646 |
| C3orf21 | 152002 | -0.652404594 | -0.082915233 | -4.379656744 | 4.71E-05 | 0.000742134 | 1.785230796 |
| NHP2L1 | 4809 | -0.918547357 | -0.277425735 | -4.37949626 | 4.71E-05 | 0.000742254 | 1.784703102 |
| LOC644827 | 644827 | 0.390071111 | -0.026906108 | 4.378153138 | 4.73E-05 | 0.000744945 | 1.780287115 |
| NDUFA7 | 4701 | -0.746076092 | -0.216800735 | -4.37811711 | 4.73E-05 | 0.000744945 | 1.78016867 |
| C14orf112 | 51241 | -0.743181393 | -0.206263234 | -4.377786106 | 4.74E-05 | 0.000745509 | 1.77908049 |
| C1orf101 | 257044 | 0.269946264 | -0.064176117 | 4.37662772 | 4.76E-05 | 0.000748232 | 1.775272603 |
| PFKP | 5214 | -0.810381937 | 0.067785505 | -4.376416578 | 4.76E-05 | 0.000748486 | 1.774578586 |
| KIAA0141 | 9812 | -0.593890402 | -0.126242556 | -4.375810611 | 4.77E-05 | 0.000749772 | 1.772586884 |
| THUMPD1 | 55623 | -0.926348153 | -0.153267865 | -4.375443174 | 4.78E-05 | 0.000749838 | 1.771379256 |
| CHCHD1 | 118487 | -0.479609758 | -0.138708864 | -4.37471058 | 4.79E-05 | 0.000751161 | 1.768971648 |
| LOC646654 | 646654 | 0.544558957 | -0.141585993 | 4.374240648 | 4.80E-05 | 0.000751974 | 1.767427364 |
| LOC652968 | 652968 | -0.34049043 | -0.096402184 | -4.374172893 | 4.80E-05 | 0.000751974 | 1.767204713 |
| LOC728032 | 728032 | 0.532240007 | -0.086056309 | 4.373251089 | 4.81E-05 | 0.000754098 | 1.764175765 |
| MRPL14 | 64928 | -0.634659712 | -0.126874033 | -4.372932374 | 4.82E-05 | 0.000754638 | 1.763128576 |
| CLPP | 8192 | -0.476855417 | -0.073275058 | -4.372649216 | 4.82E-05 | 0.000755084 | 1.762198249 |
| LOC646515 | 646515 | 0.436504063 | -0.055045619 | 4.371721394 | 4.84E-05 | 0.000756933 | 1.759150071 |
| EIF3E | 3646 | -0.669147295 | -0.154974562 | -4.370710852 | 4.86E-05 | 0.000758404 | 1.755830505 |
| MAGEE1 | 57692 | -0.635600469 | -0.126730714 | -4.369981366 | 4.87E-05 | 0.000759618 | 1.753434435 |
| C19orf56 | 51398 | -0.682556712 | -0.159975858 | -4.369625426 | 4.88E-05 | 0.000760078 | 1.752265389 |
| LOC347549 | 347549 | 0.37183442 | -0.004048183 | 4.36879666 | 4.89E-05 | 0.000761978 | 1.749543585 |
| C6orf117 | 112609 | -1.379571631 | -0.196238771 | -4.368333899 | 4.90E-05 | 0.000762908 | 1.748023918 |
| SYT11 | 23208 | -0.935876424 | -0.218036804 | -4.367709316 | 4.91E-05 | 0.000763706 | 1.745972967 |
| MTA2 | 9219 | -0.651447721 | -0.165831795 | -4.367623361 | 4.91E-05 | 0.000763706 | 1.745690729 |
| ARHGEF7 | 8874 | -0.689278686 | -0.10745173 | -4.367477764 | 4.91E-05 | 0.000763706 | 1.745212658 |
| ZC3HAV1 | 56829 | 0.562956414 | 0.101888788 | 4.367355469 | 4.91E-05 | 0.000763706 | 1.744811106 |
| LOC728498 | 728498 | 0.408012454 | -0.094847634 | 4.367008075 | 4.92E-05 | 0.000764331 | 1.743670476 |
| DUSP26 | 78986 | -0.728379376 | -0.086713149 | -4.366563145 | 4.93E-05 | 0.000765159 | 1.74220967 |
| METTL7A | 25840 | -1.047125314 | -0.291811504 | -4.366472041 | 4.93E-05 | 0.000765159 | 1.741910564 |
| LOC729744 | 729744 | 0.338393101 | -0.061550655 | 4.366209248 | 4.93E-05 | 0.000765559 | 1.741047798 |
| PPP1R3F | 89801 | -0.405963349 | -0.071009444 | -4.365826113 | 4.94E-05 | 0.000766282 | 1.739789989 |
| RBM9 | 23543 | -0.29153764 | -0.126252143 | -4.365455238 | 4.95E-05 | 0.000766972 | 1.738572486 |
| TCEB1 | 6921 | -0.605424309 | -0.086761574 | -4.364986075 | 4.96E-05 | 0.000767926 | 1.737032399 |
| LOC648570 | 648570 | 0.347020809 | -0.091976817 | 4.364840137 | 4.96E-05 | 0.000768015 | 1.736553356 |
| USP41 | 373856 | 0.571806074 | -0.030440002 | 4.364540663 | 4.96E-05 | 0.000768516 | 1.735570355 |
| FOSB | 2354 | 2.03025461 | -0.041848108 | 4.36360832 | 4.98E-05 | 0.000770716 | 1.73251023 |
| ATP9A | 10079 | -0.892642438 | -0.2505002 | -4.363414105 | 4.98E-05 | 0.000770936 | 1.731872823 |
| TTC37 | 9652 | -0.863706682 | -0.19210143 | -4.362102226 | 5.01E-05 | 0.00077356 | 1.727567656 |
| LOC650822 | 650822 | 0.489184997 | -0.123898239 | 4.3613902 | 5.02E-05 | 0.000775032 | 1.725231293 |
| CISD1 | 55847 | -0.777622221 | -0.112421103 | -4.361195821 | 5.02E-05 | 0.000775032 | 1.724593511 |
| IL17RC | 84818 | 0.441207326 | -0.107117453 | 4.361181636 | 5.02E-05 | 0.000775032 | 1.724546969 |
| GNB1L | 54584 | -0.343421073 | -0.102534101 | -4.361107633 | 5.02E-05 | 0.000775032 | 1.724304161 |
| KCNK3 | 3777 | -0.498647074 | -0.162392533 | -4.360841128 | 5.03E-05 | 0.000775146 | 1.723429761 |
| FLJ39061 | 165057 | 0.40051913 | -0.153335014 | 4.360391297 | 5.04E-05 | 0.000775638 | 1.721953933 |
| LOC727735 | 727735 | 0.407218404 | -0.017559681 | 4.360378332 | 5.04E-05 | 0.000775638 | 1.721911395 |
| GLTPD2 | 388323 | 0.428305456 | -0.079448901 | 4.360210121 | 5.04E-05 | 0.000775638 | 1.721359543 |
| EPPK1 | 83481 | 0.408743529 | -0.110076107 | 4.360126918 | 5.04E-05 | 0.000775638 | 1.72108658 |
| HMGB1 | 3146 | -0.738031613 | -0.167287322 | -4.360004741 | 5.04E-05 | 0.000775638 | 1.72068576 |
| RAB3GAP1 | 22930 | -1.03038653 | -0.323031986 | -4.359875022 | 5.04E-05 | 0.000775638 | 1.720260204 |
| SLC30A5 | 64924 | -0.63808607 | -0.09377936 | -4.359121569 | 5.06E-05 | 0.000776818 | 1.717788563 |
| CCDC126 | 90693 | -0.433080197 | -0.12999353 | -4.359052575 | 5.06E-05 | 0.000776818 | 1.717562244 |
| TTLL7 | 79739 | -0.546966641 | -0.232491984 | -4.358998339 | 5.06E-05 | 0.000776818 | 1.717384335 |
| CSE1L | 1434 | -0.748040085 | -0.197066769 | -4.358991595 | 5.06E-05 | 0.000776818 | 1.717362213 |
| TMEM80 | 283232 | 0.452471488 | -0.121400035 | 4.358674 | 5.07E-05 | 0.000777376 | 1.716320452 |
| RPS21 | 6227 | -1.140713695 | -0.405727031 | -4.35855329 | 5.07E-05 | 0.000777401 | 1.715924513 |
| TUBG1 | 7283 | -0.502937178 | -0.070161742 | -4.358228751 | 5.07E-05 | 0.000777978 | 1.714860025 |
| LOC650454 | 650454 | 0.518588832 | -0.052676743 | 4.357191995 | 5.09E-05 | 0.000780491 | 1.711459744 |
| PCMT1 | 5110 | -0.87434682 | -0.16212003 | -4.356699173 | 5.10E-05 | 0.000781495 | 1.709843568 |
| LOC642350 | 642350 | 0.462565247 | -0.130000518 | 4.356512036 | 5.10E-05 | 0.000781495 | 1.709229887 |
| ZNF416 | 55659 | -0.380723299 | -0.153998297 | -4.356488448 | 5.10E-05 | 0.000781495 | 1.709152536 |
| C12orf29 | 91298 | -0.665804881 | -0.074040694 | -4.356237192 | 5.11E-05 | 0.000781876 | 1.708328616 |
| PRDX6 | 9588 | -0.858637409 | -0.218487694 | -4.355804384 | 5.12E-05 | 0.000782703 | 1.706909404 |
| KIAA0748 | 9840 | 0.452475011 | 0.011420095 | 4.355707579 | 5.12E-05 | 0.000782703 | 1.706591983 |
| COX7A1 | 1346 | -1.070119081 | -0.254130287 | -4.355578354 | 5.12E-05 | 0.000782703 | 1.706168265 |
| SMC2 | 10592 | -0.537678173 | -0.144555808 | -4.35548878 | 5.12E-05 | 0.000782703 | 1.705874562 |
| IL2RG | 3561 | 0.767196848 | 0.107869724 | 4.355159299 | 5.13E-05 | 0.00078318 | 1.704794256 |
| LOC728006 | 728006 | -0.962966515 | -0.282347725 | -4.355040025 | 5.13E-05 | 0.00078318 | 1.704403187 |
| ABCF1 | 23 | -0.584937386 | -0.030111559 | -4.354980368 | 5.13E-05 | 0.00078318 | 1.704207592 |
| LOC388931 | 388931 | 0.419258261 | -0.0419097 | 4.354846329 | 5.13E-05 | 0.000783242 | 1.703768126 |
| IMP4 | 92856 | -0.660086277 | -0.125153023 | -4.354650019 | 5.14E-05 | 0.000783475 | 1.703124508 |
| RPN1 | 6184 | -0.669581922 | -0.107805742 | -4.354350013 | 5.14E-05 | 0.000783991 | 1.702140939 |
| ERBB2 | 2064 | -0.872948417 | -0.209378734 | -4.352859753 | 5.17E-05 | 0.000787463 | 1.697255654 |
| PFDN6 | 10471 | -0.402973564 | -0.088174539 | -4.352693536 | 5.17E-05 | 0.000787549 | 1.696710824 |
| LOC651728 | 651728 | 0.409816698 | -0.076284357 | 4.352606937 | 5.17E-05 | 0.000787549 | 1.696426973 |
| RHOF | 54509 | 0.404986387 | -0.042293998 | 4.352284828 | 5.18E-05 | 0.000788129 | 1.695371197 |
| TIPRL | 261726 | -0.74421567 | -0.147312422 | -4.351848599 | 5.19E-05 | 0.000788879 | 1.693941438 |
| B3GALNT1 | 8706 | -0.682079684 | -0.119611925 | -4.35170621 | 5.19E-05 | 0.000788879 | 1.693474767 |
| LOC653972 | 653972 | -0.380082306 | -0.149200671 | -4.351679583 | 5.19E-05 | 0.000788879 | 1.6933875 |
| HOPX | 84525 | 0.341570943 | -0.008910582 | 4.350326951 | 5.21E-05 | 0.000791993 | 1.68895474 |
| GNPAT | 8443 | -0.673205924 | -0.130000783 | -4.34966686 | 5.23E-05 | 0.000793205 | 1.686791792 |
| IL6R | 3570 | 0.390316454 | -0.09705946 | 4.349047568 | 5.24E-05 | 0.000794367 | 1.684762683 |
| SLC7A5P1 | 81893 | 0.475369487 | -0.022184235 | 4.34898005 | 5.24E-05 | 0.000794367 | 1.68454147 |
| ATP7B | 540 | 0.319856296 | -0.12168303 | 4.348914957 | 5.24E-05 | 0.000794367 | 1.684328204 |
| OR5K4 | 403278 | 0.354011669 | -0.076176184 | 4.348468935 | 5.25E-05 | 0.000794966 | 1.682866929 |
| LRRC59 | 55379 | 0.391931767 | -0.070849415 | 4.348458976 | 5.25E-05 | 0.000794966 | 1.682834301 |
| LOC652260 | 652260 | 0.343219787 | -0.061778748 | 4.348367512 | 5.25E-05 | 0.000794966 | 1.682534655 |
| SKIL | 6498 | 0.407681919 | -0.040135437 | 4.348202195 | 5.25E-05 | 0.000795119 | 1.681993061 |
| MKNK2 | 2872 | -0.739318841 | -0.124038592 | -4.34806397 | 5.26E-05 | 0.000795196 | 1.681540234 |
| ITGAX | 3687 | 0.617173064 | -0.093059754 | 4.346807085 | 5.28E-05 | 0.000797841 | 1.677422992 |
| LAMA5 | 3911 | -0.847918734 | -0.157078967 | -4.34677994 | 5.28E-05 | 0.000797841 | 1.677334077 |
| RUSC2 | 9853 | -0.717857229 | -0.038027248 | -4.346491395 | 5.28E-05 | 0.000798108 | 1.676388968 |
| FSCN3 | 29999 | 0.408776741 | -0.115099844 | 4.346435215 | 5.29E-05 | 0.000798108 | 1.676204957 |
| BTF3 | 689 | -0.458049905 | -0.012893346 | -4.346078518 | 5.29E-05 | 0.000798567 | 1.675036668 |
| MRPS11 | 64963 | -0.601504983 | -0.127816642 | -4.34586289 | 5.30E-05 | 0.000798861 | 1.674330448 |
| PDE5A | 8654 | -1.287709968 | -0.418054654 | -4.345701399 | 5.30E-05 | 0.000799005 | 1.673801547 |
| EPB41 | 2035 | 0.459505212 | -0.065710273 | 4.345417133 | 5.30E-05 | 0.00079949 | 1.67287057 |
| RNF181 | 51255 | -0.803553199 | -0.189148526 | -4.345066439 | 5.31E-05 | 0.000800161 | 1.671722082 |
| OPN5 | 221391 | 0.427697427 | -0.187202578 | 4.344339192 | 5.32E-05 | 0.000801882 | 1.669340575 |
| ASNA1 | 439 | -0.345243229 | -0.23737493 | -4.343838719 | 5.33E-05 | 0.000802974 | 1.6677018 |
| ADO | 84890 | -0.856831971 | -0.286771447 | -4.34317553 | 5.35E-05 | 0.000804522 | 1.66553037 |
| LOC650038 | 650038 | 0.467311122 | -0.075519319 | 4.342542026 | 5.36E-05 | 0.00080599 | 1.663456295 |
| LOC642960 | 642960 | 0.380460721 | -0.103265672 | 4.34187119 | 5.37E-05 | 0.000807566 | 1.661260168 |
| FLJ46020 | 400863 | 0.397086756 | -0.035623916 | 4.341736201 | 5.37E-05 | 0.000807637 | 1.660818273 |
| LOC389523 | 389523 | 0.344880102 | 0.001465671 | 4.341582526 | 5.38E-05 | 0.000807761 | 1.660315217 |
| LOC644262 | 644262 | 0.335306177 | -0.114526236 | 4.34107658 | 5.39E-05 | 0.000808754 | 1.658659065 |
| GSTO1 | 9446 | -1.039401963 | -0.427813852 | -4.341010668 | 5.39E-05 | 0.000808754 | 1.658443317 |
| FAM13A | 10144 | 0.400744354 | -0.034590691 | 4.340553239 | 5.40E-05 | 0.000809734 | 1.656946078 |
| LPAL2 | 80350 | 0.435600209 | -0.016625578 | 4.339978581 | 5.41E-05 | 0.000810737 | 1.655065244 |
| ATP1B3 | 483 | -0.979285594 | -0.232297305 | -4.339791977 | 5.41E-05 | 0.000810955 | 1.654454523 |
| EIF3F | 8665 | -0.66570266 | -0.164323611 | -4.339561261 | 5.41E-05 | 0.000811098 | 1.653699453 |
| LRRC44 | 127255 | 0.479788899 | -0.066928099 | 4.33952289 | 5.41E-05 | 0.000811098 | 1.653573877 |
| LOC653450 | 653450 | 0.55690536 | -0.098331292 | 4.339262666 | 5.42E-05 | 0.000811328 | 1.652722262 |
| LOC650274 | 650274 | 0.383586279 | -0.069188147 | 4.339223041 | 5.42E-05 | 0.000811328 | 1.652592585 |
| COPS6 | 10980 | -0.504409431 | -0.082660613 | -4.338770136 | 5.43E-05 | 0.000812299 | 1.651110467 |
| ADAM17 | 6868 | 0.308481118 | -0.036940638 | 4.337971659 | 5.44E-05 | 0.000814182 | 1.648497673 |
| SORBS2 | 8470 | -1.278167927 | -0.28656513 | -4.337886395 | 5.45E-05 | 0.000814182 | 1.648218683 |
| LOC649946 | 649946 | -0.999117437 | -0.334423861 | -4.337648898 | 5.45E-05 | 0.00081447 | 1.647441597 |
| ZFP1 | 162239 | -0.401010638 | -0.10946402 | -4.337566518 | 5.45E-05 | 0.00081447 | 1.647172056 |
| LOC731884 | 731884 | 0.412903762 | -0.048527052 | 4.337169252 | 5.46E-05 | 0.000814979 | 1.645872267 |
| PSMG1 | 8624 | -0.616831667 | -0.090587638 | -4.336800034 | 5.47E-05 | 0.000815148 | 1.644664304 |
| PEG3 | 5178 | -0.75142305 | -0.064277751 | -4.336783102 | 5.47E-05 | 0.000815148 | 1.644608908 |
| TCEA3 | 6920 | -0.908887075 | -0.093838095 | -4.336285642 | 5.48E-05 | 0.000816251 | 1.642981463 |
| LOC643008 | 643008 | 0.512323429 | -0.147316414 | 4.335416715 | 5.49E-05 | 0.000818103 | 1.640138993 |
| SHKBP1 | 92799 | 0.729411303 | 0.133828448 | 4.335236155 | 5.50E-05 | 0.000818308 | 1.639548375 |
| AMY1B | 277 | 0.338677948 | -0.044655522 | 4.334899402 | 5.50E-05 | 0.000818957 | 1.638446879 |
| PHF21B | 112885 | 0.407378488 | -0.071029844 | 4.334060266 | 5.52E-05 | 0.000821035 | 1.635702311 |
| PFKM | 5213 | -0.687551458 | -0.107039267 | -4.333953458 | 5.52E-05 | 0.000821035 | 1.635352992 |
| FAM134C | 162427 | -0.437657379 | -0.172953321 | -4.333769638 | 5.52E-05 | 0.00082125 | 1.634751815 |
| ACTR1B | 10120 | -0.541060954 | -0.110317229 | -4.333270963 | 5.53E-05 | 0.000821746 | 1.633120983 |
| LOC644852 | 644852 | 0.567481398 | 0.011699303 | 4.33237442 | 5.55E-05 | 0.000823932 | 1.630189234 |
| POLR2H | 5437 | -0.80086818 | -0.17342494 | -4.3322902 | 5.55E-05 | 0.000823932 | 1.629913847 |
| KHNYN | 23351 | 0.668863103 | 0.165893361 | 4.331938541 | 5.56E-05 | 0.000824378 | 1.628763997 |
| CLEC2D | 29121 | 0.332795095 | -0.007774843 | 4.331917998 | 5.56E-05 | 0.000824378 | 1.628696829 |
| NUBP2 | 10101 | -0.445257453 | -0.16227039 | -4.330393291 | 5.59E-05 | 0.000828447 | 1.623711942 |
| Septin 5 | 5413 | 0.426666487 | -0.029825624 | 4.330249348 | 5.59E-05 | 0.00082855 | 1.623241381 |
| BCL2L13 | 23786 | -0.676432539 | -0.176727402 | -4.329728595 | 5.60E-05 | 0.00082974 | 1.621539064 |
| C22orf13 | 83606 | -0.536627949 | -0.119434967 | -4.32941731 | 5.61E-05 | 0.000830233 | 1.62052154 |
| PLK4 | 10733 | 0.454918879 | -0.025705781 | 4.329341269 | 5.61E-05 | 0.000830233 | 1.620272984 |
| LOC652683 | 652683 | 0.459633791 | -0.083518919 | 4.329143695 | 5.61E-05 | 0.00083026 | 1.619627181 |
| YIF1A | 10897 | -0.728184489 | -0.165267383 | -4.329107646 | 5.61E-05 | 0.00083026 | 1.619509351 |
| SURF1 | 6834 | -0.546928456 | -0.059563494 | -4.329002083 | 5.62E-05 | 0.00083026 | 1.61916431 |
| COX7A2 | 1347 | -1.119398237 | -0.460074832 | -4.328900382 | 5.62E-05 | 0.00083026 | 1.618831895 |
| AKAP8 | 10270 | -0.597620771 | -0.060065059 | -4.32836053 | 5.63E-05 | 0.000831507 | 1.617067431 |
| SERF1A | 8293 | -0.289854723 | -0.12790984 | -4.328151138 | 5.63E-05 | 0.000831801 | 1.616383083 |
| MTHFD1 | 4522 | -0.366538929 | -0.066867002 | -4.326955256 | 5.66E-05 | 0.000834569 | 1.612474944 |
| SIVA | 10572 | -0.507692297 | -0.121982212 | -4.326787791 | 5.66E-05 | 0.000834569 | 1.611927712 |
| LOC440277 | 440277 | 0.382087487 | -0.065428522 | 4.326764255 | 5.66E-05 | 0.000834569 | 1.611850804 |
| PDXP | 57026 | -0.673717352 | -0.185942275 | -4.326452828 | 5.67E-05 | 0.00083516 | 1.610833176 |
| C10orf84 | 63877 | -0.353905319 | -0.126293446 | -4.326324528 | 5.67E-05 | 0.00083522 | 1.610413951 |
| TRAK2 | 66008 | -0.852492135 | -0.173075221 | -4.326047243 | 5.67E-05 | 0.000835713 | 1.609507933 |
| TTC33 | 23548 | -0.541247663 | -0.147809777 | -4.325030208 | 5.69E-05 | 0.000838357 | 1.606185068 |
| PADI4 | 23569 | 1.096481865 | 0.322469839 | 4.324445657 | 5.71E-05 | 0.000839748 | 1.604275403 |
| NANOS3 | 342977 | 0.399401163 | -0.124160883 | 4.323112229 | 5.73E-05 | 0.000843014 | 1.599919736 |
| LOC338799 | 338799 | 0.463866804 | 0.045410381 | 4.323005775 | 5.73E-05 | 0.000843014 | 1.599572035 |
| SRP14 | 6727 | -0.943331018 | -0.060859601 | -4.322681327 | 5.74E-05 | 0.00084365 | 1.598512338 |
| ZNF684 | 127396 | -0.527512044 | -0.172045344 | -4.322479982 | 5.75E-05 | 0.000843925 | 1.597854736 |
| MIS12 | 79003 | -0.731387541 | -0.225094069 | -4.322164385 | 5.75E-05 | 0.000844003 | 1.596824011 |
| ZCCHC17 | 51538 | -0.673006776 | -0.144336572 | -4.322153765 | 5.75E-05 | 0.000844003 | 1.596789329 |
| TMEM150B | 284417 | 0.375497579 | -0.024917571 | 4.322131953 | 5.75E-05 | 0.000844003 | 1.596718095 |
| SLC35A2 | 7355 | -0.444106557 | -0.053434929 | -4.321668291 | 5.76E-05 | 0.000845048 | 1.595203875 |
| METTL13 | 51603 | -0.573405527 | -0.163636173 | -4.320603511 | 5.78E-05 | 0.000847863 | 1.591726858 |
| TTC27 | 55622 | -0.755507912 | -0.174939373 | -4.320335847 | 5.79E-05 | 0.000848018 | 1.590852875 |
| DPH2 | 1802 | -0.647692277 | -0.126242258 | -4.320208129 | 5.79E-05 | 0.000848018 | 1.59043586 |
| LOC651029 | 651029 | 0.331412982 | -0.031044001 | 4.319976807 | 5.80E-05 | 0.000848133 | 1.589680577 |
| TFE3 | 7030 | -0.413289868 | -0.082327855 | -4.319101623 | 5.81E-05 | 0.000849885 | 1.586823231 |
| LOC653683 | 653683 | 0.4497834 | -0.031094236 | 4.318757958 | 5.82E-05 | 0.000850467 | 1.585701295 |
| NICN1 | 84276 | -0.660252903 | -0.141960772 | -4.318171496 | 5.83E-05 | 0.000851475 | 1.583786834 |
| LTC4S | 4056 | 0.389234381 | -0.128181508 | 4.318087836 | 5.83E-05 | 0.000851475 | 1.583513741 |
| KIAA1191 | 57179 | -0.821894672 | -0.023899033 | -4.318001466 | 5.84E-05 | 0.000851475 | 1.583231808 |
| DDA1 | 79016 | -0.559354926 | -0.070789931 | -4.317989935 | 5.84E-05 | 0.000851475 | 1.583194166 |
| HUS1B | 135458 | 0.36654132 | -0.057996508 | 4.317586679 | 5.84E-05 | 0.000851916 | 1.581877873 |
| PEF1 | 553115 | -0.537779862 | -0.090699918 | -4.317564816 | 5.84E-05 | 0.000851916 | 1.581806512 |
| NT5C3L | 115024 | -0.649161679 | -0.112494122 | -4.317520967 | 5.84E-05 | 0.000851916 | 1.581663385 |
| GUSBL2 | 375513 | -0.461432926 | -0.178643936 | -4.317038436 | 5.85E-05 | 0.000853029 | 1.58008842 |
| LOC442015 | 442015 | 0.438263472 | -0.147325592 | 4.31662336 | 5.86E-05 | 0.000853943 | 1.5787337 |
| MEIS3P1 | 4213 | -0.628454965 | 0.016109144 | -4.315821821 | 5.88E-05 | 0.000855689 | 1.576117842 |
| TSPAN2 | 10100 | -0.483916272 | -0.143486557 | -4.315636561 | 5.88E-05 | 0.000855736 | 1.575513271 |
| C1orf189 | 388701 | -0.481009772 | -0.282208703 | -4.315434263 | 5.89E-05 | 0.000855893 | 1.574853118 |
| TCEAL3 | 85012 | -0.894794728 | -0.126207663 | -4.314612656 | 5.90E-05 | 0.000858021 | 1.57217215 |
| SLC15A4 | 121260 | -0.774498582 | -0.115803426 | -4.314376875 | 5.91E-05 | 0.000858295 | 1.571402828 |
| ZFAND1 | 79752 | -0.640993785 | -0.032639595 | -4.314308049 | 5.91E-05 | 0.000858295 | 1.571178262 |
| RABEPK | 10244 | -0.752621543 | -0.272752656 | -4.313881657 | 5.92E-05 | 0.000858767 | 1.569787069 |
| PLCB1 | 23236 | 0.532086513 | -0.063910666 | 4.31385818 | 5.92E-05 | 0.000858767 | 1.569710474 |
| LOC643373 | 643373 | 0.34360919 | 0.019797093 | 4.313831437 | 5.92E-05 | 0.000858767 | 1.569623221 |
| PLCE1 | 51196 | -0.483269002 | -0.089694254 | -4.313419905 | 5.93E-05 | 0.000859454 | 1.568280586 |
| PTPLA | 9200 | -1.007081924 | -0.110879639 | -4.311518586 | 5.97E-05 | 0.000864651 | 1.562078354 |
| C20orf177 | 63939 | -1.089645849 | -0.198350339 | -4.311439222 | 5.97E-05 | 0.000864651 | 1.561819494 |
| COMMD3 | 23412 | -1.01797914 | -0.243233248 | -4.310932344 | 5.98E-05 | 0.000865537 | 1.560166279 |
| UGT2B7 | 7364 | 0.393930071 | -0.025035607 | 4.308574815 | 6.03E-05 | 0.000872288 | 1.552478385 |
| LOC653057 | 653057 | 0.607777071 | -0.190612182 | 4.308480371 | 6.03E-05 | 0.000872288 | 1.552170447 |
| ZNF74 | 7625 | 0.263434501 | -0.058738513 | 4.308132737 | 6.04E-05 | 0.00087302 | 1.551037013 |
| LEMD3 | 23592 | -0.767848846 | -0.178526312 | -4.307802515 | 6.05E-05 | 0.0008737 | 1.549960392 |
| NCF2 | 4688 | 1.087860027 | 0.129358497 | 4.307211038 | 6.06E-05 | 0.000874567 | 1.548032112 |
| PSMF1 | 9491 | -0.716039091 | -0.164882267 | -4.307201234 | 6.06E-05 | 0.000874567 | 1.548000153 |
| KIAA1671 | 85379 | -0.883178835 | -0.26496327 | -4.307199389 | 6.06E-05 | 0.000874567 | 1.547994139 |
| LOC653261 | 653261 | 0.478402966 | -0.164189235 | 4.30699643 | 6.06E-05 | 0.000874863 | 1.547332502 |
| SLC25A26 | 115286 | -0.393472284 | -0.224383814 | -4.30679848 | 6.07E-05 | 0.000875143 | 1.54668721 |
| LOC647415 | 647415 | 0.452176658 | -0.018290954 | 4.306674636 | 6.07E-05 | 0.000875199 | 1.546283504 |
| VPS26B | 112936 | -0.460412325 | -0.114273475 | -4.305745469 | 6.09E-05 | 0.000877381 | 1.543254792 |
| HIBADH | 11112 | -0.710896787 | -0.096927374 | -4.30525471 | 6.10E-05 | 0.000878554 | 1.541655254 |
| LOC642521 | 642521 | 0.53364395 | -0.100093716 | 4.305132892 | 6.10E-05 | 0.000878604 | 1.541258226 |
| ZNF275 | 10838 | -0.952117042 | -0.137488796 | -4.305006367 | 6.10E-05 | 0.000878668 | 1.540845862 |
| CHRNA2 | 1135 | 0.3693068 | 0.003996292 | 4.304571624 | 6.11E-05 | 0.000879672 | 1.53942902 |
| GOLPH3 | 64083 | -0.903177434 | -0.144510995 | -4.304348603 | 6.12E-05 | 0.00087972 | 1.538702216 |
| MRPL40 | 64976 | -0.69836955 | -0.160315982 | -4.304163879 | 6.12E-05 | 0.000879953 | 1.538100232 |
| B3GNT7 | 93010 | 0.483667977 | -0.076373888 | 4.30400342 | 6.13E-05 | 0.000880122 | 1.537577334 |
| GLRX5 | 51218 | -0.751606468 | -0.222608286 | -4.303530472 | 6.14E-05 | 0.000881244 | 1.536036172 |
| ERCC1 | 2067 | 0.365766927 | 0.018667795 | 4.303168601 | 6.14E-05 | 0.000881904 | 1.534857028 |
| LOC652326 | 652326 | 0.365023066 | 0.002308667 | 4.303087135 | 6.15E-05 | 0.000881904 | 1.53459158 |
| SGCD | 6444 | -0.843969488 | 0.01072729 | -4.30299946 | 6.15E-05 | 0.000881904 | 1.534305906 |
| KIF1B | 23095 | -0.704405246 | -0.136206941 | -4.302767382 | 6.15E-05 | 0.000882292 | 1.533549728 |
| LOC652530 | 652530 | 0.407619809 | -0.037658798 | 4.302523368 | 6.16E-05 | 0.000882718 | 1.532754686 |
| PSMC6 | 5706 | -0.783956816 | -0.19731404 | -4.302265304 | 6.16E-05 | 0.000883186 | 1.531913893 |
| LOC648133 | 648133 | 0.356586672 | 0.006781914 | 4.301791343 | 6.17E-05 | 0.000884317 | 1.530369753 |
| PRAMEF7 | 441871 | 0.403875001 | -0.124019113 | 4.301636495 | 6.18E-05 | 0.00088447 | 1.529865286 |
| LOC652150 | 652150 | 0.37583335 | -0.075412051 | 4.301446164 | 6.18E-05 | 0.000884732 | 1.529245237 |
| UBE2D4 | 51619 | -0.591255665 | -0.095278382 | -4.301104702 | 6.19E-05 | 0.000885207 | 1.528132875 |
| KIAA0367 | 23273 | -1.436430708 | -0.332015454 | -4.301081827 | 6.19E-05 | 0.000885207 | 1.528058359 |
| COL8A1 | 1295 | -1.289912646 | -0.312308544 | -4.300948742 | 6.19E-05 | 0.000885294 | 1.527624829 |
| IFT57 | 55081 | -0.566246083 | -0.133864958 | -4.300728183 | 6.20E-05 | 0.00088565 | 1.526906362 |
| C6orf153 | 88745 | -0.758370605 | -0.13395843 | -4.300297145 | 6.20E-05 | 0.000886652 | 1.525502326 |
| CHCHD5 | 84269 | -0.623094457 | -0.185469561 | -4.298777734 | 6.24E-05 | 0.000890722 | 1.520553673 |
| KIAA2026 | 158358 | 0.41558157 | 0.016469605 | 4.29876593 | 6.24E-05 | 0.000890722 | 1.520515231 |
| CCNL2 | 81669 | 0.326989683 | -0.133212441 | 4.297949846 | 6.26E-05 | 0.00089292 | 1.517857664 |
| SERPINB9 | 5272 | 0.552602687 | -0.091409441 | 4.297677232 | 6.26E-05 | 8.93E-04 | 1.516969959 |
| COMT | 1312 | 0.57866291 | -0.10523242 | 4.297101438 | 6.27E-05 | 0.000894577 | 1.515095114 |
| RNF14 | 9604 | -0.786481407 | -0.260150924 | -4.296813688 | 6.28E-05 | 0.000895145 | 1.51415822 |
| LOC652234 | 652234 | 0.49403282 | -0.046546279 | 4.296568223 | 6.29E-05 | 0.000895503 | 1.513359028 |
| LOC284215 | 284215 | 0.508594604 | -0.102623371 | 4.295376364 | 6.31E-05 | 0.000898313 | 1.509478883 |
| FLJ20972 | 80098 | 0.375738586 | -0.043832423 | 4.294488964 | 6.33E-05 | 0.000900752 | 1.506590284 |
| ATP6V0A2 | 23545 | -0.544138618 | -0.102736234 | -4.294271713 | 6.34E-05 | 0.00090078 | 1.505883151 |
| TMPRSS7 | 344805 | 0.338052267 | -0.077285966 | 4.293722109 | 6.35E-05 | 0.000902068 | 1.504094325 |
| SPIN4 | 139886 | -0.736074621 | -0.141204927 | -4.293634044 | 6.35E-05 | 0.000902068 | 1.503807707 |
| MRPL46 | 26589 | -0.585269328 | -0.131549059 | -4.293546897 | 6.35E-05 | 0.000902068 | 1.503524078 |
| CHCHD9 | 645345 | -0.637850967 | -0.138902437 | -4.293293362 | 6.36E-05 | 0.000902535 | 1.502698943 |
| SIRT5 | 23408 | -0.556724117 | -0.122090185 | -4.292978022 | 6.36E-05 | 0.00090287 | 1.501672697 |
| LOC651686 | 651686 | 0.468492027 | -0.08181362 | 4.292017734 | 6.39E-05 | 0.000905044 | 1.498547766 |
| OTUD4 | 54726 | 0.678643849 | 0.066565137 | 4.291878831 | 6.39E-05 | 0.000905044 | 1.498095783 |
| DEFB103B | 414325 | 0.369605929 | -0.076924649 | 4.291867935 | 6.39E-05 | 0.000905044 | 1.49806033 |
| SLC2A3 | 6515 | 1.340430509 | 0.436341316 | 4.291434601 | 6.40E-05 | 0.000905931 | 1.496650337 |
| MLL3 | 58508 | 0.308559697 | -0.005845629 | 4.291377533 | 6.40E-05 | 0.000905931 | 1.496464652 |
| ZNF124 | 7678 | 0.360698997 | -0.124045599 | 4.291116241 | 6.41E-05 | 0.000906425 | 1.495614496 |
| LBH | 81606 | 0.575807456 | 0.145394065 | 4.290810315 | 6.41E-05 | 0.00090706 | 1.494619152 |
| TMEM99 | 147184 | -0.620438724 | -0.09527082 | -4.290418956 | 6.42E-05 | 0.000907964 | 1.493345902 |
| RBM17 | 84991 | -0.686207052 | -0.137564464 | -4.289468527 | 6.44E-05 | 0.000910465 | 1.490254021 |
| EEF2 | 1938 | -0.608421505 | -0.152107662 | -4.289327239 | 6.44E-05 | 0.000910465 | 1.489794422 |
| GRPR | 2925 | 0.525101182 | -0.083285929 | 4.289313282 | 6.45E-05 | 0.000910465 | 1.489749022 |
| GOLGA6B | 55889 | 0.365331684 | -0.033479513 | 4.288624123 | 6.46E-05 | 0.000912312 | 1.487507354 |
| TDRD10 | 126668 | 0.485224455 | 0.061320962 | 4.287999624 | 6.47E-05 | 0.000913356 | 1.485476176 |
| LOC642855 | 642855 | 0.316218803 | -0.088755206 | 4.287984872 | 6.47E-05 | 0.000913356 | 1.4854282 |
| CPSF3 | 51692 | -0.798942845 | -0.140732698 | -4.287860083 | 6.48E-05 | 0.000913419 | 1.485022344 |
| F2RL1 | 2150 | 0.499538705 | -0.051760835 | 4.286974523 | 6.50E-05 | 0.000915894 | 1.482142382 |
| LOC644725 | 644725 | 0.301423897 | 0.019675211 | 4.286776504 | 6.50E-05 | 0.0009159 | 1.481498439 |
| LOC642574 | 642574 | 0.425083686 | -0.035627174 | 4.286727801 | 6.50E-05 | 0.0009159 | 1.481340063 |
| LOC642268 | 642268 | 0.46274264 | -0.044558774 | 4.286570642 | 6.51E-05 | 0.0009159 | 1.480829009 |
| ZNF296 | 162979 | 0.816675205 | 0.163438874 | 4.286555925 | 6.51E-05 | 0.0009159 | 1.480781154 |
| CR1 | 1378 | 0.438662018 | -0.013257982 | 4.286427624 | 6.51E-05 | 0.0009159 | 1.480363949 |
| LOC642769 | 642769 | 0.37555711 | -0.080556009 | 4.286353323 | 6.51E-05 | 0.0009159 | 1.480122342 |
| CHCHD2 | 51142 | -0.728887626 | -0.266418074 | -4.285970938 | 6.52E-05 | 0.000916786 | 1.478878965 |
| DNAJC19 | 131118 | -0.528179298 | -0.173902092 | -4.285761803 | 6.52E-05 | 0.000917122 | 1.478198959 |
| ESRRG | 2104 | 0.582957988 | -0.195104144 | 4.28533563 | 6.53E-05 | 0.000918148 | 1.476813304 |
| LOC644863 | 644863 | -1.143229373 | -0.14911768 | -4.285203179 | 6.54E-05 | 0.000918241 | 1.47638267 |
| MRPL28 | 10573 | -0.371117609 | -0.139857113 | -4.284318239 | 6.56E-05 | 0.000920401 | 1.473505662 |
| ZNF653 | 115950 | -0.433229562 | -0.092466474 | -4.283667383 | 6.57E-05 | 0.000921819 | 1.47138988 |
| TP53RK | 112858 | -0.568837639 | -0.126520924 | -4.283367114 | 6.58E-05 | 0.000922448 | 1.470413831 |
| ISOC2 | 79763 | -0.565562525 | -0.009071945 | -4.282820979 | 6.59E-05 | 0.000923535 | 1.468638667 |
| CDH26 | 60437 | 0.373340463 | -0.090275325 | 4.282494395 | 6.60E-05 | 0.000924251 | 1.467577193 |
| OR5AN1 | 390195 | 0.386802542 | -0.083144114 | 4.282007501 | 6.61E-05 | 0.00092543 | 1.465994752 |
| FAM189B | 10712 | -0.777895554 | -0.272611804 | -4.28192035 | 6.61E-05 | 0.00092543 | 1.465711512 |
| CCDC6 | 8030 | -0.787355252 | -0.216491911 | -4.281384857 | 6.62E-05 | 0.00092669 | 1.463971251 |
| LARP1B | 55132 | -0.698359344 | -0.134585942 | -4.281321468 | 6.63E-05 | 0.00092669 | 1.463765255 |
| SHC1 | 6464 | -0.691585009 | -0.199490922 | -4.280351294 | 6.65E-05 | 0.000929474 | 1.460612664 |
| ATF4 | 468 | -0.713096422 | -0.186910006 | -4.280009548 | 6.66E-05 | 0.000930243 | 1.459502248 |
| AATK | 9625 | 0.414329557 | -0.013571756 | 4.279528813 | 6.67E-05 | 0.000931461 | 1.4579403 |
| LOC391475 | 391475 | 0.428851079 | -0.031025433 | 4.279078627 | 6.68E-05 | 0.000932338 | 1.456477694 |
| ARL5A | 26225 | -0.782081465 | -0.250753271 | -4.279051635 | 6.68E-05 | 0.000932338 | 1.456390001 |
| MYO5B | 4645 | 0.495662847 | -0.076442182 | 4.278634571 | 6.69E-05 | 0.000933046 | 1.455035082 |
| PLD2 | 5338 | 0.511363736 | -0.021938666 | 4.278627259 | 6.69E-05 | 0.000933046 | 1.455011328 |
| MTFMT | 123263 | -0.385474236 | -0.066089439 | -4.278502982 | 6.69E-05 | 0.000933116 | 1.454607601 |
| ANKRD44 | 91526 | 0.34157339 | -0.038468136 | 4.277578054 | 6.71E-05 | 0.000935774 | 1.451603075 |
| FAM22A | 283008 | 0.510958526 | -0.12465799 | 4.276786781 | 6.73E-05 | 0.000937417 | 1.449032982 |
| NOTCH3 | 4854 | -0.841072549 | -0.235252213 | -4.276727963 | 6.73E-05 | 0.000937417 | 1.44884195 |
| TIGD7 | 91151 | -0.514619556 | -0.119927134 | -4.276573291 | 6.74E-05 | 0.000937417 | 1.448339601 |
| URM1 | 81605 | -0.551499669 | -0.14210459 | -4.276559124 | 6.74E-05 | 0.000937417 | 1.448293589 |
| GTPBP6 | 8225 | -0.524989067 | -0.060794337 | -4.276147621 | 6.75E-05 | 0.000937576 | 1.446957147 |
| NHLRC4 | 283948 | 0.325050242 | -0.036404605 | 4.276138947 | 6.75E-05 | 0.000937576 | 1.446928977 |
| LOC651694 | 651694 | 0.389005038 | -0.093959554 | 4.276060237 | 6.75E-05 | 0.000937576 | 1.446673358 |
| PM20D2 | 135293 | -0.61012101 | -0.121954823 | -4.275999911 | 6.75E-05 | 0.000937576 | 1.446477442 |
| FOXD4L2 | 100036519 | 0.373530712 | -0.018803907 | 4.275507847 | 6.76E-05 | 0.000938841 | 1.444879479 |
| LOC646503 | 646503 | 0.369908558 | -0.038164821 | 4.275403874 | 6.76E-05 | 0.000938848 | 1.444541839 |
| LOC651732 | 651732 | 0.508158978 | -0.042854971 | 4.274436815 | 6.79E-05 | 0.000940919 | 1.441401663 |
| FGFRL1 | 53834 | -0.660578098 | 0.013451746 | -4.274417931 | 6.79E-05 | 0.000940919 | 1.44134035 |
| LOC401056 | 401056 | 0.269872935 | -0.113116813 | 4.274361297 | 6.79E-05 | 0.000940919 | 1.441156462 |
| NUDT9 | 53343 | -0.785003327 | -0.134438632 | -4.274167984 | 6.79E-05 | 0.000941209 | 1.440528798 |
| AIM1L | 55057 | 0.418714003 | 0.016363602 | 4.272566476 | 6.83E-05 | 0.000945766 | 1.435329477 |
| INTS9 | 55756 | -0.630433423 | -0.113841996 | -4.272372125 | 6.83E-05 | 0.000946069 | 1.434698583 |
| C1QBP | 708 | -0.681904162 | -0.149938497 | -4.272268483 | 6.84E-05 | 0.000946076 | 1.43436215 |
| MYO1E | 4643 | 0.38019055 | -0.018037449 | 4.272161824 | 6.84E-05 | 0.000946092 | 1.43401593 |
| LOC646358 | 646358 | 0.487255088 | -0.047813066 | 4.271980829 | 6.84E-05 | 0.00094619 | 1.43342842 |
| SSB | 6741 | 0.429533633 | -0.107402389 | 4.271928713 | 6.84E-05 | 0.00094619 | 1.433259255 |
| ACLY | 47 | -0.628827686 | -0.042319629 | -4.271417902 | 6.86E-05 | 0.000947022 | 1.431601246 |
| LOC651150 | 651150 | 0.611929213 | -0.054155072 | 4.271370018 | 6.86E-05 | 0.000947022 | 1.431445827 |
| LOC649991 | 649991 | 0.327334507 | -0.096064151 | 4.27111714 | 6.86E-05 | 0.000947276 | 1.430625071 |
| ATP5I | 521 | -1.012012198 | -0.266179387 | -4.271067177 | 6.86E-05 | 0.000947276 | 1.43046291 |
| DHCR24 | 1718 | -0.687936322 | -0.054018073 | -4.27084602 | 6.87E-05 | 0.000947276 | 1.429745132 |
| WDR52 | 55779 | 0.369646607 | -0.045680092 | 4.27081873 | 6.87E-05 | 0.000947276 | 1.429656564 |
| CAMK1D | 57118 | 0.357923223 | -0.105979756 | 4.27078569 | 6.87E-05 | 0.000947276 | 1.429549332 |
| RPS15 | 6209 | -0.29315447 | -0.149570826 | -4.270084638 | 6.89E-05 | 0.000948909 | 1.427274182 |
| ZNF434 | 54925 | -0.54362742 | -0.127716644 | -4.269125759 | 6.91E-05 | 0.000951724 | 1.424162611 |
| PEA15 | 8682 | -0.656424976 | -0.222505524 | -4.268891419 | 6.92E-05 | 0.00095204 | 1.423402233 |
| EIF3B | 8662 | -0.504914407 | -0.044232052 | -4.268827437 | 6.92E-05 | 0.00095204 | 1.42319463 |
| H2AFZ | 3015 | -0.984237267 | -0.316073935 | -4.26772041 | 6.94E-05 | 0.000955353 | 1.419602914 |
| AHCTF1 | 25909 | -0.653140793 | -0.129061551 | -4.267598295 | 6.95E-05 | 0.000955422 | 1.419206745 |
| PIR | 8544 | -0.622835645 | -0.018953086 | -4.267415868 | 6.95E-05 | 0.000955691 | 1.418614921 |
| LOC653086 | 653086 | 0.463779907 | -0.098037322 | 4.265122833 | 7.01E-05 | 0.00096228 | 1.411177083 |
| SLC35F2 | 54733 | -0.640878935 | -0.070295211 | -4.264418333 | 7.02E-05 | 0.000964063 | 1.408892346 |
| LOC727762 | 727762 | 0.549288986 | 0.058478924 | 4.264384702 | 7.03E-05 | 0.000964063 | 1.408783283 |
| LBA1 | 9881 | 0.663189327 | 0.0495185 | 4.263928943 | 7.04E-05 | 0.000965245 | 1.40730534 |
| C18orf45 | 85019 | -0.494660329 | -0.190404811 | -4.263742611 | 7.04E-05 | 0.00096553 | 1.406701126 |
| AIMP2 | 7965 | -0.681849062 | -0.276801321 | -4.263173112 | 7.05E-05 | 0.000967093 | 1.404854505 |
| MRPL36 | 64979 | -0.651518818 | -0.196802884 | -4.262545536 | 7.07E-05 | 0.000968853 | 1.402819721 |
| MFN1 | 55669 | -0.372470581 | -0.084102916 | -4.262197192 | 7.08E-05 | 0.000969681 | 1.401690357 |
| DNAJC8 | 22826 | -0.606649214 | -0.139363268 | -4.261946488 | 7.08E-05 | 0.000970183 | 1.400877579 |
| LOC653673 | 653673 | 0.327102585 | -0.020997003 | 4.261594972 | 7.09E-05 | 0.000971024 | 1.399738018 |
| LOC400682 | 400682 | 0.428141257 | -0.131033695 | 4.26114268 | 7.10E-05 | 0.000972203 | 1.398271826 |
| LOC653188 | 653188 | 0.286518364 | -0.130124162 | 4.260856717 | 7.11E-05 | 0.000972519 | 1.397344865 |
| LOC648154 | 648154 | 0.540876787 | -0.098303851 | 4.260746802 | 7.11E-05 | 0.000972519 | 1.396988579 |
| C6orf125 | 84300 | -0.726050197 | -0.224502087 | -4.260291463 | 7.13E-05 | 0.00097371 | 1.395512667 |
| IL17RB | 55540 | 0.334437026 | -0.007567356 | 4.259830714 | 7.14E-05 | 0.000974921 | 1.394019305 |
| TMEM5 | 10329 | -0.69091927 | -0.157202026 | -4.259497966 | 7.14E-05 | 0.000975703 | 1.392940866 |
| TREML2 | 79865 | 0.347171664 | 0.015827883 | 4.259213654 | 7.15E-05 | 0.000976322 | 1.392019446 |
| ALDH18A1 | 5832 | -0.755068903 | -0.214772994 | -4.258228497 | 7.18E-05 | 0.000978968 | 1.388826923 |
| SCN2B | 6327 | 0.466962858 | -0.014962525 | 4.257955555 | 7.18E-05 | 0.000979551 | 1.387942488 |
| SNAI2 | 6591 | -1.005678576 | -0.023833764 | -4.257771723 | 7.19E-05 | 0.000979833 | 1.387346823 |
| C1orf41 | 51668 | -0.847468045 | -0.282545975 | -4.257443467 | 7.20E-05 | 0.000980535 | 1.386283217 |
| RPS6KL1 | 83694 | 0.303668309 | -0.092295654 | 4.257363868 | 7.20E-05 | 0.000980535 | 1.386025308 |
| POLR2G | 5436 | -0.717559906 | -0.176432684 | -4.257195465 | 7.20E-05 | 0.000980635 | 1.385479677 |
| ZNF516 | 9658 | 0.419207497 | -0.018894899 | 4.257133893 | 7.20E-05 | 0.000980635 | 1.385280183 |
| CWC15 | 51503 | -0.655475769 | -0.174508451 | -4.256692729 | 7.21E-05 | 0.000981567 | 1.38385086 |
| LOC643424 | 643424 | 0.394974863 | -0.083739886 | 4.256586029 | 7.22E-05 | 0.000981567 | 1.383505174 |
| ATXN3 | 4287 | -0.537204443 | -0.205802815 | -4.256558329 | 7.22E-05 | 0.000981567 | 1.383415434 |
| FLJ46154 | 196296 | 0.438152093 | -0.057017779 | 4.256056521 | 7.23E-05 | 0.000982928 | 1.381789751 |
| C10orf104 | 119504 | -0.787184341 | -0.106146129 | -4.255834057 | 7.24E-05 | 0.000983343 | 1.38106908 |
| LOC440337 | 440337 | 0.291987588 | -0.066346346 | 4.25566138 | 7.24E-05 | 0.00098359 | 1.380509706 |
| DYSF | 8291 | 0.776084382 | 0.127714783 | 4.255402253 | 7.25E-05 | 0.00098413 | 1.379670304 |
| LOC645228 | 645228 | 0.359736948 | -0.010308653 | 4.255140738 | 7.25E-05 | 0.000984678 | 1.3788232 |
| HYAL2 | 8692 | -0.759448051 | -0.031627417 | -4.255037018 | 7.26E-05 | 0.000984692 | 1.378487233 |
| PGBD1 | 84547 | -0.366085462 | -0.066019809 | -4.25487614 | 7.26E-05 | 0.000984899 | 1.377966134 |
| LOC653163 | 653163 | 0.392995738 | -0.022309016 | 4.254690683 | 7.26E-05 | 0.00098519 | 1.377365432 |
| LOC653750 | 653750 | 0.498017002 | -0.052478294 | 4.253597182 | 7.29E-05 | 0.000988571 | 1.37382383 |
| LOC650526 | 650526 | 0.351808982 | -0.044794082 | 4.251505128 | 7.34E-05 | 0.000995042 | 1.36704949 |
| FLJ46363 | 400002 | 0.359189031 | -0.011358014 | 4.2512911 | 7.35E-05 | 0.000995434 | 1.366356538 |
| SEPHS1 | 22929 | -0.731879822 | -0.138035527 | -4.250037501 | 7.38E-05 | 0.000999401 | 1.362298181 |
| LOC285444 | 285444 | 0.406748094 | -0.068245907 | 4.249882998 | 7.39E-05 | 0.00099959 | 1.361798042 |
