# Supplementary material for: Establishment of a Combined Diagnostic Model of Abdominal Aortic Aneurysm with Random Forest and Artificial Neural Network
Source: Biomed Res Int. 2022 Mar 7;2022:7173972. doi: 10.1155/2022/7173972 (PMC8922147; doi:10.1155/2022/7173972)
Supplement: Supplementary 2 — Supplementary Table 2: 1464 differentially expressed genes in GSE47472 dataset. [file 7173972.f2.docx]

| Supplementary Table 2. Differentially Expressed Genes in GSE47472 (1464) | | | | | | | |
| --- | --- | --- | --- | --- | --- | --- | --- |
| Symbol | Entrez | logFC | AveExpr | t | P.Value | adj.P.Val | B |
| CYP19A1 | 1588 | -1.234637994 | 0.278492624 | -13.8985574 | 4.85258E-13 | 1.09345E-08 | 19.53072187 |
| LOC651738 | 651738 | 1.399348418 | -0.1134748 | 13.81283544 | 5.54683E-13 | 1.09345E-08 | 19.40916428 |
| SOCS4 | 122809 | -0.9404008 | 0.143643728 | -5.07134178 | 3.38102E-05 | 0.000850127 | 2.239676161 |
| MGC35361 | 222234 | -1.786276794 | 0.514215378 | -13.01996359 | 1.97198E-12 | 1.92187E-08 | 18.24853437 |
| ACOT2 | 10965 | -2.175008287 | 0.619767537 | -12.89101021 | 2.43731E-12 | 1.92187E-08 | 18.05339206 |
| ZNF575 | 284346 | -2.30365433 | 0.0002417 | -12.68065747 | 3.45544E-12 | 2.16371E-08 | 17.73111649 |
| PCDHGA4 | 56111 | 2.084596356 | -0.687988793 | 12.50856213 | 4.61248E-12 | 2.16371E-08 | 17.46375551 |
| LOC652610 | 652610 | -2.176909135 | 0.601959882 | -12.46803607 | 4.93922E-12 | 2.16371E-08 | 17.40030577 |
| AKT1S1 | 84335 | -1.932945359 | 0.575336286 | -12.16184148 | 8.32868E-12 | 3.28367E-08 | 16.91478709 |
| FHOD1 | 29109 | -1.949966089 | 0.421469043 | -12.0590349 | 9.94728E-12 | 3.56529E-08 | 16.74931567 |
| NAT1 | 9 | 0.92943269 | -0.18168429 | 6.357166389 | 1.3652E-06 | 8.29066E-05 | 5.37694494 |
| LILRP2 | 79166 | -1.282223692 | 0.288496814 | -11.68032504 | 1.93193E-11 | 5.85909E-08 | 16.12888641 |
| CRYZL1 | 9946 | -1.366994202 | 0.180866204 | -11.60368379 | 2.21383E-11 | 6.23446E-08 | 16.00121384 |
| PMP22 | 5376 | -1.426341899 | 0.317486554 | -11.4878957 | 2.72294E-11 | 7.15698E-08 | 15.80695895 |
| A26C3 | 23784 | 0.866206908 | -0.097200765 | 6.405040063 | 1.21514E-06 | 7.65305E-05 | 5.490811568 |
| SCML4 | 256380 | -1.237365958 | 0.23329398 | -10.88952378 | 8.127E-11 | 1.72882E-07 | 14.77627862 |
| HAUS8 | 93323 | -1.980123131 | 0.556870112 | -10.87618191 | 8.33144E-11 | 1.72882E-07 | 14.7527763 |
| LOC644038 | 644038 | 1.889940261 | -0.501284198 | 10.73539338 | 1.08425E-10 | 2.13738E-07 | 14.50335969 |
| LOC643626 | 643626 | -1.58139843 | 0.458339324 | -10.6292664 | 1.32445E-10 | 2.34154E-07 | 14.31363391 |
| RPS19BP1 | 91582 | 2.116589358 | -0.483078645 | 10.62159434 | 1.34381E-10 | 2.34154E-07 | 14.29986093 |
| DDX12 | 440081 | 1.252680098 | -0.414715513 | 10.61294973 | 1.36599E-10 | 2.34154E-07 | 14.28433269 |
| MSTN | 2660 | -2.399118829 | 0.531549829 | -10.58384879 | 1.44345E-10 | 2.37123E-07 | 14.2319864 |
| LOC646691 | 646691 | -1.121362665 | 0.289874934 | -10.48582687 | 1.73945E-10 | 2.74319E-07 | 14.05484057 |
| CDC2L2 | 985 | -1.335749828 | 0.378169531 | -10.44618875 | 1.87635E-10 | 2.84526E-07 | 13.98284344 |
| BTNL8 | 79908 | -1.644943077 | 0.459263851 | -8.796243397 | 5.223E-09 | 1.74949E-06 | 10.79412279 |
| FLYWCH2 | 114984 | -0.921388378 | 0.109132135 | -10.36363141 | 2.19836E-10 | 3.00931E-07 | 13.83221546 |
| LOC650285 | 650285 | -1.365707588 | 0.401097185 | -10.35102162 | 2.25236E-10 | 3.00931E-07 | 13.80912817 |
| SNX11 | 29916 | -1.211181217 | 0.277282209 | -10.33798504 | 2.30962E-10 | 3.00931E-07 | 13.78523699 |
| PRSS2 | 5645 | -1.37217963 | 0.316000676 | -10.31953897 | 2.39322E-10 | 3.00931E-07 | 13.75139322 |
| IL21R | 50615 | -0.643277152 | 0.135977413 | -5.689316322 | 7.10439E-06 | 0.000277041 | 3.763716883 |
| KIAA0101 | 9768 | 1.880082196 | -0.517906915 | 10.2869725 | 2.54853E-10 | 3.0448E-07 | 13.69153032 |
| MCL1 | 4170 | -0.784570845 | 0.188503469 | -6.134712522 | 2.35294E-06 | 0.000121922 | 4.844525955 |
| LOC643011 | 643011 | -1.379597757 | 0.334633985 | -10.22175216 | 2.89165E-10 | 3.25732E-07 | 13.57121352 |
| TTC7A | 57217 | -0.84471581 | 0.084099082 | -10.20610428 | 2.98085E-10 | 3.26453E-07 | 13.54226115 |
| SPAG9 | 9043 | -1.341784612 | 0.360141492 | -10.17650389 | 3.15744E-10 | 3.29676E-07 | 13.48740243 |
| TMPRSS3 | 64699 | -0.873981506 | 0.006208416 | -10.17324652 | 3.17752E-10 | 3.29676E-07 | 13.48135825 |
| CARM1 | 10498 | -2.404761612 | 0.507145873 | -10.08113723 | 3.80347E-10 | 3.84101E-07 | 13.30984829 |
| CCDC40 | 55036 | -1.262046606 | -0.136190897 | -10.06874193 | 3.89693E-10 | 3.84101E-07 | 13.28667961 |
| HS6ST3 | 266722 | -0.919909307 | -0.068122405 | -10.010107 | 4.37229E-10 | 4.20443E-07 | 13.17679736 |
| C12orf69 | 440087 | 1.122847296 | -0.176906764 | 9.945833334 | 4.96265E-10 | 4.65851E-07 | 13.05580655 |
| CPT1C | 126129 | -1.130238304 | 0.294732638 | -9.932755759 | 5.09251E-10 | 4.66924E-07 | 13.03111938 |
| BNIPL | 149428 | 1.123326066 | -0.268172863 | 9.909645171 | 5.33065E-10 | 4.77651E-07 | 12.98743483 |
| ECHDC1 | 55862 | -1.258450174 | 0.28146926 | -9.869857064 | 5.76793E-10 | 5.05347E-07 | 12.91205322 |
| LOC644228 | 644228 | 0.966944793 | -0.208741116 | 9.824924892 | 6.30651E-10 | 5.29086E-07 | 12.82666283 |
| WDR82 | 80335 | -3.22098369 | 0.351458408 | -9.824863881 | 6.30727E-10 | 5.29086E-07 | 12.82654669 |
| HINT3 | 135114 | -0.923040201 | 0.092763312 | -9.796007888 | 6.68032E-10 | 5.48705E-07 | 12.77156031 |
| TNFSF14 | 8740 | 1.175117405 | -0.31507789 | 9.759514059 | 7.18494E-10 | 5.78109E-07 | 12.70185431 |
| ZNF236 | 7776 | 0.884106992 | -0.084254886 | 9.748667429 | 7.34239E-10 | 5.78962E-07 | 12.68110076 |
| LOC649856 | 649856 | -2.278593717 | 0.616904341 | -9.714762633 | 7.85787E-10 | 6.0746E-07 | 12.61612305 |
| ZNF462 | 58499 | -1.744169963 | 0.34801547 | -9.696988891 | 8.14287E-10 | 6.08823E-07 | 12.58199617 |
| LOC440421 | 440421 | -1.004281484 | 0.087383224 | -9.69445586 | 8.18435E-10 | 6.08823E-07 | 12.57712899 |
| DPP8 | 54878 | -0.937732912 | 0.077576176 | -9.664457225 | 8.69245E-10 | 6.34646E-07 | 12.51941895 |
| PVT1 | 5820 | 1.183581197 | -0.284568725 | 9.577408885 | 1.03592E-09 | 7.29325E-07 | 12.35124747 |
| LOC389517 | 389517 | 0.575679683 | -0.125185731 | 5.350328289 | 1.66649E-05 | 0.000503471 | 2.930363921 |
| CHKA | 1119 | 0.765909043 | -0.053988923 | 9.52675033 | 1.14777E-09 | 7.63609E-07 | 12.25288998 |
| MBNL1 | 4154 | -1.079232901 | -0.058622987 | -9.526421194 | 1.14854E-09 | 7.63609E-07 | 12.25224976 |
| LOC649396 | 649396 | -2.085149948 | 0.461769405 | -9.510715101 | 1.18571E-09 | 7.63609E-07 | 12.22168127 |
| FIGF | 2277 | 1.297658165 | -0.32906918 | 9.508992015 | 1.18987E-09 | 7.63609E-07 | 12.21832554 |
| LOC653125 | 653125 | -1.676554254 | 0.352555089 | -9.504474075 | 1.20083E-09 | 7.63609E-07 | 12.20952484 |
| MAGED1 | 9500 | 1.18434443 | -0.224220447 | 9.454535053 | 1.32916E-09 | 8.31799E-07 | 12.112055 |
| ZNF257 | 113835 | -1.357816471 | 0.304776943 | -9.441376541 | 1.36527E-09 | 8.41049E-07 | 12.08631405 |
| NCK2 | 8440 | -1.303846802 | 0.393874614 | -9.422400773 | 1.41914E-09 | 8.55129E-07 | 12.04915027 |
| PCDP1 | 200373 | 1.406184142 | -0.353106167 | 9.410782565 | 1.45319E-09 | 8.55129E-07 | 12.0263711 |
| PKN3 | 29941 | -1.225363738 | 0.321556257 | -9.378019071 | 1.55385E-09 | 9.00912E-07 | 11.96203088 |
| ZCCHC14 | 23174 | 0.9303576 | -0.016181103 | 9.370146701 | 1.57909E-09 | 9.02277E-07 | 11.94654869 |
| C1orf83 | 127428 | 0.937575484 | 0.035006677 | 9.352152865 | 1.63837E-09 | 9.03276E-07 | 11.91112822 |
| ZMYM3 | 9203 | 1.016154972 | -0.210608849 | 9.35174208 | 1.63975E-09 | 9.03276E-07 | 11.91031906 |
| SERPINC1 | 462 | 1.357627379 | -0.391009526 | 9.348469617 | 1.65079E-09 | 9.03276E-07 | 11.90387218 |
| DMP1 | 1758 | 1.471358288 | -0.294552277 | 9.340894669 | 1.67663E-09 | 9.03276E-07 | 11.88894341 |
| AGK | 55750 | 0.715001528 | -0.046060516 | 9.335470767 | 1.69539E-09 | 9.03276E-07 | 11.87824895 |
| LASS2 | 29956 | -1.090819399 | 0.32829141 | -9.253032767 | 2.00869E-09 | 1.0285E-06 | 11.71519035 |
| TM6SF2 | 53345 | 1.039113835 | -0.226513004 | 8.197237959 | 1.90645E-08 | 4.18244E-06 | 9.540848341 |
| LOC392275 | 392275 | 1.518383499 | -0.430560764 | 9.212808986 | 2.18265E-09 | 1.08928E-06 | 11.63527937 |
| GOLGA5 | 9950 | 0.878696773 | -0.126685348 | 9.172303136 | 2.37356E-09 | 1.15639E-06 | 11.55457547 |
| LOC644297 | 644297 | 1.232234305 | -0.335285564 | 9.168511745 | 2.39229E-09 | 1.15639E-06 | 11.54700955 |
| APOA4 | 337 | -1.211431042 | 0.284036192 | -9.165932844 | 2.40511E-09 | 1.15639E-06 | 11.54186204 |
| DDX59 | 83479 | 0.765010202 | -0.141349359 | 5.16715395 | 2.65045E-05 | 0.000707491 | 2.477246038 |
| DCDC5 | 196296 | -0.960825366 | 0.151949203 | -9.139113874 | 2.54277E-09 | 1.19347E-06 | 11.48827501 |
| ACADVL | 37 | -1.347696381 | 0.338871112 | -9.069064406 | 2.94193E-09 | 1.34944E-06 | 11.34782514 |
| LOC654133 | 654133 | 1.471112322 | -0.017323277 | 9.066872251 | 2.95542E-09 | 1.34944E-06 | 11.34341854 |
| OR2T3 | 343173 | 1.370550538 | -0.42756213 | 9.063260581 | 2.97777E-09 | 1.34944E-06 | 11.33615699 |
| LOC652544 | 652544 | -1.041922379 | 0.231409891 | -9.041570272 | 3.11576E-09 | 1.37106E-06 | 11.29250764 |
| LOC653759 | 653759 | -0.818887706 | -0.018140898 | -9.039997772 | 3.12601E-09 | 1.37106E-06 | 11.28934054 |
| PPP1R12C | 54776 | -1.404321329 | 0.311186377 | -9.039419698 | 3.12979E-09 | 1.37106E-06 | 11.28817618 |
| TFAP4 | 7023 | -1.05850997 | 0.244696307 | -9.03148519 | 3.18214E-09 | 1.37867E-06 | 11.27218962 |
| ACPT | 93650 | 2.101301717 | -0.376517064 | 9.007738991 | 3.34426E-09 | 1.43316E-06 | 11.22429163 |
| TLK1 | 9874 | -1.019753127 | -0.051059263 | -8.996080425 | 3.42694E-09 | 1.4528E-06 | 11.20074584 |
| ZNF567 | 163081 | -1.273420316 | 0.160457091 | -8.979959349 | 3.54475E-09 | 1.48676E-06 | 11.16815545 |
| SACS | 26278 | -1.039077468 | 0.271371587 | -8.970650602 | 3.61467E-09 | 1.48689E-06 | 11.14931992 |
| LOC645039 | 645039 | -1.341082755 | 0.414085842 | -8.969884545 | 3.62049E-09 | 1.48689E-06 | 11.14776931 |
| HOXD3 | 3232 | -0.79439643 | 0.167926808 | -8.937608159 | 3.87455E-09 | 1.55786E-06 | 11.08236086 |
| HK1 | 3098 | 0.438084455 | -0.056152397 | 5.857014823 | 4.67595E-06 | 0.000203257 | 4.172780402 |
| FAM89A | 375061 | 1.315475186 | -0.315552189 | 8.924616334 | 3.98192E-09 | 1.55786E-06 | 11.05599064 |
| MAGEA2 | 4101 | 1.488202201 | -0.305208853 | 8.92355082 | 3.99087E-09 | 1.55786E-06 | 11.05382683 |
| PTPRT | 11122 | 0.867453415 | -0.118822186 | 8.909823257 | 4.10793E-09 | 1.58784E-06 | 11.02593484 |
| CDC25B | 994 | -0.79893748 | 0.14284696 | -8.899803025 | 4.19561E-09 | 1.60598E-06 | 11.00555843 |
| TWF1 | 5756 | -1.520469934 | 0.420166621 | -8.895020907 | 4.23813E-09 | 1.60666E-06 | 10.99582879 |
| LOC728302 | 728302 | -1.964232702 | 0.328595594 | -8.887494163 | 4.30595E-09 | 1.61198E-06 | 10.98050831 |
| LOC127602 | 127602 | 0.599736439 | -0.156291685 | 6.0701563 | 2.75834E-06 | 0.000137485 | 4.689039004 |
| ELA3A | 10136 | 1.284003949 | -0.328323426 | 8.869164633 | 4.47584E-09 | 1.64029E-06 | 10.94316506 |
| LARGE | 9215 | 0.866617915 | -0.09414347 | 8.867326173 | 4.49325E-09 | 1.64029E-06 | 10.93941685 |
| HOXA13 | 3209 | -1.590475293 | 0.554033181 | -8.862239617 | 4.5418E-09 | 1.6428E-06 | 10.92904398 |
| OR2A7 | 401427 | -1.360012221 | 0.394766992 | -8.825199189 | 4.91203E-09 | 1.73217E-06 | 10.85339641 |
| DOK3 | 79930 | -1.513204196 | 0.368233009 | -8.824367866 | 4.92069E-09 | 1.73217E-06 | 10.85169634 |
| MIB2 | 142678 | 1.362027651 | -0.374461737 | 8.815954046 | 5.00919E-09 | 1.74502E-06 | 10.83448434 |
| SERPINB4 | 6318 | -1.007114891 | 0.204565256 | -8.81252746 | 5.0457E-09 | 1.74502E-06 | 10.82747172 |
| CARS | 833 | -0.932259074 | 0.078609733 | -8.804192123 | 5.13567E-09 | 1.74949E-06 | 10.81040614 |
| ACAD9 | 28976 | 1.27078551 | -0.305339793 | 8.79411424 | 5.24665E-09 | 1.74949E-06 | 10.78975957 |
| LOC388237 | 388237 | -1.070105862 | 0.033210004 | -8.791082786 | 5.28052E-09 | 1.74949E-06 | 10.78354617 |
| SLBP | 7884 | 0.913268286 | -0.161404068 | 8.778177399 | 5.42722E-09 | 1.78311E-06 | 10.75707993 |
| THPO | 7066 | -0.759128267 | 0.166795613 | -8.765771527 | 5.5722E-09 | 1.81562E-06 | 10.73161552 |
| ZNF552 | 79818 | 1.020958352 | -0.209333651 | 8.756069217 | 5.68837E-09 | 1.83827E-06 | 10.71168505 |
| C17orf101 | 79701 | 0.789600082 | -0.013806828 | 8.709618064 | 6.28003E-09 | 2.01298E-06 | 10.61607746 |
| LOC650369 | 650369 | 1.084758149 | -0.180368635 | 8.702843352 | 6.37147E-09 | 2.02582E-06 | 10.60210754 |
| SPATA6 | 54558 | -1.052804451 | -0.020991174 | -8.659658837 | 6.98746E-09 | 2.2039E-06 | 10.51290275 |
| GIT1 | 28964 | -0.952387017 | 0.273326355 | -8.644967124 | 7.21072E-09 | 2.25627E-06 | 10.48249334 |
| TRIM3 | 10612 | 1.254542508 | -0.29200143 | 8.634476005 | 7.37464E-09 | 2.28939E-06 | 10.46075944 |
| FAM133A | 286499 | 0.900302811 | -0.272717745 | 8.590371183 | 8.10679E-09 | 2.48814E-06 | 10.36921632 |
| TCOF1 | 6949 | -0.968000505 | 0.258981112 | -8.588407246 | 8.14108E-09 | 2.48814E-06 | 10.36513349 |
| FLJ25076 | 134111 | -1.031118057 | 0.148944588 | -8.575774024 | 8.36525E-09 | 2.51762E-06 | 10.33885699 |
| CPNE8 | 144402 | -1.042825288 | -0.094581873 | -8.570793452 | 8.45536E-09 | 2.52397E-06 | 10.32849132 |
| LOC647493 | 647493 | -1.273704164 | 0.276988026 | -8.56756043 | 8.51439E-09 | 2.52397E-06 | 10.32176077 |
| ICOSLG | 23308 | -0.689261195 | 0.092769381 | -8.555908662 | 8.73067E-09 | 2.56877E-06 | 10.29749146 |
| PLXDC1 | 57125 | 0.697746091 | -0.07966708 | 8.544344908 | 8.95091E-09 | 2.61406E-06 | 10.27338613 |
| C7orf20 | 51608 | 1.823033193 | -0.401269683 | 8.527284276 | 9.28632E-09 | 2.66941E-06 | 10.23778703 |
| SENP2 | 59343 | 1.025960392 | -0.119410204 | 8.52538786 | 9.3244E-09 | 2.66941E-06 | 10.23382733 |
| PPP2R2B | 5521 | 1.606928633 | -0.184923395 | 7.360574073 | 1.2597E-07 | 1.47373E-05 | 7.704362465 |
| C10orf25 | 220979 | -0.795535797 | 0.094474303 | -8.495664719 | 9.94257E-09 | 2.81497E-06 | 10.17169799 |
| C12orf64 | 283310 | -0.954407781 | 0.241630868 | -8.490652095 | 1.00509E-08 | 2.81497E-06 | 10.1612077 |
| CORO6 | 84940 | 1.065679664 | -0.171785551 | 8.487140066 | 1.01275E-08 | 2.81497E-06 | 10.15385567 |
| WISP2 | 8839 | 0.688332484 | 0.04889454 | 8.48663285 | 1.01386E-08 | 2.81497E-06 | 10.15279372 |
| SIT1 | 27240 | -1.01887869 | 0.278414416 | -8.478509136 | 1.03184E-08 | 2.84485E-06 | 10.13578024 |
| NDUFB6 | 4712 | 1.02759172 | -0.171813686 | 8.467877174 | 1.05586E-08 | 2.89086E-06 | 10.11349936 |
| P2RY8 | 286530 | 0.796237344 | -0.024457095 | 8.416086986 | 1.18137E-08 | 3.21219E-06 | 10.00473214 |
| LOC158730 | 158730 | -2.218772474 | 0.152074424 | -8.397870989 | 1.22908E-08 | 3.28283E-06 | 9.966383874 |
| CXorf22 | 170063 | -1.15325588 | 0.28003832 | -8.395488332 | 1.23547E-08 | 3.28283E-06 | 9.961364373 |
| LOC644404 | 644404 | 0.858726993 | -0.257782894 | 8.387778713 | 1.25636E-08 | 3.28283E-06 | 9.945117047 |
| ITGB1BP1 | 9270 | -0.988592615 | 0.205584315 | -8.387658504 | 1.25669E-08 | 3.28283E-06 | 9.94486365 |
| EIF4G1 | 1981 | 1.05738101 | -0.269884707 | 8.383973838 | 1.26681E-08 | 3.28283E-06 | 9.93709546 |
| LOC221981 | 221981 | -0.841657021 | 0.168531931 | -8.381501283 | 1.27365E-08 | 3.28283E-06 | 9.931881602 |
| GABRR3 | 200959 | 1.391462403 | -0.120420158 | 8.381386002 | 1.27397E-08 | 3.28283E-06 | 9.931638489 |
| LOC730340 | 730340 | -1.90056284 | 0.471878003 | -8.37546993 | 1.29048E-08 | 3.3038E-06 | 9.919159642 |
| VGF | 7425 | 1.239182332 | -0.086771286 | 8.368217077 | 1.31103E-08 | 3.32989E-06 | 9.90385422 |
| ELK4 | 2005 | 1.068285232 | -0.230246926 | 8.365934575 | 1.31756E-08 | 3.32989E-06 | 9.899035973 |
| SETD4 | 54093 | 1.503248602 | -0.396528087 | 8.346789438 | 1.37372E-08 | 3.4497E-06 | 9.858591995 |
| KIAA2022 | 340533 | -1.223809889 | 0.315655863 | -8.340325813 | 1.39323E-08 | 3.47655E-06 | 9.8449257 |
| RIOK3 | 8780 | -0.838930778 | 0.136175221 | -8.331666015 | 1.41982E-08 | 3.51443E-06 | 9.826606517 |
| TUBGCP6 | 85378 | 0.810508068 | -0.16044242 | 8.329600378 | 1.42624E-08 | 3.51443E-06 | 9.822235213 |
| CANX | 821 | -1.916197327 | 0.442183556 | -8.323029631 | 1.44685E-08 | 3.54308E-06 | 9.8083261 |
| LOC652543 | 652543 | 1.430942171 | -0.397335367 | 8.319648515 | 1.45758E-08 | 3.54732E-06 | 9.801166451 |
| HAO2 | 51179 | 0.830187292 | -0.119253413 | 8.309872629 | 1.48906E-08 | 3.57973E-06 | 9.78045635 |
| AGBL1 | 123624 | -0.924738161 | 0.278739482 | -8.305793648 | 1.5024E-08 | 3.58992E-06 | 9.771811004 |
| GBP5 | 115362 | 1.021417268 | -0.190899322 | 8.302860404 | 1.51207E-08 | 3.59125E-06 | 9.765592554 |
| C1orf26 | 54823 | 1.217618812 | -0.249333167 | 8.28848447 | 1.56039E-08 | 3.65253E-06 | 9.735097774 |
| ENTPD5 | 957 | -1.400503205 | 0.310229895 | -8.286944058 | 1.56566E-08 | 3.65253E-06 | 9.731828426 |
| FADS6 | 283985 | 0.839551209 | -0.098266837 | 8.257727179 | 1.66918E-08 | 3.87112E-06 | 9.669754268 |
| R3HCC1 | 203069 | -0.643715667 | 0.082032022 | -8.253326317 | 1.68537E-08 | 3.88581E-06 | 9.660393547 |
| NTRK1 | 4914 | -1.007344872 | 0.260367648 | -8.236473269 | 1.74888E-08 | 3.98877E-06 | 9.624521 |
| LGR6 | 59352 | -1.277254553 | 0.174096038 | -8.236114717 | 1.75026E-08 | 3.98877E-06 | 9.62375736 |
| USP1 | 7398 | -0.738479638 | 0.050382748 | -8.233330749 | 1.76099E-08 | 3.99017E-06 | 9.617827468 |
| NFIX | 4784 | 0.705592907 | 0.005051488 | 8.214096068 | 1.83703E-08 | 4.13868E-06 | 9.576826841 |
| LOC644714 | 644714 | 1.487896329 | -0.269694884 | 8.204376629 | 1.87673E-08 | 4.18244E-06 | 9.55608865 |
| KRT38 | 8687 | -0.659687343 | -0.039295481 | -8.203562758 | 1.8801E-08 | 4.18244E-06 | 9.554351491 |
| OR4K15 | 81127 | 1.023445767 | -0.229653093 | 8.199993301 | 1.89492E-08 | 4.18244E-06 | 9.546731575 |
| GRK4 | 2868 | 0.916259639 | -0.103872431 | 8.196511789 | 1.9095E-08 | 4.18244E-06 | 9.539297634 |
| AMBRA1 | 55626 | -1.011355334 | 0.245914215 | -8.193579257 | 1.92187E-08 | 4.18628E-06 | 9.533034556 |
| LOC642280 | 642280 | -1.065014177 | 0.262245698 | -8.186413846 | 1.95244E-08 | 4.22949E-06 | 9.517726016 |
| NANP | 140838 | 0.8339733 | 0.06228577 | 5.688218937 | 7.12392E-06 | 0.000277263 | 3.761032396 |
| LOC126536 | 126536 | 0.798445604 | -0.00573201 | 8.167879777 | 2.03384E-08 | 4.35795E-06 | 9.478094659 |
| C1orf104 | 284618 | 1.105058073 | -0.296635985 | 8.141101083 | 2.15767E-08 | 4.57237E-06 | 9.420746621 |
| C10orf72 | 196740 | -1.641537291 | 0.493374535 | -8.138791303 | 2.1687E-08 | 4.57237E-06 | 9.415795273 |
| C12orf67 | 144360 | 0.997919789 | -0.155437181 | 8.135100309 | 2.18646E-08 | 4.58529E-06 | 9.407881502 |
| CCDC117 | 150275 | 1.553349288 | -0.312685499 | 8.128515224 | 2.21851E-08 | 4.6266E-06 | 9.393757719 |
| LOC647571 | 647571 | -2.590031538 | 0.077028859 | -8.125732341 | 2.2322E-08 | 4.6266E-06 | 9.387787079 |
| ICA1L | 130026 | 1.013374126 | -0.288197851 | 8.123879561 | 2.24137E-08 | 4.6266E-06 | 9.383811347 |
| TMEM38B | 55151 | -0.882926783 | -0.044160529 | -8.104003206 | 2.34213E-08 | 4.78663E-06 | 9.341129257 |
| LOC650566 | 650566 | -2.577115811 | 0.270852689 | -8.10198228 | 2.35263E-08 | 4.78663E-06 | 9.336786384 |
| C9orf71 | 169693 | -1.725095817 | 0.267962944 | -8.101467338 | 2.35531E-08 | 4.78663E-06 | 9.335679705 |
| ANKRD34B | 340120 | 0.862175095 | -0.174455177 | 8.095215162 | 2.38815E-08 | 4.82848E-06 | 9.322239902 |
| PRDM2 | 7799 | 1.165027713 | -0.313385074 | 8.082355763 | 2.45718E-08 | 4.9176E-06 | 9.294579444 |
| HNRPC | 3183 | -1.279812648 | 0.37951904 | -8.063894561 | 2.55987E-08 | 5.09725E-06 | 9.254828075 |
| KHDRBS2 | 202559 | -0.695754602 | 0.033563245 | -8.056227081 | 2.60381E-08 | 5.1373E-06 | 9.238303795 |
| LOC723972 | 723972 | -3.188200412 | 0.142658022 | -8.051358672 | 2.63211E-08 | 5.1373E-06 | 9.227807453 |
| ENPP7 | 339221 | 0.854191504 | -0.246764396 | 8.029154325 | 2.76524E-08 | 5.34417E-06 | 9.179891553 |
| AKR1B15 | 441282 | 0.840369156 | -0.186811311 | 8.028739318 | 2.76779E-08 | 5.34417E-06 | 9.178995317 |
| C17orf79 | 55352 | 1.09180667 | -0.202351038 | 8.022603019 | 2.80583E-08 | 5.34417E-06 | 9.165740658 |
| ARHGAP17 | 55114 | -0.676943626 | -0.005340239 | -5.775115184 | 5.73389E-06 | 0.000234994 | 3.973300954 |
| NCOA5 | 57727 | -1.573864948 | 0.427377795 | -8.008622201 | 2.8945E-08 | 5.46022E-06 | 9.135521372 |
| AHR | 196 | 1.590374155 | -0.274879929 | 8.002013243 | 2.93742E-08 | 5.49291E-06 | 9.121226486 |
| TSPYL2 | 64061 | -1.515629575 | 0.365371575 | -7.995989204 | 2.9771E-08 | 5.53657E-06 | 9.108191307 |
| GPNMB | 10457 | -1.011376163 | 0.262698748 | -7.989325689 | 3.02164E-08 | 5.59302E-06 | 9.093766342 |
| DKFZp781N1041 | 387712 | -0.770507822 | 0.15717354 | -7.971328646 | 3.14539E-08 | 5.79486E-06 | 9.054775166 |
| LOC652195 | 652195 | -1.0071886 | 0.188434215 | -7.9535632 | 3.27265E-08 | 6.00128E-06 | 9.016240325 |
| LOC91661 | 91661 | 0.946348182 | -0.252893652 | 7.94898472 | 3.30629E-08 | 6.00673E-06 | 9.006301882 |
| PDPK1 | 5170 | 1.252440472 | 0.110919454 | 7.948363548 | 3.31089E-08 | 6.00673E-06 | 9.004953282 |
| RCN1 | 5954 | -1.4685981 | 0.385282872 | -7.946954442 | 3.32133E-08 | 6.00673E-06 | 9.001893828 |
| TMEM143 | 55260 | -0.925870616 | -0.129353198 | -7.94240913 | 3.35524E-08 | 6.04035E-06 | 8.9920231 |
| PLCG1 | 5335 | -1.630135323 | 0.465236199 | -7.935751214 | 3.40556E-08 | 6.10307E-06 | 8.977559255 |
| RNPC3 | 55599 | -1.051496002 | 0.242311728 | -7.929458022 | 3.45383E-08 | 6.13381E-06 | 8.963881925 |
| LOC730347 | 730347 | -0.961124264 | 0.144292556 | -7.925034075 | 3.48818E-08 | 6.15493E-06 | 8.954263741 |
| LOC389786 | 389786 | -1.4538023 | 0.363908932 | -7.921458096 | 3.51621E-08 | 6.15493E-06 | 8.946487099 |
| OTUD6B | 51633 | 1.051408709 | -0.268722101 | 7.92070724 | 3.52212E-08 | 6.15493E-06 | 8.944853988 |
| TAGLN3 | 29114 | -1.237164375 | 0.316823796 | -7.919941014 | 3.52817E-08 | 6.15493E-06 | 8.943187366 |
| CPVL | 54504 | 1.085343546 | -0.204873001 | 7.915402544 | 3.56419E-08 | 6.19039E-06 | 8.933313991 |
| LIPL2 | 643414 | 1.292054397 | -0.331922455 | 7.910444965 | 3.60398E-08 | 6.21318E-06 | 8.922525495 |
| JAKMIP1 | 152789 | -1.194467162 | -0.284175908 | -7.909119067 | 3.6147E-08 | 6.21318E-06 | 8.91963953 |
| TRIM33 | 51592 | 1.732373335 | -0.314400518 | 7.907898332 | 3.62459E-08 | 6.21318E-06 | 8.916982245 |
| LOC645931 | 645931 | -1.344201219 | 0.322177454 | -7.899884356 | 3.69026E-08 | 6.29836E-06 | 8.899532203 |
| CARTPT | 9607 | -0.890623627 | 0.154410311 | -7.875120358 | 3.90099E-08 | 6.60876E-06 | 8.845551944 |
| LOC652547 | 652547 | -1.428260308 | 0.344447537 | -7.869521557 | 3.95032E-08 | 6.60876E-06 | 8.833335633 |
| CASKIN1 | 57524 | 1.298351672 | -0.116885705 | 7.868704044 | 3.95758E-08 | 6.60876E-06 | 8.831551486 |
| AP3D1 | 8943 | -2.459532645 | 0.674914703 | -7.867774095 | 3.96585E-08 | 6.60876E-06 | 8.82952184 |
| SUPT6H | 6830 | 0.74837468 | -0.149366282 | 7.867601484 | 3.96739E-08 | 6.60876E-06 | 8.829145099 |
| TSSK2 | 23617 | -1.076729365 | 0.27695447 | -7.866564381 | 3.97664E-08 | 6.60876E-06 | 8.826881416 |
| SPATS1 | 221409 | -0.880923991 | 0.153124762 | -7.863827846 | 4.00115E-08 | 6.60876E-06 | 8.820907653 |
| FAM118A | 55007 | 0.688080815 | -0.040720225 | 7.8632635 | 4.00622E-08 | 6.60876E-06 | 8.819675573 |
| TRIM31 | 11074 | -1.365274246 | 0.42494464 | -7.852904955 | 4.10054E-08 | 6.70821E-06 | 8.797052734 |
| SFRS14 | 10147 | -0.745075188 | 0.109190509 | -7.849251071 | 4.13435E-08 | 6.73557E-06 | 8.789069085 |
| PIP4K2C | 79837 | -0.868943656 | 0.200324566 | -7.838750219 | 4.23312E-08 | 6.8681E-06 | 8.766114405 |
| ZC3H8 | 84524 | 1.771347857 | -0.196887173 | 7.832547151 | 4.2926E-08 | 6.90776E-06 | 8.752547235 |
| NAV3 | 89795 | -0.783158857 | 0.102868417 | -7.827643916 | 4.34022E-08 | 6.956E-06 | 8.741819149 |
| PLP1 | 5354 | 1.02420585 | -0.159319382 | 7.819050719 | 4.42499E-08 | 7.06315E-06 | 8.723009333 |
| MPZL2 | 10205 | -0.825833332 | 0.210604447 | -7.802275752 | 4.59541E-08 | 7.30559E-06 | 8.686260047 |
| FLJ20628 | 55006 | 1.052864004 | -0.262344293 | 7.800443472 | 4.61443E-08 | 7.30637E-06 | 8.68224361 |
| LOC641912 | 641912 | -0.742836653 | 0.019321063 | -7.798361475 | 4.63614E-08 | 7.31138E-06 | 8.677679205 |
| LOC653071 | 653071 | 1.05888019 | -0.164194877 | 7.796014325 | 4.66074E-08 | 7.32088E-06 | 8.672532761 |
| PER3 | 8863 | -1.377129139 | 0.3115412 | -7.792831044 | 4.69431E-08 | 7.32104E-06 | 8.665551737 |
| NUDT13 | 25961 | 0.645889031 | -0.035858958 | 7.779044031 | 4.84262E-08 | 7.51673E-06 | 8.635299835 |
| PLTP | 5360 | -1.187863024 | 0.293979685 | -7.771476771 | 4.92605E-08 | 7.61625E-06 | 8.618684054 |
| SLC4A9 | 83697 | -0.860497644 | 0.180258087 | -7.766279778 | 4.9842E-08 | 7.6462E-06 | 8.607268077 |
| LOC651133 | 651133 | 1.263687986 | 0.069867114 | 7.763217469 | 5.0188E-08 | 7.66943E-06 | 8.600539461 |
| LOC344875 | 344875 | -1.039649664 | 0.225830656 | -7.757317117 | 5.08616E-08 | 7.72788E-06 | 8.587571249 |
| MRPS36 | 92259 | 0.91844771 | -0.285119157 | 7.756439336 | 5.09626E-08 | 7.72788E-06 | 8.585641577 |
| LGR5 | 8549 | 1.147915306 | -0.303634391 | 7.749049803 | 5.1821E-08 | 7.82794E-06 | 8.569392465 |
| PPAT | 5471 | 1.414604974 | -0.048181921 | 7.734264534 | 5.35833E-08 | 8.00218E-06 | 8.536857392 |
| TMEM192 | 201931 | 0.955010025 | -0.265120711 | 7.723800103 | 5.48676E-08 | 8.16306E-06 | 8.513811657 |
| WDR16 | 146845 | 0.794815769 | -0.154637717 | 7.714041928 | 5.60938E-08 | 8.31411E-06 | 8.492307353 |
| LOC641953 | 641953 | 1.00976497 | -0.213700116 | 7.710827825 | 5.65038E-08 | 8.3148E-06 | 8.485221419 |
| PLD3 | 23646 | 0.731494242 | -0.023902901 | 7.71069946 | 5.65202E-08 | 8.3148E-06 | 8.48493839 |
| C1R | 715 | -1.137909832 | 0.267623569 | -7.703014525 | 5.75133E-08 | 8.39822E-06 | 8.467989832 |
| OR2H1 | 26716 | -0.870276998 | -0.009442928 | -7.687539565 | 5.95677E-08 | 8.66611E-06 | 8.433835648 |
| ZGPAT | 84619 | -1.498157968 | 0.271569221 | -7.677838275 | 6.08938E-08 | 8.82647E-06 | 8.412407078 |
| PIP | 5304 | -0.802550926 | 0.139364167 | -7.674286874 | 6.13869E-08 | 8.86534E-06 | 8.404559293 |
| SLC30A7 | 148867 | -0.634295766 | 0.048623874 | -7.662845378 | 6.30032E-08 | 9.0326E-06 | 8.37926411 |
| LOC643083 | 643083 | 0.928640934 | -0.246744892 | 7.658749104 | 6.35924E-08 | 9.05788E-06 | 8.370203469 |
| C9orf41 | 138199 | -1.33896725 | 0.219299584 | -7.658324584 | 6.36538E-08 | 9.05788E-06 | 8.369264329 |
| C8orf59 | 401466 | -1.641765082 | -0.381479886 | -7.655332609 | 6.40882E-08 | 9.05788E-06 | 8.362644639 |
| MS4A10 | 341116 | 0.963871555 | -0.176068842 | 7.655261754 | 6.40985E-08 | 9.05788E-06 | 8.362487857 |
| C13orf18 | 80183 | -0.815729757 | 0.141336682 | -7.651968199 | 6.45803E-08 | 9.08358E-06 | 8.355199437 |
| LOC647264 | 647264 | 0.630966237 | -0.053575745 | 7.650873854 | 6.47412E-08 | 9.08358E-06 | 8.352777386 |
| LOC644613 | 644613 | 1.138747088 | -0.237891338 | 7.648885667 | 6.50346E-08 | 9.09239E-06 | 8.348376616 |
| PLK4 | 10733 | -0.780728259 | 0.1641118 | -7.63608534 | 6.69563E-08 | 9.32798E-06 | 8.320030289 |
| MSL3 | 10943 | 0.862324414 | -0.129959882 | 7.631907616 | 6.7596E-08 | 9.38394E-06 | 8.310773733 |
| LOC442512 | 442512 | 0.916330349 | -0.177335839 | 7.62373664 | 6.88654E-08 | 9.46737E-06 | 8.292662258 |
| LOC644373 | 644373 | 0.552732632 | -0.03430528 | 7.620515191 | 6.93725E-08 | 9.49681E-06 | 8.285519139 |
| NIPSNAP3A | 25934 | 1.230870505 | -0.015627976 | 7.614404326 | 7.03451E-08 | 9.58677E-06 | 8.271965134 |
| LOC652781 | 652781 | 1.160968263 | -0.27285032 | 7.613339327 | 7.0516E-08 | 9.58677E-06 | 8.269602412 |
| OR4C46 | 119749 | -1.61378233 | 0.322588133 | -7.604013962 | 7.2031E-08 | 9.75909E-06 | 8.248907088 |
| TNFSF4 | 7292 | 0.845080663 | -0.071862583 | 7.601106608 | 7.25101E-08 | 9.76332E-06 | 8.242452447 |
| AADAC | 13 | 0.900831369 | -0.183227215 | 7.600819866 | 7.25575E-08 | 9.76332E-06 | 8.241815785 |
| TMED7 | 51014 | 1.25061349 | -0.297725488 | 7.59383074 | 7.37235E-08 | 9.88647E-06 | 8.226294033 |
| CTSL2 | 1515 | 1.163088871 | -0.146641592 | 7.588745954 | 7.45838E-08 | 9.94073E-06 | 8.214997218 |
| LOC391045 | 391045 | 1.322339392 | -0.204159509 | 7.588460935 | 7.46324E-08 | 9.94073E-06 | 8.214363885 |
| KLF11 | 8462 | -1.247226712 | 0.228107776 | -7.577693459 | 7.64896E-08 | 1.01538E-05 | 8.190429508 |
| RSL1D1 | 26156 | -0.871114133 | 0.129135005 | -7.558437111 | 7.993E-08 | 1.05749E-05 | 8.147585256 |
| MARS2 | 92935 | -1.029854532 | 0.269203877 | -7.555406346 | 8.04858E-08 | 1.06128E-05 | 8.140837255 |
| LYST | 1130 | -0.802756798 | 0.145077402 | -7.550484343 | 8.13968E-08 | 1.06972E-05 | 8.129875674 |
| RAB24 | 53917 | 0.84741057 | -0.18820757 | 7.541917454 | 8.30077E-08 | 1.08726E-05 | 8.110788654 |
| CDAN1 | 146059 | 0.849864065 | -0.022726724 | 7.53546583 | 8.42425E-08 | 1.09978E-05 | 8.096407677 |
| GNG10 | 2790 | -1.31623189 | 0.277525522 | -7.532717952 | 8.47741E-08 | 1.10307E-05 | 8.090280762 |
| POLG2 | 11232 | -0.95128942 | 0.073390161 | -7.523992989 | 8.64849E-08 | 1.12084E-05 | 8.070819816 |
| FLJ20444 | 403323 | 0.709427596 | -0.157903491 | 7.522867548 | 8.67082E-08 | 1.12084E-05 | 8.068308757 |
| LOC283824 | 283824 | 1.433394946 | -0.14279232 | 7.5159482 | 8.80938E-08 | 1.13154E-05 | 8.052866585 |
| LOC387693 | 387693 | -1.170836897 | 0.281802535 | -7.515869169 | 8.81097E-08 | 1.13154E-05 | 8.052690169 |
| LOC653520 | 653520 | -1.419563137 | 0.251195833 | -7.503534884 | 9.06366E-08 | 1.15885E-05 | 8.025146595 |
| JAM2 | 58494 | 0.800047553 | -0.196228811 | 7.502633198 | 9.08243E-08 | 1.15885E-05 | 8.023132215 |
| LOC652448 | 652448 | -0.649067102 | 0.056322595 | -7.496509575 | 9.2109E-08 | 1.17145E-05 | 8.009448974 |
| FLJ33387 | 161145 | 0.718912412 | -0.000473108 | 7.493023295 | 9.28487E-08 | 1.17337E-05 | 8.001656546 |
| GAL3ST4 | 79690 | 1.132203027 | -0.245140288 | 7.492993776 | 9.2855E-08 | 1.17337E-05 | 8.001590558 |
| CRELD1 | 78987 | 1.119870424 | -0.243776837 | 7.483994202 | 9.4793E-08 | 1.19403E-05 | 7.98146713 |
| LOC645682 | 645682 | 0.786702921 | -0.192708098 | 7.481112316 | 9.54223E-08 | 1.1955E-05 | 7.97502073 |
| STAMBP | 10617 | 0.644244424 | -0.136226837 | 7.480684524 | 9.55161E-08 | 1.1955E-05 | 7.974063718 |
| CTNNA3 | 29119 | -1.555550416 | -0.45931118 | -7.470925283 | 9.76813E-08 | 1.21873E-05 | 7.952224432 |
| TCEAL7 | 56849 | 0.662253236 | -0.091678209 | 7.45499858 | 1.01324E-07 | 1.25831E-05 | 7.916555168 |
| SEMA6B | 10501 | -1.946911244 | 0.415251783 | -7.45332014 | 1.01715E-07 | 1.25831E-05 | 7.912794103 |
| IL28RA | 163702 | -0.727644254 | 0.014819023 | -7.452149342 | 1.0199E-07 | 1.25831E-05 | 7.910170335 |
| ANKRD13C | 81573 | -1.380708441 | 0.299482739 | -7.451551011 | 1.0213E-07 | 1.25831E-05 | 7.908829398 |
| LOC441996 | 441996 | -1.343919117 | -0.38471823 | -7.447151886 | 1.03169E-07 | 1.26665E-05 | 7.898968865 |
| LOC653050 | 653050 | 0.625998418 | -0.070263304 | 7.445968901 | 1.0345E-07 | 1.26665E-05 | 7.896316775 |
| NEDD9 | 4739 | 1.165393409 | -0.215514864 | 7.439990742 | 1.04883E-07 | 1.28022E-05 | 7.882911596 |
| LOC642398 | 642398 | 1.024481398 | -0.053237106 | 7.415256941 | 1.11031E-07 | 1.35109E-05 | 7.827396978 |
| LOC402715 | 402715 | 0.837025045 | -0.176066721 | 7.40934722 | 1.12554E-07 | 1.36541E-05 | 7.814120188 |
| DOHH | 83475 | -0.856341814 | 0.189103287 | -7.407774704 | 1.12963E-07 | 1.36616E-05 | 7.810586557 |
| TNFSF8 | 944 | -1.092322228 | 0.3300399 | -7.400814121 | 1.14791E-07 | 1.38402E-05 | 7.794941201 |
| LOC400721 | 400721 | -1.796622034 | 0.323687234 | -7.398285841 | 1.15463E-07 | 1.38745E-05 | 7.789256714 |
| PDE11A | 50940 | -0.631261007 | 0.148481255 | -7.397098261 | 1.15779E-07 | 1.38745E-05 | 7.7865863 |
| CLDN8 | 9073 | 1.624433688 | -0.26837803 | 7.392612742 | 1.16984E-07 | 1.39764E-05 | 7.776498324 |
| LOC653787 | 653787 | -1.040884113 | 0.270852535 | -7.388254789 | 1.18167E-07 | 1.4075E-05 | 7.76669459 |
| C12orf26 | 84190 | 1.335062814 | -0.280375719 | 7.38316893 | 1.19562E-07 | 1.41984E-05 | 7.755250033 |
| DDX11 | 1663 | -1.026053124 | 0.256097906 | -7.381097457 | 1.20135E-07 | 1.42236E-05 | 7.750587641 |
| LOC647499 | 647499 | -0.803638474 | 0.084897412 | -7.371932288 | 1.22706E-07 | 1.44844E-05 | 7.729951931 |
| LOC647444 | 647444 | -1.279704026 | 0.31034738 | -7.365677865 | 1.24492E-07 | 1.46514E-05 | 7.715863235 |
| DLD | 1738 | 2.173101784 | -0.182108155 | 7.363625934 | 1.25084E-07 | 1.46773E-05 | 7.71123989 |
| DKFZp686J0529 | 388468 | -1.403157854 | 0.399905523 | -7.356956514 | 1.27028E-07 | 1.48054E-05 | 7.696208569 |
| LOC729396 | 729396 | -1.589648256 | 0.384787307 | -7.35602333 | 1.27302E-07 | 1.48054E-05 | 7.694104902 |
| LOC127295 | 127295 | 0.843539705 | -0.062753367 | 7.353665685 | 1.27998E-07 | 1.48425E-05 | 7.688789554 |
| LOC643102 | 643102 | -1.48822965 | 0.39464806 | -7.348695425 | 1.29478E-07 | 1.49701E-05 | 7.677581524 |
| GRASP | 160622 | 1.669806615 | 0.004527228 | 7.342889566 | 1.31229E-07 | 1.50984E-05 | 7.664484909 |
| LBR | 3930 | -0.777883398 | 0.101197832 | -7.342479932 | 1.31354E-07 | 1.50984E-05 | 7.663560698 |
| VARS | 7407 | 1.188752426 | -0.170836691 | 7.33683834 | 1.3308E-07 | 1.52424E-05 | 7.650829884 |
| FRMD4B | 23150 | -0.572095231 | 0.06664453 | -7.335868663 | 1.33379E-07 | 1.52424E-05 | 7.648641271 |
| FERMT3 | 83706 | -0.690033838 | 0.131783741 | -7.334051525 | 1.33942E-07 | 1.52624E-05 | 7.644539547 |
| FLAD1 | 80308 | 1.238382524 | 0.1455529 | 7.325837526 | 1.36514E-07 | 1.54661E-05 | 7.625992902 |
| NOL4 | 8715 | -1.176670851 | 0.276335733 | -7.320001877 | 1.38372E-07 | 1.56317E-05 | 7.612810796 |
| LOC644690 | 644690 | -0.781034302 | 0.134793873 | -7.318162062 | 1.38964E-07 | 1.56397E-05 | 7.608653885 |
| DRD3 | 1814 | -1.642938569 | 0.354782803 | -7.317316808 | 1.39236E-07 | 1.56397E-05 | 7.606743946 |
| ZNF507 | 22847 | -1.568496669 | 0.323934925 | -7.311735901 | 1.41049E-07 | 1.57983E-05 | 7.594130864 |
| LOC646262 | 646262 | -0.803694149 | 0.166829042 | -7.309803237 | 1.41682E-07 | 1.58243E-05 | 7.589761972 |
| FGFR1 | 2260 | 2.152405824 | -0.098748268 | 7.291028688 | 1.47989E-07 | 1.64355E-05 | 7.547294509 |
| ZFP106 | 64397 | 0.636971443 | -0.078874705 | 7.288876123 | 1.4873E-07 | 1.64715E-05 | 7.542422398 |
| LOC654164 | 654164 | 0.817481338 | -0.1863166 | 7.282558469 | 1.50928E-07 | 1.65939E-05 | 7.528119384 |
| CCDC46 | 201134 | 0.831945021 | -0.052623977 | 7.28220546 | 1.51052E-07 | 1.65939E-05 | 7.527320021 |
| LOC731823 | 731823 | -1.262870405 | 0.382619723 | -7.281820594 | 1.51187E-07 | 1.65939E-05 | 7.526448498 |
| FER1L5 | 90342 | -0.89043077 | 0.155726209 | -7.275398096 | 1.53459E-07 | 1.67597E-05 | 7.511901899 |
| CNGA3 | 1261 | -2.10753983 | 0.650262244 | -7.272063551 | 1.54652E-07 | 1.68434E-05 | 7.504347126 |
| PAG1 | 55824 | -1.19772043 | 0.284554256 | -7.250764132 | 1.62503E-07 | 1.76012E-05 | 7.45605529 |
| LOC285550 | 285550 | -0.742553219 | -0.091337592 | -7.249097286 | 1.63134E-07 | 1.76212E-05 | 7.452273477 |
| DCUN1D3 | 123879 | -1.402520446 | 0.136131973 | -7.241995646 | 1.65852E-07 | 1.78173E-05 | 7.436156732 |
| GAS2L1 | 10634 | -1.118720809 | 0.298618768 | -7.241992494 | 1.65853E-07 | 1.78173E-05 | 7.436149577 |
| PCDHB5 | 26167 | 0.968799136 | -0.223562369 | 7.234825395 | 1.68644E-07 | 1.80678E-05 | 7.419877339 |
| MGC33407 | 284382 | 0.546920805 | -0.018863677 | 7.221743083 | 1.73861E-07 | 1.85763E-05 | 7.390157206 |
| MBTPS1 | 8720 | 1.187411706 | -0.273139997 | 7.216759724 | 1.75892E-07 | 1.87424E-05 | 7.378830018 |
| LOC642219 | 642219 | -2.017133003 | 0.258319473 | -7.204650269 | 1.80928E-07 | 1.92271E-05 | 7.351291202 |
| CASP14 | 23581 | 1.16697135 | -0.209662935 | 7.199399988 | 1.83157E-07 | 1.94117E-05 | 7.339345075 |
| C2orf19 | 394261 | 1.820681913 | -0.148512445 | 7.192207547 | 1.86257E-07 | 1.96873E-05 | 7.322973855 |
| SLC22A12 | 116085 | 1.021090124 | -0.228309894 | 7.187956566 | 1.88114E-07 | 1.98304E-05 | 7.313294619 |
| CINP | 51550 | 0.994454452 | -0.219004451 | 7.184466375 | 1.89653E-07 | 1.98883E-05 | 7.305345837 |
| RAD21 | 5885 | 0.776856744 | -0.050319154 | 7.183969827 | 1.89873E-07 | 1.98883E-05 | 7.304214833 |
| FOXF1 | 2294 | -1.144227696 | -0.304256166 | -7.181835085 | 1.90822E-07 | 1.98883E-05 | 7.299352085 |
| C2orf76 | 130355 | 1.440608736 | -0.322763656 | 7.181073935 | 1.91161E-07 | 1.98883E-05 | 7.297618106 |
| TCF3 | 6929 | -1.188755255 | 0.209942006 | -7.180152951 | 1.91573E-07 | 1.98883E-05 | 7.295519905 |
| SP4 | 6671 | 1.066294685 | 0.09369143 | 7.179892225 | 1.91689E-07 | 1.98883E-05 | 7.294925893 |
| TMOD3 | 29766 | 0.88419999 | -0.194165651 | 7.177008275 | 1.92985E-07 | 1.99701E-05 | 7.288354789 |
| SHOX | 6473 | 0.669542702 | -0.067950666 | 7.175003364 | 1.93891E-07 | 2.00113E-05 | 7.283785921 |
| TYSND1 | 219743 | -0.694396006 | -0.049590954 | -7.170945686 | 1.95737E-07 | 2.01492E-05 | 7.274537478 |
| OVOS2 | 144203 | 1.082948554 | -0.187669104 | 7.164671149 | 1.98628E-07 | 2.03406E-05 | 7.260231911 |
| LOC651728 | 651728 | -1.498454828 | 0.278326464 | -7.149080706 | 2.06003E-07 | 2.09867E-05 | 7.224663744 |
| PRL | 5617 | 0.52651399 | 0.021341448 | 7.138116879 | 2.11356E-07 | 2.14715E-05 | 7.199631257 |
| ADAMTS6 | 11174 | 0.621102081 | -0.103546868 | 7.137118268 | 2.1185E-07 | 2.14715E-05 | 7.19735044 |
| DMD | 1756 | 1.219195122 | -0.217946199 | 7.13177451 | 2.14517E-07 | 2.16861E-05 | 7.185143081 |
| SERPINB7 | 8710 | 0.645090015 | -0.102193535 | 7.130098812 | 2.15361E-07 | 2.17156E-05 | 7.181314307 |
| LOC653314 | 653314 | 0.678605018 | -0.116628409 | 7.12774455 | 2.16551E-07 | 2.178E-05 | 7.175934455 |
| SNIP | 80725 | -0.672959164 | 0.104980063 | -7.126569598 | 2.17148E-07 | 2.17844E-05 | 7.173249233 |
| CYP4Z2P | 163720 | 1.093417103 | -0.215742627 | 7.123938093 | 2.18491E-07 | 2.18635E-05 | 7.16723455 |
| RNF190 | 162333 | -1.339462694 | 0.240256835 | -7.116075133 | 2.22553E-07 | 2.22136E-05 | 7.149257112 |
| ZNF519 | 162655 | 1.017054883 | 0.125478482 | 7.111925401 | 2.24728E-07 | 2.2374E-05 | 7.139766065 |
| OGT | 8473 | 0.910929097 | -0.178702894 | 7.108537993 | 2.26519E-07 | 2.24956E-05 | 7.132016861 |
| HEATR4 | 399671 | -0.938277046 | 0.079101742 | -7.104982699 | 2.28416E-07 | 2.25971E-05 | 7.123881951 |
| SUPT4H1 | 6827 | 0.705483269 | -0.165176685 | 7.103824692 | 2.29037E-07 | 2.25971E-05 | 7.121231936 |
| RSPO1 | 284654 | 0.798166202 | -0.168358273 | 7.103407676 | 2.29261E-07 | 2.25971E-05 | 7.120277582 |
| DUSP3 | 1845 | -0.93417083 | -0.116819624 | -7.091394838 | 2.35813E-07 | 2.31816E-05 | 7.092775879 |
| NAPSA | 9476 | -1.820661436 | 0.156572278 | -7.090394327 | 2.36367E-07 | 2.31816E-05 | 7.090484483 |
| GRIA4 | 2893 | 0.953925086 | -0.208092637 | 7.087249853 | 2.38118E-07 | 2.32954E-05 | 7.083282063 |
| CCDC13 | 152206 | 0.789811674 | -0.131056007 | 7.082696732 | 2.40677E-07 | 2.34874E-05 | 7.072850807 |
| LOC646094 | 646094 | 1.037263337 | 0.109421597 | 7.076618155 | 2.44136E-07 | 2.37077E-05 | 7.058920419 |
| C15orf48 | 84419 | -1.230644736 | -0.257788608 | -7.065409465 | 2.5065E-07 | 2.42804E-05 | 7.033220397 |
| STRADB | 55437 | 1.427865624 | -0.192273614 | 7.063792398 | 2.51604E-07 | 2.43131E-05 | 7.029511305 |
| BCOR | 54880 | -0.978829757 | 0.223138016 | -7.058798024 | 2.54575E-07 | 2.454E-05 | 7.01805344 |
| KLHL34 | 257240 | -0.864422315 | 0.204619942 | -7.057665288 | 2.55254E-07 | 2.45455E-05 | 7.015454311 |
| EEF1D | 1936 | -0.773693692 | 0.069335646 | -7.055299757 | 2.56677E-07 | 2.46223E-05 | 7.010025911 |
| LOC641788 | 641788 | 0.632128543 | -0.097908378 | 7.051545583 | 2.58953E-07 | 2.47803E-05 | 7.001409345 |
| LOC644623 | 644623 | 1.115732611 | 0.247421061 | 7.046183736 | 2.62239E-07 | 2.49905E-05 | 6.989099622 |
| ADAM18 | 8749 | -1.100286373 | 0.258009233 | -7.045895842 | 2.62417E-07 | 2.49905E-05 | 6.988438569 |
| SPRN | 503542 | 2.202067061 | 0.105693554 | 7.034986907 | 2.6924E-07 | 2.5517E-05 | 6.963381711 |
| SRMS | 6725 | 0.93098113 | -0.208876897 | 7.033876039 | 2.69945E-07 | 2.55224E-05 | 6.960829263 |
| PCDHGB6 | 56100 | 1.358439096 | -0.230372358 | 7.032443719 | 2.70857E-07 | 2.55474E-05 | 6.957537975 |
| RGS3 | 5998 | 0.608694166 | -0.068686284 | 7.030017386 | 2.72408E-07 | 2.56017E-05 | 6.951961954 |
| TP53AIP1 | 63970 | -0.798385764 | 0.068428653 | -7.029514272 | 2.72731E-07 | 2.56017E-05 | 6.950805636 |
| ARPP-21 | 10777 | 0.767744878 | 0.100054707 | 6.596042178 | 7.65604E-07 | 5.52833E-05 | 5.942458553 |
| LOC643441 | 643441 | -1.033490682 | 0.250070164 | -7.01426935 | 2.82702E-07 | 2.63494E-05 | 6.915752116 |
| IGF2 | 3481 | -0.743932109 | 0.12524327 | -7.006296859 | 2.88064E-07 | 2.67859E-05 | 6.897408339 |
| C8orf44 | 56260 | 1.067866112 | -0.088230725 | 7.001992495 | 2.91002E-07 | 2.69954E-05 | 6.887501025 |
| FOXL1 | 2300 | 1.548771618 | -0.209729605 | 6.997541789 | 2.94072E-07 | 2.72162E-05 | 6.877254319 |
| UPK3A | 7380 | -0.825157273 | 0.098624255 | -6.994470658 | 2.9621E-07 | 2.73498E-05 | 6.870182245 |
| UBL5 | 59286 | -0.973284299 | 0.28242555 | -6.989607822 | 2.99628E-07 | 2.76008E-05 | 6.858981781 |
| LEPR | 3953 | -2.326834608 | 0.306595386 | -6.987697987 | 3.00981E-07 | 2.76123E-05 | 6.854582054 |
| LOC654165 | 654165 | -0.974954261 | 0.172585508 | -6.987016906 | 3.01465E-07 | 2.76123E-05 | 6.853012916 |
| EFS | 10278 | -1.07717899 | 0.157745109 | -6.986471037 | 3.01854E-07 | 2.76123E-05 | 6.851755248 |
| LOC648943 | 648943 | -1.769927273 | 0.179107175 | -6.978251998 | 3.07767E-07 | 2.8059E-05 | 6.832814099 |
| MGC4677 | 112597 | 1.171489282 | -0.217733212 | 6.977710618 | 3.0816E-07 | 2.8059E-05 | 6.831566154 |
| DOK6 | 220164 | 1.220568106 | -0.121877262 | 6.972247566 | 3.12161E-07 | 2.83578E-05 | 6.818971031 |
| HCN1 | 348980 | -0.810361262 | 0.190062513 | -6.963846387 | 3.18418E-07 | 2.87389E-05 | 6.799594415 |
| NUPR1 | 26471 | -1.090890787 | 0.08309554 | -6.963824848 | 3.18434E-07 | 2.87389E-05 | 6.799544725 |
| IPMK | 253430 | 0.92041893 | -0.240642577 | 6.963132316 | 3.18955E-07 | 2.87389E-05 | 6.797947046 |
| LIG4 | 3981 | -1.292545367 | 0.364281273 | -6.962624351 | 3.19338E-07 | 2.87389E-05 | 6.796775125 |
| RLF | 6018 | -0.987174928 | -0.29081725 | -6.960952314 | 3.20602E-07 | 2.87389E-05 | 6.792917346 |
| IFIT3 | 3437 | -1.122146964 | 0.32267336 | -6.960086799 | 3.21259E-07 | 2.87389E-05 | 6.790920259 |
| LOC642515 | 642515 | 1.073767922 | -0.166778441 | 6.955025548 | 3.25125E-07 | 2.88881E-05 | 6.779239985 |
| PDE2A | 5138 | 1.375914735 | -0.147789052 | 6.954933601 | 3.25195E-07 | 2.88881E-05 | 6.779027759 |
| GHRHR | 2692 | -1.149585609 | 0.070817182 | -6.950299263 | 3.28778E-07 | 2.9129E-05 | 6.768329727 |
| EPB41L1 | 2036 | 0.900994628 | -0.239313824 | 6.946269459 | 3.31926E-07 | 2.93419E-05 | 6.759024945 |
| CUL2 | 8453 | 1.742318049 | 0.233318433 | 6.936034582 | 3.4006E-07 | 2.99023E-05 | 6.735383211 |
| LOC653210 | 653210 | -1.04367631 | 0.265141741 | -6.927122454 | 3.47308E-07 | 3.03614E-05 | 6.714785845 |
| NEUROG3 | 50674 | 0.652549611 | -0.008691452 | 6.923633768 | 3.50189E-07 | 3.05454E-05 | 6.706720124 |
| RBM15B | 29890 | 1.581099484 | -0.345852619 | 6.905747947 | 3.65344E-07 | 3.1727E-05 | 6.665343983 |
| KLC2 | 64837 | -1.094551818 | 0.000594878 | -6.900037686 | 3.70322E-07 | 3.20182E-05 | 6.652125452 |
| PLXNA3 | 55558 | 0.570835419 | -0.092509653 | 6.893503662 | 3.76104E-07 | 3.2447E-05 | 6.636994854 |
| LOC648763 | 648763 | 0.978294177 | -0.200064381 | 6.88781776 | 3.8121E-07 | 3.28157E-05 | 6.623823743 |
| C1orf64 | 149563 | -0.859071088 | 0.264782072 | -6.886830672 | 3.82104E-07 | 3.2821E-05 | 6.621536779 |
| OLFM1 | 10439 | -0.801624303 | 0.19106423 | -6.885540657 | 3.83275E-07 | 3.285E-05 | 6.618547781 |
| SCGB2A2 | 4250 | 0.742342395 | 0.090850595 | 6.882304916 | 3.86229E-07 | 3.30313E-05 | 6.611049542 |
| ITGB3BP | 23421 | -0.770239533 | 0.122312753 | -6.876830728 | 3.91279E-07 | 3.33908E-05 | 6.598361045 |
| PCDHGC3 | 5098 | 0.854543421 | -0.20019836 | 6.857640837 | 4.09521E-07 | 3.4804E-05 | 6.553850854 |
| GPR155 | 151556 | 1.021574347 | -0.186839684 | 6.857556128 | 4.09604E-07 | 3.4804E-05 | 6.553654268 |
| LOC643772 | 643772 | -1.343676159 | 0.368929085 | -6.854440502 | 4.12647E-07 | 3.49872E-05 | 6.546423213 |
| LNPEP | 4012 | -1.643382286 | 0.341403447 | -6.850803032 | 4.1623E-07 | 3.51671E-05 | 6.537979436 |
| RBM23 | 55147 | -1.135237866 | 0.238991433 | -5.362406202 | 1.61639E-05 | 0.000492869 | 2.960181769 |
| ZCCHC4 | 29063 | 0.669096911 | -0.09222078 | 6.840189516 | 4.26866E-07 | 3.5822E-05 | 6.513332277 |
| PDC | 5132 | 0.68184002 | -0.125945804 | 6.839699367 | 4.27363E-07 | 3.5822E-05 | 6.512193686 |
| RP11-49G10.8 | 317716 | -0.930821502 | 0.300473106 | -6.839127765 | 4.27945E-07 | 3.5822E-05 | 6.510865843 |
| BTN2A1 | 11120 | -1.846220499 | 0.413270544 | -6.834428073 | 4.32755E-07 | 3.61479E-05 | 6.49994679 |
| ANGPTL6 | 83854 | 0.996898362 | -0.173159617 | 6.819681594 | 4.4821E-07 | 3.71617E-05 | 6.465667225 |
| LOC642434 | 642434 | -0.994352082 | 0.265762706 | -6.819257744 | 4.48663E-07 | 3.71617E-05 | 6.464681536 |
| ARSB | 411 | -0.916999494 | 0.131188787 | -6.816365514 | 4.51763E-07 | 3.734E-05 | 6.457954878 |
| CAB39L | 81617 | 0.887066247 | -0.142193103 | 5.625100141 | 8.34391E-06 | 0.000310346 | 3.606469782 |
| WDR8 | 49856 | 0.785964806 | -0.172437081 | 6.808703175 | 4.60081E-07 | 3.77917E-05 | 6.440128917 |
| BAP1 | 8314 | 0.619036292 | -0.183923026 | 6.807653602 | 4.61233E-07 | 3.77917E-05 | 6.437686571 |
| LOC339674 | 339674 | -0.713385583 | 0.052133987 | -6.806937494 | 4.62021E-07 | 3.77917E-05 | 6.436020113 |
| EFCAB3 | 146779 | 0.976437016 | -0.010407701 | 6.805019597 | 4.64136E-07 | 3.78862E-05 | 6.431556651 |
| LOC644625 | 644625 | 0.870550747 | -0.069692155 | 6.79622723 | 4.73963E-07 | 3.86084E-05 | 6.411088475 |
| RNF17 | 56163 | -2.070001007 | 0.339238682 | -6.779769217 | 4.92932E-07 | 4.00708E-05 | 6.372748811 |
| KPNA3 | 3839 | 0.61256631 | -0.104243441 | 6.771641514 | 5.02584E-07 | 4.07155E-05 | 6.353802369 |
| LOC727805 | 727805 | 0.822697496 | -0.140240987 | 6.771353922 | 5.02929E-07 | 4.07155E-05 | 6.353131813 |
| LOC644097 | 644097 | 0.55051131 | -0.017460228 | 6.768363446 | 5.06531E-07 | 4.09231E-05 | 6.346158529 |
| PLEKHA8 | 84725 | -1.580601589 | 0.200728989 | -6.767141061 | 5.08011E-07 | 4.09588E-05 | 6.343307812 |
| NFIC | 4782 | -0.966472172 | 0.173805756 | -6.758239446 | 5.18923E-07 | 4.17532E-05 | 6.322542736 |
| WDR45 | 11152 | 0.794395633 | -0.148058524 | 6.756556283 | 5.21013E-07 | 4.1836E-05 | 6.318615252 |
| RAET1G | 353091 | 1.017758976 | -0.201642314 | 6.750228105 | 5.28948E-07 | 4.22906E-05 | 6.30384593 |
| HOXA10 | 3206 | -1.249651262 | -0.405038856 | -6.749499824 | 5.29869E-07 | 4.22906E-05 | 6.302145874 |
| CXorf41 | 139212 | -1.483108744 | 0.178203246 | -6.749480988 | 5.29893E-07 | 4.22906E-05 | 6.302101905 |
| LOC647841 | 647841 | -1.056451497 | 0.289620521 | -6.747466666 | 5.32449E-07 | 4.24088E-05 | 6.297399441 |
| DOK2 | 9046 | 1.441424202 | -0.161215178 | 6.742281334 | 5.39088E-07 | 4.2851E-05 | 6.285291873 |
| LOC652054 | 652054 | 0.920147078 | -0.255758958 | 6.737918574 | 5.44739E-07 | 4.3213E-05 | 6.275102377 |
| TMEM14A | 28978 | -0.957122425 | 0.232594928 | -6.731881789 | 5.52658E-07 | 4.37532E-05 | 6.260999171 |
| STX5 | 6811 | -0.748272124 | 0.000266335 | -6.72031856 | 5.68157E-07 | 4.48337E-05 | 6.233972335 |
| LOC388080 | 388080 | -1.817832518 | -0.153391115 | -6.720006854 | 5.68581E-07 | 4.48337E-05 | 6.233243551 |
| ST3GAL3 | 6487 | -0.615863103 | 0.066699319 | -6.716676242 | 5.7313E-07 | 4.51022E-05 | 6.225455664 |
| LOC649937 | 649937 | -0.655193996 | 0.068043593 | -6.715632034 | 5.74564E-07 | 4.5125E-05 | 6.223013735 |
| DLX2 | 1746 | 1.274497325 | -0.194251904 | 6.714787279 | 5.75727E-07 | 4.51264E-05 | 6.221038137 |
| EMR3 | 84658 | 1.960862323 | -0.118194542 | 6.711771904 | 5.79897E-07 | 4.52733E-05 | 6.213985463 |
| RNF208 | 727800 | 1.762001566 | -0.171942736 | 6.707657066 | 5.85637E-07 | 4.55748E-05 | 6.204359429 |
| OGFOD1 | 55239 | -0.924686695 | 0.156331928 | -6.70734785 | 5.86071E-07 | 4.55748E-05 | 6.20363598 |
| KLHL7 | 55975 | 0.688598875 | -0.140144051 | 6.696416369 | 6.01616E-07 | 4.65998E-05 | 6.178052832 |
| UGT3A2 | 167127 | -0.750290217 | 0.095195995 | -6.6924762 | 6.07322E-07 | 4.69496E-05 | 6.168827951 |
| C2CD4A | 145741 | -0.641430999 | 0.071899303 | -6.686745098 | 6.1572E-07 | 4.74534E-05 | 6.155406642 |
| BCAP31 | 10134 | 0.699060154 | -0.0958273 | 6.6849145 | 6.18427E-07 | 4.75285E-05 | 6.151118823 |
| NXPH4 | 11247 | 0.769886318 | -0.103740384 | 6.678699489 | 6.2771E-07 | 4.8062E-05 | 6.136558288 |
| MGC27382 | 149047 | 0.803142393 | -0.167807394 | 6.678635441 | 6.27807E-07 | 4.8062E-05 | 6.136408212 |
| FLJ40722 | 285966 | -1.838378838 | 0.501555198 | -6.671420037 | 6.38764E-07 | 4.8806E-05 | 6.119497926 |
| SLITRK6 | 84189 | 1.028916573 | -0.095307079 | 6.665114192 | 6.485E-07 | 4.94503E-05 | 6.104714067 |
| LOC652608 | 652608 | -1.008624301 | 0.229681589 | -6.663889906 | 6.50408E-07 | 4.94503E-05 | 6.101843199 |
| OR4X2 | 119764 | 0.765253765 | -0.169286641 | 6.663536379 | 6.5096E-07 | 4.94503E-05 | 6.101014168 |
| MAF1 | 84232 | 0.999875151 | -0.23019325 | 6.660751616 | 6.55324E-07 | 4.96389E-05 | 6.094483283 |
| LOC650008 | 650008 | -1.374786052 | 0.209950436 | -6.660347158 | 6.5596E-07 | 4.96389E-05 | 6.093534659 |
| PIK3C2A | 5286 | 0.935873926 | -0.24575371 | 6.65283752 | 6.67891E-07 | 5.0445E-05 | 6.075917778 |
| NXNL1 | 115861 | -1.664372537 | 0.105700809 | -6.651079155 | 6.70717E-07 | 5.05615E-05 | 6.071791826 |
| ASAH2 | 56624 | -0.813948629 | 0.079428871 | -6.649842392 | 6.72711E-07 | 5.06151E-05 | 6.068889571 |
| CACYBP | 27101 | 0.871315012 | -0.155044519 | 6.645781916 | 6.79302E-07 | 5.08733E-05 | 6.059359721 |
| RBMX2 | 51634 | -0.573752112 | 0.079815728 | -6.644441612 | 6.81493E-07 | 5.08733E-05 | 6.056213614 |
| LINS1 | 55180 | 1.175721032 | -0.210519852 | 6.643718208 | 6.82678E-07 | 5.08733E-05 | 6.054515469 |
| PCBD1 | 5092 | -0.923676912 | 0.265050567 | -6.643431133 | 6.83149E-07 | 5.08733E-05 | 6.053841562 |
| NFYB | 4801 | 0.668084765 | -0.067335155 | 6.6429828 | 6.83885E-07 | 5.08733E-05 | 6.052789082 |
| OR8B12 | 219858 | 1.023181993 | -0.17526319 | 6.641916743 | 6.85638E-07 | 5.09076E-05 | 6.050286368 |
| FLJ00312 | 399761 | 0.843465862 | 0.045582869 | 6.639030845 | 6.90408E-07 | 5.11479E-05 | 6.043510639 |
| MRGPRF | 219928 | 1.094477379 | -0.123952082 | 6.638391888 | 6.91468E-07 | 5.11479E-05 | 6.042010308 |
| DEPDC5 | 9681 | -1.615455409 | 0.181231303 | -6.635641846 | 6.96052E-07 | 5.13905E-05 | 6.035552391 |
| SCUBE2 | 57758 | -0.710029265 | 0.190661634 | -6.633810129 | 6.99122E-07 | 5.15207E-05 | 6.031250461 |
| C7orf25 | 79020 | 1.103536294 | -0.280829134 | 6.618633969 | 7.25098E-07 | 5.3236E-05 | 5.995592347 |
| PLEKHG4 | 25894 | -0.648604684 | 0.123005145 | -6.612183519 | 7.36435E-07 | 5.39678E-05 | 5.980427798 |
| VKORC1 | 79001 | -0.709841919 | 0.128462182 | -6.610736393 | 7.39003E-07 | 5.40556E-05 | 5.977025013 |
| TBXA2R | 6915 | -0.97011383 | 0.219050475 | -6.065573533 | 2.78969E-06 | 0.000138347 | 4.677985129 |
| NOX1 | 27035 | 0.708778336 | -0.114064828 | 6.603220801 | 7.52489E-07 | 5.48385E-05 | 5.959348709 |
| NRG3 | 10718 | 0.567060616 | 0.027662168 | 6.60051272 | 7.57409E-07 | 5.50534E-05 | 5.952977759 |
| AGPS | 8540 | -0.82589621 | 0.035773031 | -6.599298272 | 7.59626E-07 | 5.50534E-05 | 5.950120396 |
| FBXW7 | 55294 | 1.234662486 | -0.09842145 | 6.598119065 | 7.61786E-07 | 5.51086E-05 | 5.947345778 |
| SEC24C | 9632 | 0.56468206 | 0.08586945 | 6.586469853 | 7.83456E-07 | 5.6469E-05 | 5.919926713 |
| WNK1 | 65125 | 0.826870476 | -0.205966612 | 6.579121129 | 7.97449E-07 | 5.73727E-05 | 5.902621406 |
| C16orf48 | 84080 | -0.913749687 | 0.228816969 | -6.57704066 | 8.01457E-07 | 5.75423E-05 | 5.897720988 |
| LOC652684 | 652684 | -0.812635873 | 0.177426768 | -6.576384394 | 8.02725E-07 | 5.75423E-05 | 5.896175085 |
| LOC388955 | 388955 | -1.580588294 | 0.249326001 | -6.573726322 | 8.07884E-07 | 5.77022E-05 | 5.889913189 |
| LOC652324 | 652324 | -0.932898428 | 0.258996863 | -6.570003122 | 8.15166E-07 | 5.81171E-05 | 5.881140636 |
| SPRR2C | 6702 | 0.880392774 | 0.068911322 | 6.569248166 | 8.16651E-07 | 5.81178E-05 | 5.879361614 |
| GCK | 2645 | 1.266177429 | -0.140512937 | 6.567918928 | 8.19272E-07 | 5.81993E-05 | 5.876229159 |
| FCGR3A | 2214 | 0.536342217 | -0.021200112 | 6.565137313 | 8.24785E-07 | 5.84617E-05 | 5.869673374 |
| SPATS2L | 26010 | -0.787949004 | 0.183825566 | -6.563988366 | 8.27073E-07 | 5.84617E-05 | 5.866965233 |
| EVC | 2121 | -1.281598334 | 0.249518901 | -6.561878953 | 8.3129E-07 | 5.86305E-05 | 5.861992803 |
| KIF23 | 9493 | 0.836062723 | -0.183399902 | 6.56049044 | 8.34079E-07 | 5.87221E-05 | 5.858719429 |
| GGT3P | 2679 | 0.750325242 | -0.143727341 | 6.559424155 | 8.36226E-07 | 5.87684E-05 | 5.856205541 |
| ADAMTS19 | 171019 | -0.970038826 | 0.255642776 | -6.55780211 | 8.39504E-07 | 5.88938E-05 | 5.852381122 |
| LOC390231 | 390231 | 0.555467697 | -0.09351425 | 6.555717383 | 8.43736E-07 | 5.90526E-05 | 5.847465344 |
| OR2A9P | 441295 | 1.055870094 | -0.047735254 | 6.555212515 | 8.44764E-07 | 5.90526E-05 | 5.846274788 |
| PRPSAP2 | 5636 | 0.931997078 | -0.168147353 | 6.552895886 | 8.49499E-07 | 5.92785E-05 | 5.840811435 |
| LRRC58 | 116064 | 0.875468148 | -0.169702897 | 6.536350494 | 8.84107E-07 | 6.14758E-05 | 5.801773483 |
| ABCC11 | 85320 | -0.733853627 | 0.182071018 | -6.532365624 | 8.92655E-07 | 6.18662E-05 | 5.79236653 |
| FAM18B | 51030 | -1.470633247 | 0.197072161 | -6.531748854 | 8.93986E-07 | 6.18662E-05 | 5.790910372 |
| MPZL3 | 196264 | 0.724491653 | -0.004184427 | 6.531543759 | 8.94428E-07 | 6.18662E-05 | 5.790426144 |
| PEX16 | 9409 | -1.496222427 | 0.115526181 | -6.530482513 | 8.96724E-07 | 6.19163E-05 | 5.787920474 |
| FKBP6 | 8468 | -1.433077044 | 0.134235383 | -6.529748449 | 8.98315E-07 | 6.19178E-05 | 5.786187223 |
| GNB1 | 2782 | 0.698834975 | -0.130265052 | 6.526237166 | 9.05966E-07 | 6.22377E-05 | 5.777895595 |
| TEAD4 | 7004 | 0.66557273 | -0.100761228 | 6.526170095 | 9.06113E-07 | 6.22377E-05 | 5.777737198 |
| C3orf72 | 401089 | 0.996779696 | -0.177862332 | 6.521989585 | 9.15311E-07 | 6.27601E-05 | 5.767863303 |
| MAGEF1 | 64110 | -1.013565045 | 0.214462556 | -6.51733778 | 9.25657E-07 | 6.32859E-05 | 5.756873829 |
| SULT1A1 | 6817 | -0.612874515 | 0.041383437 | -6.517100067 | 9.26189E-07 | 6.32859E-05 | 5.756312182 |
| GPRC5D | 55507 | 1.002777913 | -0.209042158 | 6.514257563 | 9.32574E-07 | 6.36119E-05 | 5.749595679 |
| P2RXL1 | 9127 | -1.45384566 | 0.353048509 | -6.513343567 | 9.34636E-07 | 6.36424E-05 | 5.747435813 |
| DHRS12 | 79758 | -1.623416865 | 0.025110385 | -6.512613043 | 9.36288E-07 | 6.3645E-05 | 5.745709436 |
| CLCN6 | 1185 | 0.928706136 | -0.199429957 | 6.510749272 | 9.40516E-07 | 6.38224E-05 | 5.741304683 |
| TTTY12 | 83867 | 0.718786415 | -0.133036264 | 6.504313437 | 9.55265E-07 | 6.47118E-05 | 5.726091369 |
| GRIPAP1 | 56850 | -0.994329759 | 0.240776971 | -6.502937177 | 9.5845E-07 | 6.48162E-05 | 5.72283747 |
| PSMC3IP | 29893 | 0.691942947 | -0.143522187 | 6.50087236 | 9.63248E-07 | 6.50291E-05 | 5.717955194 |
| RAB9P1 | 9366 | 0.854536959 | -0.234957858 | 6.498776435 | 9.68144E-07 | 6.52479E-05 | 5.712998849 |
| LOC643738 | 643738 | 0.937761106 | -0.145090817 | 6.49361528 | 9.80307E-07 | 6.57696E-05 | 5.700791787 |
| EFHB | 151651 | 0.871914782 | -0.215183406 | 6.493416326 | 9.80779E-07 | 6.57696E-05 | 5.700321161 |
| LOC442020 | 442020 | 0.550787296 | -0.057294235 | 6.493370232 | 9.80888E-07 | 6.57696E-05 | 5.700212126 |
| HEPACAM2 | 253012 | 1.842771975 | 0.063761701 | 6.492271788 | 9.83499E-07 | 6.58144E-05 | 5.697613672 |
| LOC646951 | 646951 | -1.04695771 | 0.186983687 | -6.491685054 | 9.84896E-07 | 6.58144E-05 | 5.696225651 |
| CLN5 | 1203 | -0.874074447 | 0.225220927 | -6.489472674 | 9.90183E-07 | 6.60557E-05 | 5.690991514 |
| MID2 | 11043 | 0.636072612 | -0.15914268 | 6.486816443 | 9.96568E-07 | 6.63694E-05 | 5.684706535 |
| CCR4 | 1233 | 0.566087838 | -0.035640218 | 6.483717251 | 1.00407E-06 | 6.67564E-05 | 5.677372412 |
| EPN2 | 22905 | 0.575132555 | -0.027805578 | 6.476087406 | 1.02279E-06 | 6.78579E-05 | 5.659311869 |
| MGC57359 | 441272 | 1.044199063 | -0.129845691 | 6.474306896 | 1.02721E-06 | 6.78579E-05 | 5.655096258 |
| CTAGE1 | 64693 | -0.928171472 | 0.216302053 | -6.474180665 | 1.02752E-06 | 6.78579E-05 | 5.654797374 |
| TMCC1 | 23023 | -0.916936112 | 0.288521659 | -6.472849652 | 1.03084E-06 | 6.79632E-05 | 5.651645749 |
| BACH2 | 60468 | 2.002952249 | -0.142808872 | 6.47068869 | 1.03625E-06 | 6.82057E-05 | 5.646528499 |
| ZNF274 | 10782 | -0.761112936 | 0.197829227 | -6.467328877 | 1.04472E-06 | 6.86485E-05 | 5.638571236 |
| UBASH3A | 53347 | -0.997178322 | 0.195231244 | -6.461221459 | 1.06029E-06 | 6.95296E-05 | 5.624103273 |
| NKX6-1 | 4825 | 0.897459324 | -0.198363386 | 6.460006322 | 1.06342E-06 | 6.95296E-05 | 5.621224196 |
| FLJ46109 | 653399 | -1.271404617 | -0.205534954 | -6.452751834 | 1.08228E-06 | 7.06458E-05 | 5.604032252 |
| PAQR6 | 79957 | 1.22921536 | -0.135361616 | 6.445740888 | 1.10084E-06 | 7.17381E-05 | 5.587411656 |
| LOC652860 | 652860 | -0.749924517 | 0.07660425 | -6.442897991 | 1.10845E-06 | 7.20401E-05 | 5.580670479 |
| PTCHD3 | 374308 | -1.698651692 | 0.172877827 | -6.442647636 | 1.10912E-06 | 7.20401E-05 | 5.580076783 |
| GNA12 | 2768 | 1.346216285 | 0.060129549 | 6.439380331 | 1.11795E-06 | 7.24468E-05 | 5.572327986 |
| ZNF233 | 353355 | 1.158118291 | -0.133317916 | 6.43897046 | 1.11906E-06 | 7.24468E-05 | 5.571355841 |
| OVCA2 | 124641 | 0.600281393 | -0.061035427 | 6.430396117 | 1.14258E-06 | 7.3743E-05 | 5.551014505 |
| LOC644019 | 644019 | 0.878659283 | -0.211441844 | 6.095865144 | 2.589E-06 | 0.000130864 | 4.751010887 |
| FGD6 | 55785 | -1.182333335 | 0.356213594 | -6.42808212 | 1.14902E-06 | 7.39008E-05 | 5.545523443 |
| LILRA2 | 11027 | -0.734453916 | 0.216625626 | -6.426197535 | 1.15429E-06 | 7.41187E-05 | 5.541050909 |
| LOC653537 | 653537 | -0.771735588 | -0.15225794 | -6.422338598 | 1.16515E-06 | 7.44525E-05 | 5.531891529 |
| HPS4 | 89781 | -0.722983889 | 0.153743243 | -6.420985662 | 1.16898E-06 | 7.45593E-05 | 5.528679862 |
| HR | 55806 | 0.843721553 | -0.13116057 | 6.420414975 | 1.1706E-06 | 7.45593E-05 | 5.527325074 |
| ACSL6 | 23305 | 0.896548085 | -0.199551088 | 6.417010345 | 1.18032E-06 | 7.50569E-05 | 5.519241838 |
| SIRT2 | 22933 | 0.754684395 | -0.205466071 | 6.414966012 | 1.18619E-06 | 7.5309E-05 | 5.514387566 |
| ARG2 | 384 | -0.80851589 | 0.133500791 | -6.411546692 | 1.19609E-06 | 7.58149E-05 | 5.506267312 |
| LOC649365 | 649365 | -0.643041948 | 0.031905183 | -6.409387459 | 1.20237E-06 | 7.60912E-05 | 5.501138842 |
| LOC644695 | 644695 | -0.795634211 | 0.246589426 | -6.407018813 | 1.20931E-06 | 7.63675E-05 | 5.495512374 |
| STIP1 | 10963 | 0.957201839 | -0.183273254 | 6.406576237 | 1.21061E-06 | 7.63675E-05 | 5.494461009 |
| RTBDN | 83546 | -0.625883725 | 0.074507717 | -6.401875109 | 1.22452E-06 | 7.69463E-05 | 5.483291836 |
| ZNF648 | 127665 | 0.793482955 | -0.177852034 | 6.401497549 | 1.22564E-06 | 7.69463E-05 | 5.4823947 |
| RUNX1T1 | 862 | 1.202764392 | -0.16490793 | 6.399843054 | 1.23058E-06 | 7.7066E-05 | 5.478463197 |
| SHISA3 | 152573 | 0.821043854 | -0.196134785 | 6.399549303 | 1.23146E-06 | 7.7066E-05 | 5.477765137 |
| TAS2R7 | 50837 | 0.556255621 | -0.027283545 | 6.397793267 | 1.23673E-06 | 7.72729E-05 | 5.473591944 |
| A1CF | 29974 | 0.637949761 | 0.049372158 | 6.395377726 | 1.24401E-06 | 7.76049E-05 | 5.467850873 |
| RPL32 | 6161 | 0.73823408 | -0.005594002 | 6.385001839 | 1.27579E-06 | 7.93364E-05 | 5.443182705 |
| ZMAT5 | 55954 | -0.568650183 | 0.067876608 | -6.382364344 | 1.284E-06 | 7.96463E-05 | 5.436910236 |
| tAKR | 389932 | 0.848958908 | -0.025348029 | 6.382104103 | 1.28481E-06 | 7.96463E-05 | 5.436291292 |
| C7orf43 | 55262 | 0.558856729 | -0.091578534 | 6.378816287 | 1.29513E-06 | 8.01407E-05 | 5.428471032 |
| LOC100101266 | 100101266 | -0.593835811 | 0.130182313 | -6.375037316 | 1.30709E-06 | 8.05208E-05 | 5.419481017 |
| LOC645946 | 645946 | 0.68009241 | -0.056120722 | 6.373925218 | 1.31063E-06 | 8.0613E-05 | 5.416835073 |
| CPNE7 | 27132 | -0.564032939 | 0.039790209 | -6.371360157 | 1.31884E-06 | 8.09914E-05 | 5.410731655 |
| MBTD1 | 54799 | -1.037420292 | 0.207051386 | -6.369706552 | 1.32415E-06 | 8.11915E-05 | 5.4067966 |
| CA12 | 771 | 1.088597652 | 0.06907481 | 6.36440727 | 1.34134E-06 | 8.19904E-05 | 5.394183908 |
| C10orf41 | 283065 | -0.775573346 | 0.149367082 | -6.362664602 | 1.34705E-06 | 8.22115E-05 | 5.390035533 |
| IPO4 | 79711 | -0.742340381 | 0.025288233 | -6.357983583 | 1.36248E-06 | 8.29066E-05 | 5.378890798 |
| LOC646779 | 646779 | 1.203492689 | -0.025862149 | 6.357293326 | 1.36478E-06 | 8.29066E-05 | 5.377247199 |
| LOC389031 | 389031 | 0.820035783 | -0.143903192 | 6.356199506 | 1.36842E-06 | 8.29066E-05 | 5.374642554 |
| GSTO1 | 9446 | 1.264609557 | -0.218472727 | 6.356039249 | 1.36895E-06 | 8.29066E-05 | 5.374260932 |
| C8orf37 | 157657 | 0.531586915 | -0.047961905 | 6.352232281 | 1.3817E-06 | 8.35505E-05 | 5.365194515 |
| C19orf12 | 83636 | -0.945423349 | -0.131774235 | -6.347482254 | 1.39778E-06 | 8.43933E-05 | 5.353879884 |
| QARS | 5859 | -0.728587183 | 0.212532374 | -6.344874629 | 1.40669E-06 | 8.48013E-05 | 5.347667411 |
| VHL | 7428 | 0.839205534 | -0.204308784 | 6.343156491 | 1.41259E-06 | 8.5027E-05 | 5.343573655 |
| MAGOHB | 55110 | -1.935421609 | 0.314891188 | -6.342477269 | 1.41493E-06 | 8.5038E-05 | 5.341955202 |
| LOC645908 | 645908 | -1.180033012 | 0.145602926 | -6.338830893 | 1.42756E-06 | 8.56664E-05 | 5.333265722 |
| EPHA5 | 2044 | 1.967272863 | -0.043722952 | 6.336434298 | 1.43592E-06 | 8.59067E-05 | 5.327553716 |
| LOC400986 | 400986 | -1.076684478 | 0.242418675 | -6.330113811 | 1.45821E-06 | 8.71083E-05 | 5.312486487 |
| TUBGCP3 | 10426 | -1.345862093 | 0.342338413 | -6.329100391 | 1.46182E-06 | 8.71917E-05 | 5.310070209 |
| PCMT1 | 5110 | -0.6604352 | 0.096849519 | -6.322513639 | 1.48549E-06 | 8.83361E-05 | 5.294362754 |
| MMP15 | 4324 | 0.520315588 | 0.063829538 | 6.321476366 | 1.48925E-06 | 8.84265E-05 | 5.291888724 |
| XRN1 | 54464 | 0.557909826 | -0.099410038 | 6.320400388 | 1.49316E-06 | 8.85255E-05 | 5.289322251 |
| APEX1 | 328 | -0.709572731 | 0.121238322 | -6.318934947 | 1.49851E-06 | 8.87091E-05 | 5.285826605 |
| ZNF367 | 195828 | 0.608892314 | -0.060671465 | 6.314927035 | 1.51323E-06 | 8.94463E-05 | 5.276264963 |
| ZNF354B | 117608 | 1.01557053 | -0.146357935 | 6.313220763 | 1.51955E-06 | 8.9685E-05 | 5.272193783 |
| TBC1D3 | 84218 | 0.581413965 | -0.093805665 | 6.312330913 | 1.52285E-06 | 8.97456E-05 | 5.270070463 |
| FOXP1 | 27086 | 0.916886711 | -0.142879831 | 6.310938841 | 1.52803E-06 | 8.99165E-05 | 5.266748591 |
| C1QL3 | 389941 | -1.17636465 | 0.205136435 | -6.308084502 | 1.53871E-06 | 9.04101E-05 | 5.25993667 |
| RAPGEF1 | 2889 | 0.863105914 | 0.219612577 | 6.302930843 | 1.55819E-06 | 9.14182E-05 | 5.247635101 |
| TRO | 7216 | -0.936778941 | 0.234139016 | -6.301559875 | 1.56341E-06 | 9.15884E-05 | 5.244362163 |
| LOC728758 | 728758 | -0.701897301 | 0.133795173 | -6.299797647 | 1.57015E-06 | 9.18467E-05 | 5.240154858 |
| LOC644422 | 644422 | 1.288418634 | -0.169968483 | 6.296358857 | 1.58339E-06 | 9.24839E-05 | 5.231943787 |
| GPC5 | 2262 | 0.561716536 | 0.007913223 | 6.294693684 | 1.58984E-06 | 9.27233E-05 | 5.227967254 |
| SPACA5B | 729201 | -0.952355228 | 0.184846165 | -6.290111923 | 1.60773E-06 | 9.35196E-05 | 5.217024156 |
| CRMP1 | 1400 | 0.604615382 | -0.035029496 | 6.289982672 | 1.60823E-06 | 9.35196E-05 | 5.216715417 |
| CST4 | 1472 | 0.865479616 | -0.110856836 | 6.288010733 | 1.616E-06 | 9.38327E-05 | 5.212004891 |
| C10orf96 | 374355 | -1.689839049 | -0.300214895 | -6.286874672 | 1.62049E-06 | 9.39551E-05 | 5.209290898 |
| PDZK1IP1 | 10158 | 0.721099212 | -0.112354869 | 6.285044258 | 1.62775E-06 | 9.42375E-05 | 5.204917832 |
| TMEM76 | 138050 | -0.933124995 | 0.155041446 | -6.283968375 | 1.63204E-06 | 9.4347E-05 | 5.202347256 |
| CLTB | 1212 | -1.149727941 | -0.219799575 | -6.280571544 | 1.64564E-06 | 9.4994E-05 | 5.194230466 |
| LOC654002 | 654002 | 1.083691487 | 0.198667315 | 6.279230159 | 1.65104E-06 | 9.51665E-05 | 5.191024853 |
| NFX1 | 4799 | -1.154971904 | -0.104650507 | -6.27774912 | 1.65703E-06 | 9.52574E-05 | 5.187485272 |
| FGL1 | 2267 | -0.872777657 | 0.224073644 | -6.277644663 | 1.65745E-06 | 9.52574E-05 | 5.187235617 |
| TRPC1 | 7220 | -0.73529895 | 0.176005981 | -6.276447375 | 1.66231E-06 | 9.53632E-05 | 5.18437399 |
| LOC647447 | 647447 | -1.273788493 | 0.313541856 | -6.275999285 | 1.66413E-06 | 9.53632E-05 | 5.183302975 |
| LOC387723 | 387723 | 1.591230659 | -0.092448241 | 6.271024948 | 1.68448E-06 | 9.63896E-05 | 5.171411936 |
| GLT8D1 | 55830 | 0.597564724 | 0.066958281 | 6.270028209 | 1.68859E-06 | 9.64125E-05 | 5.169028929 |
| MAP4K4 | 9448 | 0.60703742 | -0.128811322 | 6.269741974 | 1.68977E-06 | 9.64125E-05 | 5.168344578 |
| AP1B1 | 162 | 0.846841611 | -0.246541272 | 6.268415903 | 1.69526E-06 | 9.65858E-05 | 5.165173992 |
| CRYBA1 | 1411 | -1.447774484 | 0.196146795 | -6.266217549 | 1.7044E-06 | 9.69662E-05 | 5.15991739 |
| MAP1S | 55201 | 1.061213338 | -0.150477888 | 6.26025278 | 1.72944E-06 | 9.81008E-05 | 5.145652056 |
| LOC647634 | 647634 | -0.932665519 | 0.215901141 | -6.259906156 | 1.73091E-06 | 9.81008E-05 | 5.144822952 |
| LOC644668 | 644668 | -0.664109073 | 0.102282136 | -6.259693666 | 1.7318E-06 | 9.81008E-05 | 5.144314682 |
| DNAJB11 | 51726 | 0.818266552 | -0.219395608 | 6.258532965 | 1.73673E-06 | 9.82385E-05 | 5.14153823 |
| DDX19A | 55308 | -0.925183731 | -0.096728693 | -6.257594324 | 1.74072E-06 | 9.83232E-05 | 5.139292848 |
| LOC649009 | 649009 | -1.654713876 | 0.304444673 | -6.251131369 | 1.76846E-06 | 9.97472E-05 | 5.12382982 |
| AAA1 | 404744 | -0.672125258 | 0.141897134 | -6.248646081 | 1.77925E-06 | 0.000100212 | 5.117882404 |
| FLJ13224 | 79857 | -1.304966345 | -0.009110014 | -6.247897577 | 1.78251E-06 | 0.000100253 | 5.116091067 |
| ZNF766 | 90321 | -0.670882101 | 0.207149601 | -6.245720183 | 1.79203E-06 | 0.000100645 | 5.110879732 |
| LOC440354 | 440354 | -0.67133442 | 0.089809395 | -6.245107478 | 1.79472E-06 | 0.000100652 | 5.109413201 |
| LOC650488 | 650488 | -0.581944905 | 0.047316976 | -6.242200916 | 1.80753E-06 | 0.000101227 | 5.102455695 |
| APOBEC3F | 200316 | -0.780528266 | 0.0971195 | -6.239218412 | 1.82078E-06 | 0.000101824 | 5.095315456 |
| SLC8A1 | 6546 | -0.695759117 | 0.175996924 | -6.226624063 | 1.8778E-06 | 0.000104858 | 5.065153488 |
| RIBC1 | 158787 | 0.592835725 | -0.077944631 | 6.223845788 | 1.89062E-06 | 0.000105282 | 5.058497552 |
| GPR125 | 166647 | -1.413737272 | 0.306463424 | -6.223023594 | 1.89443E-06 | 0.000105312 | 5.056527657 |
| FAHD2A | 51011 | 0.904156637 | -0.13907307 | 6.222031499 | 1.89904E-06 | 0.000105312 | 5.054150599 |
| CTU2 | 348180 | 0.651702146 | 0.011133636 | 6.222005282 | 1.89917E-06 | 0.000105312 | 5.054087781 |
| FLJ26850 | 400710 | -0.696275172 | -0.09574258 | -6.218858451 | 1.91386E-06 | 0.000105978 | 5.046547275 |
| HSF2BP | 11077 | 0.646083933 | -0.023979307 | 6.218092041 | 1.91746E-06 | 0.000106028 | 5.044710625 |
| TRIM34 | 53840 | 0.860010166 | -0.141068473 | 6.217116085 | 1.92205E-06 | 0.000106133 | 5.042371721 |
| C1orf14 | 81626 | -0.712015841 | 0.135216346 | -6.210486454 | 1.95353E-06 | 0.000107628 | 5.026480946 |
| OR2AG2 | 338755 | 0.683628497 | -0.20419618 | 6.209874904 | 1.95646E-06 | 0.000107628 | 5.025014865 |
| GUCA1B | 2979 | 0.593663932 | -0.13072077 | 6.209697821 | 1.95731E-06 | 0.000107628 | 5.024590334 |
| LOC652881 | 652881 | 0.645112966 | -0.118897012 | 6.208897304 | 1.96116E-06 | 0.000107689 | 5.022671163 |
| LOC643936 | 643936 | -1.426320594 | 0.280393628 | -6.208327979 | 1.9639E-06 | 0.000107689 | 5.021306214 |
| ZNF626 | 199777 | -0.766967125 | 0.15502245 | -6.204360828 | 1.98309E-06 | 0.000108591 | 5.011794067 |
| SPEF2 | 79925 | -0.758921071 | 0.113181058 | -6.201722213 | 1.99596E-06 | 0.00010908 | 5.00546646 |
| CRSP2 | 9282 | -1.273955757 | 0.211202769 | -6.201396527 | 1.99756E-06 | 0.00010908 | 5.004685386 |
| ANP32B | 10541 | -0.742886449 | 0.075721241 | -6.197211931 | 2.01816E-06 | 0.0001099 | 4.99464873 |
| ATP6AP1L | 92270 | -1.222939729 | 0.272397509 | -6.193246141 | 2.03788E-06 | 0.000110822 | 4.98513516 |
| LOC730274 | 730274 | -1.051050418 | 0.175029752 | -6.190006262 | 2.05414E-06 | 0.000111552 | 4.977361748 |
| CDC2L6 | 23097 | -0.935108442 | 0.239485343 | -6.188423542 | 2.06214E-06 | 0.000111682 | 4.973563937 |
| SLTM | 79811 | 0.48891679 | -0.050732194 | 6.188410643 | 2.0622E-06 | 0.000111682 | 4.973532985 |
| TNRC6B | 23112 | -0.774028051 | 0.20848754 | -6.183849437 | 2.08541E-06 | 0.000112705 | 4.962586672 |
| C19orf6 | 91304 | -0.839983497 | -0.169695926 | -6.183576572 | 2.08681E-06 | 0.000112705 | 4.961931761 |
| LOC196993 | 196993 | -0.74844678 | -0.138849587 | -6.181120247 | 2.09942E-06 | 0.000113141 | 4.956035916 |
| UFC1 | 51506 | 0.471967693 | -0.02415703 | 6.18088624 | 2.10063E-06 | 0.000113141 | 4.955474201 |
| PTPRD | 5789 | 0.707107663 | -0.203961403 | 6.177959544 | 2.11577E-06 | 0.000113801 | 4.948448432 |
| TMEM144 | 55314 | 0.890389152 | -0.19044602 | 6.176947234 | 2.12104E-06 | 0.000113929 | 4.946018093 |
| GPSM1 | 26086 | -1.251179451 | -0.048500977 | -6.176088561 | 2.12551E-06 | 0.000114014 | 4.943956515 |
| HERC2 | 8924 | -1.857026751 | -0.394945593 | -6.174019592 | 2.13633E-06 | 0.000114439 | 4.938988838 |
| PRO1853 | 55471 | -0.712248669 | 0.100929712 | -6.168161068 | 2.16728E-06 | 0.000115782 | 4.924919848 |
| ACTL7B | 10880 | -1.009625245 | 0.165704217 | -6.166321195 | 2.1771E-06 | 0.000115992 | 4.920500731 |
| NAP1L1 | 4673 | -0.76127582 | 0.108441524 | -6.162859969 | 2.19568E-06 | 0.000116825 | 4.912186389 |
| CCDC48 | 79825 | 1.028375739 | -0.254190371 | 6.158211686 | 2.2209E-06 | 0.000117848 | 4.901018599 |
| DCST2 | 127579 | -0.772445301 | 0.1086509 | -6.156515279 | 2.23017E-06 | 0.000118181 | 4.896942315 |
| BRD8 | 10902 | -1.297660372 | 0.233419384 | -6.154820542 | 2.23948E-06 | 0.000118515 | 4.892869742 |
| LTBP4 | 8425 | 0.608984494 | -0.144285967 | 6.153776918 | 2.24523E-06 | 0.000118554 | 4.890361691 |
| METTL14 | 57721 | -0.674428231 | 0.146512607 | -6.153596607 | 2.24622E-06 | 0.000118554 | 4.889928352 |
| OR10A4 | 283297 | -0.928002412 | 0.262543855 | -6.149816219 | 2.26718E-06 | 0.000119345 | 4.880842253 |
| PPP1CA | 5499 | 1.039803346 | -0.114333642 | 6.14785894 | 2.27812E-06 | 0.000119756 | 4.876137381 |
| MYOZ2 | 51778 | 0.741598176 | -0.151802505 | 6.147315018 | 2.28116E-06 | 0.000119757 | 4.874829842 |
| FKSG30 | 440915 | -0.730622045 | 0.138989993 | -6.145211192 | 2.29299E-06 | 0.000120217 | 4.869772139 |
| PTGS2 | 5743 | 1.159185424 | 0.002236968 | 6.1432442 | 2.3041E-06 | 0.000120596 | 4.865042976 |
| RAB18 | 22931 | 0.707509416 | -0.024595438 | 6.142852647 | 2.30632E-06 | 0.000120596 | 4.864101533 |
| LRIG2 | 9860 | 0.537259669 | -0.0822339 | 6.141831436 | 2.31212E-06 | 0.000120739 | 4.861646075 |
| ZNF212 | 7988 | 0.893239221 | -0.042990455 | 6.138471945 | 2.33129E-06 | 0.00012151 | 4.853567564 |
| KIAA1751 | 85452 | 0.661584995 | -0.112856851 | 6.138165074 | 2.33305E-06 | 0.00012151 | 4.852829577 |
| IL13 | 3596 | 0.603100937 | -0.141453982 | 6.136848907 | 2.34062E-06 | 0.000121682 | 4.849664253 |
| LOC645582 | 645582 | 1.097298556 | -0.217863373 | 6.136517309 | 2.34252E-06 | 0.000121682 | 4.848866745 |
| LOC649432 | 649432 | 0.677138957 | -0.066431869 | 6.134644756 | 2.35334E-06 | 0.000121922 | 4.844362962 |
| TMEM105 | 284186 | 0.500659857 | -0.02776008 | 6.1311157 | 2.37385E-06 | 0.000122823 | 4.835874043 |
| LOC645882 | 645882 | -0.625838897 | 0.101532864 | -6.129205675 | 2.38503E-06 | 0.00012324 | 4.831279066 |
| LRRC37A2 | 474170 | 0.514322294 | -0.080425452 | 6.12848449 | 2.38926E-06 | 0.000123297 | 4.829544001 |
| ADSS | 159 | 0.52919178 | -0.079291848 | 6.125812087 | 2.40502E-06 | 0.000123948 | 4.823114125 |
| DSCR10 | 259234 | -0.613305193 | -0.05129243 | -6.122293933 | 2.42592E-06 | 0.000124862 | 4.814648231 |
| MTRR | 4552 | -0.828130464 | 0.202909578 | -6.120826066 | 2.4347E-06 | 0.000125151 | 4.811115661 |
| LOC653658 | 653658 | 0.699828043 | -0.20357509 | 6.11695775 | 2.45799E-06 | 0.000126183 | 4.801805103 |
| LOC441081 | 441081 | -1.169701995 | 0.236003994 | -6.109940912 | 2.50081E-06 | 0.000128214 | 4.784912528 |
| NP | 4860 | -1.364911811 | 0.19529859 | -6.106508899 | 2.52202E-06 | 0.000128967 | 4.776648351 |
| LOC642954 | 642954 | -0.733290096 | 0.136403767 | -6.105235961 | 2.52994E-06 | 0.000129204 | 4.773582849 |
| OR10J1 | 26476 | 1.030687688 | -0.159591678 | 6.104350721 | 2.53546E-06 | 0.000129318 | 4.771450909 |
| IL12A | 3592 | 0.587940607 | -0.077672209 | 6.102809288 | 2.5451E-06 | 0.000129492 | 4.767738457 |
| ZC3H7B | 23264 | 0.750530484 | -0.182017615 | 6.102757838 | 2.54543E-06 | 0.000129492 | 4.767614539 |
| RBM11 | 54033 | 0.625002677 | -0.127830018 | 6.101577808 | 2.55283E-06 | 0.000129645 | 4.764772335 |
| CRYM | 1428 | 0.478430199 | -0.047764705 | 6.100537062 | 2.55938E-06 | 0.0001297 | 4.76226549 |
| LOC401072 | 401072 | 0.642334174 | -0.104888545 | 6.097452574 | 2.5789E-06 | 0.000130521 | 4.754835239 |
| PLEKHG1 | 57480 | -0.621182295 | -0.049203142 | -6.094303255 | 2.59898E-06 | 0.0001312 | 4.747247818 |
| ZNF324B | 388569 | 0.842693586 | -0.114919759 | 6.092519196 | 2.61043E-06 | 0.00013161 | 4.742949171 |
| LOC647031 | 647031 | -0.861898723 | 0.153521683 | -6.09058479 | 2.6229E-06 | 0.000132069 | 4.7382879 |
| NCF1B | 654816 | 1.147937449 | -0.296601458 | 6.084202753 | 2.66447E-06 | 0.000133828 | 4.722906642 |
| OSBPL10 | 114884 | 0.885478443 | -0.215338252 | 6.084180421 | 2.66462E-06 | 0.000133828 | 4.722852813 |
| ZBED3 | 84327 | 1.185411657 | 0.018263014 | 6.082677043 | 2.67451E-06 | 0.000134154 | 4.719228938 |
| IQCB1 | 9657 | 0.700920719 | -0.14635085 | 6.079049829 | 2.69852E-06 | 0.000135187 | 4.710484648 |
| SLC36A3 | 285641 | -0.878924093 | 0.121935123 | -6.076288961 | 2.71695E-06 | 0.000135937 | 4.703828008 |
| GYS2 | 2998 | 0.642704181 | -0.132590265 | 6.070742819 | 2.75435E-06 | 0.000137485 | 4.690453568 |
| RBMY1J | 378951 | 0.747996967 | -0.110323921 | 6.070230443 | 2.75784E-06 | 0.000137485 | 4.689217825 |
| C6orf208 | 80069 | -0.612038618 | 0.137354724 | -6.069281748 | 2.76429E-06 | 0.000137607 | 4.686929701 |
| COL13A1 | 1305 | -1.38561199 | 0.019652717 | -6.0683556 | 2.77061E-06 | 0.000137748 | 4.684695872 |
| CTAG1B | 1485 | 0.580886777 | 0.012041776 | 6.066131071 | 2.78585E-06 | 0.000138331 | 4.679330053 |
| LRGUK | 136332 | 0.380165476 | -0.016279015 | 6.063159594 | 2.80635E-06 | 0.000138999 | 4.672161735 |
| GUCY2F | 2986 | 0.5784261 | -0.128511809 | 6.062442272 | 2.81132E-06 | 0.00013907 | 4.670431152 |
| LOC653904 | 653904 | -1.232900968 | 0.170543229 | -6.061225197 | 2.81977E-06 | 0.000139313 | 4.667494768 |
| DSPP | 1834 | -0.846751234 | 0.187007131 | -6.055649468 | 2.85882E-06 | 0.00014096 | 4.654040558 |
| MMP20 | 9313 | 0.673693471 | -0.224776929 | 6.055448914 | 2.86024E-06 | 0.00014096 | 4.653556563 |
| LOC643284 | 643284 | -0.566135265 | 0.015407045 | -6.053619043 | 2.87318E-06 | 0.000141287 | 4.649140381 |
| LOC644033 | 644033 | -0.679508461 | -0.067748853 | -6.0534971 | 2.87404E-06 | 0.000141287 | 4.648846073 |
| LOC650009 | 650009 | 0.794317865 | -0.009114738 | 6.051558322 | 2.88783E-06 | 0.000141788 | 4.64416667 |
| CD96 | 10225 | 0.734747283 | -0.133565831 | 6.050002204 | 2.89894E-06 | 0.000142156 | 4.640410575 |
| LOC650677 | 650677 | -0.618406013 | 0.136520256 | -6.046878374 | 2.92137E-06 | 0.000142978 | 4.632869679 |
| HRASLS3 | 11145 | -0.618743982 | 0.123068448 | -6.046658037 | 2.92296E-06 | 0.000142978 | 4.632337751 |
| LOC652379 | 652379 | 1.253228629 | -0.17802708 | 6.044342157 | 2.93972E-06 | 0.00014362 | 4.626746562 |
| LOC652202 | 652202 | 0.738822339 | -0.143359136 | 6.043712535 | 2.94429E-06 | 0.000143665 | 4.625226385 |
| FAM153A | 285596 | 1.382489479 | -0.10684758 | 6.033286328 | 3.02106E-06 | 0.000146799 | 4.60004735 |
| SOCS5 | 9655 | -1.245214332 | 0.1094494 | -6.033226076 | 3.02151E-06 | 0.000146799 | 4.59990181 |
| LOC649112 | 649112 | -0.65395019 | -0.249494272 | -6.033084941 | 3.02257E-06 | 0.000146799 | 4.599560898 |
| IQCC | 55721 | -1.166272807 | 0.226284896 | -6.032973521 | 3.0234E-06 | 0.000146799 | 4.599291761 |
| LOC645099 | 645099 | -0.754251385 | 0.179333133 | -6.031254416 | 3.03626E-06 | 0.000147242 | 4.595139082 |
| CYP7A1 | 1581 | -0.92289005 | 0.16685301 | -6.02599901 | 3.07593E-06 | 0.000148799 | 4.582442289 |
| HNRPK | 3190 | -0.484898323 | 0.055885023 | -6.019396443 | 3.12651E-06 | 0.000150876 | 4.56648698 |
| NPAL3 | 57185 | -0.592880278 | 0.09179013 | -6.017829433 | 3.13864E-06 | 0.000151276 | 4.562699625 |
| LOC645012 | 645012 | 0.552897668 | -0.046700841 | 6.01474852 | 3.16263E-06 | 0.000152172 | 4.555252569 |
| LOC285047 | 285047 | -0.687629926 | 0.139034666 | -6.014452116 | 3.16495E-06 | 0.000152172 | 4.554536065 |
| PCDHGA2 | 56113 | -0.656312671 | 0.125936674 | -6.012856714 | 3.17745E-06 | 0.000152552 | 4.550679316 |
| PTP4A2 | 8073 | 0.828912639 | -0.167055736 | 6.012457427 | 3.18059E-06 | 0.000152552 | 4.549714035 |
| SCAND2 | 54581 | 1.184463123 | -0.256006286 | 6.010373739 | 3.19701E-06 | 0.000153154 | 4.544676439 |
| NLRP12 | 91662 | 1.152364186 | -0.085911406 | 6.009283906 | 3.20564E-06 | 0.00015338 | 4.542041453 |
| SLC35F3 | 148641 | 0.742161766 | -0.134013988 | 6.006621411 | 3.2268E-06 | 0.000154206 | 4.535603615 |
| WIPF1 | 7456 | -0.786387971 | 0.126472239 | -6.001323943 | 3.26934E-06 | 0.000155861 | 4.522792441 |
| TYROBP | 7305 | -1.192862621 | 0.164790679 | -6.000731146 | 3.27414E-06 | 0.000155901 | 4.521358676 |
| TTTY7 | 246122 | 1.277874157 | -0.122249928 | 5.998550169 | 3.29185E-06 | 0.000156449 | 4.516083375 |
| LOC650254 | 650254 | 1.107265058 | -0.185410315 | 5.998336636 | 3.29358E-06 | 0.000156449 | 4.51556686 |
| NOTCH2 | 4853 | -0.882103842 | 0.167161395 | -5.996666067 | 3.30722E-06 | 0.000156908 | 4.511525777 |
| LOC648304 | 648304 | -1.231511139 | 0.346166956 | -5.994600976 | 3.32415E-06 | 0.000157522 | 4.50652998 |
| P4HB | 5034 | 0.801213086 | -0.176445099 | 5.991702328 | 3.34807E-06 | 0.000158275 | 4.499516974 |
| LOC650853 | 650853 | -1.063802748 | 0.158472561 | -5.991112032 | 3.35297E-06 | 0.000158316 | 4.498088706 |
| LOC649184 | 649184 | 0.834147882 | -0.226588647 | 5.985489817 | 3.39993E-06 | 0.000160321 | 4.484483656 |
| LOC647315 | 647315 | 1.451154565 | -0.04939049 | 5.98396771 | 3.41276E-06 | 0.000160528 | 4.480799825 |
| SLC7A6OS | 84138 | 0.711493414 | -0.085475529 | 5.98301817 | 3.42078E-06 | 0.000160555 | 4.478501619 |
| SNAPC1 | 6617 | 0.979654668 | -0.035676645 | 5.982541583 | 3.42482E-06 | 0.000160555 | 4.477348086 |
| WHAMML1 | 339005 | 0.802907126 | -0.031912109 | 5.981245866 | 3.43582E-06 | 0.000160792 | 4.474211815 |
| MAP4K1 | 11184 | 0.599227917 | 0.032748299 | 5.980987 | 3.43802E-06 | 0.000160792 | 4.473585214 |
| NEDD4L | 23327 | 1.277676843 | -0.161113461 | 5.979138917 | 3.45378E-06 | 0.000161337 | 4.469111623 |
| MXRA7 | 439921 | 0.556140638 | -0.059265591 | 5.977924237 | 3.46418E-06 | 0.000161444 | 4.466171112 |
| NAT15 | 79903 | 0.497676683 | -0.128987763 | 5.977916374 | 3.46425E-06 | 0.000161444 | 4.466152076 |
| LOC644635 | 644635 | 0.570812125 | -0.093227462 | 5.97546646 | 3.48532E-06 | 0.000162167 | 4.460220861 |
| OR4C16 | 219428 | -0.623171463 | -0.136344952 | -5.975155796 | 3.488E-06 | 0.000162167 | 4.459468707 |
| LOC642948 | 642948 | 0.571056373 | -0.110808224 | 5.970222955 | 3.53085E-06 | 0.000163661 | 4.447524466 |
| TNFRSF19 | 55504 | -0.886295365 | 0.187086391 | -5.970026275 | 3.53257E-06 | 0.000163661 | 4.447048184 |
| TCF19 | 6941 | -2.185638933 | -0.247126466 | -5.967430646 | 3.55535E-06 | 0.000164522 | 4.440762221 |
| COX19 | 90639 | -0.584575864 | 0.035970953 | -5.965337188 | 3.57383E-06 | 0.00016515 | 4.435691923 |
| GMCL1 | 64395 | -0.85137983 | 0.269466316 | -5.964625074 | 3.58013E-06 | 0.00016515 | 4.433967106 |
| LOC440396 | 440396 | -0.483054652 | -0.070739412 | -5.96440047 | 3.58212E-06 | 0.00016515 | 4.433423083 |
| NDUFS3 | 4722 | -0.779388915 | 0.20228514 | -5.964000396 | 3.58568E-06 | 0.00016515 | 4.432454031 |
| LHX9 | 56956 | 0.484148542 | 0.031875769 | 5.962745525 | 3.59684E-06 | 0.000165471 | 4.429414408 |
| DAPK1 | 1612 | -0.744818852 | 0.143538174 | -5.961545222 | 3.60754E-06 | 0.00016577 | 4.426506825 |
| KRI1 | 65095 | 0.653962438 | -0.213435137 | 5.959008937 | 3.63028E-06 | 0.000166567 | 4.42036254 |
| MRPL15 | 29088 | 0.981393577 | 0.134041427 | 5.957152119 | 3.64701E-06 | 0.000167 | 4.415863912 |
| EPR1 | 8475 | 0.887900055 | -0.106210691 | 5.954522637 | 3.67084E-06 | 0.000167714 | 4.409492747 |
| LIN28 | 79727 | 1.421688455 | -0.093154329 | 5.954493231 | 3.67111E-06 | 0.000167714 | 4.409421493 |
| KIAA1026 | 23254 | -1.424823039 | 0.031037073 | -5.952277328 | 3.69132E-06 | 0.000168442 | 4.404051905 |
| CEACAM6 | 4680 | 1.251857597 | 0.040445824 | 5.951334374 | 3.69995E-06 | 0.000168641 | 4.401766793 |
| LOC645712 | 645712 | -1.420138369 | 0.135616435 | -5.947433296 | 3.73589E-06 | 0.000169886 | 4.392312205 |
| LOC375748 | 375748 | 0.577654572 | 0.138109789 | 5.946606973 | 3.74355E-06 | 0.000170038 | 4.390309359 |
| ASPHD1 | 253982 | 0.502889449 | -0.0822147 | 5.94397673 | 3.76803E-06 | 0.000170752 | 4.383933732 |
| CBX2 | 84733 | 1.435985119 | -0.014842932 | 5.943646267 | 3.77112E-06 | 0.000170752 | 4.383132655 |
| RNF213 | 57674 | -0.677221211 | -0.051924477 | -5.943523165 | 3.77227E-06 | 1.71E-04 | 4.38283424 |
| KIAA1409 | 57578 | 0.883497419 | -0.187326205 | 5.940601871 | 3.79968E-06 | 0.000171785 | 4.375752227 |
| COMTD1 | 118881 | -1.008489635 | 0.22946989 | -5.940165964 | 3.80379E-06 | 0.000171785 | 4.374695401 |
| C7orf28B | 221960 | 0.943809231 | -0.149335243 | 5.939020386 | 3.8146E-06 | 0.000172076 | 4.371917943 |
| GRPEL2 | 134266 | 0.593615226 | -0.095591744 | 5.938506283 | 3.81947E-06 | 0.000172099 | 4.370671459 |
| SEC15L2 | 23233 | 1.248958591 | -0.098255346 | 5.937507391 | 3.82894E-06 | 0.000172328 | 4.368249492 |
| LOC389787 | 389787 | -0.54462296 | -0.003825533 | -5.93679927 | 3.83566E-06 | 0.000172401 | 4.366532488 |
| IL1RN | 3557 | 0.785480179 | -0.183409423 | 5.936417175 | 3.8393E-06 | 0.000172401 | 4.36560599 |
| LOC440402 | 440402 | 1.537119654 | 0.009423997 | 5.935905114 | 3.84417E-06 | 0.000172424 | 4.364364331 |
| C15orf29 | 79768 | -1.276612259 | 0.217551964 | -5.93466854 | 3.85598E-06 | 0.000172757 | 4.361365754 |
| C2orf40 | 84417 | -0.839472272 | 0.163664028 | -5.929510702 | 3.90561E-06 | 0.000174781 | 4.348856933 |
| RBMS1 | 5937 | 0.638381064 | -0.120041428 | 5.922890112 | 3.97026E-06 | 0.000177473 | 4.332797002 |
| SCN9A | 6335 | -0.927690431 | 0.210178098 | -5.921071819 | 3.98821E-06 | 0.000178074 | 4.328385555 |
| GREB1 | 9687 | -1.136877973 | 0.150214247 | -5.920610617 | 3.99278E-06 | 0.000178076 | 4.327266562 |
| LOC642762 | 642762 | 0.531589047 | -0.000432073 | 5.899036741 | 4.21237E-06 | 0.000187023 | 4.274900909 |
| ZNF831 | 128611 | 2.193087315 | 0.247623986 | 5.896474885 | 4.23924E-06 | 0.000188005 | 4.268679747 |
| ARFGAP1 | 55738 | 0.737770503 | -0.206540514 | 5.893843148 | 4.26704E-06 | 0.000188875 | 4.262288262 |
| MGAM | 8972 | 1.274804531 | -0.105832551 | 5.893710896 | 4.26844E-06 | 1.89E-04 | 4.261967056 |
| POT1 | 25913 | 0.890602131 | -0.134078547 | 5.891935884 | 4.28729E-06 | 0.000189496 | 4.257655848 |
| RAP1A | 5906 | 0.583861058 | -0.049252963 | 5.888313909 | 4.32603E-06 | 0.00019053 | 4.248857789 |
| GGNBP2 | 79893 | -1.028301214 | 0.162474735 | -5.888252611 | 4.32669E-06 | 0.00019053 | 4.248708883 |
| ADAM8 | 101 | 0.893597974 | -0.062122748 | 5.888087432 | 4.32847E-06 | 0.00019053 | 4.248307618 |
| ANKRD36 | 375248 | -1.14161797 | 0.136753556 | -5.887944099 | 4.33001E-06 | 0.00019053 | 4.247959426 |
| LOC650594 | 650594 | 0.64696958 | -0.15916218 | 5.88508081 | 4.36091E-06 | 0.000191676 | 4.241003331 |
| PRKAR2A | 5576 | 0.510603638 | -0.016064339 | 5.882172808 | 4.39253E-06 | 0.000192728 | 4.233937849 |
| PSAP | 5660 | 0.568541267 | -0.1892477 | 5.881980174 | 4.39463E-06 | 0.000192728 | 4.233469785 |
| FGF9 | 2254 | 1.095734186 | -0.08435451 | 5.877511171 | 4.4437E-06 | 0.000194664 | 4.222610014 |
| MTCH2 | 23788 | -0.890717781 | 0.144378732 | -5.876521281 | 4.45464E-06 | 0.000194926 | 4.220204316 |
| KAAG1 | 353219 | -1.839256346 | 0.067335612 | -5.871325984 | 4.51252E-06 | 0.00019724 | 4.207576903 |
| PRMT5 | 10419 | 0.477490124 | -0.069215764 | 5.868193843 | 4.54779E-06 | 0.000198342 | 4.199962912 |
| CNOT8 | 9337 | -0.742358332 | 0.129943686 | -5.862685387 | 4.61049E-06 | 0.000200854 | 4.186570143 |
| ISG20 | 3669 | -1.154498136 | 0.169163968 | -5.858163158 | 4.66262E-06 | 0.000202901 | 4.175573167 |
| KIAA0692 | 23141 | 0.611075786 | -0.161341644 | 5.364391448 | 1.60831E-05 | 0.000491279 | 2.965082148 |
| LOC100008589 | 100008589 | -1.073277879 | 0.349994167 | -5.855257526 | 4.69643E-06 | 0.000203698 | 4.168506403 |
| CNTNAP1 | 8506 | -1.031905423 | 0.197709279 | -5.853728256 | 4.71433E-06 | 0.000204249 | 4.164786777 |
| LOC283174 | 283174 | -0.878798737 | 0.152746631 | -5.84789536 | 4.78322E-06 | 0.000207007 | 4.150597572 |
| ZNF230 | 7773 | -1.316987061 | 0.029943319 | -5.845810071 | 4.80809E-06 | 0.00020776 | 4.145524127 |
| LOC646178 | 646178 | 1.113873087 | -0.110549807 | 5.845158979 | 4.81589E-06 | 0.00020776 | 4.143939962 |
| C4orf6 | 10141 | -0.811276608 | 0.183914789 | -5.845114113 | 4.81643E-06 | 0.00020776 | 4.143830796 |
| PXMP2 | 5827 | -1.247704949 | 0.020643956 | -5.840810829 | 4.86827E-06 | 0.000209542 | 4.133359556 |
| KIAA1530 | 57654 | 0.660498529 | -0.007083007 | 5.840802041 | 4.86837E-06 | 0.000209542 | 4.133338171 |
| LOC441956 | 441956 | -0.865040604 | 0.218538863 | -5.839446081 | 4.88483E-06 | 0.000210021 | 4.130038355 |
| LOC730092 | 730092 | 0.689242433 | -0.162539608 | 5.835166415 | 4.93713E-06 | 0.000212038 | 4.119622446 |
| SLC28A1 | 9154 | -1.170485751 | 0.13867008 | -5.834291434 | 4.94789E-06 | 0.000212269 | 4.117492707 |
| RGS22 | 26166 | 0.828543371 | -0.061749045 | 5.82784022 | 5.02798E-06 | 0.000215471 | 4.101788112 |
| TTC19 | 54902 | -0.718966279 | 0.126256281 | -5.827292147 | 5.03485E-06 | 0.000215531 | 4.100453735 |
| TMEM86B | 255043 | 0.570374425 | 0.090278121 | 5.826605696 | 5.04346E-06 | 0.000215665 | 4.098782418 |
| PLEC1 | 5339 | 0.766265474 | -0.156430614 | 5.824915854 | 5.06472E-06 | 0.000216197 | 4.094667943 |
| CHD6 | 84181 | -0.729137234 | 0.157807458 | -5.824745887 | 5.06686E-06 | 0.000216197 | 4.094254088 |
| LAT2 | 7462 | -1.169617436 | 0.249312709 | -5.823357682 | 5.0844E-06 | 0.000216503 | 4.09087384 |
| LOC645402 | 645402 | -0.537844366 | 0.044303623 | -5.82330945 | 5.08501E-06 | 0.000216503 | 4.090756394 |
| LOC401357 | 401357 | -0.588678784 | 0.010225919 | -5.820906617 | 5.11553E-06 | 0.000217567 | 4.084905142 |
| SFRS12 | 140890 | 1.085623601 | -0.047728976 | 5.819465705 | 5.13391E-06 | 0.000218114 | 4.081396069 |
| SLC44A1 | 23446 | 0.709920806 | -0.179222703 | 5.817619166 | 5.15758E-06 | 0.000218883 | 4.076898901 |
| STAB2 | 55576 | 0.559214589 | -0.147302487 | 5.816718575 | 5.16916E-06 | 0.000219139 | 4.074705442 |
| SEC24D | 9871 | 0.518333137 | -0.072003868 | 5.815793369 | 5.18108E-06 | 0.000219408 | 4.072451957 |
| LOC643100 | 643100 | 0.954729396 | -0.140271399 | 5.814164977 | 5.20213E-06 | 0.000220064 | 4.068485573 |
| HTR1F | 3355 | 1.371685176 | -0.229227039 | 5.81340888 | 5.21194E-06 | 0.000220242 | 4.066643818 |
| RFC4 | 5984 | 0.616274371 | -0.17262311 | 5.811923487 | 5.23126E-06 | 0.000220822 | 4.063025447 |
| C14orf72 | 145200 | -0.633231875 | 0.149715582 | -5.81009259 | 5.25517E-06 | 0.000221431 | 4.058565175 |
| PEG10 | 23089 | 0.845624922 | -0.113932543 | 5.809958236 | 5.25693E-06 | 0.000221431 | 4.058237864 |
| FPR2 | 2358 | -0.698395629 | 0.208952782 | -5.808598047 | 5.27477E-06 | 0.000221946 | 4.054924087 |
| KIAA0947 | 23379 | -0.68920317 | -0.020069621 | -5.807907002 | 5.28386E-06 | 0.000222091 | 4.053240457 |
| SCAMP1 | 9522 | -1.007749139 | -0.101138766 | -5.805354453 | 5.31757E-06 | 0.000223032 | 4.047021195 |
| TIAL1 | 7073 | -0.683662928 | 0.172893346 | -5.803518727 | 5.34194E-06 | 0.000223713 | 4.042548116 |
| LOC644241 | 644241 | -0.70010665 | 0.148928482 | -5.803278945 | 5.34514E-06 | 0.000223713 | 4.041963825 |
| LOC652067 | 652067 | 0.47619226 | 0.106734208 | 5.801879696 | 5.3638E-06 | 0.000224256 | 4.038554078 |
| WTAP | 9589 | -0.72169165 | 0.204524027 | -5.800405453 | 5.38354E-06 | 0.000224843 | 4.034961398 |
| LOC651614 | 651614 | -3.080290954 | -0.354915672 | -5.79989159 | 5.39044E-06 | 0.000224893 | 4.033709089 |
| LOC653082 | 653082 | 0.700859401 | -0.155702259 | 5.798571014 | 5.40821E-06 | 0.000225395 | 4.030490676 |
| LOC651850 | 651850 | -1.14093226 | 0.270715762 | -5.798107232 | 5.41446E-06 | 0.000225418 | 4.029360344 |
| EPHA6 | 285220 | -1.360111586 | 0.115452423 | -5.797086203 | 5.42826E-06 | 0.000225754 | 4.026871822 |
| PACRG | 135138 | -1.104130551 | 0.205075168 | -5.79534269 | 5.4519E-06 | 0.000226459 | 4.022622204 |
| ADAR | 103 | 0.756369392 | 0.036699189 | 5.794913068 | 5.45774E-06 | 0.000226459 | 4.021575008 |
| TGM2 | 7052 | 0.657316001 | -0.140472905 | 5.791824727 | 5.49991E-06 | 0.000227773 | 4.014046769 |
| RNF126 | 55658 | -1.112963241 | -0.489326883 | -5.790600224 | 5.51672E-06 | 0.000228229 | 4.011061657 |
| NBPF20 | 400818 | 0.942645615 | -0.041825105 | 5.789711037 | 5.52896E-06 | 0.000228332 | 4.008893902 |
| ZAP70 | 7535 | -0.693071658 | 0.163743649 | -5.788857646 | 5.54074E-06 | 0.000228503 | 4.00681335 |
| OSBPL6 | 114880 | 0.631441209 | -0.0964749 | 5.780428421 | 5.65842E-06 | 0.000233113 | 3.986259726 |
| LGMN | 5641 | 0.806329936 | 0.077372276 | 5.779367598 | 5.67341E-06 | 0.000233486 | 3.983672612 |
| PNPLA4 | 8228 | -0.627085976 | 0.114934383 | -5.778558468 | 5.68486E-06 | 0.000233714 | 3.981699259 |
| LOC643866 | 643866 | -0.736298945 | 0.153431077 | -5.775663325 | 5.72606E-06 | 0.000234917 | 3.974637958 |
| SLC23A2 | 9962 | 0.504121637 | -0.098261439 | 5.771554674 | 5.78504E-06 | 0.000236844 | 3.964615675 |
| MGC72104 | 284802 | -0.664370632 | 0.017093628 | -5.769713269 | 5.81168E-06 | 0.000237688 | 3.960123452 |
| BARX1 | 56033 | -0.942904147 | 0.197361198 | -5.768982013 | 5.82229E-06 | 0.000237875 | 3.958339427 |
| LOC643346 | 643346 | 0.731486589 | -0.089981687 | 5.768256162 | 5.83284E-06 | 0.00023806 | 3.956568544 |
| LOC389111 | 389111 | 1.042120281 | -0.148211679 | 5.764125602 | 5.89326E-06 | 0.000240277 | 3.946490239 |
| CORO2B | 10391 | 0.597203043 | -0.086171926 | 5.759980665 | 5.95453E-06 | 0.000242524 | 3.936375415 |
| MESP1 | 55897 | -0.877688517 | 0.207059309 | -5.75869582 | 5.97365E-06 | 0.000243052 | 3.933239737 |
| AHNAK | 79026 | 0.775158077 | -0.08482096 | 5.755034459 | 6.02849E-06 | 0.000244676 | 3.92430339 |
| LOC641848 | 641848 | 0.563990466 | -0.069344488 | 5.754920435 | 6.0302E-06 | 0.000244676 | 3.92402507 |
| HES6 | 55502 | -0.952027526 | 0.208276805 | -5.754788319 | 6.03219E-06 | 0.000244676 | 3.92370259 |
| LOC284672 | 284672 | 0.645920469 | -0.15711573 | 5.75414524 | 6.04188E-06 | 0.000244817 | 3.922132887 |
| GRIK2 | 2898 | 1.070954305 | -0.149376512 | 5.752255611 | 6.07045E-06 | 0.000245722 | 3.917520258 |
| LOC285735 | 285735 | 0.983952552 | 0.000260847 | 5.750038725 | 6.10413E-06 | 0.000246832 | 3.912108411 |
| BCCIP | 56647 | -0.525589021 | 0.090468292 | -5.74321107 | 6.20908E-06 | 0.000250819 | 3.89543822 |
| POLR3H | 171568 | 1.163257729 | -0.157631047 | 5.741958132 | 6.22854E-06 | 0.000251347 | 3.892378668 |
| LOC652742 | 652742 | 0.690950052 | -0.116559268 | 5.741263166 | 6.23935E-06 | 0.000251526 | 3.890681573 |
| ERCC6 | 2074 | -0.675312561 | 0.106554914 | -5.740014477 | 6.25884E-06 | 0.000252054 | 3.887632193 |
| LOC643493 | 643493 | 1.028896424 | -0.18533115 | 5.738872352 | 6.27672E-06 | 0.000252363 | 3.884842938 |
| LOC646782 | 646782 | 1.661421217 | -0.098822655 | 5.738483238 | 6.28282E-06 | 0.000252363 | 3.883892634 |
| LOC651714 | 651714 | -0.758041022 | 0.047381352 | -5.738299443 | 6.2857E-06 | 0.000252363 | 3.883443762 |
| NDUFA4L2 | 56901 | -0.823926055 | 0.195830241 | -5.733907182 | 6.35504E-06 | 0.000254628 | 3.872715941 |
| GFOD1 | 54438 | -1.012956398 | -0.116718995 | -5.731946332 | 6.38624E-06 | 0.000255383 | 3.867926178 |
| C22orf9 | 23313 | -0.75508808 | -0.25296272 | -5.731908728 | 6.38684E-06 | 0.000255383 | 3.867834318 |
| HOXD10 | 3236 | -0.874341711 | -0.401956144 | -5.729240706 | 6.42955E-06 | 0.000256672 | 3.861316634 |
| TNRC18 | 27320 | -1.312561117 | -0.108270335 | -5.72908231 | 6.4321E-06 | 0.000256672 | 3.860929671 |
| LOC643320 | 643320 | 0.635919438 | 0.029979209 | 5.726313609 | 6.47674E-06 | 0.000258192 | 3.854165386 |
| KIAA0556 | 23247 | 0.582021716 | -0.122112933 | 5.725534321 | 6.48936E-06 | 0.000258434 | 3.852261374 |
| EDG1 | 1901 | 1.717731729 | -0.02463141 | 5.719584193 | 6.58657E-06 | 0.00026204 | 3.837721975 |
| LCAT | 3931 | 0.618965627 | -0.141170236 | 5.719018939 | 6.59588E-06 | 0.000262146 | 3.836340601 |
| F9 | 2158 | -0.761029085 | 0.183467634 | -5.717317806 | 6.62398E-06 | 0.000262998 | 3.832183201 |
| TMX4 | 56255 | -0.659364195 | 0.141545326 | -5.716442767 | 6.63848E-06 | 0.000263309 | 3.830044599 |
| NECAB3 | 63941 | -2.13657452 | -0.487274448 | -5.711697471 | 6.71769E-06 | 0.000266183 | 3.818445993 |
| ARL5A | 26225 | -0.716768348 | 0.150352177 | -5.711154137 | 6.72682E-06 | 0.000266277 | 3.817117842 |
| C3orf35 | 339883 | -0.732241931 | 0.1168627 | -5.709841504 | 6.74893E-06 | 0.000266884 | 3.813909084 |
| BTC | 685 | -0.759440026 | 0.200080658 | -5.705231847 | 6.82716E-06 | 0.000269707 | 3.802639582 |
| KYNU | 8942 | -1.239562809 | 0.13683404 | -5.70363908 | 6.8544E-06 | 0.000270512 | 3.798745253 |
| GDF5 | 8200 | 1.314648208 | -0.051177756 | 5.702566924 | 6.8728E-06 | 0.000270967 | 3.796123708 |
| B3GALT5 | 10317 | -0.756162185 | 0.138516557 | -5.701063593 | 6.89868E-06 | 0.000271716 | 3.792447734 |
| C1QB | 713 | -0.61909644 | 0.019537321 | -5.69999873 | 6.91708E-06 | 0.000272063 | 3.789843802 |
| PCDHA4 | 56144 | 0.848511463 | 0.059997901 | 5.699373606 | 6.9279E-06 | 0.000272063 | 3.788315132 |
| SMYD4 | 114826 | -1.34397756 | 0.150097033 | -5.699356392 | 6.9282E-06 | 0.000272063 | 3.788273036 |
| GPR110 | 266977 | 0.689541849 | -0.116586049 | 5.698115749 | 6.94973E-06 | 0.000272366 | 3.785239088 |
| LOC731444 | 731444 | 0.751659874 | -0.144442963 | 5.695363668 | 6.99773E-06 | 0.000273975 | 3.778508534 |
| HSD17B11 | 51170 | 1.139372064 | -0.155716185 | 5.692279543 | 7.05192E-06 | 0.000275822 | 3.770965206 |
| OSAP | 84709 | 1.005075455 | -0.118823614 | 5.691033393 | 7.07393E-06 | 0.000276409 | 3.767917088 |
| STX1A | 6804 | 0.815607428 | -0.170393809 | 5.689282005 | 7.105E-06 | 0.000277041 | 3.763632936 |
| CORO1C | 23603 | -0.680102593 | 0.164418059 | -5.688933842 | 7.11119E-06 | 0.000277041 | 3.762781249 |
| SRD5A2 | 6716 | -1.196644289 | 0.145334244 | -5.687504339 | 7.13666E-06 | 0.000277485 | 3.759284253 |
| APBB2 | 323 | -0.827468794 | 0.130272995 | -5.683753509 | 7.20395E-06 | 0.000279826 | 3.750107826 |
| HOOK1 | 51361 | -0.781676516 | 0.028490624 | -5.682766711 | 7.22176E-06 | 0.000280241 | 3.747693435 |
| KRT82 | 3888 | -1.061916414 | 0.204874961 | -5.681907863 | 7.2373E-06 | 0.000280419 | 3.745592035 |
| C21orf100 | 118421 | 0.496818033 | -0.063383718 | 5.681726855 | 7.24058E-06 | 0.000280419 | 3.745149143 |
| KIAA1147 | 57189 | 1.070322947 | -0.201461275 | 5.680348862 | 7.26559E-06 | 0.000280999 | 3.741777377 |
| C5orf62 | 85027 | 1.563547671 | -0.088603116 | 5.679963975 | 7.27259E-06 | 0.000280999 | 3.740835583 |
| MANEA | 79694 | 0.487766281 | -0.019925236 | 5.679725478 | 7.27693E-06 | 0.000280999 | 3.740251991 |
| PLCH1 | 23007 | 0.514121887 | -0.122014747 | 5.679191963 | 7.28665E-06 | 0.000281099 | 3.738946483 |
| LOC441294 | 441294 | 0.437597487 | 0.086042606 | 5.674225637 | 7.37778E-06 | 0.000284337 | 3.72679285 |
| SNORD4A | 26773 | 0.916543778 | -0.186091391 | 5.671850072 | 7.42178E-06 | 0.000285753 | 3.720978666 |
| ZNF189 | 7743 | 1.240467509 | -0.05851492 | 5.670399113 | 7.44879E-06 | 0.000286513 | 3.717427231 |
| FLI1 | 2313 | -0.606339099 | 0.134354352 | -5.669968572 | 7.45682E-06 | 0.000286543 | 3.716373387 |
| HIGD1A | 25994 | -0.656209392 | 0.118561341 | -5.66691153 | 7.51411E-06 | 0.000288226 | 3.708890193 |
| C20orf24 | 55969 | 0.457342647 | -0.041274373 | 5.666850627 | 7.51525E-06 | 0.000288226 | 3.708741104 |
| RFWD2 | 64326 | 1.055746124 | -0.147037762 | 5.664670753 | 7.55638E-06 | 0.000289379 | 3.703404636 |
| BRWD1 | 54014 | -0.73443788 | 0.078674963 | -5.664479407 | 7.56E-06 | 0.000289379 | 3.70293619 |
| DCHS2 | 54798 | 0.504593017 | -0.115899722 | 5.6632949 | 7.58246E-06 | 0.000289957 | 3.700036272 |
| MRPS12 | 6183 | -0.920837747 | 0.206134453 | -5.661242118 | 7.62153E-06 | 0.000291169 | 3.695010375 |
| FLRT3 | 23767 | 0.549326581 | -0.137744248 | 5.660440417 | 7.63685E-06 | 0.000291472 | 3.693047453 |
| DHX57 | 90957 | -0.581925699 | -0.032010512 | -5.659680357 | 7.6514E-06 | 0.000291745 | 3.691186442 |
| SSX5 | 6758 | 0.831711894 | -0.067138517 | 5.652571238 | 7.78884E-06 | 0.000296412 | 3.673777567 |
| TMEM151A | 256472 | 0.862226403 | -0.139848346 | 5.651989748 | 7.8002E-06 | 0.000296558 | 3.672353437 |
| GAS1 | 2619 | -0.874028263 | 0.240038422 | -5.650337027 | 7.83256E-06 | 0.000297215 | 3.668305608 |
| FLJ42133 | 400844 | 0.889354274 | -0.173509898 | 5.647716199 | 7.88415E-06 | 0.000298469 | 3.661886268 |
| PFDN1 | 5201 | -0.80032975 | 0.184127288 | -5.64749899 | 7.88844E-06 | 0.000298469 | 3.661354223 |
| ZSWIM3 | 140831 | 0.481003239 | 0.04428711 | 5.647156379 | 7.89521E-06 | 0.000298469 | 3.660515003 |
| SPINK9 | 643394 | 0.809090516 | -0.159366161 | 5.647122346 | 7.89589E-06 | 0.000298469 | 3.660431639 |
| JAZF1 | 221895 | -0.802732352 | 0.125338454 | -5.645287028 | 7.93228E-06 | 0.000299557 | 3.655935896 |
| AUTS2 | 26053 | 0.777590663 | -0.208148777 | 5.642707511 | 7.98371E-06 | 0.00030097 | 3.649616752 |
| SEC61A1 | 29927 | 0.641401311 | -0.172646325 | 5.640481465 | 8.02836E-06 | 0.000302317 | 3.64416311 |
| CYP26A1 | 1592 | -0.864640926 | 0.143385113 | -5.639081452 | 8.05658E-06 | 0.00030309 | 3.640732991 |
| HNF4A | 3172 | 1.053154056 | -0.049118917 | 5.638041596 | 8.0776E-06 | 0.000303591 | 3.638185185 |
| JPH1 | 56704 | -0.480038218 | -0.049941833 | -5.636688511 | 8.10503E-06 | 0.000304332 | 3.634869796 |
| IL2RB | 3560 | 0.951212424 | -0.186381987 | 5.635481172 | 8.12959E-06 | 0.000304899 | 3.631911404 |
| CDC42SE2 | 56990 | 0.429556405 | -0.075697094 | 5.635045957 | 8.13847E-06 | 0.000304899 | 3.63084495 |
| CADM4 | 199731 | 0.881331439 | -0.178500331 | 5.634808537 | 8.14331E-06 | 0.000304899 | 3.630263168 |
| C19orf31 | 404664 | 0.901241459 | -0.22190284 | 5.632628534 | 8.18793E-06 | 0.000306278 | 3.624921027 |
| LOC731777 | 731777 | 1.280873217 | -0.054783874 | 5.630632843 | 8.22899E-06 | 0.000307522 | 3.620030229 |
| LOC730027 | 730027 | -0.546961416 | -0.015178153 | -5.62980203 | 8.24614E-06 | 0.000307872 | 3.617994085 |
| C11orf61 | 79684 | 0.745385544 | -0.153247303 | 5.628469059 | 8.27374E-06 | 0.000308403 | 3.614727151 |
| MAGEA6 | 4105 | 0.911984588 | -0.156868259 | 5.62835871 | 8.27603E-06 | 0.000308403 | 3.614456692 |
| SAMD11 | 148398 | 0.520576152 | -0.124721994 | 5.626516347 | 8.31434E-06 | 0.000309538 | 3.609941071 |
| KLK3 | 354 | 0.527729049 | -0.120493758 | 5.622764466 | 8.39292E-06 | 0.000311833 | 3.600744442 |
| DFNA5 | 1687 | 0.833373653 | -0.101915711 | 5.622442574 | 8.39969E-06 | 0.000311833 | 3.599955368 |
| MMP19 | 4327 | -0.730054336 | -0.213181703 | -5.621962055 | 8.40982E-06 | 0.000311915 | 3.59877743 |
| OR2L8 | 391190 | 0.531609472 | -0.016724973 | 5.621153199 | 8.42689E-06 | 0.000312254 | 3.596794571 |
| RBKS | 64080 | 0.607452624 | -0.068247078 | 5.620075564 | 8.44969E-06 | 0.000312805 | 3.594152742 |
| ZNF581 | 51545 | -0.963972737 | 0.211665329 | -5.618492208 | 8.48331E-06 | 0.000313461 | 3.590270977 |
| LPA | 4018 | 1.103952984 | -0.18262289 | 5.615279509 | 8.55193E-06 | 0.000315701 | 3.582394129 |
| F2R | 2149 | 0.536150236 | -0.137181597 | 5.614292783 | 8.57312E-06 | 0.000316187 | 3.579974736 |
| PGS1 | 9489 | 0.873042111 | -0.140171357 | 5.613598405 | 8.58806E-06 | 0.000316368 | 3.578272118 |
| ZNHIT2 | 741 | 0.744797967 | -0.096792891 | 5.612964906 | 8.60171E-06 | 0.000316368 | 3.576718745 |
| SOBP | 55084 | 0.483496509 | -0.103411693 | 5.612947561 | 8.60209E-06 | 0.000316368 | 3.576676213 |
| LARP4 | 113251 | -0.838547297 | 0.149240135 | -5.612311512 | 8.61582E-06 | 0.000316577 | 3.575116557 |
| SEC24B | 10427 | -0.581104104 | 0.119213581 | -5.610632378 | 8.65218E-06 | 0.000317027 | 3.570999004 |
| PPP2R5A | 5525 | 0.482886699 | -0.159791349 | 5.608560226 | 8.69727E-06 | 0.000318383 | 3.565917412 |
| OR5K3 | 403277 | -0.665778325 | 0.148982588 | -5.608153579 | 8.70615E-06 | 0.000318412 | 3.564920142 |
| PTRH2 | 51651 | 0.713400071 | -0.050427346 | 5.606145444 | 8.75012E-06 | 0.000319724 | 3.559995177 |
| LOC440145 | 440145 | 0.387846473 | -0.010769612 | 5.60489343 | 8.77764E-06 | 0.000320433 | 3.556924453 |
| SYCP1 | 6847 | 0.568069427 | -0.116485683 | 5.602509185 | 8.8303E-06 | 0.000321759 | 3.551076465 |
| ZFATAS | 594840 | 0.606448913 | -0.103848692 | 5.601594938 | 8.85058E-06 | 0.0003222 | 3.548833924 |
| CSNK2A2 | 1459 | -0.958866258 | 0.175022846 | -5.599989655 | 8.8863E-06 | 0.000323202 | 3.544896204 |
| BTBD11 | 121551 | -0.620585853 | 0.137960787 | -5.598428322 | 8.92118E-06 | 0.000324172 | 3.541066111 |
| RAPGEFL1 | 51195 | -1.330960172 | 0.16302106 | -5.597275975 | 8.94701E-06 | 0.000324416 | 3.538239183 |
| PLAT | 5327 | -0.591165477 | 0.123425315 | -5.596759065 | 8.95863E-06 | 0.000324416 | 3.536971072 |
| PSMD13 | 5719 | -0.694442413 | 0.160509189 | -5.596661463 | 8.96082E-06 | 0.000324416 | 3.536731627 |
| LOC644952 | 644952 | -0.583314877 | 0.072190916 | -5.595489844 | 8.9872E-06 | 0.000325073 | 3.53385727 |
| JPH2 | 57158 | 1.045563061 | -0.124409344 | 5.592695663 | 9.05045E-06 | 0.00032706 | 3.527001839 |
| SLC22A6 | 9356 | 0.463593146 | -0.046085164 | 5.592268525 | 9.06015E-06 | 0.000327111 | 3.52595382 |
| PAOX | 196743 | -0.691128438 | -0.2535922 | -5.589432441 | 9.12487E-06 | 0.000329147 | 3.518994914 |
| PRR7 | 80758 | 0.952192886 | -0.105783883 | 5.586799195 | 9.18538E-06 | 0.000331026 | 3.512533186 |
| RORC | 6097 | -0.958477775 | 0.154703879 | -5.581736084 | 9.30288E-06 | 0.000334955 | 3.500107398 |
| RNASE4 | 6038 | 0.534821091 | 0.039099966 | 5.579317951 | 9.35953E-06 | 0.000336687 | 3.49417221 |
| MKRN2 | 23609 | -0.770120813 | -0.041107679 | -5.576072553 | 9.43611E-06 | 0.000339132 | 3.486205881 |
| TFG | 10342 | 0.780974166 | -0.211753382 | 5.57430443 | 9.4781E-06 | 0.000340331 | 3.481865433 |
| ESF1 | 51575 | 1.076641907 | 0.288837299 | 5.57286393 | 9.51244E-06 | 0.000340757 | 3.478329077 |
| MYNN | 55892 | -1.532183791 | 0.101901739 | -5.57271998 | 9.51588E-06 | 0.000340757 | 3.47797568 |
| FANCB | 2187 | 0.761960701 | -0.119706304 | 5.572193496 | 9.52847E-06 | 0.000340829 | 3.476683145 |
| LOC643719 | 643719 | -0.887990733 | 0.176900356 | -5.571913339 | 9.53518E-06 | 0.000340829 | 3.475995344 |
| YTHDC1 | 91746 | 0.799837939 | -0.127768581 | 5.571495336 | 9.54519E-06 | 0.000340878 | 3.474969111 |
| GRIK1 | 2897 | 0.950708696 | -0.19516295 | 5.570669074 | 9.56502E-06 | 0.000341276 | 3.472940532 |
| TIRAP | 114609 | 1.222068888 | -0.169120253 | 5.567354458 | 9.64498E-06 | 0.000343818 | 3.464802236 |
| LPP | 4026 | 0.507571738 | 0.024700816 | 5.563024515 | 9.75045E-06 | 0.000346951 | 3.454169852 |
| HNF4G | 3174 | -0.911068767 | -0.188385905 | -5.562423731 | 9.76518E-06 | 0.000347161 | 3.452694492 |
| LOC644827 | 644827 | -1.024510188 | -0.060505467 | -5.559937135 | 9.82637E-06 | 0.000349022 | 3.446587831 |
| LOC642197 | 642197 | 0.862025702 | -0.084912349 | 5.559501253 | 9.83714E-06 | 0.00034909 | 3.445517332 |
| PCSK6 | 5046 | -0.979527417 | 0.209699675 | -5.556585592 | 9.90946E-06 | 0.000351077 | 3.438356314 |
| PPAP2A | 8611 | -0.732228326 | 0.147538387 | -5.555833728 | 9.9282E-06 | 0.000351326 | 3.436509599 |
| GFRA3 | 2676 | 0.584447223 | 0.025206967 | 5.555529259 | 9.9358E-06 | 0.000351326 | 3.435761754 |
| MIS12 | 79003 | 1.225475478 | -0.066572421 | 5.554557233 | 9.9601E-06 | 0.00035187 | 3.433374202 |
| NACC2 | 138151 | -1.143695883 | 0.260513963 | -5.553844267 | 9.97796E-06 | 0.000352185 | 3.431622925 |
| SLC27A1 | 376497 | 0.693906843 | -0.111467121 | 5.551461878 | 1.00379E-05 | 0.000353983 | 3.425770747 |
| SR140 | 23350 | 0.442046423 | -0.073140065 | 5.548079847 | 1.01235E-05 | 0.000356189 | 3.417462335 |
| TSC22D3 | 1831 | 0.608869339 | -0.124847732 | 5.54801501 | 1.01252E-05 | 0.000356189 | 3.417303045 |
| LOC648815 | 648815 | 0.479830814 | -0.069795912 | 5.547923776 | 1.01275E-05 | 0.000356189 | 3.417078905 |
| CACNA2D3 | 55799 | -0.784022613 | 0.222558494 | -5.547250404 | 1.01447E-05 | 0.000356474 | 3.415424574 |
| ZSCAN18 | 65982 | -1.05541501 | 0.150823447 | -5.546400052 | 1.01664E-05 | 0.000356919 | 3.413335394 |
| MGST1 | 4257 | 0.550159716 | -0.038358115 | 5.543717207 | 1.02352E-05 | 0.000359014 | 3.406743742 |
| CEPT1 | 10390 | 0.851849494 | -0.19367695 | 5.542232376 | 1.02734E-05 | 0.000360036 | 3.403095352 |
| FZD8 | 8325 | 0.86291354 | -0.227766151 | 5.541413039 | 1.02946E-05 | 0.000360458 | 3.401082086 |
| GALNT1 | 2589 | 0.459182961 | -0.022138632 | 5.540501571 | 1.03182E-05 | 0.000360799 | 3.398842383 |
| FAM129B | 64855 | 0.667193509 | -0.058664266 | 5.540233233 | 1.03252E-05 | 0.000360799 | 3.398182998 |
| KLHL17 | 339451 | -0.520663289 | 0.071563519 | -5.53962616 | 1.0341E-05 | 0.000360799 | 3.396691227 |
| CCNL1 | 57018 | 0.982174822 | -0.198162848 | 5.538109838 | 1.03805E-05 | 0.000361825 | 3.392965028 |
| LOC650280 | 650280 | 0.955228639 | -0.150736751 | 5.537792988 | 1.03887E-05 | 0.000361825 | 3.392186382 |
| LOC388564 | 388564 | -0.459958224 | -0.103092965 | -5.536281818 | 1.04283E-05 | 0.000362882 | 3.388472651 |
| GON4L | 54856 | -0.817172666 | 0.151471292 | -5.535437914 | 1.04504E-05 | 0.000363332 | 3.386398672 |
| CAMK2D | 817 | 0.615414337 | -0.098506767 | 5.533796946 | 1.04936E-05 | 0.000364512 | 3.382365688 |
| ZNF509 | 166793 | -0.666674239 | 0.202078524 | -5.531851458 | 1.05451E-05 | 0.000365656 | 3.37758405 |
| LOC401137 | 401137 | -1.09666154 | 0.33999705 | -5.530541282 | 1.05799E-05 | 0.000366533 | 3.374363739 |
| OR56B4 | 196335 | -0.659254906 | 0.135726202 | -5.53019973 | 1.0589E-05 | 0.000366533 | 3.373524212 |
| MX1 | 4599 | 1.462729536 | -0.132118503 | 5.529490514 | 1.06079E-05 | 0.000366865 | 3.371780947 |
| ACE | 1636 | 0.780259167 | -0.05126771 | 5.528679281 | 1.06295E-05 | 0.000367292 | 3.369786884 |
| IP6K2 | 51447 | 1.283890509 | -0.003290317 | 5.523718841 | 1.0763E-05 | 0.000371577 | 3.357592815 |
| ZNF318 | 24149 | -0.381117788 | -0.074717079 | -5.522216478 | 1.08037E-05 | 0.000372393 | 3.353899279 |
| MASP1 | 5648 | 0.796117856 | -0.145339427 | 5.522150409 | 1.08055E-05 | 0.000372393 | 3.353736845 |
| HIPK3 | 10114 | -0.675226245 | 0.163619708 | -5.520954434 | 1.08381E-05 | 0.000373189 | 3.350796445 |
| TDRD9 | 122402 | -0.88472325 | 0.216219399 | -5.519644945 | 1.08738E-05 | 0.000373767 | 3.347576849 |
| NR1I3 | 9970 | -0.797107472 | 0.189547191 | -5.517920435 | 1.09211E-05 | 0.000374995 | 3.34333668 |
| MAP3K4 | 4216 | 0.92204497 | -0.077485414 | 5.517340083 | 1.0937E-05 | 0.000374995 | 3.341909682 |
| FBXO32 | 114907 | -0.814213346 | -0.242485384 | -5.517302821 | 1.09381E-05 | 0.000374995 | 3.341818062 |
| KIF19 | 124602 | -0.679982444 | -0.091649842 | -5.516537666 | 1.09591E-05 | 0.000375065 | 3.339936627 |
| LOC730419 | 730419 | -0.640281162 | 0.045167044 | -5.513142705 | 1.10531E-05 | 0.000377238 | 3.331588298 |
| LOC653528 | 653528 | -0.928362053 | 0.096629695 | -5.512864119 | 1.10609E-05 | 0.000377238 | 3.330903213 |
| LOC400752 | 400752 | 1.035688724 | -0.172562932 | 5.509898447 | 1.11437E-05 | 0.000379735 | 3.323609831 |
| GLYATL2 | 219970 | 0.434277667 | -0.109034324 | 5.507839695 | 1.12016E-05 | 0.000381056 | 3.318546461 |
| AFG3L1 | 172 | -1.773807908 | 0.455118499 | -5.507832075 | 1.12018E-05 | 0.000381056 | 3.318527719 |
| DLL3 | 10683 | 0.811991138 | -0.036178992 | 5.506723503 | 1.12331E-05 | 0.000381791 | 3.315801139 |
| PLAG1 | 5324 | -0.502649595 | 0.011664244 | -5.505201147 | 1.12762E-05 | 0.000382794 | 3.312056707 |
| LRP2 | 4036 | -0.766107669 | 0.186290191 | -5.50465366 | 1.12918E-05 | 0.000382794 | 3.310710052 |
| BPTF | 2186 | 0.564181165 | -0.154436161 | 5.50230212 | 1.13588E-05 | 0.000384308 | 3.304925742 |
| LOC729692 | 729692 | -0.484174568 | 0.014228889 | -5.502062209 | 1.13657E-05 | 0.000384308 | 3.30433559 |
| NME7 | 29922 | -0.621975676 | 0.078160522 | -5.497472673 | 1.14977E-05 | 0.000388107 | 3.293045132 |
| SCN4B | 6330 | -0.995801065 | 0.068248974 | -5.494350958 | 1.15884E-05 | 0.000390834 | 3.285364779 |
| KLK5 | 25818 | 0.826154009 | -0.132410213 | 5.492969826 | 1.16288E-05 | 0.00039186 | 3.281966577 |
| MYBPHL | 343263 | 0.72486401 | -0.15841863 | 5.491686082 | 1.16664E-05 | 0.000392516 | 3.27880788 |
| TRPV2 | 51393 | 0.790465902 | -0.183818249 | 5.491626898 | 1.16682E-05 | 0.000392516 | 3.278662253 |
| NHS | 4810 | 0.887716348 | -0.107779077 | 5.490622154 | 1.16977E-05 | 0.000392976 | 3.276189964 |
| ZNF362 | 149076 | 0.886768721 | -0.175726933 | 5.490408394 | 1.1704E-05 | 0.000392976 | 3.275663977 |
| LOC653702 | 653702 | 1.264646207 | -0.222156643 | 5.490146425 | 1.17117E-05 | 0.000392976 | 3.275019355 |
| ARMC4 | 55130 | 0.973854392 | -0.210270236 | 5.488835153 | 1.17505E-05 | 0.000393745 | 3.271792682 |
| LOC648487 | 648487 | 0.851243009 | -0.037188687 | 5.488694199 | 1.17546E-05 | 0.000393745 | 3.271445824 |
| C8orf79 | 57604 | 1.101820042 | -0.097425464 | 5.484293509 | 1.18856E-05 | 0.000397795 | 3.260616089 |
| LOC643373 | 643373 | 0.582672849 | 0.057706751 | 5.483230484 | 1.19175E-05 | 0.00039821 | 3.257999881 |
| RIG | 10530 | -0.79284024 | 0.202393676 | -5.482885431 | 1.19278E-05 | 0.00039821 | 3.257150656 |
| LOC653238 | 653238 | 0.590425045 | -0.152331093 | 5.482868542 | 1.19283E-05 | 0.00039821 | 3.25710909 |
| LOC644056 | 644056 | -0.962604378 | -0.243535621 | -5.481774565 | 1.19612E-05 | 0.000398971 | 3.254416603 |
| C17orf55 | 284185 | 0.730056233 | -0.012623249 | 5.481282926 | 1.19761E-05 | 0.000399128 | 3.253206562 |
| LOC646226 | 646226 | 1.214148458 | 0.000792824 | 5.480664195 | 1.19947E-05 | 0.000399412 | 3.251683693 |
| P4HA2 | 8974 | 0.568244129 | -0.079432333 | 5.479766834 | 1.20219E-05 | 0.000399978 | 3.249474993 |
| LOC652062 | 652062 | 1.372628959 | -0.143325387 | 5.47816318 | 1.20705E-05 | 0.000401258 | 3.245527746 |
| IL17RD | 54756 | 0.936511208 | -0.126811949 | 5.475811216 | 1.21422E-05 | 0.000403302 | 3.2397383 |
| LNX1 | 84708 | 0.524076861 | 0.087899238 | 5.47537748 | 1.21555E-05 | 0.000403403 | 3.238670604 |
| LOC649456 | 649456 | 1.197486991 | -0.178889185 | 5.472871482 | 1.22325E-05 | 0.000405275 | 3.232501535 |
| TRERF1 | 55809 | 0.751910933 | -0.154470593 | 5.472328738 | 1.22492E-05 | 0.000405489 | 3.231165396 |
| LOC728734 | 728734 | 1.218361836 | 0.038082271 | 5.471302507 | 1.22809E-05 | 0.000406069 | 3.22863895 |
| FRMPD2 | 143162 | -0.893962758 | 0.183819934 | -5.471094903 | 1.22873E-05 | 0.000406069 | 3.228127846 |
| SLC35B4 | 84912 | 0.71949067 | -0.149942983 | 5.468934243 | 1.23544E-05 | 0.000407944 | 3.222808331 |
| LOC650494 | 650494 | 1.328571845 | -0.133518498 | 5.46852525 | 1.23671E-05 | 0.000408022 | 3.221801363 |
| MTX3 | 345778 | -0.675988169 | 0.076521126 | -5.465901253 | 1.24492E-05 | 0.000410385 | 3.215340647 |
| POLR3GL | 84265 | 0.732137721 | -0.166936839 | 5.4636891 | 1.25187E-05 | 0.000411571 | 3.209893619 |
| LOC730996 | 730996 | 1.193420966 | -0.040432785 | 5.463575412 | 1.25223E-05 | 0.000411571 | 3.209613673 |
| TRIM46 | 80128 | 0.538010997 | -0.102138954 | 5.463118087 | 1.25368E-05 | 0.000411571 | 3.208487552 |
| MAS1L | 116511 | -0.743440212 | -0.169386866 | -5.463100127 | 1.25373E-05 | 0.000411571 | 3.208443325 |
| SKA1 | 220134 | -0.871603793 | 0.177530228 | -5.457133159 | 1.27273E-05 | 0.000417112 | 3.193748964 |
| C14orf45 | 80127 | 1.196649093 | -0.162029466 | 5.456519692 | 1.2747E-05 | 0.000417298 | 3.192238102 |
| ASB4 | 51666 | 1.286158128 | 0.240150074 | 5.455993229 | 1.27639E-05 | 0.000417298 | 3.190941497 |
| NANOS2 | 339345 | 0.408914052 | -0.026065396 | 5.45596774 | 1.27647E-05 | 0.000417298 | 3.190878719 |
| DEFB137 | 613210 | -1.396672532 | 0.15401336 | -5.453698735 | 1.28379E-05 | 0.000419343 | 3.185290268 |
| PMS2L1 | 5379 | -1.080894593 | 0.193402843 | -5.452990382 | 1.28608E-05 | 0.000419745 | 3.183545563 |
| KDELR1 | 10945 | -0.606155527 | -0.135842685 | -5.451084029 | 1.29228E-05 | 0.000420872 | 3.17884997 |
| ECH1 | 1891 | 0.855415815 | -0.154712065 | 5.450998694 | 1.29256E-05 | 0.000420872 | 3.178639775 |
| LRRC10 | 376132 | 1.695672886 | -0.191920562 | 5.450703455 | 1.29352E-05 | 0.000420872 | 3.177912541 |
| LOC400831 | 400831 | -0.583460824 | 0.087157829 | -5.450454786 | 1.29433E-05 | 0.000420872 | 3.177300016 |
| DNMBP | 23268 | 0.568191637 | -0.132166629 | 5.450287559 | 1.29488E-05 | 0.000420872 | 3.176888096 |
| NCOR1 | 9611 | -1.110668549 | 0.259080793 | -5.449318054 | 1.29804E-05 | 0.000421219 | 3.174499944 |
| SPDYA | 245711 | -1.718341908 | -0.035260189 | -5.44930697 | 1.29808E-05 | 0.000421219 | 3.174472641 |
| KIAA0564 | 23078 | 0.831741956 | -0.098956818 | 5.44779763 | 1.30303E-05 | 0.000422246 | 3.170754614 |
| LOC647910 | 647910 | 0.759534559 | 0.009433248 | 5.447688877 | 1.30339E-05 | 0.000422246 | 3.170486712 |
| LOC646501 | 646501 | 0.85578133 | -0.143477524 | 5.4470818 | 1.30538E-05 | 0.000422545 | 3.168991226 |
| CCBL2 | 56267 | 1.312603768 | -0.047419132 | 5.445479638 | 1.31066E-05 | 0.000423213 | 3.165044317 |
| C3orf14 | 57415 | -1.019175021 | -0.407835696 | -5.444665943 | 1.31336E-05 | 0.000423735 | 3.163039728 |
| PARP10 | 84875 | -0.850927098 | 0.164753388 | -5.443675434 | 1.31664E-05 | 0.000424447 | 3.160599491 |
| KRT80 | 144501 | -0.617382668 | 0.128410644 | -5.438464942 | 1.33405E-05 | 0.000429708 | 3.147761811 |
| TMEM47 | 83604 | 0.974971992 | -0.118067747 | 5.436597863 | 1.34035E-05 | 0.000431286 | 3.143161268 |
| LOC649293 | 649293 | 0.704338885 | 0.050663572 | 5.43636345 | 1.34114E-05 | 0.000431286 | 3.142583653 |
| TXNDC11 | 51061 | -1.114906984 | 0.060214881 | -5.435869174 | 1.34281E-05 | 0.000431472 | 3.141365697 |
| CSNK1A1L | 122011 | 0.444129587 | -0.060154486 | 5.433948803 | 1.34933E-05 | 0.000433214 | 3.136633531 |
| LOC644204 | 644204 | 0.832555291 | 0.225510701 | 5.432638503 | 1.35379E-05 | 0.000434294 | 3.133404569 |
| OR1L8 | 138881 | 1.009666075 | -0.128445487 | 5.430738915 | 1.3603E-05 | 0.00043567 | 3.128723238 |
| KCNMB2 | 10242 | 1.269265316 | -0.219424615 | 5.43038034 | 1.36153E-05 | 0.000435711 | 3.127839545 |
| AMICA1 | 120425 | 0.38859614 | -0.022424808 | 5.42926803 | 1.36535E-05 | 0.00043658 | 3.125098251 |
| OXSR1 | 9943 | 0.754110552 | -0.089899119 | 5.428643679 | 1.3675E-05 | 0.000436914 | 3.123559501 |
| MDN1 | 23195 | -0.649868734 | 0.125933359 | -5.420150048 | 1.39712E-05 | 0.000445472 | 3.102624087 |
| WDR75 | 84128 | -0.683397975 | 0.101233308 | -5.419990633 | 1.39768E-05 | 0.000445472 | 3.102231113 |
| BTBD3 | 22903 | 1.442869512 | -0.106681298 | 5.418767061 | 1.402E-05 | 0.000446488 | 3.099214836 |
| HSFY2 | 159119 | -0.576439416 | 0.103172311 | -5.415751689 | 1.41271E-05 | 0.000449457 | 3.091781134 |
| C6orf111 | 25957 | 1.318190046 | -0.044533363 | 5.415500437 | 1.4136E-05 | 0.000449457 | 3.091161705 |
| XBP1 | 7494 | 0.754382058 | -0.130091543 | 5.415172793 | 1.41477E-05 | 0.000449466 | 3.090353936 |
| LOC401206 | 401206 | 0.794959229 | -0.092424553 | 5.413860282 | 1.41946E-05 | 0.000450594 | 3.087118029 |
| VDR | 7421 | 0.466991944 | -0.039860831 | 5.413222474 | 1.42175E-05 | 0.000450957 | 3.085545518 |
| DNM1 | 1759 | 0.456048337 | 0.035039375 | 5.412726063 | 1.42353E-05 | 0.000451159 | 3.084321604 |
| CAPN12 | 147968 | -0.773621535 | 0.131470773 | -5.412358639 | 1.42485E-05 | 0.000451215 | 3.083415701 |
| LSM3 | 27258 | -0.910468368 | 0.256246135 | -5.409963008 | 1.43349E-05 | 0.000453357 | 3.07750895 |
| ISG20L1 | 64782 | 0.635247934 | -0.200654007 | 5.409610066 | 1.43477E-05 | 0.000453357 | 3.076638694 |
| HCCS | 3052 | 1.487521773 | 0.176967982 | 5.409528075 | 1.43507E-05 | 0.000453357 | 3.076436528 |
| LOC652265 | 652265 | 0.873321798 | -0.079052539 | 5.406777147 | 1.44506E-05 | 0.000456149 | 3.069653252 |
| THSD1 | 55901 | -0.575939092 | 0.03100294 | -5.405657183 | 1.44915E-05 | 0.000457075 | 3.066891498 |
| DGKB | 1607 | -0.919517583 | 0.238710716 | -5.404080006 | 1.45493E-05 | 0.000458531 | 3.063002165 |
| LOC650028 | 650028 | 2.330981351 | 0.499105067 | 5.403693384 | 1.45635E-05 | 0.000458612 | 3.06204873 |
| LOC651830 | 651830 | -0.90035569 | 0.143744663 | -5.403311092 | 1.45776E-05 | 0.000458688 | 3.061105963 |
| UPF2 | 26019 | -0.539436492 | 0.04941158 | -5.402748925 | 1.45983E-05 | 0.000458973 | 3.059719594 |
| SPOPL | 339745 | -0.560205455 | -0.034268165 | -5.401248615 | 1.46537E-05 | 0.000460347 | 3.056019562 |
| LOC652857 | 652857 | -0.455872687 | -0.036516032 | -5.400716555 | 1.46734E-05 | 0.000460599 | 3.054707374 |
| MIER3 | 166968 | -0.683425501 | 0.094390586 | -5.399565433 | 1.47161E-05 | 0.000461572 | 3.051868374 |
| SERPINA2 | 390502 | 0.616997602 | -0.115270553 | 5.398933877 | 1.47395E-05 | 0.000461667 | 3.050310743 |
| MOSPD2 | 158747 | 1.114368959 | -0.244177171 | 5.398853565 | 1.47425E-05 | 0.000461667 | 3.050112665 |
| METTL10 | 399818 | 0.504118869 | -0.062260932 | 5.396345456 | 1.48362E-05 | 0.000464051 | 3.04392657 |
| MR1 | 3140 | 0.623761583 | -0.151345133 | 5.396184774 | 1.48422E-05 | 0.000464051 | 3.043530245 |
| FARP1 | 10160 | -1.39632631 | 0.024614753 | -5.393681324 | 1.49363E-05 | 0.000466623 | 3.03735525 |
| RPL17 | 6139 | 0.826528707 | -0.163494141 | 5.391835438 | 1.50061E-05 | 0.000468306 | 3.032801964 |
| LOC648001 | 648001 | -0.626696941 | -0.113035026 | -5.39159511 | 1.50152E-05 | 0.000468306 | 3.032209128 |
| COL9A3 | 1299 | 0.48411798 | 0.0528556 | 5.391282376 | 1.5027E-05 | 0.000468306 | 3.031437676 |
| ATP10D | 57205 | 0.634807762 | -0.095814548 | 5.39100166 | 1.50377E-05 | 0.000468306 | 3.030745201 |
| THUMPD1 | 55623 | 0.734284903 | -0.170528071 | 5.389757623 | 1.5085E-05 | 0.000469408 | 3.027676337 |
| HTR7 | 3363 | -0.775203826 | 0.107465531 | -5.387276267 | 1.51798E-05 | 0.000471986 | 3.021554911 |
| LOC645870 | 645870 | -1.104046735 | 0.303210437 | -5.386317949 | 1.52166E-05 | 0.000472534 | 3.019190676 |
| GPX3 | 2878 | 0.70795649 | 0.132788073 | 5.38494596 | 1.52694E-05 | 0.000473651 | 3.015805794 |
| ZNF827 | 152485 | 1.130292546 | -0.028985461 | 5.383632303 | 1.53201E-05 | 0.000474851 | 3.012564724 |
| OR1S1 | 219959 | -1.235092315 | 0.059477153 | -5.38167523 | 1.5396E-05 | 0.000476829 | 3.007736023 |
| NR4A3 | 8013 | 0.641271962 | -0.147490557 | 5.380400752 | 1.54456E-05 | 0.00047799 | 3.004591377 |
| DIDO1 | 11083 | 0.89226271 | -0.114609079 | 5.37961095 | 1.54765E-05 | 0.000478431 | 3.002642574 |
| NBPF3 | 84224 | -0.451581142 | -0.123678009 | -5.379415225 | 1.54841E-05 | 0.000478431 | 3.002159626 |
| ST6GAL1 | 6480 | 0.715235516 | 0.067181538 | 5.374197877 | 1.56895E-05 | 0.000484397 | 2.98928508 |
| LOC646562 | 646562 | 0.630443012 | 0.042423584 | 5.373563487 | 1.57147E-05 | 0.000484559 | 2.987719527 |
| PTPN9 | 5780 | -1.069985901 | 0.205722006 | -5.373446432 | 1.57193E-05 | 0.000484559 | 2.987430656 |
| LOC130678 | 130678 | 0.566656025 | -0.100276909 | 5.373120837 | 1.57323E-05 | 0.000484578 | 2.98662714 |
| SORCS3 | 22986 | -0.564474067 | 0.098524824 | -5.371312776 | 1.58043E-05 | 0.000486417 | 2.982165021 |
| ITK | 3702 | 0.772112576 | 0.004215818 | 5.369597167 | 1.58729E-05 | 0.000487599 | 2.977930894 |
| C12orf49 | 79794 | -0.946018116 | 0.108607209 | -5.369425806 | 1.58798E-05 | 0.000487599 | 2.977507964 |
| CDH9 | 1007 | -0.80460277 | 0.165245629 | -5.368648087 | 1.5911E-05 | 0.000488178 | 2.975588489 |
| OR5T2 | 219464 | 0.532985664 | -0.076141213 | 5.368263016 | 1.59265E-05 | 0.000488273 | 2.97463809 |
| PVRL1 | 5818 | 0.925295659 | -0.19835408 | 5.367422619 | 1.59604E-05 | 0.00048893 | 2.972563865 |
| LOC727762 | 727762 | 1.363948166 | -0.053156052 | 5.364616201 | 1.60739E-05 | 0.000491279 | 2.965636915 |
| POPDC3 | 64208 | 0.798919597 | -0.090053566 | 5.364297405 | 1.60869E-05 | 0.000491279 | 2.964850018 |
| ACTL6A | 86 | -0.57221706 | 0.098320213 | -5.363181917 | 1.61323E-05 | 0.000492284 | 2.962096569 |
| KLC3 | 147700 | 0.946275312 | -0.191377145 | 5.361436674 | 1.62036E-05 | 0.000493696 | 2.957788506 |
| SPG3A | 51062 | 0.624015512 | -0.062148119 | 5.35937444 | 1.62882E-05 | 0.000495509 | 2.952697743 |
| LOC647451 | 647451 | -0.870245546 | 0.119548763 | -5.358668874 | 1.63173E-05 | 0.000496011 | 2.950955951 |
| LOC441907 | 441907 | -0.899157095 | -0.316618449 | -5.357336676 | 1.63723E-05 | 0.0004973 | 2.947667152 |
| LOC644172 | 644172 | -0.98225718 | 0.160577506 | -5.356151441 | 1.64214E-05 | 0.000498321 | 2.944741079 |
| LOC90586 | 90586 | 0.597097477 | -0.106729505 | 5.355915926 | 1.64312E-05 | 0.000498321 | 2.944159636 |
| RBM10 | 8241 | -0.865757633 | 0.28247427 | -5.354570983 | 1.64872E-05 | 0.000499633 | 2.940839168 |
| PCDHAC2 | 56134 | -0.777951536 | -0.308834214 | -5.352548047 | 1.65717E-05 | 0.000501808 | 2.935844648 |
| C17orf66 | 256957 | 1.376145544 | -0.127642876 | 5.350993027 | 1.66369E-05 | 0.000503398 | 2.932005232 |
| DNAJB4 | 11080 | -0.967034536 | 0.171268041 | -5.350525434 | 1.66566E-05 | 0.000503471 | 2.930850697 |
| PFDN4 | 5203 | 1.91757796 | 0.115851147 | 5.349562195 | 1.66972E-05 | 0.000504061 | 2.928472323 |
| SEMA4C | 54910 | 0.769226097 | -0.108917658 | 5.348421239 | 1.67454E-05 | 0.00050513 | 2.925655077 |
| XPO5 | 57510 | 0.545424213 | -0.081167767 | 5.344913377 | 1.68946E-05 | 0.000508934 | 2.916993024 |
| ATXN2L | 11273 | -0.934694866 | -0.26499801 | -5.344848214 | 1.68973E-05 | 0.000508934 | 2.916832107 |
| PRAMEF9 | 343070 | 0.911910345 | -0.152533622 | 5.343898717 | 1.69379E-05 | 0.000509767 | 2.914487371 |
| PRSS35 | 167681 | 0.940135262 | -0.080065054 | 5.342878849 | 1.69817E-05 | 0.000510672 | 2.911968803 |
| SNORA66 | 26782 | -1.114452995 | -0.204102261 | -5.342593929 | 1.69939E-05 | 0.000510672 | 2.911265182 |
| LOC643006 | 643006 | -0.85410261 | 0.110704133 | -5.341741776 | 1.70306E-05 | 0.000511383 | 2.909160729 |
| LOC440748 | 440748 | 0.414720161 | -0.044706261 | 5.340976412 | 1.70635E-05 | 0.000511903 | 2.907270574 |
| LHX4 | 89884 | -0.962690572 | 0.201884744 | -5.34073786 | 1.70738E-05 | 0.000511903 | 2.906681437 |
| POU5F2 | 134187 | -0.661699288 | 0.179624003 | -5.340126423 | 1.71002E-05 | 0.000512306 | 2.905171391 |
| INSM2 | 84684 | -0.713289735 | 0.213912646 | -5.339056953 | 1.71465E-05 | 0.00051265 | 2.902530111 |
| CENTD2 | 116985 | -0.723971403 | -0.049860124 | -5.338521786 | 1.71698E-05 | 0.00051265 | 2.901208378 |
| BRP44 | 25874 | -1.070109442 | 0.094686411 | -5.33838644 | 1.71756E-05 | 0.00051265 | 2.900874104 |
| HAT1 | 8520 | -0.601546602 | 0.146545241 | -5.33836084 | 1.71767E-05 | 0.00051265 | 2.90081088 |
| PHKB | 5257 | -0.688455428 | 0.148809224 | -5.335851324 | 1.72861E-05 | 0.000515253 | 2.894612765 |
| KAT5 | 10524 | 0.613632419 | -0.074850218 | 5.333798533 | 1.7376E-05 | 0.000517282 | 2.889542436 |
| TIGD7 | 91151 | 0.519326445 | -0.014762729 | 5.333607637 | 1.73844E-05 | 0.000517282 | 2.889070918 |
| USP51 | 158880 | -1.258888619 | 0.124834723 | -5.333007702 | 1.74108E-05 | 0.000517676 | 2.887589049 |
| ACTG1 | 71 | -0.973374437 | 0.06102776 | -5.331950246 | 1.74574E-05 | 0.000518671 | 2.884977033 |
| KRTAP3-1 | 83896 | 0.558619503 | -0.04559312 | 5.330912881 | 1.75033E-05 | 0.000519254 | 2.882414587 |
| LOC649133 | 649133 | -0.935844361 | 0.149078312 | -5.330910549 | 1.75034E-05 | 0.000519254 | 2.882408827 |
| MPPE1 | 65258 | -0.842646051 | 0.210551421 | -5.330091282 | 1.75397E-05 | 0.000519733 | 2.880385075 |
| LOC654126 | 654126 | 0.531349298 | -0.132461082 | 5.329777032 | 1.75536E-05 | 0.000519733 | 2.879608803 |
| MAGEA11 | 4110 | 0.843783275 | -0.096179086 | 5.329654333 | 1.75591E-05 | 0.000519733 | 2.879305708 |
| RGPD1 | 400966 | 0.543760702 | -0.082337325 | 5.327929084 | 1.76359E-05 | 0.000521614 | 2.875043843 |
| LOC285359 | 285359 | -0.987425949 | 0.258390844 | -5.325495305 | 1.77447E-05 | 0.000524441 | 2.869031432 |
| AKR1C2 | 1646 | -0.589829772 | 0.081789848 | -5.323843687 | 1.7819E-05 | 0.000526242 | 2.864951094 |
| UBE2E3 | 10477 | 0.571396531 | -0.081470507 | 5.322639842 | 1.78734E-05 | 0.000527451 | 2.861976893 |
| GAGE12G | 645073 | 0.710943894 | -0.191556404 | 5.321608633 | 1.792E-05 | 0.000528433 | 2.859429144 |
| ATF4 | 468 | -0.774799147 | 0.155457563 | -5.319733034 | 1.80053E-05 | 0.000529758 | 2.854795063 |
| TNNI3 | 7137 | 1.5092989 | -0.296838135 | 5.318111859 | 1.80792E-05 | 0.000531538 | 2.850789446 |
| SUNC1 | 256979 | 0.586393409 | -0.067091511 | 5.317224246 | 1.81199E-05 | 0.000532336 | 2.848596263 |
| IRAK1BP1 | 134728 | -0.60297379 | 0.09098894 | -5.31647811 | 1.81541E-05 | 0.000532944 | 2.84675262 |
| LOC645143 | 645143 | -0.924913711 | 0.280058731 | -5.315991155 | 1.81765E-05 | 0.000533204 | 2.845549377 |
| LOC729744 | 729744 | 0.979443711 | -0.038981451 | 5.313704196 | 1.8282E-05 | 0.000535899 | 2.839898245 |
| TMEM191B | 728229 | -0.694020933 | 0.081553389 | -5.313409904 | 1.82956E-05 | 0.0005359 | 2.839171022 |
| XPNPEP2 | 7512 | 0.52708221 | -0.09191885 | 5.312581459 | 1.8334E-05 | 0.000536626 | 2.837123835 |
| LEREPO4 | 55854 | -1.307122859 | 0.060358833 | -5.31158907 | 1.838E-05 | 0.000537575 | 2.834671474 |
| TTRAP | 51567 | 1.025003935 | -0.152477433 | 5.310592478 | 1.84264E-05 | 0.000538519 | 2.832208676 |
| ACOT7 | 11332 | 0.730596743 | 0.081887481 | 5.310309847 | 1.84396E-05 | 0.000538519 | 2.831510223 |
| SNRNP35 | 11066 | -0.791657554 | 0.158388798 | -5.305968628 | 1.86433E-05 | 0.000544064 | 2.820781456 |
| SNTN | 132203 | 0.690542619 | -0.12751407 | 5.304179887 | 1.87279E-05 | 0.000545888 | 2.816360528 |
| LOC732424 | 732424 | 0.961555867 | -0.026509455 | 5.303425026 | 1.87637E-05 | 0.000546364 | 2.814494818 |
| TMEM104 | 54868 | 0.475011304 | -0.00276189 | 5.299863738 | 1.89335E-05 | 0.000550696 | 2.805692362 |
| PDZRN3 | 23024 | 0.903193711 | -0.111345609 | 5.299559725 | 1.89481E-05 | 0.000550696 | 2.804940903 |
| LOC644152 | 644152 | 0.465194224 | -0.095176293 | 5.299429531 | 1.89544E-05 | 0.000550696 | 2.804619089 |
| LOC81691 | 81691 | 0.738059511 | 0.051228254 | 5.298641567 | 1.89922E-05 | 0.000551389 | 2.802671368 |
| LBH | 81606 | -0.468232812 | -0.005007625 | -5.297263659 | 1.90585E-05 | 0.000552908 | 2.799265327 |
| SDHC | 6391 | 0.495326805 | -0.03200781 | 5.2956421 | 1.91369E-05 | 0.000554256 | 2.795256883 |
| STARD13 | 90627 | 1.109618541 | -0.135288656 | 5.295430527 | 1.91472E-05 | 0.000554256 | 2.794733872 |
| LOC647625 | 647625 | -0.830580564 | 0.033392403 | -5.294948828 | 1.91705E-05 | 0.000554525 | 2.793543096 |
| KLK4 | 9622 | 0.640324932 | -0.104282964 | 5.294406588 | 1.91969E-05 | 0.000554879 | 2.792202646 |
| ZNF761 | 388561 | -0.712651107 | 0.148566296 | -5.292646808 | 1.92826E-05 | 0.000556948 | 2.787852266 |
| LOC647042 | 647042 | 2.190033435 | 0.469530669 | 5.292209169 | 1.93039E-05 | 0.000557157 | 2.786770347 |
| LOC651345 | 651345 | 0.566175163 | -0.119954162 | 5.290798581 | 1.9373E-05 | 0.000558741 | 2.783283066 |
| RASSF7 | 8045 | 0.761586179 | -0.145928111 | 5.289759042 | 1.9424E-05 | 0.000559395 | 2.780713033 |
| INPP5B | 3633 | -0.921326408 | 0.117718462 | -5.28693067 | 1.95636E-05 | 0.000562179 | 2.773720234 |
| PIK3R3 | 8503 | -0.675153248 | 0.123749365 | -5.286910165 | 1.95646E-05 | 0.000562179 | 2.773669535 |
| PHF17 | 79960 | -0.783881372 | 0.136041368 | -5.286676584 | 1.95762E-05 | 0.000562179 | 2.773092019 |
| TAF13 | 6884 | 0.522947007 | 0.061635372 | 5.286645108 | 1.95777E-05 | 0.000562179 | 2.773014196 |
| NSL1 | 25936 | -0.705871228 | 0.108349116 | -5.286119467 | 1.96038E-05 | 0.000562196 | 2.77171456 |
| FBXO11 | 80204 | -1.07212401 | 0.158958587 | -5.286058449 | 1.96068E-05 | 0.000562196 | 2.771563694 |
| MTUS1 | 57509 | -0.640251913 | 0.162743078 | -5.285133197 | 1.96528E-05 | 0.000563105 | 2.769275998 |
| CYC1 | 1537 | 1.067879611 | -0.018875052 | 5.284382486 | 1.96902E-05 | 0.000563766 | 2.767419823 |
| TEX9 | 374618 | 0.527909016 | -0.079499494 | 5.283952517 | 1.97116E-05 | 0.000563971 | 2.766356688 |
| UBC | 7316 | 0.518433546 | -0.080627304 | 5.283046718 | 1.97569E-05 | 0.000564855 | 2.764116995 |
| CACNG5 | 27091 | -1.443802982 | 0.060498507 | -5.282414584 | 1.97885E-05 | 0.00056535 | 2.762553944 |
| SNORD54 | 26795 | 0.485924678 | -0.194552586 | 5.28102915 | 1.98581E-05 | 0.000566926 | 2.759128175 |
| GPIHBP1 | 338328 | -0.851864039 | 0.141554014 | -5.279036922 | 1.99585E-05 | 0.000568969 | 2.754201818 |
| HIPK2 | 28996 | -0.670550212 | -0.177844589 | -5.278377818 | 1.99918E-05 | 0.000569507 | 2.75257195 |
| MTMR6 | 9107 | -0.876152351 | 0.093444467 | -5.27749494 | 2.00366E-05 | 0.00057037 | 2.750388691 |
| CXorf58 | 254158 | 1.486910646 | -0.182321064 | 5.273568104 | 2.02368E-05 | 0.000575191 | 2.740677591 |
| SNAPC5 | 10302 | 0.920312709 | -0.161704248 | 5.273528157 | 2.02389E-05 | 0.000575191 | 2.740578797 |
| ATRX | 546 | 0.85167611 | -0.055203499 | 5.27331629 | 2.02497E-05 | 0.000575191 | 2.740054827 |
| VIM | 7431 | -0.563615962 | 0.001888927 | -5.271922841 | 2.03213E-05 | 0.000576809 | 2.736608618 |
| MGMT | 4255 | -0.941082647 | -0.433163354 | -5.270494089 | 2.0395E-05 | 0.000578296 | 2.733075001 |
| LOC646863 | 646863 | 0.986655103 | -0.109994933 | 5.268741868 | 2.04857E-05 | 0.000580221 | 2.728741239 |
| LOC730031 | 730031 | 0.622333238 | -0.101663939 | 5.267720524 | 2.05387E-05 | 0.000581307 | 2.726215084 |
| LOC652740 | 652740 | 0.65154268 | -0.073384593 | 5.266602911 | 2.0597E-05 | 0.000582536 | 2.723450765 |
| L3MBTL | 26013 | 0.937008644 | -0.136744033 | 5.263992483 | 2.07336E-05 | 0.00058569 | 2.716993862 |
| PNMA2 | 10687 | 1.167739003 | -0.01545191 | 5.263905155 | 2.07382E-05 | 0.00058569 | 2.716777849 |
| LOC222967 | 222967 | 0.751899518 | -0.043624517 | 5.260999275 | 2.08914E-05 | 0.000589294 | 2.709589746 |
| LOC647854 | 647854 | -0.627615508 | 0.042099465 | -5.260855188 | 2.0899E-05 | 0.000589294 | 2.709233316 |
| PDGFA | 5154 | -0.647186628 | 0.099521234 | -5.260524381 | 2.09165E-05 | 0.000589294 | 2.708414991 |
| SLC34A1 | 6569 | 1.660766155 | 0.100686154 | 5.260353665 | 2.09256E-05 | 0.000589294 | 2.707992686 |
| BEX1 | 55859 | 0.477460689 | 0.060546186 | 5.259974355 | 2.09457E-05 | 0.00058944 | 2.707054367 |
| C15orf41 | 84529 | -0.574542738 | 0.146707323 | -5.258609231 | 2.10183E-05 | 0.00059106 | 2.703677337 |
| KHDC1 | 80759 | -0.804210178 | 0.104646018 | -5.257973246 | 2.10521E-05 | 0.000591591 | 2.702104014 |
| MPP7 | 143098 | -0.532448595 | 0.074605184 | -5.254495226 | 2.12385E-05 | 0.000596401 | 2.693499619 |
| LOC647215 | 647215 | 0.736044801 | -0.047785979 | 5.253346209 | 2.13004E-05 | 0.000597714 | 2.690656898 |
| FKBP9L | 360132 | -0.737919294 | 0.147800583 | -5.252213255 | 2.13616E-05 | 0.000599006 | 2.68785386 |
| GLS2 | 27165 | 0.595840121 | -0.098794181 | 5.25053628 | 2.14526E-05 | 0.000601129 | 2.683704752 |
| PCDHB2 | 56133 | 0.733514686 | -0.139915131 | 5.249279647 | 2.1521E-05 | 0.000602618 | 2.68059555 |
| GPR27 | 2850 | 0.882166267 | -0.011274619 | 5.247455327 | 2.16207E-05 | 0.000604711 | 2.676081629 |
| LOC650628 | 650628 | 2.147470806 | 0.163378261 | 5.247351217 | 2.16264E-05 | 0.000604711 | 2.675824022 |
| PTTG1 | 9232 | 0.53457361 | -0.158855557 | 5.246417961 | 2.16776E-05 | 0.000605713 | 2.6735148 |
| ANXA8 | 244 | 1.069030668 | -0.07777459 | 5.24184698 | 2.19302E-05 | 0.000611936 | 2.662203909 |
| BDNF | 627 | -0.644318424 | 0.131809107 | -5.241825199 | 2.19314E-05 | 0.000611936 | 2.662150008 |
| LOC643171 | 643171 | -0.856379593 | 0.171161466 | -5.241341855 | 2.19583E-05 | 0.000612253 | 2.660953919 |
| ZCCHC9 | 84240 | 0.745940757 | -0.059422861 | 5.240701512 | 2.19939E-05 | 0.000612814 | 2.659369299 |
| LOC653596 | 653596 | 1.309336411 | -0.160242594 | 5.239891788 | 2.20391E-05 | 0.000613639 | 2.657365492 |
| LDHD | 197257 | 1.349235455 | 0.06548512 | 5.237470534 | 2.21748E-05 | 0.000616541 | 2.651373496 |
| SERF1A | 8293 | 0.614096303 | -0.136857044 | 5.237360686 | 2.21809E-05 | 0.000616541 | 2.651101643 |
| LOC644528 | 644528 | -0.69742705 | -0.250625238 | -5.237195297 | 2.21902E-05 | 0.000616541 | 2.650692334 |
| EBF2 | 64641 | -1.101889529 | 0.110146338 | -5.23657223 | 2.22253E-05 | 0.000617081 | 2.649150347 |
| FXYD2 | 486 | -0.991845542 | -0.024600482 | -5.235583516 | 2.22811E-05 | 0.000618194 | 2.64670341 |
| CSF3R | 1441 | 0.456514951 | 0.009068948 | 5.234737205 | 2.23289E-05 | 0.000619086 | 2.644608867 |
| GLUD1 | 2746 | 0.572033534 | 0.051908342 | 5.234233527 | 2.23574E-05 | 0.000619441 | 2.643362295 |
| SLC22A10 | 387775 | -0.605170494 | 0.143778391 | -5.23013781 | 2.25908E-05 | 0.000625466 | 2.633225211 |
| CCR7 | 1236 | 0.681360308 | 0.050007914 | 5.226090457 | 2.28237E-05 | 0.000631473 | 2.62320709 |
| RP2 | 6102 | -0.786380405 | 0.094981739 | -5.221812039 | 2.30727E-05 | 0.000637912 | 2.612616235 |
| NLGN2 | 57555 | 0.458200986 | -0.074290508 | 5.220928428 | 2.31244E-05 | 0.000638391 | 2.610428831 |
| SNX14 | 57231 | -0.820056899 | 0.107940431 | -5.220881835 | 2.31271E-05 | 0.000638391 | 2.610313488 |
| THY1 | 7070 | 0.679735818 | -0.067682079 | 5.220686927 | 2.31386E-05 | 0.000638391 | 2.609830984 |
| ALDH3B1 | 221 | -1.217500507 | 0.121492335 | -5.219556206 | 2.3205E-05 | 0.000639777 | 2.60703179 |
| PGLYRP3 | 114771 | 0.417940117 | -0.007912675 | 5.217551047 | 2.33233E-05 | 0.000642588 | 2.602067713 |
| RASGRP4 | 115727 | 0.685369552 | -7.5515E-06 | 5.217104937 | 2.33497E-05 | 0.000642766 | 2.600963278 |
| IDE | 3416 | -0.755845853 | 0.172144895 | -5.216890994 | 2.33624E-05 | 0.000642766 | 2.600433614 |
| PEX13 | 5194 | 0.648691371 | -0.197495579 | 5.214818523 | 2.34855E-05 | 0.000645308 | 2.595302654 |
| NPR2 | 4882 | 0.88805131 | -0.154980909 | 5.21478434 | 2.34875E-05 | 0.000645308 | 2.595218023 |
| LOC646207 | 646207 | -1.000696059 | 0.238948239 | -5.214137119 | 2.35261E-05 | 0.000645885 | 2.593615612 |
| PAN2 | 9924 | 0.724631043 | -0.198082389 | 5.213882779 | 2.35412E-05 | 0.000645885 | 2.592985903 |
| LOC653600 | 653600 | 0.518846288 | -0.124322093 | 5.212415298 | 2.3629E-05 | 0.000647843 | 2.589352585 |
| OR2Z1 | 284383 | 0.891910289 | 0.095770159 | 5.210364858 | 2.37522E-05 | 0.000650767 | 2.58427577 |
| NDUFB2 | 4708 | -0.467641155 | 0.023784032 | -5.208449058 | 2.38679E-05 | 0.000653201 | 2.579532153 |
| HLTF | 6596 | 0.825693818 | -0.166929203 | 5.208345763 | 2.38741E-05 | 0.000653201 | 2.579276385 |
| LOC653889 | 653889 | 0.405625164 | 0.003360558 | 5.204530189 | 2.41063E-05 | 0.000659095 | 2.569828344 |
| LOC606724 | 606724 | 1.06715332 | -0.179600006 | 5.204127223 | 2.4131E-05 | 0.000659312 | 2.568830495 |
| SNHG10 | 283596 | 0.630363673 | -0.184828749 | 5.203088076 | 2.41946E-05 | 0.00066041 | 2.566257256 |
| LOC653978 | 653978 | 1.212852287 | -0.249060153 | 5.202925148 | 2.42046E-05 | 0.00066041 | 2.565853792 |
| FADD | 8772 | -0.597864597 | 0.066581917 | -5.201459753 | 2.42948E-05 | 0.000662411 | 2.562224949 |
| FBXO9 | 26268 | 0.672941214 | -0.172697418 | 5.198569473 | 2.44736E-05 | 0.000666631 | 2.555067313 |
| UNC13C | 440279 | 0.894778638 | -0.092694177 | 5.198168256 | 2.44985E-05 | 0.000666631 | 2.55407369 |
| ACOX1 | 51 | 0.851671322 | -0.159533589 | 5.19287614 | 2.48296E-05 | 0.00067466 | 2.540967024 |
| KRT1 | 3848 | -0.641002939 | 0.140498083 | -5.192422544 | 2.48582E-05 | 0.000674972 | 2.539843573 |
| TRIM58 | 25893 | 1.147182682 | 0.13193747 | 5.191723345 | 2.49023E-05 | 0.000675705 | 2.538111809 |
| JAG1 | 182 | -1.035678652 | 0.056060816 | -5.190909448 | 2.49538E-05 | 0.000675881 | 2.536095935 |
| ACTR3B | 57180 | 0.5139411 | -0.103763891 | 5.190856561 | 2.49572E-05 | 0.000675881 | 2.535964943 |
| SUMO1P3 | 474338 | -0.672791044 | -0.030685387 | -5.190807552 | 2.49603E-05 | 0.000675881 | 2.535843558 |
| VPS13B | 157680 | 0.949522149 | -0.23019767 | 5.188528727 | 2.5105E-05 | 0.000679334 | 2.530199172 |
| WNT8A | 7478 | -0.836455369 | -0.199811877 | -5.18682571 | 2.52137E-05 | 0.000681776 | 2.525980856 |
| PRB1 | 5542 | -0.563150374 | -0.136266378 | -5.186573878 | 2.52298E-05 | 0.000681776 | 2.525357067 |
| LIMA1 | 51474 | 0.410717039 | 0.025670024 | 5.185707793 | 2.52853E-05 | 0.00068234 | 2.523211749 |
| TTTY5 | 83863 | -1.20459165 | 0.243918039 | -5.185266681 | 2.53136E-05 | 0.000682637 | 2.522119088 |
| GTF2E2 | 2961 | 0.532857993 | -0.118171881 | 5.18380544 | 2.54077E-05 | 0.000684705 | 2.518499456 |
| SLC17A4 | 10050 | 0.485359081 | -0.10774424 | 5.181873323 | 2.55326E-05 | 0.0006876 | 2.513713286 |
| LOC644638 | 644638 | 0.646044101 | -0.131371379 | 5.180334735 | 2.56324E-05 | 0.000689817 | 2.50990184 |
| LOC653127 | 653127 | -1.021916412 | 0.215213804 | -5.179816452 | 2.56662E-05 | 0.000689817 | 2.508617909 |
| FBXL15 | 79176 | 1.027768702 | -0.202568207 | 5.179798464 | 2.56674E-05 | 0.000689817 | 2.508573349 |
| MELK | 9833 | 1.205133976 | -0.045362842 | 5.177504334 | 2.58172E-05 | 0.000693372 | 2.502890022 |
| OR8B3 | 390271 | 1.052797431 | 0.330511259 | 5.177195748 | 2.58375E-05 | 0.000693443 | 2.502125534 |
| LOC642859 | 642859 | 0.646506277 | -0.076093877 | 5.174516377 | 2.60138E-05 | 0.000697699 | 2.495487534 |
| ZNF683 | 257101 | -0.653903452 | 0.103648867 | -5.170546223 | 2.62772E-05 | 0.000704261 | 2.485651161 |
| ZCCHC6 | 79670 | 0.711577193 | -0.13629716 | 5.170276395 | 2.62952E-05 | 0.000704261 | 2.484982617 |
| E2F1 | 1869 | 0.630505864 | -0.170954019 | 5.169711518 | 2.63329E-05 | 0.000704261 | 2.483583033 |
| FSHR | 2492 | -0.801712561 | 0.218141049 | -5.169596737 | 2.63406E-05 | 0.000704261 | 2.483298641 |
| DOCK4 | 9732 | -1.547542705 | -0.047090125 | -5.169087161 | 2.63747E-05 | 0.000704505 | 2.482036061 |
| RAD1 | 5810 | -1.157487551 | 0.065592503 | -5.166348792 | 2.65587E-05 | 0.000708459 | 2.475251012 |
| ZNFX1 | 57169 | -0.778801319 | -0.2947846 | -5.165977739 | 2.65837E-05 | 0.000708647 | 2.474331603 |
| CLDN15 | 24146 | 0.891143869 | -0.103286577 | 5.16550116 | 2.66159E-05 | 0.000709026 | 2.473150713 |
| PCSK9 | 255738 | 1.153715399 | -0.128390376 | 5.164960334 | 2.66525E-05 | 0.000709521 | 2.471810618 |
| ROM1 | 6094 | -0.59883702 | 0.107050906 | -5.163815925 | 2.673E-05 | 0.000711105 | 2.468974885 |
| GOSR1 | 9527 | -0.643757293 | 0.140150256 | -5.162675062 | 2.68075E-05 | 0.000712686 | 2.466147889 |
| LOC155060 | 155060 | 1.013102533 | -0.057998089 | 5.16214433 | 2.68437E-05 | 0.000713166 | 2.464832746 |
| VAMP1 | 6843 | -1.233708881 | 0.082195162 | -5.159472489 | 2.70264E-05 | 0.000717464 | 2.458211815 |
| WWOX | 51741 | -0.731493754 | 0.173797111 | -5.159247368 | 2.70418E-05 | 0.000717464 | 2.457653944 |
| CHL1 | 10752 | -0.846661012 | 0.154128741 | -5.153551073 | 2.74358E-05 | 0.000727426 | 2.443537325 |
| FOXR2 | 139628 | -0.541006821 | 0.099210623 | -5.152100872 | 2.7537E-05 | 0.000729619 | 2.439943223 |
| TRPM3 | 80036 | -0.811953941 | 0.145678009 | -5.151646097 | 2.75688E-05 | 0.000729972 | 2.438816116 |
| PADI3 | 51702 | 0.843527794 | -0.16901366 | 5.149049093 | 2.77512E-05 | 0.000733563 | 2.432379594 |
| LOC647046 | 647046 | -0.489000189 | -0.026756733 | -5.148985936 | 2.77557E-05 | 0.000733563 | 2.432223059 |
| RPL36AL | 6166 | 1.015802002 | -0.233405517 | 5.148714811 | 2.77748E-05 | 0.000733563 | 2.431551077 |
| EFCAB7 | 84455 | -0.896156747 | 0.103891731 | -5.148656939 | 2.77789E-05 | 0.000733563 | 2.43140764 |
| POLR1E | 64425 | -0.657151032 | -0.166349362 | -5.147192848 | 2.78823E-05 | 0.000735803 | 2.427778842 |
| LOC645037 | 645037 | -0.913970078 | 0.167349954 | -5.146576074 | 2.79261E-05 | 0.000736463 | 2.426250124 |
| FNDC4 | 64838 | -0.956792123 | -0.443192178 | -5.143793778 | 2.81241E-05 | 0.00074119 | 2.419353827 |
| NELL2 | 4753 | 0.879436138 | -0.143933727 | 5.143278688 | 2.81609E-05 | 0.000741408 | 2.418077075 |
| SEPHS1 | 22929 | 0.872728093 | -0.200624335 | 5.142661377 | 2.82051E-05 | 0.000741837 | 2.416546934 |
| REXO4 | 57109 | -0.853033596 | 0.143238318 | -5.140934977 | 2.8329E-05 | 0.000743685 | 2.4122676 |
| ANKRD20A1 | 84210 | 0.619050364 | -0.011344287 | 5.140903131 | 2.83313E-05 | 0.000743685 | 2.412188661 |
| RBCK1 | 10616 | -0.687657755 | 0.127326871 | -5.140894362 | 2.83319E-05 | 0.000743685 | 2.412166925 |
| SLC8A2 | 6543 | 0.525349923 | -0.085849548 | 5.139578108 | 2.84268E-05 | 0.000745529 | 2.408904169 |
| LOC731431 | 731431 | 0.671472154 | -0.018060619 | 5.139395472 | 2.844E-05 | 0.000745529 | 2.408451442 |
| UBE2Z | 65264 | 1.018186426 | -0.049129927 | 5.139052872 | 2.84648E-05 | 0.000745682 | 2.407602187 |
| LOC643904 | 643904 | -1.287720009 | 0.062913358 | -5.138636295 | 2.84949E-05 | 0.000745976 | 2.406569546 |
| LOC130773 | 130773 | 0.764637125 | -0.103321337 | 5.138185603 | 2.85275E-05 | 0.000746335 | 2.405452332 |
| FLJ32065 | 201283 | -1.546679585 | 0.155732442 | -5.136779013 | 2.86296E-05 | 0.000748509 | 2.401965506 |
| SNIP1 | 79753 | 1.466402443 | 0.078969595 | 5.135693849 | 2.87087E-05 | 0.000750078 | 2.399275423 |
| OR51E2 | 81285 | -0.875850065 | 0.122846547 | -5.13445574 | 2.87991E-05 | 0.000751942 | 2.39620614 |
| C19orf18 | 147685 | 0.430354671 | -0.076370466 | 5.132533255 | 2.89401E-05 | 0.000755123 | 2.391440171 |
| CCDC61 | 729440 | 0.638398243 | -0.149167083 | 5.130131831 | 2.91172E-05 | 0.000759241 | 2.385486697 |
| SLC5A10 | 125206 | 0.487145679 | -0.021200879 | 5.129282249 | 2.91801E-05 | 0.000760379 | 2.38338041 |
| MGC13168 | 84821 | 0.617691257 | 0.081984278 | 5.127982921 | 2.92765E-05 | 0.000762389 | 2.380159063 |
| LOC653765 | 653765 | -0.833682258 | 0.098147746 | -5.126814007 | 2.93636E-05 | 0.000764152 | 2.377260993 |
| SGTA | 6449 | -1.030353822 | 0.023634037 | -5.12561696 | 2.94531E-05 | 0.000765974 | 2.374293119 |
| LOC400388 | 400388 | -0.696498419 | 0.111584492 | -5.121343539 | 2.97746E-05 | 0.000773825 | 2.363697495 |
| FAM80A | 284716 | -0.664549954 | 0.016076907 | -5.119948449 | 2.98803E-05 | 0.00077555 | 2.360238335 |
| RNF4 | 6047 | 0.680619476 | -0.105965182 | 5.119329554 | 2.99273E-05 | 0.00077626 | 2.35870375 |
| LOC653214 | 653214 | -0.667429793 | 0.131369357 | -5.118879715 | 2.99616E-05 | 0.000776637 | 2.357588339 |
| SOHLH1 | 402381 | 0.836522193 | -0.117558695 | 5.117809067 | 3.00432E-05 | 0.000778241 | 2.354933555 |
| LOC645393 | 645393 | 0.490938304 | 0.016100134 | 5.116926133 | 3.01107E-05 | 0.00077911 | 2.352744197 |
| TMEM225 | 338661 | 1.227745057 | -0.139119048 | 5.116852897 | 3.01163E-05 | 0.00077911 | 2.352562598 |
| LOC338579 | 338579 | -0.563696104 | 0.101546565 | -5.115671527 | 3.02068E-05 | 0.00078094 | 2.34963318 |
| UGP2 | 7360 | -0.768571472 | -0.095823961 | -5.11535332 | 3.02312E-05 | 0.00078106 | 2.348844123 |
| DAAM1 | 23002 | -0.629319249 | 0.034075284 | -5.114606241 | 3.02887E-05 | 0.000782031 | 2.346991574 |
| DDX42 | 11325 | 0.868399495 | -0.050627985 | 5.110824609 | 3.05812E-05 | 0.000789066 | 2.337613885 |
| LSM2 | 57819 | -2.225019919 | 0.143543722 | -5.110036671 | 3.06425E-05 | 0.000789863 | 2.335659893 |
| FIP1L1 | 81608 | -0.946662228 | 0.231915884 | -5.109912635 | 3.06521E-05 | 0.000789863 | 2.335352298 |
| EDARADD | 128178 | -1.702025069 | 0.003514473 | -5.10936456 | 3.06949E-05 | 0.000790448 | 2.333993124 |
| MTMR3 | 8897 | -0.751343088 | 0.092272388 | -5.108570268 | 3.07569E-05 | 0.000791528 | 2.332023339 |
| SLC25A15 | 10166 | -0.847402583 | 0.119111019 | -5.106629219 | 3.0909E-05 | 0.000794923 | 2.327209586 |
| INO80 | 54617 | 0.455798949 | 0.004525434 | 5.105347368 | 3.10098E-05 | 0.000796478 | 2.324030558 |
| CMTM3 | 123920 | 0.408088096 | 0.042023537 | 5.104918732 | 3.10436E-05 | 0.000796827 | 2.322967517 |
| SCNN1D | 6339 | 0.753658643 | -0.080863312 | 5.104582182 | 3.10702E-05 | 0.00079699 | 2.322132851 |
| NME6 | 10201 | 0.785695507 | -0.167704796 | 5.102200228 | 3.12589E-05 | 0.000801309 | 2.316225327 |
| USP50 | 373509 | 0.563812635 | -0.020123698 | 5.101323236 | 3.13286E-05 | 0.000802575 | 2.31405024 |
| TBC1D3C | 414060 | 0.492724217 | 0.086081827 | 5.10024344 | 3.14148E-05 | 0.000804151 | 2.311372126 |
| MICAL3 | 57553 | -0.594528909 | 0.100959759 | -5.10004066 | 3.14309E-05 | 0.000804151 | 2.310869185 |
| EEF1A1 | 1915 | 0.894456953 | -0.15905104 | 5.096980106 | 3.16764E-05 | 0.000808857 | 2.303278162 |
| BMP1 | 649 | 0.852980904 | -0.198019371 | 5.095950847 | 3.17594E-05 | 0.000810414 | 2.300725245 |
| TM4SF5 | 9032 | 1.847495531 | 0.224870272 | 5.095714303 | 3.17785E-05 | 0.000810414 | 2.300138529 |
| BRD4 | 23476 | -1.028096835 | -0.018211083 | -5.093450919 | 3.19619E-05 | 0.000813685 | 2.294524416 |
| ZNF322B | 387328 | 0.835295061 | -0.146884702 | 5.093367141 | 3.19687E-05 | 0.000813685 | 2.294316608 |
| OR4C13 | 283092 | -0.932392851 | 0.073936046 | -5.091383653 | 3.21303E-05 | 0.000816668 | 2.289396609 |
| WDR54 | 84058 | 0.727500235 | -0.186701314 | 5.091166712 | 3.2148E-05 | 0.000816668 | 2.288858483 |
| TMEM184A | 202915 | 0.739796852 | 0.027722317 | 5.086850495 | 3.25028E-05 | 0.000825147 | 2.278151732 |
| ATP5G1 | 516 | 0.9082648 | -0.088750579 | 5.083410443 | 3.27883E-05 | 0.00083186 | 2.269617956 |
| CAPSL | 133690 | 0.91681594 | -0.092926712 | 5.083049013 | 3.28184E-05 | 0.00083209 | 2.268721331 |
| LOC646585 | 646585 | -0.628338256 | 0.114289406 | -5.08087593 | 3.30003E-05 | 0.000836162 | 2.263330321 |
| CEACAM3 | 1084 | 0.50003019 | -0.021049498 | 5.078569918 | 3.31944E-05 | 0.000840387 | 2.257609381 |
| LOC642361 | 642361 | 0.436718858 | -0.054756012 | 5.07815253 | 3.32296E-05 | 0.000840387 | 2.256573872 |
| LOC201175 | 201175 | 0.51254171 | 0.056167888 | 5.078136687 | 3.32309E-05 | 0.000840387 | 2.256534567 |
| ENAM | 10117 | 0.517768604 | -0.036116194 | 5.075414498 | 3.34618E-05 | 0.000845682 | 2.249780891 |
| ST6GALNAC4 | 27090 | 0.773717268 | -0.138599884 | 5.075089642 | 3.34894E-05 | 0.000845839 | 2.248974915 |
| PRO0628 | 29053 | -0.85647688 | 0.142708725 | -5.074677858 | 3.35245E-05 | 0.000846068 | 2.247953266 |
| MT1P2 | 645745 | 0.726809474 | -0.102252127 | 5.074285574 | 3.3558E-05 | 0.000846068 | 2.246979992 |
| NSF | 4905 | -0.773823188 | 0.190489212 | -5.073997614 | 3.35826E-05 | 0.000846068 | 2.246265549 |
| ARL4C | 10123 | 0.608405644 | -0.126896926 | 5.073976875 | 3.35843E-05 | 0.000846068 | 2.246214095 |
| SMU1 | 55234 | -1.86301104 | -0.143719408 | -5.072826635 | 3.36827E-05 | 0.000848004 | 2.24336026 |
| PGLS | 25796 | 0.829650987 | -0.157349169 | 5.072485393 | 3.3712E-05 | 0.000848199 | 2.242513606 |
| RAB39B | 116442 | -0.617907656 | 0.098626445 | -5.070737637 | 3.38621E-05 | 0.000850375 | 2.238177193 |
| RABGGTB | 5876 | 0.698355143 | -0.115584873 | 5.07049443 | 3.38831E-05 | 0.000850375 | 2.237573757 |
| C21orf59 | 56683 | -0.979663182 | -0.081976432 | -5.070475664 | 3.38847E-05 | 0.000850375 | 2.237527196 |
| CCL7 | 6354 | 0.410941279 | -0.081510263 | 5.069385975 | 3.39788E-05 | 0.000852192 | 2.234823477 |
| LCE1B | 353132 | 0.780904057 | -0.122311154 | 5.068651968 | 3.40422E-05 | 0.000853242 | 2.233002253 |
| C12orf44 | 60673 | -0.727090382 | 0.047948306 | -5.06771274 | 3.41237E-05 | 0.000854739 | 2.230671806 |
| CCDC65 | 85478 | -0.711759284 | 0.079610335 | -5.066814595 | 3.42017E-05 | 0.000855788 | 2.228443273 |
| CBARA1 | 10367 | 0.734105325 | -0.188038916 | 5.0635127 | 3.44901E-05 | 0.000862276 | 2.220250195 |
| IL22RA1 | 58985 | 1.315536998 | 0.047723254 | 5.06281513 | 3.45514E-05 | 0.000862683 | 2.218519255 |
| LOC442251 | 442251 | 0.8029697 | -0.163693918 | 5.062579901 | 3.45721E-05 | 0.000862683 | 2.217935559 |
| LOC441920 | 441920 | -0.540971287 | 0.030079841 | -5.062051609 | 3.46186E-05 | 0.000863296 | 2.216624649 |
| LRRC43 | 254050 | -0.463917706 | 0.111760328 | -5.061175026 | 3.46958E-05 | 0.000864677 | 2.214449469 |
| MAPK14 | 1432 | -0.750560105 | -0.116365568 | -5.060756999 | 3.47328E-05 | 0.00086505 | 2.213412156 |
| NCKAP5 | 344148 | 0.658526154 | -0.212933225 | 5.060406938 | 3.47637E-05 | 0.000865274 | 2.212543493 |
| SSR3 | 6747 | 1.020262789 | -0.157654024 | 5.060149935 | 3.47864E-05 | 0.000865293 | 2.211905747 |
| LARP6 | 55323 | 0.726332975 | -0.148580739 | 5.059517355 | 3.48425E-05 | 0.00086614 | 2.210336012 |
| CD55 | 1604 | -0.670508036 | -0.197555908 | -5.053357127 | 3.53928E-05 | 0.000877858 | 2.195048901 |
| CDON | 50937 | -0.714535065 | 0.129607374 | -5.053244628 | 3.54029E-05 | 0.000877858 | 2.194769713 |
| ARRDC2 | 27106 | 0.474077265 | -0.08131659 | 5.052869892 | 3.54367E-05 | 0.000878143 | 2.193839739 |
| MAPKBP1 | 23005 | -0.761363478 | 0.18882103 | -5.049415901 | 3.57494E-05 | 0.000885337 | 2.185267851 |
| CNR2 | 1269 | -0.733193854 | -0.158083348 | -5.048482911 | 3.58344E-05 | 0.000886884 | 2.182952361 |
| LATS1 | 9113 | 1.060286252 | -0.065266642 | 5.047312964 | 3.59412E-05 | 0.000888413 | 2.180048755 |
| CDC40 | 51362 | 0.747574253 | -0.207940937 | 5.046561916 | 3.601E-05 | 0.000888742 | 2.178184765 |
| Septin 6 | 23157 | 1.14171942 | -0.216736572 | 5.046428761 | 3.60222E-05 | 0.000888742 | 2.177854293 |
| EFNB2 | 1948 | -0.633339064 | -0.11876637 | -5.043043894 | 3.63338E-05 | 0.000895309 | 2.169453339 |
| LOC649210 | 649210 | 0.539600556 | -0.083302506 | 5.042052816 | 3.64255E-05 | 0.000897009 | 2.166993509 |
| LOC440268 | 440268 | 0.842353981 | -0.035884396 | 5.041392308 | 3.64868E-05 | 0.000897957 | 2.165354131 |
| AFF4 | 27125 | 1.178834207 | -0.126084617 | 5.040831764 | 3.65388E-05 | 0.000898678 | 2.163962852 |
| CD207 | 50489 | -0.925661971 | -0.300692845 | -5.039233647 | 3.66877E-05 | 0.000901777 | 2.159996254 |
| C12orf10 | 60314 | 0.771329078 | -0.115056137 | 5.03799078 | 3.6804E-05 | 0.00090407 | 2.156911354 |
| LOC650673 | 650673 | -0.588516752 | 0.070819569 | -5.036517952 | 3.69422E-05 | 0.0009069 | 2.153255618 |
| KCNN3 | 3782 | 0.568902251 | -0.083790007 | 5.032346701 | 3.73364E-05 | 0.000915438 | 2.142901754 |
| DEF8 | 54849 | 0.474163336 | -0.05820565 | 5.031767854 | 3.73914E-05 | 0.000916218 | 2.141464907 |
| KCNJ5 | 3762 | 0.654629459 | -0.030728153 | 5.02855002 | 3.76989E-05 | 0.000922033 | 2.133477245 |
| RUVBL1 | 8607 | -0.661774571 | 0.12090438 | -5.027440872 | 3.78055E-05 | 0.000924066 | 2.130723932 |
| LOC730109 | 730109 | -0.73900269 | 0.131069574 | -5.025730346 | 3.79704E-05 | 0.000927523 | 2.126477715 |
| MYH16 | 84176 | 0.637535752 | 0.090051627 | 5.023600709 | 3.81768E-05 | 0.000931987 | 2.121190991 |
| LRRC25 | 126364 | -0.61094596 | 0.121450277 | -5.022598123 | 3.82744E-05 | 0.00093379 | 2.118702078 |
| TRPV1 | 7442 | 0.949125281 | -0.079929454 | 5.021151015 | 3.84156E-05 | 0.000936078 | 2.115109594 |
| LOC651829 | 651829 | 0.841577811 | -0.181770171 | 5.020536518 | 3.84757E-05 | 0.000936425 | 2.113584074 |
| OXGR1 | 27199 | -0.869883988 | 0.121576159 | -5.020386028 | 3.84905E-05 | 0.000936425 | 2.113210475 |
| SLC22A2 | 6582 | 0.986538391 | -0.016617899 | 5.020277701 | 3.85011E-05 | 0.000936425 | 2.112941546 |
| BRWD3 | 254065 | 0.637337463 | -0.164668711 | 5.01947673 | 3.85797E-05 | 0.000937757 | 2.110953072 |
| PM20D1 | 148811 | -0.590572796 | 0.108171473 | -5.018477677 | 3.86779E-05 | 0.000939566 | 2.108472825 |
| OR7E24 | 26648 | -0.602543367 | 0.11339867 | -5.01395555 | 3.91257E-05 | 0.000948691 | 2.097245883 |
| C1orf91 | 56063 | -0.542575487 | 0.0268594 | -5.009236369 | 3.95986E-05 | 0.000959567 | 2.085529181 |
| CCDC16 | 91603 | -0.755611562 | 0.134419797 | -5.008831762 | 3.96395E-05 | 0.000959966 | 2.084524604 |
| CD36 | 948 | 0.565192997 | -0.146203106 | 5.006432733 | 3.98823E-05 | 0.000965255 | 2.078568103 |
| WWP1 | 11059 | 0.435909236 | -0.075269073 | 5.0051598 | 4.00118E-05 | 0.00096747 | 2.075407508 |
| ZNF232 | 7775 | -0.447860668 | 0.092587927 | -5.004849152 | 4.00434E-05 | 0.00096747 | 2.074636185 |
| CD160 | 11126 | 0.676268563 | -0.011858277 | 5.004809648 | 4.00475E-05 | 0.00096747 | 2.074538099 |
| TERT | 7015 | -0.744895688 | 0.140641556 | -5.00454678 | 4.00743E-05 | 0.000967525 | 2.073885412 |
| GPX5 | 2880 | -0.760352913 | 0.143471122 | -5.003609264 | 4.017E-05 | 0.000969244 | 2.071557592 |
| SAMD4A | 23034 | 0.569629453 | -0.009180591 | 5.00209101 | 4.03256E-05 | 0.000971808 | 2.067787776 |
| XGPY2 | 100132596 | 1.101466127 | -0.104546852 | 5.001593944 | 4.03767E-05 | 0.000971851 | 2.066553553 |
| UNC119 | 9094 | -0.849397665 | 0.147364473 | -4.998088777 | 4.07387E-05 | 0.000978771 | 2.057849995 |
| ZNF791 | 163049 | -0.805929337 | 0.132381901 | -4.99743553 | 4.08065E-05 | 0.000979804 | 2.056227908 |
| CFHR1 | 3078 | -0.939434089 | -0.008707656 | -4.996802348 | 4.08724E-05 | 0.000980787 | 2.054655637 |
| HCRT | 3060 | -0.584119739 | 0.155522139 | -4.996431003 | 4.0911E-05 | 0.000981118 | 2.053733534 |
| LOC645551 | 645551 | 0.641067241 | -0.177026637 | 4.993933232 | 4.11721E-05 | 0.000986778 | 2.047531139 |
| TRAPPC4 | 51399 | -1.024629845 | -0.465377505 | -4.993485762 | 4.1219E-05 | 0.000987303 | 2.046419977 |
| PSMC4 | 5704 | -1.326642871 | 0.364814589 | -4.992761298 | 4.12951E-05 | 0.000988526 | 2.044620975 |
| FAM188B | 84182 | 0.870753453 | -0.078649873 | 4.989818402 | 4.16058E-05 | 0.000995223 | 2.037312996 |
| ALPP | 250 | -0.871937279 | 0.128273229 | -4.989633382 | 4.16254E-05 | 0.000995223 | 2.036853535 |
| SYNM | 23336 | 0.806343908 | -0.163709951 | 4.988036399 | 4.1795E-05 | 0.000998673 | 2.032887721 |
