# Supplementary material for: Establishment of a Combined Diagnostic Model of Abdominal Aortic Aneurysm with Random Forest and Artificial Neural Network
Source: Biomed Res Int. 2022 Mar 7;2022:7173972. doi: 10.1155/2022/7173972 (PMC8922147; doi:10.1155/2022/7173972)
Supplement: Supplementary 3 — Supplementary Table 3: Significantly enriched GO terms in BP, CC, and MF from GSE57691 dataset. [file 7173972.f3.docx]

| Supplementary Table 3. GO Analysis with DEGs from GSE57691 (2486) | | | | | |  |  |  |  |  |  |  |
| --- | --- | --- | --- | --- | --- | --- | --- | --- | --- | --- | --- | --- |
| Category | Term | Count | % | PValue | Genes | List Total | Pop Hits | Pop Total | Fold Enrichment | Bonferroni | Benjamini | FDR |
| GOTERM_BP_DIRECT | GO:0045926~negative regulation of growth | 5 | 2.380952381 | 4.36E-05 | MT1A, IGFBP5, MT1M, MT1X, MT1E | 1.81E+02 | 1.90E+01 | 16792 | 24.41407386 | 5.27E-02 | 5.41E-02 | 5.41E-02 |
| GOTERM_BP_DIRECT | GO:0010942~positive regulation of cell death | 5 | 2.380952381 | 2.46E-04 | ZC3H12A, UCP2, AKR1C3, HBB, HBA2 | 1.81E+02 | 2.90E+01 | 16792 | 15.9954277 | 2.63E-01 | 1.52E-01 | 1.52E-01 |
| GOTERM_BP_DIRECT | GO:0071276~cellular response to cadmium ion | 4 | 1.904761905 | 7.37E-04 | MT1A, AKR1C3, MT1X, MT1E | 1.81E+02 | 1.70E+01 | 16792 | 21.82905427 | 5.99E-01 | 3.05E-01 | 3.05E-01 |
| GOTERM_BP_DIRECT | GO:0071294~cellular response to zinc ion | 4 | 1.904761905 | 1.03E-03 | MT1A, MT1M, MT1X, MT1E | 1.81E+02 | 1.90E+01 | 16792 | 19.53125909 | 7.23E-01 | 3.20E-01 | 3.20E-01 |
| GOTERM_BP_DIRECT | GO:0045600~positive regulation of fat cell differentiation | 5 | 2.380952381 | 1.59E-03 | ID2, ZBTB16, LMO3, ZC3H12A, SNAI2 | 1.81E+02 | 4.70E+01 | 16792 | 9.869519219 | 8.61E-01 | 3.94E-01 | 3.94E-01 |
| GOTERM_CC_DIRECT | GO:0005829~cytosol | 51 | 24.28571429 | 3.76E-03 | APP, AHCYL1, NCF2, C2CD2, DIXDC1, HSPB2, HBB, RPL10A, PPP1CB, PPP3CB, MECOM, TNPO1, PDE8B, GSTO1, HBA2, PPM1B, MT1A, PPP1R3C, MTHFD2, MAP1B, IL1B, PDE5A, PADI4, PHPT1, SYNC, OPTN, CD151, RABGAP1, ROCK2, RHOBTB3, PIK3R1, FOXO3, FOXO1, SOCS3, UBB, DNAJB6, GMDS, RAB11FIP3, CLASP1, NQO1, GSDMB, MDH1, ZBTB16, AKR1C3, PUM1, MYO1C, ID2, GPD1L, LPIN1, RPS21, RPS24 | 1.92E+02 | 3.32E+03 | 18224 | 1.46025641 | 6.31E-01 | 6.33E-01 | 6.33E-01 |
| GOTERM_MF_DIRECT | GO:0005515~protein binding | 112 | 53.33333333 | 4.37E-03 | FCN1, APP, AHCYL1, NCF2, DIXDC1, MT1X, RPL10A, PPP1CB, CNST, ZC3H12A, ACVR1, PHYH, IGFBP5, LMO4, LMO3, KLHL42, MT1A, PPP1R3C, MSH3, MAP1B, FEZ1, COL8A1, SIK1, PADI4, OPTN, FKBP5, CD151, RAB5C, NDUFB10, CTBP1, MGST3, COX17, RHOBTB3, LPAR1, PIK3R1, FOXO3, FOXO1, RTN4, MRPL20, SOCS3, ORC6, UBB, RAB11FIP3, WFS1, ZBTB16, HPS6, FBXO32, PUM1, MANBAL, MFHAS1, ID2, OSTC, ID1, CALU, SNAI2, PLXNB1, DYNLRB1, MRAP2, CNIH4, SERPINA3, ALAS2, TGFB1I1, CETN3, CTSZ, HSPB2, HBB, FHL5, CKS1B, PPP3CB, MECOM, RBPMS2, TNPO1, ACP1, SUN2, GSTO1, COMMD3, HBA2, NAV2, COMMD6, ANO1, PPM1B, ZMYND11, SLC25A4, HCST, RABGAP1, ROCK2, DERL1, ATP1A2, CXXC5, PCNP, PGRMC1, DNAJB6, GMDS, POLR2C, UCP2, SPTSSA, CLASP1, ZNF223, CAP2, NQO1, SEMA4A, MDH1, FAM46B, SYVN1, SORBS2, USF1, MYO1C, YIPF6, ESYT2, CLUAP1, SCRG1, RECK | 1.80E+02 | 8.79E+03 | 16881 | 1.19564409 | 8.13E-01 | 1.00E+00 | 1.00E+00 |
| GOTERM_CC_DIRECT | GO:0030176~integral component of endoplasmic reticulum membrane | 6 | 2.857142857 | 4.78E-03 | HACD1, WFS1, DERL1, SYVN1, MARCH6, RTN4 | 1.92E+02 | 1.04E+02 | 18224 | 5.475961538 | 7.19E-01 | 6.33E-01 | 6.33E-01 |
| GOTERM_MF_DIRECT | GO:0051287~NAD binding | 4 | 1.904761905 | 7.53E-03 | AHCYL1, MDH1, CTBP1, GPD1L | 1.80E+02 | 3.80E+01 | 16881 | 9.871929825 | 0.944740583 | 1.00E+00 | 1.00E+00 |
| GOTERM_MF_DIRECT | GO:1990381~ubiquitin-specific protease binding | 3 | 1.428571429 | 8.08E-03 | DERL1, SYVN1, MARCH6 | 1.80E+02 | 1.30E+01 | 16881 | 21.64230769 | 0.955214088 | 1.00E+00 | 1.00E+00 |
| GOTERM_CC_DIRECT | GO:0030018~Z disc | 6 | 2.857142857 | 8.09E-03 | PPP3CB, DNAJB6, NEXN, SORBS2, SYNC, FBXO32 | 192 | 118 | 18224 | 4.826271186 | 0.88385833 | 0.714740857 | 0.714740857 |
| GOTERM_BP_DIRECT | GO:0043124~negative regulation of I-kappaB kinase/NF-kappaB signaling | 4 | 1.904761905 | 8.95E-03 | PPM1B, ZC3H12A, ZMYND11, OPTN | 181 | 40 | 16792 | 9.277348066 | 0.999985533 | 1 | 1 |
| GOTERM_BP_DIRECT | GO:0032869~cellular response to insulin stimulus | 5 | 2.380952381 | 9.41E-03 | UCP2, PIK3R1, LPIN1, FOXO1, USF1 | 181 | 77 | 16792 | 6.024251991 | 0.99999193 | 1 | 1 |
| GOTERM_BP_DIRECT | GO:0036503~ERAD pathway | 3 | 1.428571429 | 1.09E-02 | DERL1, SYVN1, MARCH6 | 181 | 15 | 16792 | 18.55469613 | 0.999998819 | 1 | 1 |
| GOTERM_CC_DIRECT | GO:0005654~nucleoplasm | 42 | 20 | 1.36E-02 | CTBP1, TSEN15, RNF38, CXXC5, FOXO3, ARPP19, FOXO1, CKS1B, IRF2BPL, PPP1CB, PPP3CB, ORC6, DNAJB6, MECOM, UBB, POLR2C, ZC3H12A, BBX, HADH, ZBED5, RAB11FIP3, SYVN1, VEZF1, NAV2, FBXO32, USF1, LYRM1, TOX2, MYO1C, NFIA, MSH3, ID2, ID1, KLF9, PADI4, LPIN1, ZMYND11, CLUAP1, RPS21, RPS24, OPTN, FKBP5 | 192 | 2784 | 18224 | 1.431932471 | 0.9737137 | 0.732787131 | 0.732787131 |
| GOTERM_MF_DIRECT | GO:0016616~oxidoreductase activity, acting on the CH-OH group of donors, NAD or NADP as acceptor | 3 | 1.428571429 | 1.37E-02 | MDH1, RDH11, CTBP1 | 180 | 17 | 16881 | 16.55 | 0.994917832 | 1 | 1 |
| GOTERM_MF_DIRECT | GO:0017137~Rab GTPase binding | 6 | 2.857142857 | 1.41E-02 | RABGAP1, RHOBTB3, HPS6, RAB11FIP3, RAB3GAP1, OPTN | 180 | 134 | 16881 | 4.199253731 | 0.995696607 | 1 | 1 |
| GOTERM_BP_DIRECT | GO:0030335~positive regulation of cell migration | 7 | 3.333333333 | 1.45E-02 | ACVR1, SUN2, SEMA4A, MYO1C, MCAM, SNAI2, PIK3R1 | 181 | 184 | 16792 | 3.529425895 | 0.999999986 | 1 | 1 |
| GOTERM_CC_DIRECT | GO:0005813~centrosome | 11 | 5.238095238 | 1.47E-02 | RABGAP1, MDH1, ROCK2, ID2, FEZ1, ID1, CETN3, DYNLRB1, CLUAP1, RAB11FIP3, CLASP1 | 192 | 426 | 18224 | 2.450899844 | 0.98025954 | 0.732787131 | 0.732787131 |
| GOTERM_CC_DIRECT | GO:0005743~mitochondrial inner membrane | 11 | 5.238095238 | 1.81E-02 | MRPL20, NDUFA8, ALAS2, C19ORF70, NDUFB10, NDUFA12, UCP2, ATP5I, HADH, HIGD1A, SLC25A4 | 192 | 441 | 18224 | 2.367535903 | 0.992167099 | 0.732787131 | 0.732787131 |
| GOTERM_BP_DIRECT | GO:0008344~adult locomotory behavior | 4 | 1.904761905 | 1.82E-02 | APP, ID2, ATP1A2, PUM1 | 181 | 52 | 16792 | 7.136421589 | 1 | 1 | 1 |
| GOTERM_BP_DIRECT | GO:0043153~entrainment of circadian clock by photoperiod | 3 | 1.428571429 | 1.91E-02 | PPP1CB, ID2, SIK1 | 181 | 20 | 16792 | 13.9160221 | 1 | 1 | 1 |
| GOTERM_CC_DIRECT | GO:0005789~endoplasmic reticulum membrane | 17 | 8.095238095 | 1.94E-02 | HACD1, AHCYL1, CERS6, ACSL1, WFS1, MGST3, DERL1, SYVN1, RTN4, PGRMC1, RDH11, CALU, LPIN1, MRAP2, RAB3GAP1, FKBP5, TMED4 | 192 | 862 | 18224 | 1.871906419 | 0.994370896 | 0.732787131 | 0.732787131 |
| GOTERM_MF_DIRECT | GO:0004867~serine-type endopeptidase inhibitor activity | 5 | 2.380952381 | 1.97E-02 | ITIH5, SERPINA3, APP, SPINT2, RECK | 180 | 97 | 16881 | 4.83419244 | 0.999519058 | 1 | 1 |
| GOTERM_BP_DIRECT | GO:0045665~negative regulation of neuron differentiation | 4 | 1.904761905 | 0.022170736 | APP, ID2, DIXDC1, FOXO3 | 181 | 56 | 16792 | 6.62667719 | 1 | 1 | 1 |
| GOTERM_MF_DIRECT | GO:0004601~peroxidase activity | 3 | 1.428571429 | 0.022473412 | MGST3, HBB, HBA2 | 180 | 22 | 16881 | 12.78863636 | 0.999834329 | 1 | 1 |
| GOTERM_MF_DIRECT | GO:0004722~protein serine/threonine phosphatase activity | 4 | 1.904761905 | 0.02362919 | PPP1CB, PPM1B, PPP3CB, PPP1R3C | 180 | 58 | 16881 | 6.467816092 | 0.999894691 | 1 | 1 |
| GOTERM_BP_DIRECT | GO:0030433~ER-associated ubiquitin-dependent protein catabolic process | 4 | 1.904761905 | 0.026537685 | WFS1, DERL1, SYVN1, MARCH6 | 181 | 60 | 16792 | 6.184898711 | 1 | 1 | 1 |
| GOTERM_BP_DIRECT | GO:0048675~axon extension | 3 | 1.428571429 | 0.027020789 | PPP3CB, MAP1B, PLXNB1 | 181 | 24 | 16792 | 11.59668508 | 1 | 1 | 1 |
| GOTERM_CC_DIRECT | GO:0005783~endoplasmic reticulum | 16 | 7.619047619 | 0.027995633 | HACD1, CERS6, WFS1, MGST3, TMEM50B, CTSZ, DERL1, SYVN1, HPS6, RTN4, PGRMC1, CALU, YIPF6, METTL7A, MRAP2, CNIH4 | 192 | 828 | 18224 | 1.834138486 | 0.999460393 | 0.886722293 | 0.886722293 |
| GOTERM_CC_DIRECT | GO:0030134~ER to Golgi transport vesicle | 3 | 1.428571429 | 0.030115097 | APP, CTSZ, YIPF6 | 192 | 26 | 18224 | 10.95192308 | 0.999697411 | 0.886722293 | 0.886722293 |
| GOTERM_MF_DIRECT | GO:0031720~haptoglobin binding | 2 | 0.952380952 | 0.031476637 | HBB, HBA2 | 180 | 3 | 16881 | 62.52222222 | 0.999995212 | 1 | 1 |
| GOTERM_BP_DIRECT | GO:0001525~angiogenesis | 7 | 3.333333333 | 0.033207442 | SEMA4A, MCAM, ZC3H12A, ID1, PROK2, COL8A1, VEZF1 | 181 | 223 | 16792 | 2.912172039 | 1 | 1 | 1 |
| GOTERM_BP_DIRECT | GO:0007219~Notch signaling pathway | 5 | 2.380952381 | 0.035264718 | APP, UBB, GMDS, SNAI2, TIMP4 | 181 | 115 | 16792 | 4.033629594 | 1 | 1 | 1 |
| GOTERM_BP_DIRECT | GO:0035774~positive regulation of insulin secretion involved in cellular response to glucose stimulus | 3 | 1.428571429 | 0.038391296 | ANO1, PPP3CB, PHPT1 | 181 | 29 | 16792 | 9.59725662 | 1 | 1 | 1 |
| GOTERM_BP_DIRECT | GO:0007163~establishment or maintenance of cell polarity | 3 | 1.428571429 | 0.038391296 | SPINT2, CLASP1, CAP2 | 181 | 29 | 16792 | 9.59725662 | 1 | 1 | 1 |
| GOTERM_BP_DIRECT | GO:0098869~cellular oxidant detoxification | 4 | 1.904761905 | 0.039310664 | GSTO1, MGST3, HBB, HBA2 | 181 | 70 | 16792 | 5.301341752 | 1 | 1 | 1 |
| GOTERM_CC_DIRECT | GO:0031838~haptoglobin-hemoglobin complex | 2 | 0.952380952 | 0.041271607 | HBB, HBA2 | 192 | 4 | 18224 | 47.45833333 | 0.999985896 | 1 | 1 |
| GOTERM_BP_DIRECT | GO:0010951~negative regulation of endopeptidase activity | 5 | 2.380952381 | 0.04128137 | ITIH5, SERPINA3, APP, SPINT2, TIMP4 | 181 | 121 | 16792 | 3.833614903 | 1 | 1 | 1 |
| GOTERM_BP_DIRECT | GO:0070293~renal absorption | 2 | 0.952380952 | 0.042196751 | AKR1C3, HBB | 181 | 4 | 16792 | 46.38674033 | 1 | 1 | 1 |
| GOTERM_BP_DIRECT | GO:0070164~negative regulation of adiponectin secretion | 2 | 0.952380952 | 0.042196751 | IL1B, RAB11FIP3 | 181 | 4 | 16792 | 46.38674033 | 1 | 1 | 1 |
| GOTERM_BP_DIRECT | GO:0055118~negative regulation of cardiac muscle contraction | 2 | 0.952380952 | 0.042196751 | ZC3H12A, PDE5A | 181 | 4 | 16792 | 46.38674033 | 1 | 1 | 1 |
| GOTERM_BP_DIRECT | GO:1990090~cellular response to nerve growth factor stimulus | 3 | 1.428571429 | 0.043365649 | APP, ID1, FOXO3 | 181 | 31 | 16792 | 8.978078774 | 1 | 1 | 1 |
| GOTERM_MF_DIRECT | GO:0008022~protein C-terminus binding | 6 | 2.857142857 | 0.044505341 | MYO1C, NCF2, CTBP1, ZBTB16, ID1, OPTN | 180 | 182 | 16881 | 3.091758242 | 0.999999973 | 1 | 1 |
| GOTERM_BP_DIRECT | GO:0030334~regulation of cell migration | 4 | 1.904761905 | 0.045148564 | LMO4, NEXN, PLXNB1, RTN4 | 181 | 74 | 16792 | 5.014782739 | 1 | 1 | 1 |
| GOTERM_BP_DIRECT | GO:0000122~negative regulation of transcription from RNA polymerase II promoter | 14 | 6.666666667 | 0.04639644 | WFS1, CTBP1, ZBTB16, CXXC5, FOXO3, FOXO1, IRF2BPL, NFIA, UBB, ID2, ID1, FOSB, SNAI2, ZMYND11 | 181 | 720 | 16792 | 1.803928791 | 1 | 1 | 1 |
| GOTERM_BP_DIRECT | GO:0006470~protein dephosphorylation | 5 | 2.380952381 | 0.046712866 | PPP1CB, PPM1B, PPP3CB, PPP1R3C, PHPT1 | 181 | 126 | 16792 | 3.681487328 | 1 | 1 | 1 |
| GOTERM_BP_DIRECT | GO:0045893~positive regulation of transcription, DNA-templated | 11 | 5.238095238 | 0.051052117 | ACVR1, PPP3CB, MECOM, TGFB1I1, IL1B, ID2, ZBTB16, ATOH8, FOXO3, FOXO1, CKS1B | 181 | 515 | 16792 | 1.98156949 | 1 | 1 | 1 |
| GOTERM_CC_DIRECT | GO:0005925~focal adhesion | 9 | 4.285714286 | 0.054072156 | PPP1CB, CD151, TGFB1I1, MCAM, DIXDC1, NEXN, SORBS2, RPL10A, CLASP1 | 192 | 391 | 18224 | 2.184782609 | 0.9999996 | 1 | 1 |
| GOTERM_BP_DIRECT | GO:0055114~oxidation-reduction process | 12 | 5.714285714 | 0.054276002 | NQO1, NDUFA8, MAOA, NCF2, RDH11, GSTO1, MTHFD2, CTBP1, AKR1C3, GPD1L, HIGD1A, PCYOX1 | 181 | 592 | 16792 | 1.880543527 | 1 | 1 | 1 |
| GOTERM_CC_DIRECT | GO:0070062~extracellular exosome | 39 | 18.57142857 | 0.056234427 | SERPINA3, APP, AHCYL1, RAB5C, NDUFB10, MGST3, CTSZ, RHOBTB3, HBB, COX7A2, SLC2A3, RPL10A, RTN4, PPP1CB, PGRMC1, CLMP, GMDS, UBB, METTL7A, TNPO1, ST3GAL1, ACP1, CLASP1, TMED4, NQO1, MDH1, GSTO1, AKR1C3, HBA2, ANO1, PTP4A2, MYO1C, IL1B, COL8A1, GPD1L, PHPT1, RAB3GAP1, FKBP5, PCYOX1 | 192 | 2811 | 18224 | 1.316880114 | 0.999999782 | 1 | 1 |
| GOTERM_BP_DIRECT | GO:0006468~protein phosphorylation | 10 | 4.761904762 | 0.057423143 | ACVR1, APP, PPP3CB, ROCK2, CTBP1, SIK1, PIK3R1, MAP3K6, ST3GAL1, HCST | 181 | 456 | 16792 | 2.034506155 | 1 | 1 | 1 |
| GOTERM_BP_DIRECT | GO:0000086~G2/M transition of mitotic cell cycle | 5 | 2.380952381 | 0.05999171 | PPP1CB, UBB, ARPP19, CLASP1, OPTN | 181 | 137 | 16792 | 3.385893455 | 1 | 1 | 1 |
| GOTERM_BP_DIRECT | GO:0009987~cellular process | 2 | 0.952380952 | 0.062626273 | APP, MAP1B | 181 | 6 | 16792 | 30.92449355 | 1 | 1 | 1 |
| GOTERM_BP_DIRECT | GO:0045668~negative regulation of osteoblast differentiation | 3 | 1.428571429 | 0.065398143 | IGFBP5, ID2, ID1 | 181 | 39 | 16792 | 7.136421589 | 1 | 1 | 1 |
| GOTERM_BP_DIRECT | GO:0018279~protein N-linked glycosylation via asparagine | 3 | 1.428571429 | 0.06836928 | OSTC, SYVN1, ST3GAL1 | 181 | 40 | 16792 | 6.95801105 | 1 | 1 | 1 |
| GOTERM_CC_DIRECT | GO:0043231~intracellular membrane-bounded organelle | 11 | 5.238095238 | 0.069714937 | APP, AHCYL1, RAB5C, MECOM, MGST3, CTSZ, COL8A1, VEZF1, ATP1A2, CLUAP1, RAB11FIP3 | 192 | 558 | 18224 | 1.871117085 | 0.999999995 | 1 | 1 |
| GOTERM_BP_DIRECT | GO:0006734~NADH metabolic process | 2 | 0.952380952 | 0.07267794 | MDH1, GPD1L | 181 | 7 | 16792 | 26.50670876 | 1 | 1 | 1 |
| GOTERM_BP_DIRECT | GO:0045722~positive regulation of gluconeogenesis | 2 | 0.952380952 | 0.07267794 | ARPP19, FOXO1 | 181 | 7 | 16792 | 26.50670876 | 1 | 1 | 1 |
| GOTERM_BP_DIRECT | GO:0042178~xenobiotic catabolic process | 2 | 0.952380952 | 0.07267794 | ACSL1, GSTO1 | 181 | 7 | 16792 | 26.50670876 | 1 | 1 | 1 |
| GOTERM_BP_DIRECT | GO:0021554~optic nerve development | 2 | 0.952380952 | 0.07267794 | LPAR1, NAV2 | 181 | 7 | 16792 | 26.50670876 | 1 | 1 | 1 |
| GOTERM_BP_DIRECT | GO:1903140~regulation of establishment of endothelial barrier | 2 | 0.952380952 | 0.07267794 | ROCK2, IL1B | 181 | 7 | 16792 | 26.50670876 | 1 | 1 | 1 |
| GOTERM_MF_DIRECT | GO:0046983~protein dimerization activity | 5 | 2.380952381 | 0.075489015 | ANO1, PPP3CB, ID2, POLR2C, ATOH8 | 180 | 150 | 16881 | 3.126111111 | 1 | 1 | 1 |
| GOTERM_CC_DIRECT | GO:0016020~membrane | 31 | 14.76190476 | 0.078758031 | CD151, NDUFA12, MGST3, DERL1, ATP1A2, PIK3R1, RPL10A, FOXO3, PGRMC1, CNST, ORC6, DNAJB6, METTL7A, ST3GAL1, CLASP1, ACVR1, CERS6, ACSL1, SYVN1, HBA2, HPS6, MARCH6, PPM1B, MYO1C, MSH3, CALU, ESYT2, DYNLRB1, RECK, RPS24, FKBP5 | 192 | 2200 | 18224 | 1.337462121 | 1 | 1 | 1 |
| GOTERM_MF_DIRECT | GO:0008134~transcription factor binding | 7 | 3.333333333 | 0.082204158 | NFIA, LMO4, CTBP1, ID1, FOSB, PIK3R1, CXXC5 | 180 | 284 | 16881 | 2.311561033 | 1 | 1 | 1 |
| GOTERM_MF_DIRECT | GO:0004721~phosphoprotein phosphatase activity | 3 | 1.428571429 | 0.082316147 | PPP1CB, PPP3CB, PHPT1 | 180 | 45 | 16881 | 6.252222222 | 1 | 1 | 1 |
| GOTERM_BP_DIRECT | GO:2000177~regulation of neural precursor cell proliferation | 2 | 0.952380952 | 0.082622412 | FOXO3, FOXO1 | 181 | 8 | 16792 | 23.19337017 | 1 | 1 | 1 |
| GOTERM_BP_DIRECT | GO:0030968~endoplasmic reticulum unfolded protein response | 3 | 1.428571429 | 0.083856974 | WFS1, DERL1, SYVN1 | 181 | 45 | 16792 | 6.184898711 | 1 | 1 | 1 |
| GOTERM_BP_DIRECT | GO:0007049~cell cycle | 6 | 2.857142857 | 0.084777112 | PCNP, RABGAP1, DIXDC1, SIK1, ZMYND11, CKS1B | 181 | 217 | 16792 | 2.565165364 | 1 | 1 | 1 |
| GOTERM_BP_DIRECT | GO:0010595~positive regulation of endothelial cell migration | 3 | 1.428571429 | 0.08707234 | ROCK2, ATOH8, ZC3H12A | 181 | 46 | 16792 | 6.050444391 | 1 | 1 | 1 |
| GOTERM_MF_DIRECT | GO:0003735~structural constituent of ribosome | 6 | 2.857142857 | 0.087773902 | MRPL20, UCP2, RPL10A, RPS21, SLC25A4, RPS24 | 180 | 222 | 16881 | 2.534684685 | 1 | 1 | 1 |
| GOTERM_CC_DIRECT | GO:0043197~dendritic spine | 4 | 1.904761905 | 0.08787305 | APP, MAP1B, LPAR1, ATP1A2 | 192 | 100 | 18224 | 3.796666667 | 1 | 1 | 1 |
| GOTERM_BP_DIRECT | GO:0007399~nervous system development | 7 | 3.333333333 | 0.088744216 | APP, MAP1B, FEZ1, ATOH8, ZC3H12A, NAV2, SCRG1 | 181 | 287 | 16792 | 2.262767821 | 1 | 1 | 1 |
| GOTERM_CC_DIRECT | GO:0022627~cytosolic small ribosomal subunit | 3 | 1.428571429 | 0.090130193 | HBA2, RPS21, RPS24 | 192 | 48 | 18224 | 5.932291667 | 1 | 1 | 1 |
| GOTERM_MF_DIRECT | GO:0052650~NADP-retinol dehydrogenase activity | 2 | 0.952380952 | 0.091504342 | RDH11, AKR1C3 | 180 | 9 | 16881 | 20.84074074 | 1 | 1 | 1 |
| GOTERM_MF_DIRECT | GO:1904264~ubiquitin protein ligase activity involved in ERAD pathway | 2 | 0.952380952 | 0.091504342 | SYVN1, MARCH6 | 180 | 9 | 16881 | 20.84074074 | 1 | 1 | 1 |
| GOTERM_MF_DIRECT | GO:0008137~NADH dehydrogenase (ubiquinone) activity | 3 | 1.428571429 | 0.091911039 | NDUFA8, NDUFB10, NDUFA12 | 180 | 48 | 16881 | 5.861458333 | 1 | 1 | 1 |
| GOTERM_BP_DIRECT | GO:0047497~mitochondrion transport along microtubule | 2 | 0.952380952 | 0.092460828 | UBB, MAP1B | 181 | 9 | 16792 | 20.61632904 | 1 | 1 | 1 |
| GOTERM_BP_DIRECT | GO:0019852~L-ascorbic acid metabolic process | 2 | 0.952380952 | 0.092460828 | GSTO1, SLC2A3 | 181 | 9 | 16792 | 20.61632904 | 1 | 1 | 1 |
| GOTERM_BP_DIRECT | GO:2000810~regulation of bicellular tight junction assembly | 2 | 0.952380952 | 0.092460828 | MYO1C, SNAI2 | 181 | 9 | 16792 | 20.61632904 | 1 | 1 | 1 |
| GOTERM_BP_DIRECT | GO:0016567~protein ubiquitination | 8 | 3.80952381 | 0.092929301 | PCNP, KLHL9, ZYG11B, SOCS3, ZBTB16, RNF38, SYVN1, FBXO32 | 181 | 359 | 16792 | 2.067375614 | 1 | 1 | 1 |
| GOTERM_CC_DIRECT | GO:0005747~mitochondrial respiratory chain complex I | 3 | 1.428571429 | 0.093341452 | NDUFA8, NDUFB10, NDUFA12 | 192 | 49 | 18224 | 5.81122449 | 1 | 1 | 1 |
| GOTERM_BP_DIRECT | GO:0030148~sphingolipid biosynthetic process | 3 | 1.428571429 | 0.093611477 | HACD1, CERS6, SPTSSA | 181 | 48 | 16792 | 5.798342541 | 1 | 1 | 1 |
| GOTERM_BP_DIRECT | GO:0000910~cytokinesis | 3 | 1.428571429 | 0.093611477 | KLHL9, ROCK2, RAB11FIP3 | 181 | 48 | 16792 | 5.798342541 | 1 | 1 | 1 |
| GOTERM_BP_DIRECT | GO:0006120~mitochondrial electron transport, NADH to ubiquinone | 3 | 1.428571429 | 0.096932984 | NDUFA8, NDUFB10, NDUFA12 | 181 | 49 | 16792 | 5.68000902 | 1 | 1 | 1 |
| GOTERM_BP_DIRECT | GO:0042752~regulation of circadian rhythm | 3 | 1.428571429 | 0.096932984 | PPP1CB, ROCK2, ID2 | 181 | 49 | 16792 | 5.68000902 | 1 | 1 | 1 |
