# Supplementary material for: Establishment of a Combined Diagnostic Model of Abdominal Aortic Aneurysm with Random Forest and Artificial Neural Network
Source: Biomed Res Int. 2022 Mar 7;2022:7173972. doi: 10.1155/2022/7173972 (PMC8922147; doi:10.1155/2022/7173972)
Supplement: Supplementary 4 — Supplementary Table 4: KEGG Analysis with DEGs from GSE57691 dataset. [file 7173972.f4.docx]

| Supplementary Table 4. KEGG Analysis with DEGs from GSE57691 (2486) | | | | |
| --- | --- | --- | --- | --- |
| ID | Description | p.adjust | geneID |  |
| KEGG_PATHWAY | hsa04978:Mineral absorption | 3.47E-01 | MT1A, MT1M, MT1X, ATP1A2, MT1E |  |
| KEGG_PATHWAY | hsa04932:Non-alcoholic fatty liver disease (NAFLD) | 0.42659596 | SOCS3, NDUFA8, NDUFB10, NDUFA12, IL1B, COX7A2, PIK3R1, COX7A1 |  |
| KEGG_PATHWAY | hsa05010:Alzheimer's disease | 0.633380331 | NDUFA8, APP, PPP3CB, NDUFB10, NDUFA12, IL1B, COX7A2, COX7A1 |  |
| KEGG_PATHWAY | hsa00190:Oxidative phosphorylation | 0.718798154 | NDUFA8, NDUFB10, NDUFA12, COX17, COX7A2, ATP5I, COX7A1 |  |
| KEGG_PATHWAY | hsa05012:Parkinson's disease | 0.823135152 | NDUFA8, UBB, NDUFB10, NDUFA12, COX7A2, COX7A1, SLC25A4 |  |
| KEGG_PATHWAY | hsa04380:Osteoclast differentiation | 0.992055218 | SOCS3, PPP3CB, NCF2, IL1B, FOSB, PIK3R1 |  |
| KEGG_PATHWAY | hsa05016:Huntington's disease | 0.998782235 | NDUFA8, NDUFB10, NDUFA12, POLR2C, COX7A2, COX7A1, SLC25A4 |  |
| KEGG_PATHWAY | hsa04931:Insulin resistance | 0.999915495 | PPP1CB, SOCS3, PPP1R3C, PIK3R1, FOXO1 |  |
| KEGG_PATHWAY | hsa04022:cGMP-PKG signaling pathway | 0.999941775 | PPP1CB, PPP3CB, ROCK2, ATP1A2, PDE5A, SLC25A4 |  |
| KEGG_PATHWAY | hsa05031:Amphetamine addiction | 0.999955279 | PPP1CB, PPP3CB, MAOA, FOSB |  |
| KEGG_PATHWAY | hsa05143:African trypanosomiasis | 0.999997597 | IL1B, HBB, HBA2 |  |
| KEGG_PATHWAY | hsa01100:Metabolic pathways | 0.999999878 | NDUFA8, ALAS2, AHCYL1, CERS6, NDUFB10, MDH1, MAOA, GALNT15, ACSL1, NDUFA12, COX17, AKR1C3, ATP5I, GMDS, RDH11, MTHFD2, POLR2C, DBT, CHSY3, LPIN1, HADH, ST3GAL1 |  |
| KEGG_PATHWAY | hsa04910:Insulin signaling pathway | 0.999999997 | PPP1CB, SOCS3, PPP1R3C, PIK3R1, FOXO1 |  |
