# Supplementary material for: Establishment of a Combined Diagnostic Model of Abdominal Aortic Aneurysm with Random Forest and Artificial Neural Network
Source: Biomed Res Int. 2022 Mar 7;2022:7173972. doi: 10.1155/2022/7173972 (PMC8922147; doi:10.1155/2022/7173972)
Supplement: Supplementary 5 — Supplementary Table 5: Random Forest Selected Genes. [file 7173972.f5.docx]

| Supplementary Table 5. Random Forest Selected Genes | | | | |
| --- | --- | --- | --- | --- |
| Genes | AAA | Control | MeanDecreaseAccuracy | MeanDecreaseGini |
| FAM19A4 | 0.000176894 | 0.001575489 | 0.000327542 | 0.056210194 |
| CNTN6 | 0.000218126 | 0.001139716 | 0.000344353 | 0.060955795 |
| VEZF1 | 0.000173367 | 0.001367659 | 0.000347615 | 0.073109134 |
| LOC391509 | 0.000127711 | 0.001779968 | 0.000359763 | 0.058025386 |
| ARPP19 | 0.000201533 | 0.001300617 | 0.000364063 | 0.052630736 |
| ZNF331 | 0.000201326 | 0.002031376 | 0.000410763 | 0.073558316 |
| EDA | 0.000322238 | 0.001079378 | 0.000451602 | 0.052477073 |
| PDE3A | 0.00029822 | 0.001501743 | 0.000457542 | 0.056172358 |
| OR4D1 | 0.000319738 | 0.000976899 | 0.000458161 | 0.058366429 |
| CSNK2A2 | 0.000274356 | 0.001810137 | 0.000473606 | 0.055804586 |
| GPR109B | 0.000234364 | 0.002339769 | 0.000490387 | 0.067013939 |
| BAZ2A | 0.000195124 | 0.002614642 | 0.000514837 | 0.050592241 |
| LOC642691 | 0.000392745 | 0.001267096 | 0.000527724 | 0.076006842 |
| RNASEN | 0.000283639 | 0.00160901 | 0.000531844 | 0.058424331 |
| CHAT | 0.000283954 | 0.002219094 | 0.000536307 | 0.062543878 |
| LOC442421 | 0.00034467 | 0.001206758 | 0.000536621 | 0.053021591 |
| LOC642787 | 0.000306032 | 0.001870475 | 0.000552018 | 0.059882951 |
| ABI2 | 0.000265005 | 0.002768839 | 0.000552317 | 0.069110744 |
| C17orf38 | 0.000293213 | 0.002440333 | 0.000578403 | 0.06437379 |
| CXXC5 | 0.000412027 | 0.001857066 | 0.00058446 | 0.093822623 |
| LOC149950 | 0.000396393 | 0.001843658 | 0.000598887 | 0.053856076 |
| FLJ00312 | 0.000352206 | 0.002346474 | 0.000611242 | 0.053241111 |
| RAB27A | 0.000435353 | 0.001441405 | 0.000630061 | 0.072883473 |
| ROCK1 | 0.000291014 | 0.00250067 | 0.00063893 | 0.071115071 |
| DHX40 | 0.000379617 | 0.002507375 | 0.000652054 | 0.061649349 |
| PPP4R1L | 0.000334693 | 0.002241122 | 0.000655025 | 0.057787254 |
| LOC652698 | 0.000242012 | 0.003204612 | 0.000673537 | 0.081597042 |
| LOC653867 | 0.000329315 | 0.00364709 | 0.000675284 | 0.080896386 |
| LOC645349 | 0.000444935 | 0.002105122 | 0.000676754 | 0.066639784 |
| BDKRB2 | 0.000379368 | 0.002681684 | 0.000679124 | 0.05247764 |
| LOC652479 | 0.000444635 | 0.002152051 | 0.000684573 | 0.078001702 |
| ARMCX4 | 0.00045583 | 0.001682757 | 0.000688823 | 0.084255685 |
| NEB | 0.00029564 | 0.003298471 | 0.000696165 | 0.072440417 |
| SPAG5 | 0.000283831 | 0.00356664 | 0.00071665 | 0.088866398 |
| CTBP1 | 0.000488847 | 0.002648163 | 0.000752137 | 0.087207084 |
| DHRS12 | 0.000480431 | 0.002286136 | 0.000753693 | 0.082621704 |
| C2orf65 | 0.000424444 | 0.002668276 | 0.000756262 | 0.094316469 |
| CLDN8 | 0.000511536 | 0.002105122 | 0.000760467 | 0.070600768 |
| ACTR3B | 0.000477818 | 0.002259319 | 0.000763684 | 0.06080982 |
| SCN5A | 0.000488045 | 0.002193234 | 0.000770909 | 0.080916774 |
| AGAP7 | 0.000471925 | 0.002648163 | 0.000796536 | 0.069509334 |
| LOC441958 | 0.000461879 | 0.003077233 | 0.000820262 | 0.093495462 |
| BICD1 | 0.000541323 | 0.00203808 | 0.000822879 | 0.089744399 |
| TSGA10IP | 0.000325179 | 0.003754358 | 0.000883957 | 0.080170513 |
| LOC440525 | 0.00053834 | 0.002661571 | 0.000887356 | 0.098177649 |
| C12orf65 | 0.000634228 | 0.002346474 | 0.000907242 | 0.063012779 |
| CLASP1 | 0.000651176 | 0.002353178 | 0.000909714 | 0.065555487 |
| PRDM10 | 0.000537514 | 0.002581121 | 0.000909908 | 0.066980081 |
| CDK5RAP2 | 0.00049701 | 0.003861625 | 0.000952442 | 0.09025404 |
| LOC650036 | 0.000604918 | 0.003548443 | 0.001046426 | 0.094060925 |
| GMEB2 | 0.000699749 | 0.002707543 | 0.001073495 | 0.067230176 |
| KCNH4 | 0.000470077 | 0.004290695 | 0.00109845 | 0.105867375 |
| PAFAH2 | 0.000749226 | 0.002587825 | 0.001122577 | 0.080228787 |
| DLX4 | 0.000692221 | 0.003841512 | 0.001137284 | 0.07745555 |
| ZBED5 | 0.000796309 | 0.002798529 | 0.001150015 | 0.091835538 |
| LOC648057 | 0.000699804 | 0.00334157 | 0.001165234 | 0.083204395 |
| GIYD2 | 0.000580478 | 0.00466613 | 0.00117724 | 0.10214365 |
| AMMECR1 | 0.000757918 | 0.004525342 | 0.001201243 | 0.104184893 |
| DERL1 | 0.000837455 | 0.003023599 | 0.001231722 | 0.098641407 |
| MIAT | 0.000788667 | 0.003567598 | 0.001276416 | 0.105156048 |
| AMY2B | 0.000585369 | 0.005215876 | 0.001280055 | 0.127383196 |
| MT1M | 0.00083796 | 0.003506302 | 0.001282021 | 0.102119456 |
| POLR2J4 | 0.00066539 | 0.005443819 | 0.001373001 | 0.10938615 |
| CCNE1 | 0.000917405 | 0.004391258 | 0.00146224 | 0.120066151 |
| LOC641710 | 0.000868361 | 0.004572271 | 0.001477216 | 0.113043767 |
| RAB37 | 0.000923114 | 0.004042639 | 0.001496755 | 0.096345649 |
| LOC643423 | 0.000824376 | 0.006047198 | 0.001542449 | 0.127812763 |
| PUM1 | 0.001030459 | 0.004505229 | 0.001592126 | 0.123405997 |
| FLJ23754 | 0.000880441 | 0.006087423 | 0.001618716 | 0.14483695 |
| GPHB5 | 0.000827719 | 0.005926522 | 0.001675163 | 0.125566084 |
| WWOX | 0.000973315 | 0.005701931 | 0.001746669 | 0.132118892 |
| C14orf166B | 0.001002289 | 0.006147761 | 0.001823292 | 0.131401725 |
| FAM27L | 0.001125418 | 0.007036548 | 0.002051799 | 0.180316413 |
| PMS2L11 | 0.001293818 | 0.006708999 | 0.002064562 | 0.135769103 |
