# Supplementary material for: Establishment of a Combined Diagnostic Model of Abdominal Aortic Aneurysm with Random Forest and Artificial Neural Network
Source: Biomed Res Int. 2022 Mar 7;2022:7173972. doi: 10.1155/2022/7173972 (PMC8922147; doi:10.1155/2022/7173972)
Supplement: Supplementary 6 — Supplementary Table 6: TF-mRNA Network. [file 7173972.f6.docx]

| Supplementary Table 6:TF-mRNA Network | |
| --- | --- |
| target | Gene |
| HOXB4 | ABI2, CDK5RAP2, DERL1, GMEB2, RAB27A, WWOX |
| TAL1 | ABI2, BAZ2A, C12orf65, CCNE1, CLASP1, CTBP1, CXXC5, DERL1, WWOX |
| WT1 | ABI2, BICD1, C12orf65, CXXC5, EDA, PUM1, VEZF1 |
| NANOG | ABI2, ACTR3B, AMMECR1, ARPP19, BAZ2A, C12orf65, CCNE1, CDK5RAP2, CHAT, CLASP1, CSNK2A2, CXXC5, DERL1, DHX40, DLX4, GMEB2, PRDM10, PUM1, RAB27A, RAB37, SCN5A, VEZF1, WWOX, ZBED5, ZNF331 |
| CREB1 | ABI2, ACTR3B, ARPP19, C12orf65, CCNE1, CLASP1, CTBP1, DERL1, DHX40, GMEB2, PAFAH2, SPAG5, VEZF1, ZNF331 |
| SPI1 | ABI2, ACTR3B, ARPP19, BAZ2A, BDKRB2, BICD1, CDK5RAP2, CLASP1, CSNK2A2, CTBP1, CXXC5, DERL1, GMEB2, POLR2J4, RAB27A, RAB37, ROCK1, SCN5A, SPAG5, WWOX, ZBED5 |
| ERG | ABI2, ARPP19, BICD1, C12orf65, CDK5RAP2, CLASP1, CLDN8, CTBP1, MT1M, PUM1, RAB27A, ROCK1, TSGA10IP, VEZF1 |
| ZNF281 | ABI2, CCNE1, WWOX |
| HOXD13 | ABI2, RAB37 |
| TBX3 | ABI2, AMMECR1, BDKRB2, CDK5RAP2, FAM19A4, PAFAH2, PDE3A, PRDM10, SCN5A |
| SIN3B | ABI2, ACTR3B, BAZ2A, CCNE1, CSNK2A2, CTBP1, CXXC5, DERL1, DHX40, OR4D1, PAFAH2, PUM1, ZBED5 |
| MECOM | ABI2, BAZ2A, CLASP1, CTBP1, DERL1, RAB27A, ROCK1, VEZF1 |
| MEIS1 | ABI2, KCNH4, SPAG5 |
| BACH1 | ABI2, BDKRB2, CLDN8, CTBP1, EDA, WWOX |
| KLF4 | ABI2, C12orf65, CCNE1, CDK5RAP2, CSNK2A2, DLX4, PAFAH2, PUM1, ROCK1, WWOX, ZBED5 |
| EOMES | ABI2, BICD1, VEZF1, ZNF331 |
| GFI1B | ABI2, ACTR3B, ARPP19, BICD1, C12orf65, CLASP1, CXXC5, PAFAH2, RAB27A, WWOX |
| EGR1 | ABI2, BAZ2A, BDKRB2, BICD1, CCNE1, CHAT, CLASP1, CLDN8, CSNK2A2, CTBP1, CXXC5, DERL1, DLX4, GMEB2, LOC149950, NEB, PDE3A, PPP4R1L, PRDM10, SCN5A, TSGA10IP, WWOX |
| SRY | ABI2, C12orf65, CSNK2A2, PUM1, RAB37, SCN5A |
| SOX2 | ABI2, ACTR3B, BAZ2A, BDKRB2, CCNE1, CDK5RAP2, CHAT, CLASP1, CLDN8, CNTN6, CSNK2A2, CXXC5, DERL1, DHX40, DLX4, EDA, GMEB2, LOC149950, PAFAH2, PPP4R1L, PRDM10, PUM1, RAB37, SCN5A, VEZF1, WWOX, ZBED5, ZNF331 |
| CREM | ABI2, ACTR3B, ARPP19, BAZ2A, C12orf65, CCNE1, CTBP1, CXXC5, DERL1, DHX40, GMEB2, PAFAH2, PPP4R1L, PUM1, ROCK1, SPAG5, VEZF1, WWOX |
| FOXO3 | ABI2, BAZ2A, BDKRB2, CDK5RAP2, CLASP1, CSNK2A2, DHX40, ROCK1, ZBED5, ZNF331 |
| TBX5 | ABI2, ACTR3B, BICD1, CDK5RAP2, CLASP1, DHX40, DLX4, RAB37, ROCK1, SPAG5, WWOX, ZBED5 |
| MYC | ABI2, ACTR3B, BICD1, C12orf65, CCNE1, CDK5RAP2, CLASP1, CLDN8, CNTN6, CSNK2A2, CTBP1, CXXC5, DHRS12, DHX40, DLX4, GMEB2, GPHB5, PAFAH2, PDE3A, PRDM10, PUM1, RAB27A, RAB37, VEZF1, WWOX, ZBED5 |
| RBPJ | ABI2, CLASP1, CXXC5, KCNH4 |
| DMRT1 | ABI2, BAZ2A, BDKRB2, CXXC5, DHX40, PRDM10, RAB37, ROCK1 |
| YY1 | ACTR3B, CDK5RAP2, PUM1 |
| REST | ACTR3B, AMY2B, BDKRB2, CHAT, CNTN6, CTBP1, CXXC5, DLX4, EDA, GMEB2, MIAT, OR4D1, RAB37, ZBED5 |
| STAT3 | ACTR3B, BDKRB2, BICD1, CCNE1, CDK5RAP2, CHAT, CNTN6, CSNK2A2, CXXC5, DHX40, FAM19A4, MIAT, OR4D1, PDE3A, PUM1, RAB27A, RAB37, ROCK1, SCN5A, SPAG5, WWOX |
| SMAD3 | ACTR3B, BICD1, CXXC5, DHRS12, GPHB5, POLR2J4, PRDM10, PUM1, RAB37, ZBED5 |
| NR3C1 | ACTR3B, BDKRB2, CDK5RAP2, CHAT, CLASP1, CLDN8, PDE3A |
| TCF4 | ACTR3B, BDKRB2, BICD1, CDK5RAP2, CNTN6, DLX4, FAM27L, GMEB2, GPHB5, NEB, PDE3A, POLR2J4, RAB37, ROCK1, WWOX |
| NFIB | ACTR3B, CDK5RAP2 |
| SMARCA4 | ACTR3B, BICD1, CDK5RAP2, DERL1, FAM19A4, GPHB5, PDE3A, VEZF1 |
| TET1 | ACTR3B, BAZ2A, BDKRB2, CLASP1, CSNK2A2, CTBP1, CXXC5, FAM19A4, GPHB5, PAFAH2, PUM1, RAB27A, RAB37, SCN5A |
| SMAD2 | ACTR3B, BICD1, CXXC5, DHRS12, POLR2J4, PRDM10, PUM1, ZBED5 |
| POU5F1 | ACTR3B, BAZ2A, CLASP1, CXXC5, DHX40, DLX4, FAM19A4, PAFAH2, PUM1, ROCK1, SCN5A, VEZF1, WWOX, ZBED5 |
| PPARD | ACTR3B, AMMECR1, BAZ2A, BDKRB2, BICD1, CDK5RAP2, CLASP1, CNTN6, MIAT, NEB, ROCK1, ZBED5, ZNF331 |
| RNF2 | ACTR3B, CHAT, DLX4, FAM19A4, PDE3A, SCN5A |
| ESRRB | ACTR3B, BAZ2A, DLX4, PDE3A, SCN5A, WWOX |
| SMAD4 | ACTR3B, AMMECR1, BICD1, CDK5RAP2, CLASP1, CNTN6, GPHB5, PAFAH2, RAB37, ROCK1, ZBED5 |
| TCF3 | ACTR3B, AMMECR1, CDK5RAP2, CXXC5, PAFAH2, PUM1, SCN5A, ZBED5 |
| YAP1 | AMMECR1, BICD1, CDK5RAP2, CNTN6, DHX40, DLX4, PDE3A, WWOX |
| TFAP2C | AMMECR1, BAZ2A, CXXC5, GMEB2, VEZF1, WWOX, ZNF331 |
| SETDB1 | AMMECR1, ARPP19, BAZ2A, BICD1 |
| SOX17 | AMMECR1, CDK5RAP2, DERL1, EDA, PAFAH2, RAB27A |
| E2F1 | AMMECR1, BAZ2A, CCNE1, CLASP1, CLDN8, CTBP1, CXXC5, DHX40, GPHB5, OR4D1, PDE3A, PRDM10, PUM1, RAB27A, WWOX, ZBED5, ZNF331 |
| CEBPD | AMY2B, BICD1, GMEB2 |
| POU3F2 | AMY2B, BICD1, CLDN8, PRDM10, ROCK1 |
| KLF1 | AMY2B, ARPP19, CCNE1, CSNK2A2, CTBP1, DERL1 |
| PBX1 | ARMCX4, EDA, GPHB5, WWOX |
| ELF5 | ARMCX4, BAZ2A, ROCK1, ZBED5 |
| PPARG | ARPP19, BAZ2A, BICD1, CLASP1, CSNK2A2, CTBP1, DERL1, DHX40, NEB, PAFAH2, PPP4R1L, PUM1, RAB37, ROCK1, WWOX |
| ZFX | ARPP19, BICD1, CSNK2A2, CTBP1, DERL1, GMEB2, PUM1 |
| ESR1 | ARPP19, CHAT, DHX40, PDE3A |
| RUNX1 | ARPP19, CLASP1, CTBP1, CXXC5, DERL1, GMEB2, NEB, PAFAH2, PPP4R1L, PRDM10, PUM1, RAB37, VEZF1, WWOX, ZBED5, ZNF331 |
| ETS1 | ARPP19, CDK5RAP2, CLASP1, DERL1, MIAT, PAFAH2, ZNF331 |
| FOXP2 | ARPP19, CTBP1, PAFAH2, PUM1 |
| SMAD1 | ARPP19, GMEB2, SCN5A |
| FLI1 | ARPP19, BAZ2A, CLASP1, CXXC5, DERL1, MIAT, OR4D1, PAFAH2, PDE3A, RAB37, WWOX, ZNF331 |
| HOXC9 | ARPP19, PAFAH2, POLR2J4, PRDM10 |
| TP63 | ARPP19, BDKRB2, CDK5RAP2, CHAT, CLASP1, CTBP1, DHRS12, DLX4, MIAT, MT1M, NEB, PAFAH2, POLR2J4, PPP4R1L, PUM1, ROCK1, SCN5A, TSGA10IP, VEZF1, WWOX, ZBED5 |
| E2F4 | ARPP19, BICD1, CDK5RAP2, CHAT, DHRS12, PRDM10, PUM1, RAB27A, SPAG5, WWOX, ZNF331 |
| TBP | BAZ2A |
| FOXP1 | BAZ2A, DHX40, PAFAH2, PUM1, ROCK1 |
| GATA3 | BAZ2A, CTBP1, DLX4 |
| AR | BAZ2A, BICD1, CCNE1, CDK5RAP2, CHAT, CLASP1, CLDN8, CTBP1, DERL1, DHRS12, EDA, FAM27L, NEB, OR4D1, PAFAH2, PPP4R1L, PUM1, RAB27A, ROCK1, SCN5A, TSGA10IP, VEZF1, WWOX, ZBED5 |
| PADI4 | BAZ2A, VEZF1 |
| MYBL2 | BAZ2A, CDK5RAP2, RAB27A, WWOX |
| SRF | BAZ2A, BICD1, C12orf65, DHX40, PAFAH2, RAB27A |
| TEAD4 | BAZ2A, C12orf65, CDK5RAP2, CHAT, DHX40, PAFAH2, PDE3A |
| GATA4 | BAZ2A, CCNE1, CDK5RAP2, CLASP1, CTBP1, CXXC5, DHX40, PAFAH2 |
| SREBF2 | BAZ2A, DERL1, ROCK1 |
| TFCP2L1 | BAZ2A, BICD1, C12orf65, CTBP1, CXXC5, WWOX |
| NR0B1 | BAZ2A, CCNE1, PAFAH2, WWOX |
| CUX1 | BAZ2A, CSNK2A2, CTBP1, NEB, PAFAH2, PUM1, RAB27A, RAB37, SPAG5, VEZF1 |
| TFAP2A | BAZ2A, BDKRB2, CCNE1, GMEB2, OR4D1, ZNF331 |
| KDM5B | BAZ2A, CCNE1, CDK5RAP2, CSNK2A2, CTBP1, CXXC5, DHX40, PUM1, SCN5A, SPAG5, VEZF1, WWOX |
| PRDM14 | BDKRB2, CCNE1, CDK5RAP2, CLASP1, CXXC5, DHX40, PUM1 |
| CTNNB1 | BDKRB2, CTBP1, CXXC5, DLX4, FAM19A4, PDE3A, SCN5A, ZBED5 |
| RELA | BDKRB2, CXXC5, PAFAH2, RAB27A, VEZF1 |
| RCOR3 | BDKRB2, C12orf65, CCNE1, CTBP1, CXXC5, DERL1, DHX40, MIAT, OR4D1, RAB37 |
| ESR2 | BDKRB2, RAB37 |
| EWSR1 | BDKRB2 |
| SUZ12 | BDKRB2, CHAT, DLX4, FAM19A4, KCNH4, MIAT, PDE3A, SCN5A, VEZF1 |
| TRIM28 | BICD1, CSNK2A2, FAM19A4, GMEB2, KCNH4, PDE3A, PUM1, ROCK1, WWOX |
| SCLY | BICD1, CCNE1, CXXC5, PDE3A, WWOX |
| RUNX2 | BICD1, CLASP1, EDA, FAM27L, GPHB5, MIAT, PRDM10, RAB27A, ROCK1, WWOX |
| GATA1 | BICD1, CDK5RAP2, CLASP1, CTBP1, CXXC5, MIAT, OR4D1, PUM1, RAB37 |
| GATA2 | BICD1, CLASP1, CXXC5, DLX4, KCNH4, MIAT, PAFAH2, PPP4R1L, RAB27A, WWOX, ZBED5, ZNF331 |
| NFE2L2 | BICD1, CDK5RAP2, CLASP1, DHX40 |
| XRN2 | BICD1, CLASP1, PUM1 |
| PHF8 | BICD1, ROCK1, ZBED5 |
| TP53 | BICD1, C12orf65, CNTN6, DLX4, KCNH4, MIAT, PAFAH2, PDE3A, PUM1, RAB37, WWOX, ZBED5 |
| HNF4A | BICD1, CDK5RAP2, CLASP1, CXXC5, DLX4, GMEB2, NEB, PAFAH2, PDE3A, PRDM10, PUM1, RAB27A, RAB37, TSGA10IP, VEZF1, WWOX, ZNF331 |
| MYCN | C12orf65, CCNE1, CHAT, CLASP1, SPAG5 |
| SOX9 | C12orf65, CLDN8, FAM19A4, PUM1, RAB27A, SCN5A, SPAG5, WWOX |
| CCND1 | C12orf65, CTBP1, DERL1, PAFAH2, ROCK1 |
| NR1I2 | C12orf65, PUM1, WWOX |
| NUCKS1 | CCNE1, CTBP1, ROCK1 |
| ASH2L | CCNE1, CXXC5, DERL1, DHX40, FAM19A4 |
| CNOT3 | CCNE1, GMEB2, ROCK1 |
| SIN3A | CCNE1, CSNK2A2, DHX40, ZBED5 |
| FOXA2 | CDK5RAP2, CLASP1, CXXC5, DHX40, PRDM10, RAB37, TSGA10IP, VEZF1, WWOX |
| SOX11 | CDK5RAP2, PUM1 |
| PRDM5 | CDK5RAP2 |
| MITF | CDK5RAP2, CLASP1, CNTN6, CTBP1, CXXC5, DHX40, EDA, GMEB2, MIAT, PDE3A, PRDM10, PUM1, RAB27A, SCN5A, VEZF1 |
| NR1H3 | CDK5RAP2, CSNK2A2, CXXC5, GMEB2, MT1M, PAFAH2 |
| AP1S2 | CDK5RAP2, CLASP1, PAFAH2, ZNF331 |
| EP300 | CDK5RAP2, CLASP1, PRDM10, SCN5A |
| KDM5A | CDK5RAP2, CLASP1, DHX40, GMEB2 |
| SALL4 | CDK5RAP2, CXXC5, EDA, PUM1, RAB37 |
| OLIG2 | CDK5RAP2, CNTN6, DERL1, FAM19A4, GPHB5, PDE3A, ROCK1 |
| ZFP42 | CDK5RAP2 |
| TTF2 | CDK5RAP2, DHX40, DLX4, PAFAH2, PRDM10, PUM1, RAB37, ZBED5 |
| NR4A2 | CDK5RAP2 |
| ZNF322 | CDK5RAP2, WWOX |
| EZH2 | CDK5RAP2, CHAT, DLX4, MIAT, PDE3A, SCN5A |
| VDR | CDK5RAP2, DERL1, DHX40, PAFAH2, PRDM10, RAB37, ROCK1, SPAG5, WWOX, ZBED5 |
| JARID2 | CHAT, DLX4, GMEB2, PDE3A, SCN5A |
| MTF2 | CHAT, DLX4, PDE3A, SCN5A |
| IRF8 | CLASP1, VEZF1 |
| FOXP3 | CLASP1, DERL1, DHX40, PAFAH2, PRDM10, WWOX |
| PAX3 | CLASP1, PDE3A, WWOX, ZNF331 |
| MEF2A | CLASP1, PAFAH2 |
| ASXL1 | CLASP1 |
| CEBPB | CLASP1, CSNK2A2, RAB37 |
| PAX6 | CLDN8, CTBP1, PAFAH2 |
| STAT5A | CLDN8, PAFAH2, WWOX |
| TCF7 | CSNK2A2, CXXC5 |
| ELF1 | CTBP1 |
| DACH1 | CTBP1, GMEB2, PPP4R1L, PRDM10, VEZF1 |
| RCOR1 | CTBP1, DLX4, SPAG5, VEZF1 |
| ZNF217 | CXXC5, DHX40, VEZF1, WWOX |
| LYL1 | CXXC5, PAFAH2 |
| CDX2 | CXXC5, GMEB2, WWOX |
| LMO2 | CXXC5, PAFAH2, RAB37 |
| BCL3 | CXXC5, KCNH4 |
| THAP11 | DERL1 |
| PDX1 | DERL1 |
| NOTCH1 | DERL1, PAFAH2, ZNF331 |
| ATF3 | DHRS12, DLX4, PAFAH2, VEZF1, ZBED5 |
| CRX | DHX40 |
| DCP1A | DHX40, POLR2J4 |
| DNAJC2 | DLX4 |
| BMI1 | DLX4 |
| ZNF652 | EDA |
| CTCF | FAM19A4, RAB27A, RAB37 |
| JUN | FAM19A4, FAM27L, ROCK1 |
| GBX2 | FAM19A4, NEB |
| STAT6 | GMEB2, PPP4R1L, VEZF1 |
| AHR | MIAT |
| ELK1 | MIAT, PUM1 |
| ARNT | MIAT, RAB37 |
| SREBF1 | PAFAH2 |
| MYB | PAFAH2, RAB27A, RAB37 |
| STAT1 | PAFAH2 |
| RAD21 | PAFAH2, RAB27A, WWOX |
| EED | PDE3A |
| STAT4 | RAB27A, RAB37 |
| FOXM1 | SPAG5 |
| DROSHA | VEZF1 |
| ETS2 | WWOX |
| HTT | WWOX |
| TCF7L2 | WWOX |
| HSF1 | ZBED5 |
| HIF1A | ZNF331 |
