# Supplementary material for: Establishment of a Combined Diagnostic Model of Abdominal Aortic Aneurysm with Random Forest and Artificial Neural Network
Source: Biomed Res Int. 2022 Mar 7;2022:7173972. doi: 10.1155/2022/7173972 (PMC8922147; doi:10.1155/2022/7173972)
Supplement: Supplementary 7 — Supplementary Table 7: WGCNA Correlation Table. [file 7173972.f7.docx]

| Supplementary Table 7. WGCNA Correlation Table (>0.1) | | |
| --- | --- | --- |
| Source | Target | Correlation |
| ZBED5 | ARPP19 | 0.106041443 |
| ZBED5 | CTBP1 | 0.16831803 |
| ZBED5 | C12orf65 | 0.156616754 |
| VEZF1 | PUM1 | 0.226961149 |
| VEZF1 | CXXC5 | 0.110536911 |
| VEZF1 | CLASP1 | 0.120549143 |
| VEZF1 | CSNK2A2 | 0.135520569 |
| PUM1 | CLASP1 | 0.117606316 |
| PUM1 | CSNK2A2 | 0.12831289 |
| CLASP1 | CSNK2A2 | 0.156706146 |
