# Supplementary material for: Establishment of a Combined Diagnostic Model of Abdominal Aortic Aneurysm with Random Forest and Artificial Neural Network
Source: Biomed Res Int. 2022 Mar 7;2022:7173972. doi: 10.1155/2022/7173972 (PMC8922147; doi:10.1155/2022/7173972)
Supplement: Supplementary 8 — Supplementary Table 8: ANN in GSE57691 dataset. [file 7173972.f8.docx]

| Supplementary Table 8. ANN in GSE57691 | | |
| --- | --- | --- |
|  | AAA | Control |
| GSM1386783 | 0.988839495 | 0.00398816 |
| GSM1386784 | 0.986898546 | 0.005785013 |
| GSM1386785 | 0.988851909 | 0.003975559 |
| GSM1386786 | 0.986922769 | 0.005757552 |
| GSM1386787 | 0.986494071 | 0.00622906 |
| GSM1386788 | 0.988859484 | 0.003969521 |
| GSM1386789 | 0.988857062 | 0.003971551 |
| GSM1386790 | 0.988724709 | 0.004078259 |
| GSM1386791 | 0.988788916 | 0.004028444 |
| GSM1386792 | 0.988327654 | 0.004421959 |
| GSM1386793 | 0.988848514 | 0.003978873 |
| GSM1386794 | 0.988840384 | 0.003985591 |
| GSM1386795 | 0.988850874 | 0.003976737 |
| GSM1386796 | 0.988858079 | 0.003970607 |
| GSM1386797 | 0.988857695 | 0.003970872 |
| GSM1386798 | 0.988546161 | 0.004244057 |
| GSM1386799 | 0.988858989 | 0.003969959 |
| GSM1386800 | 0.988860955 | 0.003968254 |
| GSM1386801 | 0.988859141 | 0.003969723 |
| GSM1386802 | 0.988798463 | 0.004021445 |
| GSM1386803 | 0.98885787 | 0.003970894 |
| GSM1386804 | 0.987055658 | 0.00561643 |
| GSM1386805 | 0.988858758 | 0.003970087 |
| GSM1386806 | 0.986821754 | 0.005862898 |
| GSM1386807 | 0.987481857 | 0.005203008 |
| GSM1386808 | 0.987368218 | 0.005311798 |
| GSM1386809 | 0.986701198 | 0.005989071 |
| GSM1386810 | 0.987283416 | 0.005397505 |
| GSM1386811 | 0.986575409 | 0.006125531 |
| GSM1386812 | 0.988858756 | 0.003970082 |
| GSM1386813 | 0.988853428 | 0.003974529 |
| GSM1386814 | 0.987149097 | 0.005529021 |
| GSM1386815 | 0.986908459 | 0.005774315 |
| GSM1386816 | 0.987900576 | 0.004808401 |
| GSM1386817 | 0.988858912 | 0.003969995 |
| GSM1386818 | 0.98886039 | 0.00396874 |
| GSM1386819 | 0.988836053 | 0.003988684 |
| GSM1386820 | 0.988745889 | 0.004067019 |
| GSM1386821 | 0.988845698 | 0.003980956 |
| GSM1386822 | 0.988856804 | 0.003971805 |
| GSM1386823 | 0.988560188 | 0.004231891 |
| GSM1386824 | 0.988795242 | 0.004024136 |
| GSM1386825 | 0.988857995 | 0.003970765 |
| GSM1386826 | 0.98879001 | 0.004029147 |
| GSM1386827 | 0.98864733 | 0.004154928 |
| GSM1386828 | 0.988836171 | 0.003988507 |
| GSM1386829 | 0.9885105 | 0.004279267 |
| GSM1386830 | 0.98885367 | 0.003974191 |
| GSM1386831 | 0.988840966 | 0.003984798 |
| GSM1386841 | 0.020948903 | 0.990055432 |
| GSM1386842 | 0.020949822 | 0.990054767 |
| GSM1386843 | 0.021018038 | 0.990008663 |
| GSM1386844 | 0.020949674 | 0.990054894 |
| GSM1386845 | 0.020962963 | 0.990041738 |
| GSM1386846 | 0.020948923 | 0.990055408 |
| GSM1386847 | 0.020948936 | 0.990055396 |
| GSM1386848 | 0.020958687 | 0.990045234 |
| GSM1386849 | 0.02094905 | 0.990055287 |
| GSM1386850 | 0.02094899 | 0.990055342 |
