# Supplementary material for: Establishment of a Combined Diagnostic Model of Abdominal Aortic Aneurysm with Random Forest and Artificial Neural Network
Source: Biomed Res Int. 2022 Mar 7;2022:7173972. doi: 10.1155/2022/7173972 (PMC8922147; doi:10.1155/2022/7173972)
Supplement: Supplementary 9 — Supplementary Table 9: ANN in GSE47472 dataset. [file 7173972.f9.docx]

| Supplementary Table 9. ANN in GSE47472 | | |
| --- | --- | --- |
|  | AAA | Control |
| GSM1150689 | 0.988657943 | 0.004099168 |
| GSM1150690 | 0.062397473 | 0.918349324 |
| GSM1150691 | 0.953351885 | 0.012982714 |
| GSM1150692 | 0.081413821 | 0.976056514 |
| GSM1150693 | 0.030331916 | 0.980105262 |
| GSM1150694 | 0.980649598 | 0.012112565 |
| GSM1150695 | 0.974154571 | 0.01037504 |
| GSM1150696 | 0.021986809 | 0.989017667 |
| GSM1150697 | 0.981909827 | 0.007124717 |
| GSM1150698 | 0.987668041 | 0.004830589 |
| GSM1150699 | 0.979174654 | 0.010217876 |
| GSM1150700 | 0.02667953 | 0.986483907 |
| GSM1150701 | 0.03747016 | 0.984654097 |
| GSM1150702 | 0.042931631 | 0.980547703 |
| GSM1150703 | 0.98864807 | 0.004105678 |
| GSM1150704 | 0.988658621 | 0.004113986 |
| GSM1150705 | 0.976315965 | 0.012027672 |
| GSM1150706 | 0.987537653 | 0.005074303 |
| GSM1150707 | 0.988639538 | 0.004121381 |
| GSM1150708 | 0.687772489 | 0.215035683 |
| GSM1150709 | 0.981091655 | 0.006787358 |
| GSM1150710 | 0.988400055 | 0.004284873 |
